# Chemodivergent C-to-N atom swap from benzofurans to benzisoxazoles and benzoxazoles†

**DOI:** 10.1039/d5sc02032h

**Published:** 2025-05-27

**Authors:** Ann-Sophie K. Paschke, Stefanie Schiele, Camille Pinard, Filippo Sandrini, Bill Morandi

**Affiliations:** a Laboratorium fur Organische Chemie, ETH Zurich Vladimir-Prelog-Weg 3, HCI 8093 Zurich Switzerland morandib@ethz.ch

## Abstract

Facile derivatization of biologically active compounds without prefunctionalization expands the chemical space and accelerates the discovery of new molecules. Atom swap reactions have recently emerged as powerful molecular editing tools, yet such reactions remain rare. Herein, we describe a convenient, chemodivergent protocol to perform a net C-to-N atom swap in benzofurans, affording benzoxazoles or benzisoxazoles *via* a cascade of oxidative cleavage, oxime formation, and cyclization using commercially available reagents.

## Abstract 2

We present a chemodivergent net C-to-N atom swap in benzofurans, affording benzoxazoles or benzisoxazoles *via* photo-mediated oxidative benzofuran cleavage, followed by oxime or imine formation, and cyclization using commercially available reagents.

## Graphical abstract

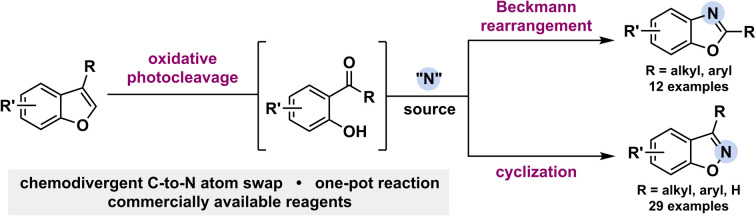

## Introduction

**Fig. 1:**
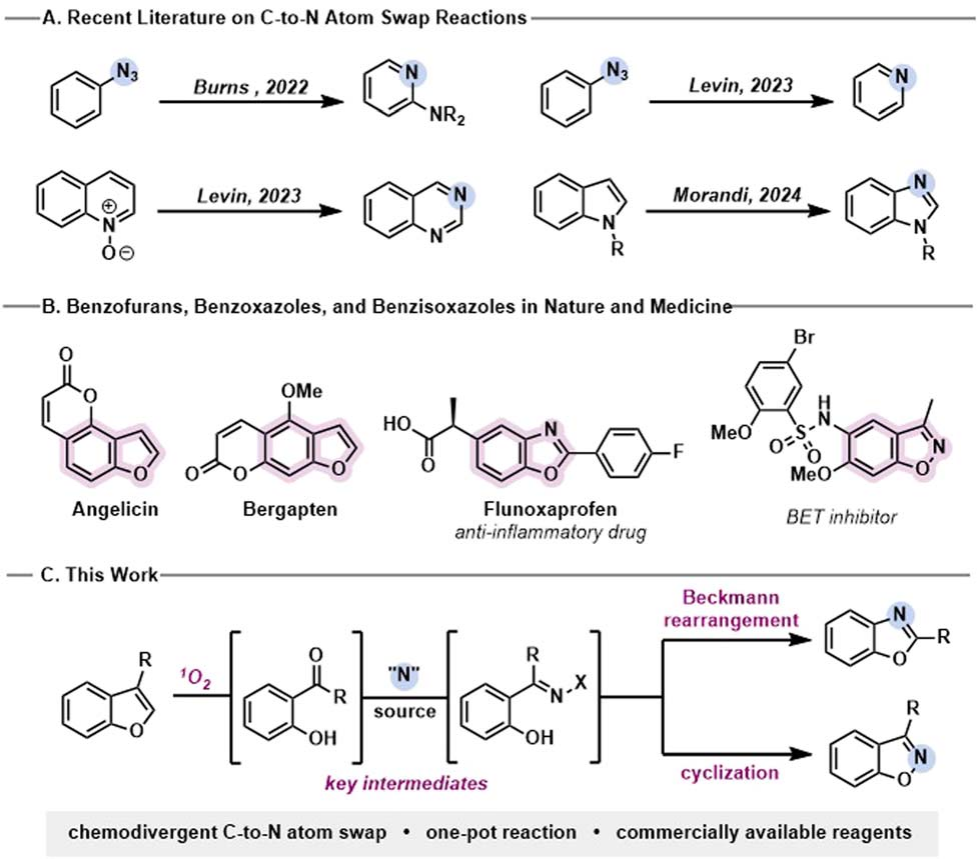
(A) Recent reports on C-to-N atom swap methodologies. (B) Presence of benzofuran, benzoxazole, and benzisoxazole scaffolds in natural products and drugs. (C) Herein described chemodivergent C-to-N atom swap.

The precise editing of the molecular skeleton has recently emerged as an alternative to traditional peripheral transformations. Insertion^1–6^ and deletion^7–9^ of (hetero)atoms allow for rapid access to novel compounds with altered bioactivity profiles, thus expanding the existing chemical space. As a third class of skeletal modifications, single-atom swap reactions have gained increasing interest in the scientific community due to their value in tuning the pharmacological properties of lead compounds without the need for time-consuming *de novo* synthesis, all while preserving the molecule's topology. Recently, several methods to perform single-atom swaps have been disclosed.^10–15^ However, C-to-N atom swaps remain scarce and require prefunctionalization,^10–13^ limiting their applicability to less complex frameworks (Fig. 1A).

We recently reported a strategy to leverage the innate reactivity of indoles to perform a rare C-to-N atom swap to benzimidazoles.^16^ Benzofurans are another class of attractive heterocycles for such a transformation, as they are ubiquitous in natural products and pharmaceuticals (Fig. 1B).^17^ Replacing a carbon with a nitrogen atom in the benzofuran core would thus facilitate chemical space exploration around this valuable motif. Unfortunately, the method we previously developed for indole editing using hypervalent iodine-mediated cleavage and Hofmann-type rearrangement could not be extended to benzofurans, calling for a new approach.^16^

Herein, we describe a facile, chemodivergent one-pot method to transform 3-substituted benzofurans to benzoxazoles or benzisoxazoles as well as benzofurans to benzisoxazoles, using commercially available reagents (Fig. 1C).§§While finalizing this manuscript, the Studer group independently reported a similar design to perform C-to-N atom swaps.^18^ Key to the reaction's success was the combination of a photo-mediated oxidative benzofuran cleavage with suitable electrophilic nitrogen sources in a one-pot sequential protocol.

## Results and discussion

**Fig. 2:**
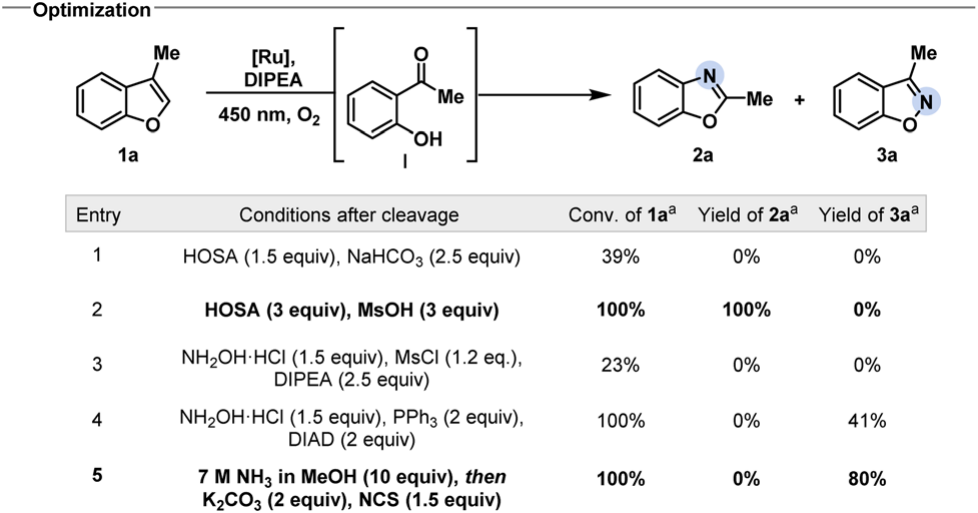
Optimization of reaction conditions to selectively access 3-methyl benzoxazoles or benzisoxazoles. ^*a*^Refers to ^1^H NMR yield determined with mesitylene as internal standard and 2-hydroxyacetophenone as the starting material.

To realize the desired net C-to-N atom swap in benzofurans, we envisioned a cascade of oxidative cleavage, intermediate oxime or imine formation, and cyclization (Fig. 2). While different methods to oxidatively cleave indoles have been extensively studied, the cleavage of benzofurans to the corresponding carbonyl compounds usually requires harsh conditions.^19^

We discovered that a ruthenium photocatalyst enables the oxidative cleavage of the C2–C3 bond of benzofuran in the presence of oxygen, inspired by previous reports on the photocleavage of indoles.^21^ Control experiments without light, without photocatalyst, or under inert atmosphere indicated that the oxidative cleavage likely proceeds *via* singlet oxygen formation (see ESI† for detail). While the cleavage of benzofurans with singlet oxygen had been studied before – mostly postulating a dioxetane as an intermediate – the synthetic value for remodelling the molecular skeleton has not been exploited yet.^20^ Having established a successful and general oxidative cleavage method, we next focused on the ring-closing sequence (Fig. 2). We initially aimed to develop conditions for the synthesis of benzoxazoles starting from 2-hydroxyacetophenone I, which can be easily obtained from the photocleavage of 3-methyl benzofuran 1a. Gratifyingly, under acidic conditions, the treatment with hydroxylamine-*O*-sulfonic acid (HOSA) afforded benzoxazoles *via* a Beckmann rearrangement from the intermediate oxime followed by cyclization (Fig. 2, entry 2).^22^ We next targeted the corresponding 3-substituted benzisoxazoles from the same starting material, as this would provide a powerful chemodivergent atom-swap tool for synthetic practitioners. We discovered that the *N*-chloro imine, accessed *via* oxidation of the *in situ* generated imine by *N*-chlorosuccinimide (NCS), favoured the desired direct cyclization over rearrangement under basic conditions (Fig. 2, entry 5).^23^

**Fig. 3:**
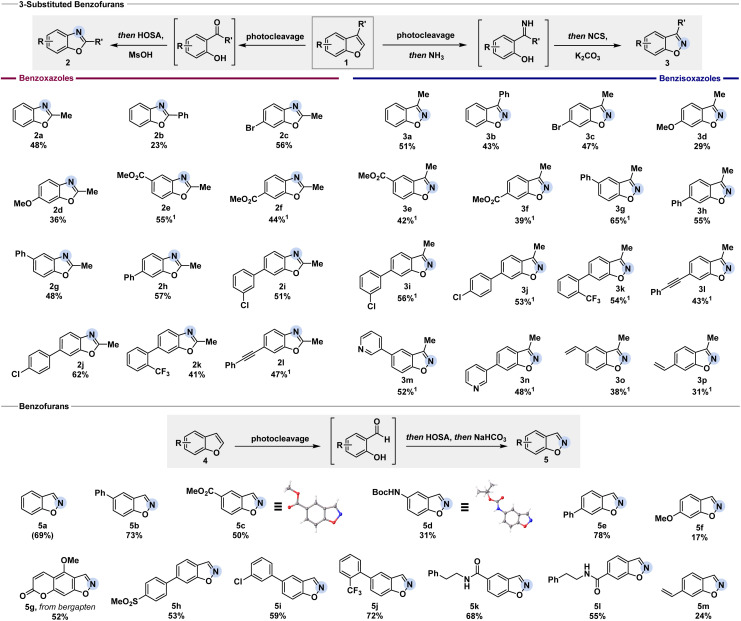
C-to-N atom swap of benzofurans to benzoxazoles and benzisoxazoles. ^1^Refers to products isolated from reactions on 0.1 mmol scale. Yield in brackets refers to ^1^H NMR yield determined with mesitylene as internal standard. Reaction conditions for benzoxazole 2: Ru(phen)_3_Cl_2_·*x*H_2_O (1.5 mol%), DIPEA (0.5 equiv.), ACN (0.1 M), 450 nm, O_2_, 35 °C, 16 h. 2. HOSA (3.0 equiv.), MsOH (3.0 equiv.), ACN (0.1 M), 90 °C, 3 h. Reaction conditions for benzisoxazole 3: Ru(phen)_3_Cl_2_·*x*H_2_O (1.5 mol%), DIPEA (0.5 equiv.), ACN (0.1 M), 450 nm, O_2_, 35 °C, 16 h. 2.7 M NH_3_ in MeOH (10 equiv.), rt, 3 h. 3. NCS (1.5 equiv.), K_2_CO_3_ (2.0 equiv.), THF (0.1 M), rt, 16 h. Reaction conditions for benzisoxazole 5 : 1. Ru(phen)_3_Cl_2_·*x*H_2_O (3 mol%), DIPEA (0.2 equiv.), H_2_O (60 equiv.), ACN (0.4 M), 450 nm, O_2_, 35 °C, 16 h. 2. HOSA (1.5 equiv.), ACN : H_2_O (1 : 1, 0.2 M), 0 °C, 1 h. 3. NaHCO_3_ (2.5 equiv.), ACN : H_2_O (1 : 1, 0.2 M), rt, 1 h.

Notably, both protocols can be performed as user-friendly one-pot processes starting directly from the corresponding benzofurans. With the optimized reaction conditions in hand, we set out to convert a series of 3-substituted benzofurans into benzoxazoles and benzisoxazoles (Fig. 3). Electron-withdrawing and -donating groups in the 5- and 6-position were well-tolerated as showcased by the successful conversion of substrates 1c to 1n. Halogens, such as chloro- (2i, 2j, 3i, and 3j) and bromo-substituents (2c and 3c), performed well for benzoxazole and benzisoxazole formation. Alkynes remained untouched, giving the desired benzoxazole 2l or benzisoxazole 3l in 47% or 43% yield, respectively. Generally, the initial oxidative cleavage also tolerated 3-phenyl substituted benzofurans (1b). The low yield for benzoxazole 2b (23%) was assigned to the competition in migration between the 2-hydroxybenzene ring and phenyl group in the subsequent Beckmann rearrangement. In contrast, the respective benzisoxazole formation was not influenced. By using NCS as a chlorinating agent to activate the previously formed N–H ketimine and treating it with base to facilitate the N–O bond formation, benzisoxazole 3b was obtained in 43% yield. When the reaction was performed with 3-isopropyl or 3-benzyl benzofuran, the cleavage was successful. However, neither benzoxazole nor benzisoxazole formation was observed, presumably due to increased steric bulk (see ESI† for detail).

To further examine the applicability of our methods, we tested benzofurans without a substituent in the 3-position. Treatment with HOSA under basic conditions after the oxidative cleavage gave access to the corresponding benzisoxazole.^24^ Minor re-optimization was needed (see ESI† for detail) to successfully transform various benzofurans as depicted in Fig. 3. After the oxidative cleavage of the benzofuran, we propose the formation of an oxime-*O*-sulfonate intermediate by the addition of HOSA. Basic conditions facilitate the subsequent ring-closing, affording the respective benzisoxazole. Incomplete photocleavage led to the isolation of remaining starting material, as noted below. Esters (5c), carbamates (5d), amides (5k and 5l), and alkenes (5m) were well-tolerated. The structure of the products 5c and 5d was unambiguously confirmed by single-crystal X-ray analysis. Free aniline and bromo substituents in 5-position were not tolerated (see ESI† for detail). Sulfone groups did not interfere with the reaction, giving the desired product 5h in 53% yield. Chloro- and trifluoromethyl groups gave the benzisoxazoles 5i and 5j in 59% and 72% yield, respectively. We were further pleased to see that the reaction performs well with more complex substrates such as bergapten, giving the corresponding benzisoxazoles 5g in 52% yield.

## Conclusions

In conclusion, we demonstrated the efficient chemodivergent conversion of benzofurans to benzoxazoles or benzisoxazoles *via* C-to-N atom swap. Given the user-friendly nature and broad range of tolerated functional groups, we believe that this reaction will find immediate utility in both academic and industrial settings.

## Data availability

X-ray data for compounds are freely available at the Cambridge Crystallographic Data Centre under deposition CCDC 2418538–2418540.

## Author contributions

A.-S. K. P. conceived the project. A.-S. K. P., S. S., C. P., and F. S. conducted the experimental work and analysed the data. B. M. supervised the research. A.-S. K. P., S. S., and B. M. wrote the manuscript with input from all authors.

## Conflicts of interest

There are no conflicts to declare.

## Supplementary Material

SC-OLF-D5SC02032H-s001

SC-OLF-D5SC02032H-s002
