# Supplementary material for: Chemodivergent C-to-N atom swap from benzofurans to benzisoxazoles and benzoxazoles
Source: Chem Sci. 2025 May 27;16(25):11464–7. doi: 10.1039/d5sc02032h (PMC12109606; doi:10.1039/d5sc02032h)
Supplement: SC-016-D5SC02032H-s001 [file SC-016-D5SC02032H-s001.pdf]

Supplementary Information (SI) for Chemical Science.
This journal is © The Royal Society of Chemistry 2025

Supporting Information for

Chemodivergent C-to-N atom swap from benzofurans to benzisoxazoles and benzoxazoles

Ann-Sophie K. Paschke†[a], Stefanie Schiele†[a], Camille Pinard[a], Fillippo Sandrini[a], Bill Morandi*[a]

† These authors contributed equally.

[a]Laboratorium für Organische Chemie, ETH Zürich, 8093 Zürich, Switzerland

*Corresponding author. Email: bill.morandi@org.chem.ethz.ch

Content

General Information ... 2
Reaction Optimization ... 3
Reaction Optimization with 3-Methyl Benzofuran or Methyl 3-Methylbenzofuran-5-Carboxylate as Starting Material ... 3
Optimization of the Oxidative Cleavage with 3-Methyl Benzofuran as Starting Material ... 3
Optimization of the Oxidative Cleavage with Methyl 3-Methylbenzofuran-5-Carboxylate as Starting Material ... 3
Optimization of the Benzoxazole Formation ... 5
Optimization of the Benzisoxazole Formation ... 5
Reaction Optimization with Benzofuran as Starting Material ... 7
Optimization of the Oxidative Cleavage with Benzofuran as Starting Material ... 7
Optimization of Benzisoxazole formation ... 10
Preparation of Starting Materials ... 11
General Procedure A for the Synthesis of Alkylated 2-Iodophenol ... 11
General Procedure B for the Synthesis of Benzofurans ... 11
General Procedure C for the Synthesis of Benzofurans ... 11
Synthesis of Alkylated 2-Iodophenol ... 11
Synthesis of Benzofurans ... 14
Synthesis of 3-substituted Benzofurans ... 17
C-to-N Atom Swap ... 22
General Procedure D to Access Benzisoxazoles from Benzofurans ... 22
General Procedure E to Access Benzoxazoles from 3-Substituted Benzofurans ... 23
General Procedure F to Access Benzisoxazoles from 3-Substituted Benzofurans ... 23
Benzisoxazoles from Benzofurans ... 23
Benzoxazoles from 3-Substituted Benzofurans ... 27
Benzisoxazoles from 3-Substituted Benzofurans ... 31
Failed Substrates ... 36
NMR Spectra of Starting Materials ... 37
NMR Spectra of Alkylated Iodophenols ... 37
NMR Spectra of Benzofurans ... 43
NMR Spectra of 3-substituted Benzofurans ... 54
NMR Spectra of Products ... 71
NMR Spectra of Benzisoxazoles from Benzofurans ... 71
NMR Spectra of Benzoxazoles from 3-Substituted Benzofurans ... 85
NMR Spectra of Benzisoxazoles from 3-Substituted Benzofurans ... 98
Crystallographic Data ... 115
References ... 118

General Information

Materials: Unless otherwise stated, reagents were used as supplied from commercial sources without any further purification. Tris(2,2′-bipyridyl)ruthenium(II) chloride hexahydrate (CAS: 50525-27-4, MW = 748.6 g/mol) and tris(1,10-phenanthroline)ruthenium dichloride (CAS: 207802-45-7, MW = 712.6 g/mol), hydroxylamine-*O*-sulfonic acid (HOSA, CAS: 2950-43-8, MW = 113.09 g/mol), and methanesulfonic acid (MsOH, CAS: 75-75-2, MW = 96.10 g/mol) were purchased from Sigma Aldrich. *N,N*-Diisopropylethylamine (DIPEA, CAS: 7087-68-5, MW = 129.25 g/mol) was purchased from abcr. 7 M NH_3 in methanol (CAS: 7664-41-7) and N-chlorosuccinimide (NCS, CAS: 128-09-6, MW = 133.53 g/mol) were purchased from Thermo Scientific Chemicals.

NMR: ^{1}H- and ^{13}C-NMR spectra were recorded on a Bruker AVIII 400 MHz, a Bruker Neo 400 MHz or a Bruker Neo 500 MHz spectrometer and are reported in parts per million (ppm). ^{1}H-NMR spectra are calibrated with respect to the corresponding solvent residual peak ($CHCl_3$: 7.26 ppm; CH_3CN: 194 ppm). ^{13}C-NMR spectra are calibrated with respect to the corresponding solvent residual peak ($CHCl_3$: 77.16 ppm; CH_3CN: 1.32 ppm). Multiplet signals are reported as follows: s = singlet, d = doublet, t = triplet, q = quartet, p = pentet, h = heptet, m = multiplet, or combinations thereof. ^{13}C signals are acquired with proton decoupling and are singlets unless otherwise stated. NMR yields were determined using mesitylene, 1-2-dimethoxyethane (1,2-DME), or dibromomethane as an internal standard.

Analytical thin-layer chromatography (TLC) was performed using silica gel 60 F254 coated aluminum sheets (Merck). Visualization was achieved by ultraviolet fluorescence (λ = 254 nm) and/or staining with potassium permanganate ($KMnO_4$).

Flash column chromatography was performed using silica gel 60 (pore size = 60 Å, mesh: 40-63 μm from Sigma-Aldrich or SiliCycle). Automated flash column chromatography was performed on a Biotage Isolera One system with Sfär columns.

High resolution mass spectrometry (HRMS): HRMS data were obtained by the mass spectrometry service in the Laboratorium für Organische Chemie at ETH Zürich on VG-TRIBRIB for electron impact ionization (EI), a Varian IonSpec Spectrometer for electrospray ionization (ESI) or an IonSpec Ultima Fourier Transform Mass Spectrometer for matrix-assisted laser desorption/ionization (MALDI) and are reported as (m/z).

X-Ray analysis: Single crystalline samples were measured on a Riguka Oxford Diffraction XtaLAB Synergy-S Dualflex kappa diffractometer equipped with a Dectris Pilatus 300 HPAD detector and using microfocus sealed tube Cu-Kα radiation with mirror optics (λ = 1.54178 Å). All measurements were carried out at 100 K (unless otherwise noted) using an Oxford Cryosystems Cryostream 800 sample cryostat. Data collected on the Rigaku instrument were integrated using CrysAlisPro and corrected for absorption effects using a combination of empirical (ABSPACK) and numerical corrections.[26] The structures were solved using SHELXT[27] or SHELXS[28] and refined by full-matrix least-squares analysis (SHELXL), [29] using the program package OLEX2. [30] Unless otherwise indicated below, all non-hydrogen atoms were refined anisotropically and hydrogen atoms were constrained to ideal geometries and refined with fixed isotropic displacement parameters (in terms of a riding model).

Photochemistry: The photocatalytic oxidative cleavage was performed in Evoluchem photoboxes cooled by air flow.

Reaction Optimization

Reaction Optimization with 3-Methyl Benzofuran or Methyl 3-Methylbenzofuran-5-Carboxylate as Starting Material

Optimization of the Oxidative Cleavage with 3-Methyl Benzofuran as Starting Material

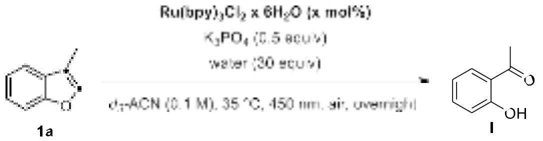

In a 4-mL screw-cap vial equipped with a magnetic stirring bar, benzofuran **1a** (13 mg, 12 µL, 0.10 mmol, 1.0 equiv), Ru(bpy)$_3$Cl$_2$ x 6H$_2$O (2.3 mg, 0.003 mmol, 3 mol%), and potassium phosphate tribasic (11 mg, 0.05 mmol, 0.5 equiv) were dissolved in acetonitrile-d_3 (1.0 mL). Then, water (54 µL, 3.0 mmol, 30 equiv) was added to the reaction mixture. The mixture was placed in the photoreactor (EvoluChem 450PF LED, 18W), and stirred at 35 °C for 16h in an open vial. Mesitylene (14 µL, 0.10 mmol) was added as an internal standard.

Entry	Deviation from above	NMR yield of 1a [%]	NMR conv. of I [%]
1	None	50	100
2	Vial lid closed	14	28
3	2h reaction time	51	87
4	0.5 mL solvent	24	40
5	6 mol% catalyst, 2h reaction time	63	97
6	9 mol% catalyst, 2h reaction time	75	100

Application of the optimized reaction conditions proved unsuccessful when applied to other starting materials. Thus, the reaction was further optimized with another model substrate.

Optimization of the Oxidative Cleavage with Methyl 3-Methylbenzofuran-5-Carboxylate as Starting Material

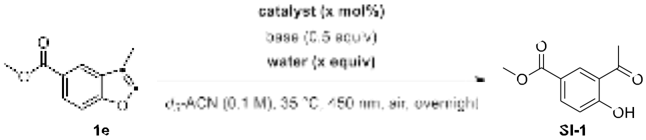

In a 4-mL screw-cap vial equipped with a magnetic stirring bar, benzofuran **1e** (20 mg, 0.10 mmol, 1.0 equiv), catalyst (0.003 mmol, 3 mol%), and K_2HPO_4 (11 mg, 0.05 mmol, 0.5 equiv) were dissolved in acetonitrile-d_3 (1.0 mL). Then, water (108 µL, 6 mmol, 60 equiv) was added to the reaction mixture. The mixture was placed in the photoreactor (EvoluChem 450PF LED, 18W), and stirred at 35 °C for 16h in an open vial. Mesitylene (14 µL, 0.10 mmol) was added as an internal standard.

Entry	Catalyst (x mol%)	Base (0.5 equiv)	Amount of water (equiv)	NMR yield of SI-1 [%]	NMR conv. of 1e [%]
	$Ru(bpy)_3Cl_2$ x $6H_2O$ (9 mol%)	K_3PO_4	30	25	100
	$Ru(bpy)_3Cl_2$ x $6H_2O$ (3 mol%)	K_3PO_4	30	31	100
	$Ru(bpy)_3(PF_6)_2$ (3 mol%)	K_3PO_4	30	56	100
	$Ru(bpy)_3(PF_6)_2$ (3 mol%)	K_3PO_4	60	60	100
	$Ru(bpy)_3(PF_6)_2$ (3 mol%)	K_2CO_3	60	40	100
	$Ru(bpy)_3(PF_6)_2$ (3 mol%)	Na_3PO_4	60	10	100
	$Ru(bpy)_3(PF_6)_2$ (3 mol%)	*t*BuOK	60	60	100
	$Ru(bpy)_3(PF_6)_2$ (3 mol%)	K_2HPO_4	60	70	100
	Rose Bengal (3 mol%)	K_2HPO_4	60	43	100
	Eosin Y (3 mol%)	K_2HPO_4	60	56	100
	$Ru(phen)_3Cl_2$ (3 mol%)	K_2HPO_4	60	81	100
	4CzIPN (3 mol%)	K_2HPO_4	60	0	100
	$Ir(ppy)_3$ (3 mol%)	K_2HPO_4	60	50	98

Cleavages Screening in the Absence of Water

The application of the optimized reaction conditions in combination with the ring closure using HOSA and MsOH to form benzoxazoles in a one-pot fashion did not yield the desired product. Therefore, the first cleavage step was further optimized in the absence of water and under O_2 atmosphere (balloon).

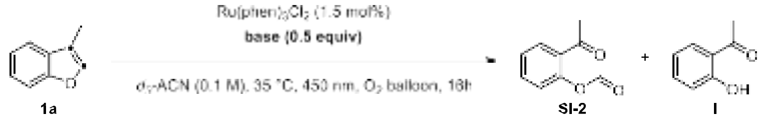

In a 4-mL screw-cap vial equipped with a magnetic stirring bar, 3-methylbenzofuran **1a** (13 mg, 0.10 mmol, 1.0 equiv), tris(1,10-phenanthroline)ruthenium dichloride (2.7 mg, 0.004 mmol, 1.5 mol%), and base (0.05 mmol, 0.5 equiv) were dissolved in acetonitrile-d_3 (1.0 mL). The vial was equipped with an oxygen balloon, placed in the photoreactor (EvoluChem 450PF LED, 18W), and stirred at 35 °C for 16h. Mesitylene (14 µL, 0.10 mmol) was added as an internal standard.

Entry	base (0.5 equiv)	NMR conv. of 1e [%]	NMR yield of SI-2 [%]	NMR yield of I [%]
1	K_2HPO_4	84	0	44
2	K_3PO_4	50	0	25
3	*t*BuOK	0	0	0
4	DIPEA	100	43	37

Optimization of the Benzoxazole Formation

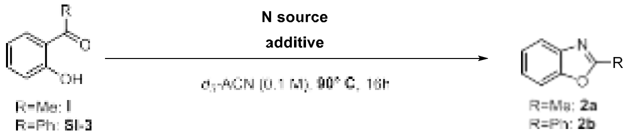

In a 4-mL screw-cap vial equipped with a magnetic stirring bar, 2-hydroxybenzoketone (**I** or **SI-3**, 0.10 mmol, 1.0 equiv), N source, and the corresponding additive were dissolved in acetonitrile-d_3 (1.0 mL). The vial was closed and stirred at 90 °C for 16h, unless otherwise stated. Mesitylene (14 µL, 0.10 mmol) was added as an internal standard.

Entry	R	N source	additive	comment	NMR conv. of I or SI-3 [%]	NMR yield of 2a or 2b [%]
1	Me	$NH_2OH\cdot HCl$ (3.0 equiv)	MsOH (3.0 equiv)	-	100	quant.
2	Me	HOSA (3.0 equiv)	MsOH (3.0 equiv)	-	100	quant.
3	Ph	$NH_2OH\cdot HCl$ (3.0 equiv)	MsOH (3.0 equiv)	-	52	36
4	Ph	$NH_2OH\cdot HCl$ (3.0 equiv)	MsOH (3.0 equiv)<br>$ZnCl_2$ (0.5 equiv)	-	40	27
5	Ph	$NH_2OH\cdot HCl$ (6.0 equiv)	MsOH (6.0 equiv)	-	43	31
6	Ph	HOSA (1.5 equiv)	MsOH (1.5 equiv)<br>$ZnCl_2$ (0.5 equiv)	-	60	43
7	Ph	HOSA (3.0 equiv)	MsOH (3.0 equiv)<br>$ZnCl_2$ (0.5 equiv)	-	100	78
8	Ph	HOSA (3.0 equiv)	MsOH (3.0 equiv)	-	100	72
9	Ph	HOSA (3.0 equiv)	MsOH (3.0 equiv)	16h at 60° C	14	12
10	Ph	HOSA (3.0 equiv)	MsOH (3.0 equiv)	3h at 90° C	100	75

Optimization of the Benzisoxazole Formation

Benzisoxazole formation starting from oxime

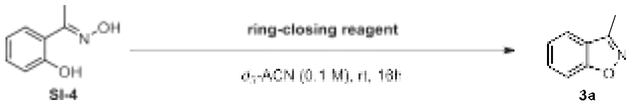

In a 4-mL screw-cap vial equipped with a magnetic stirring bar, 2-hydroxyacetophenoneoxime (**SI-4**, 0.10 mmol, 1.0 equiv) was dissolved in acetonitrile-d_3 (1.0 mL) and the corresponding reagent was added. The vial was closed and stirred at room temperature for 16h, unless otherwise stated. Mesitylene (14 µL, 0.10 mmol) was added as an internal standard.

Entry	reagent	comment	NMR conv. of SI-4 [%]	NMR yield of 3a [%]
1	PPh_3 (1.0 equiv), DIAD (1.0 equiv)	-	87	46
2	PPh_3 (1.0 equiv), DIAD (2.0 equiv)	-	83	44
3	PPh_3 (2.0 equiv), DIAD (2.0 equiv)	-	100	41
4	MsCl (1.2 equiv), DIPEA (2.5 equiv)	at 60 °C	100	64

5	MsCl (1.2 equiv), NEt_3 (2.5 equiv	at 60 °C	100	70
6	MsCl (1.2 equiv), $NaHCO_3$ (2.5 equiv)	at 60 °C	40	0
7	MsCl (1.2 equiv), K_3PO_4 (2.5 equiv)	at 60 °C	74	35

Benzisoxazole formation *via in situ* oxime formation and ring closing

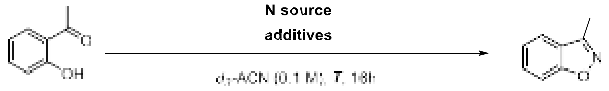

I   3a

In a 4-mL screw-cap vial equipped with a magnetic stirring bar, 2-hydroxyacetophenone (**I**, 0.10 mmol, 1.0 equiv) was dissolved in acetonitrile-d_3 (1.0 mL) and the corresponding N source and additives were added. The vial was closed and stirred at the corresponding temperature for 16h, unless otherwise stated. Mesitylene (14 µL, 0.10 mmol) was added as an internal standard.

Entry	reagents	*T* [°C]	NMR conv. of I [%]	NMR yield of 3a [%]
1	1) $NH_2OH{\cdot}HCl$ (1.5 equiv), 2h<br>2) MsCl (1.2 equiv), NEt_3 (2.5 equiv), 1h	80	10	0
2	1) HOSA (1.5 equiv), 2h<br>2) MsCl (1.2 equiv), NEt_3 (2.5 equiv), 1h	80	62	0
3	HOSA (1.5 equiv), DABCO (2.0 equiv)	80	0	0
4	HOSA (1.5 equiv), K_2CO_3 (2.0 equiv)	80	0	0
5	HOSA (1.5 equiv), *t*BuOK (2.0 equiv)	80	0	0
6	1) $NH_2OH{\cdot}HCl$ (3.0 equiv), NaOAc (2.5 equiv), 5h<br>2) MsCl (1.2 equiv), NEt_3 (2.5 equiv), 1h	90	40	0
7	1) $NH_2OH{\cdot}HCl$ (5.0 equiv), NaOAc (2.5 equiv), 5h<br>2) MsCl (1.2 equiv), NEt_3 (2.5 equiv), 1h	90	47	0
8	HOSA (3.0 equiv), NEt_3 (5.0 equiv), MsCl (2.0 equiv)	rt	67	0
9	HOSA (3.0 equiv), NEt_3 (5.0 equiv), MsCl (2.0 equiv)	50	79	0
10	HOSA (3.0 equiv), NEt_3 (5.0 equiv), MsCl (2.0 equiv)	90	73	0

Oxime formation and subsequent cyclization in acetonitrile was not successful. Thus, a different approach, involving imine formation followed by oxidation via NCS and cyclization, was explored.[59]

Benzisoxazole formation with solvent swap

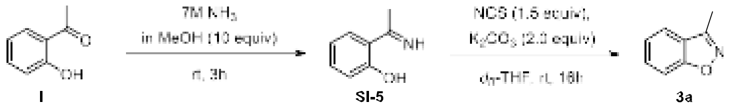

In a 4-mL screw-cap vial equipped with a magnetic stirring bar, 2-hydroxyacetophenone (**I**, 0.10 mmol, 1.0 equiv) was dissolved in 7M NH_3 in methanol (140 µL, 1.0 mmol, 10 equiv). The vial was closed and stirred at room temperature for 3h. Then, the crude mixture was concentrated under reduced pressure and 1mL THF-d_8 was added, followed by NCS (20 mg, 0.15 mmol, 1.5 equiv) and K_2CO_3 (28 mg, 0.20 mmol, 2.0 equiv). The reaction mixture was stirred at room temperature for 16h. Mesitylene (14 µL, 0.10 mmol) was added as an internal standard. The desired 3-methylbenzo[*d*]isoxazole **3a** was obtained in 80% NMR yield.

Reaction Optimization with Benzofuran as Starting Material

Optimization of the Oxidative Cleavage with Benzofuran as Starting Material

Base screening

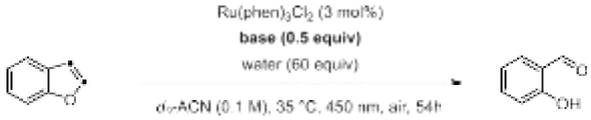

In a 4-mL screw-cap vial equipped with a magnetic stirring bar, benzofuran **4a** (12 mg, 11.0 µL, 0.10 mmol), $Ru(phen)_3Cl_2$ (1.1 mg, 0.003 mmol, 3 mol%) and base (0.05 mmol, 0.5 equiv) were dissolved in acetonitrile-d_3 (1.0 mL). Then, water (108 µL, 6 mmol, 60 equiv) was added to the reaction mixture. The mixture was placed in the photoreactor (EvoluChem 450PF LED, 18W), and stirred at 35 °C for 54h in an open vial. 1,2-DME (10.5 µL, 0.10 mmol) was added as an internal standard.

Entry	Catalyst (0.03 equiv)	NMR yield of SI-6 [%]	NMR conv. of 4a [%]
1	K_2HPO_4	39	100
2	KOH	51	100
3	NaOH	53	100

Concentration screening

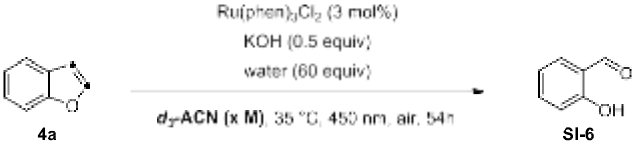

In a 4-mL screw-cap vial equipped with a magnetic stirring bar, benzofuran **4a** (12 mg, 11.0 µL, 0.10 mmol), $Ru(phen)_3Cl_2$ (1.1 mg, 0.003 mmol, 3 mol%) and KOH (2.8 mg, 0.05 mmol, 0.5 equiv) were dissolved in acetonitrile-d_3. Then, water (108 µL, 6 mmol, 60 equiv) was added to the reaction mixture. The mixture was placed in the photoreactor (EvoluChem 450PF LED, 18W), and stirred at 35 °C for 54h in an open vial. 1,2-DME (10.5 µL, 0.10 mmol) was added as an internal standard.

Entry	Concentration (M)	NMR yield of SI-6 [%]	NMR conv. of 4a [%]
1	0.1	51	100
2	0.05	25	40

3	0.2	30	100

Catalyst screening

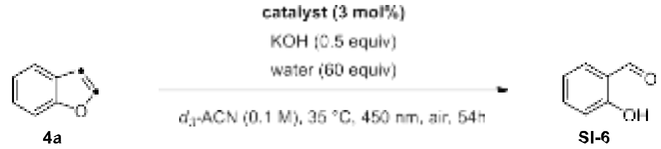

In a 4-mL screw-cap vial equipped with a magnetic stirring bar, benzofuran **4a** (12 mg, 11.0 µL, 0.10 mmol), catalyst (0.003 mmol, 3 mol%) and KOH (2.8 mg, 0.05 mmol, 0.5 equiv) were dissolved in acetonitrile-d_3 (1.0 mL). Then, water (108 µL, 6 mmol, 60 equiv) was added to the reaction mixture. The mixture was placed in the photoreactor (EvoluChem 450PF LED, 18W), and stirred at 35 °C for 54h in an open vial. 1,2-DME (10.5 µL, 0.10 mmol) was added as an internal standard.

Entry	Catalyst (0.03 equiv)	NMR yield of SI-6 [%]	NMR conv. of 4a [%]
1	$Ru(phen)_3Cl_2$	51	100
2	$Ru(bpy)_3(PF_6)_2$	26	100
3	$Ru(bpy)_3Cl_2$	11	100

Water amount screening

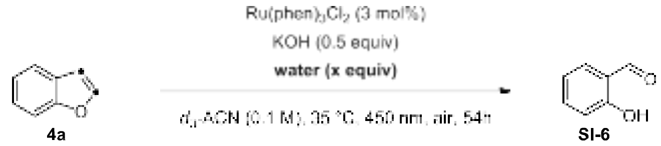

In a 4-mL screw-cap vial equipped with a magnetic stirring bar, benzofuran **4a** (12 mg, 11.0 µL, 0.10 mmol), $Ru(phen)_3Cl_2$ (1.1 mg, 0.003 mmol, 3 mol%) and KOH (2.8 mg, 0.05 mmol, 0.5 equiv) were dissolved in acetonitrile-d_3 (1.0 mL). Then, water (equiv) was added to the reaction mixture. The mixture was placed in the photoreactor (EvoluChem 450PF LED, 18W), and stirred at 35 °C for 54h in an open vial. 1,2-DME (10.5 µL, 0.10 mmol) was added as an internal standard.

Entry	Water amount	NMR yield of SI-6 [%]	NMR conv. of 4a [%]
1	60 equiv	51	100
2	120 equiv	51	100

Catalyst amount screening

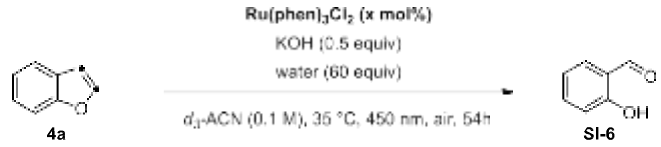

In a 4-mL screw-cap vial equipped with a magnetic stirring bar, benzofuran **4a** (12 mg, 11.0 µL, 0.10 mmol), $Ru(phen)_3Cl_2$ (x mol%) and KOH (2.8 mg, 0.05 mmol, 0.5 equiv) were dissolved in acetonitrile-d_3 (1.0 mL). Then, water (108 µL, 6 mmol, 60 equiv)

was added to the reaction mixture. The mixture was placed in the photoreactor (EvoluChem 450PF LED, 18W), and stirred at 35 °C for 54h in an open vial. 1,2-DME (10.5 µL, 0.10 mmol) was added as an internal standard.

Entry	Catalyst amount	NMR yield of SI-6 [%]	NMR conv. of 4a [%]
1	0.03 equiv	51	100
2	0.015 equiv	53	100

Base amount screening

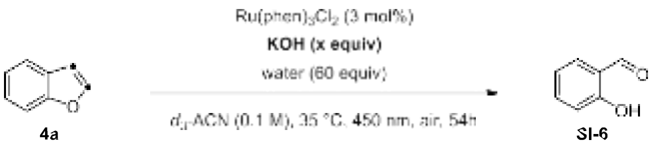

In a 4-mL screw-cap vial equipped with a magnetic stirring bar, benzofuran **4a** (12 mg, 11.0 µL, 0.10 mmol), $Ru(phen)_3Cl_2$ (1.1 mg, 0.003 mmol, 3 mol%) and KOH (x equiv) were dissolved in acetonitrile-d_3 (1.0 mL). Then, water (108 µL, 6 mmol, 60 equiv) was added to the reaction mixture. The mixture was placed in the photoreactor (EvoluChem 450PF LED, 18W), and stirred at 35 °C for 54h in an open vial. 1,2-DME (10.5 µL, 0.10 mmol) was added as an internal standard.

Entry	Base amount	NMR yield of SI-6 [%]	NMR conv. of 4a [%]
1	0.5 equiv	51	100
2	0.2 equiv	59	100
3	none	33	100

Further Optimization

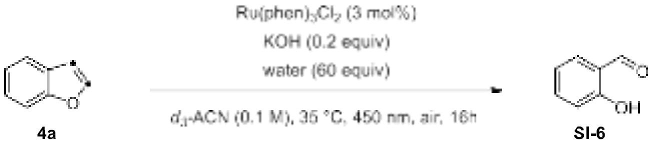

In a 4-mL screw-cap vial equipped with a magnetic stirring bar, benzofuran **4a** (12 mg, 11.0 µL, 0.10 mmol), $Ru(phen)_3Cl_2$ (1.1 mg, 0.003 mmol, 3 mol%) and KOH (1.2 mg, 0.02 mmol, 0.2 equiv) were dissolved in acetonitrile-d_3 (1.0 mL). Then, water (108 µL, 6 mmol, 60 equiv) was added to the reaction mixture. The mixture was placed in the photoreactor (EvoluChem 450PF LED, 18W), and stirred at 35 °C for 16h in an open vial. 1,2-DME (10.5 µL, 0.10 mmol) was added as an internal standard.

Entry	Deviation from above	NMR yield of SI-6 [%]	NMR conv. of 4a [%]
1	none	59	100
2	O_2 balloon	64	100
3	0.2 M, O_2 balloon	61	100
4	DIPEA instead of KOH, O_2 balloon	78	100

Optimization of Benzisoxazole formation

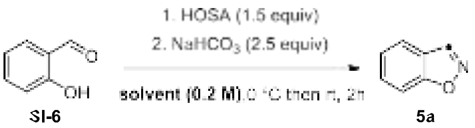

In a 4-mL screw-cap vial equipped with a magnetic stirring bar, 2-hydroxybenzaldehyde (12 mg, 11.0 µL, 0.10 mmol) was dissolved in the solvent mixture (0.5 mL). HOSA (17 mg, 0.15 mmol, 1.5 equiv) was added at 0 °C. The mixture was stirred for 1h before $NaHCO_3$ (21 mg, 0.25 mmol, 2.5 equiv) was added and the mixture was stirred at room temperature for 1h. 1,2-DME (10.5 µL, 0.10 mmol) was added as an internal standard.

**Entry**	**Solvent**	**NMR yield of 5a [%]**	**NMR conv. of SI-6 [%]**
1	d_3-ACN:H_2O 9:1	5	100
2	d_3-ACN:H_2O 5:1	71	100
3	d_3-ACN:H_2O 2:1	75	100
4	d_3-ACN:H_2O 1:1	93	100
5	d_3-ACN:H_2O 1:2	quant.	100
6	d_3-ACN:H_2O 1:5	81	100
7	d_3-ACN:H_2O 1:9	78	100

Preparation of Starting Materials

General Procedure A for the Synthesis of Alkylated 2-Iodophenol

In a 250 mL round bottom flask, 2-iodophenol derivate (5.0 mmol, 1 equiv) was dissolved in DMF (50 mL). Sodium hydride (220 mg, 60% wt, 5.5 mmol, 1.1 equiv) was then slowly added to the reaction mixture that was cooled down to 0°C. Once the reaction stopped producing H_2, allyl bromide derivative was added (6.0 mmol, 1.2 equiv) and the reaction mixture was brought to room temperature. The resulting mixture was quenched with H_2O after 2 h and the organic phase was extracted with ethyl acetate. Combined organic layers were dried, filtered, and concentrated *in vacuo*. The crude was purified by flash chromatography on silica gel with ethyl acetate in cyclohexane.

General Procedure B for the Synthesis of Benzofurans

In a Schlenk tube under nitrogen atmosphere, 6- or 5-bromobenzofuran (99 mg, 0.50 mmol, 1 equiv), phenylboronic acid (1.1 equiv, 0.55 mmol), and potassium phosphate tribasic (318 mg, 1.50 mmol, 3 equiv) were dissolved in 1,4-dioxane (2 mL). Then, water (45 mg, 45 µL, 2.5 mmol, 5 equiv) was added. The mixture was stirred at 80 °C for 4h. The flask was then allowed to cool to room temperature. The crude mixture was diluted with ethyl acetate (10 mL) and filtered through a plug of celite, eluting with ethyl acetate. The resulting solution was washed with water (3 x 10 mL) followed by brine (10 mL) and the organic phases were collected. The organic phase was dried over anhydrous Na_2SO_4, filtered, and concentrated *in vacuo*. The crude residue was purified by column chromatography on silica gel with ethyl acetate in cyclohexane.

General Procedure C for the Synthesis of Benzofurans

To a 10 mL Schlenk flask under nitrogen atmosphere, tetrakis(triphenylphosphine)palladium(0) (11 mg, 9.5 µmol, 0.019 equiv), potassium carbonate (59 µL, 138 mg, 1.0 mmol, 2 equiv), and phenylboronic acid (61.0 mg, 0.5 µmol, 1.0 equiv) were added. A deoxygenated solution of 6-bromo-3-methylbenzofuran or 5-bromo-3-methylbenzofuran (69 µL, 106 mg, 0.5 mmol, 1.0 equiv) in toluene (0.84 mL), 1,4-dioxane (0.84 mL), and water (0.42 mL) was added. The reaction mixture was refluxed for 12 hours. After completion of the reaction, the aqueous layer was removed, and the organic layer was concentrated under reduced pressure. The crude was purified *via* flash chromatography on silica gel with ethyl acetate in cyclohexane. For the reactions starting from 1.0 mmol of alkylated 2-iodophenol, the reactions were set up in a 20 mL Schlenk flask.

Synthesis of Alkylated 2-Iodophenol

1-Iodo-2-((3-methylbut-2-en-1-yl)oxy)benzene (***SI-7***)

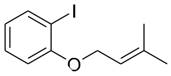

1-Iodo-2-((3-methylbut-2-en-1-yl)oxy)benzene (**SI-7**) was prepared according to the general procedure A using 2-iodophenol (1.10 g, 5.0 mmol) and 1-bromo-3-methylbut-2-ene (894 mg, 6.0 mmol). Flash column chromatography on silica gel (cyclohexane) of the crude afforded the product **SI-7** as a yellow oil (1.38 g, 96%).

^{1}H NMR (400 MHz, $CDCl_3$) δ 7.77 (dd, *J* = 7.8, 1.6 Hz, 1H), 7.28 (ddd, *J* = 8.2, 7.4, 1.6 Hz, 1H), 6.82 (dd, *J* = 8.3, 1.4 Hz, 1H), 6.70 (ddd, *J* = 7.8, 7.3, 1.4 Hz, 1H), 5.51 (dddd, *J* = 6.5, 5.1, 2.8, 1.4 Hz, 1H), 4.58 (dp, *J* = 6.5, 0.9 Hz, 2H), 1.79 (q, *J* = 1.3 Hz, 3H), 1.75 (d, *J* = 1.3 Hz, 3H).

^{13}C NMR (101 MHz, $CDCl_3$) δ 157.6, 139.6, 138.1, 129.5, 122.6, 119.7, 112.9, 87.1, 66.4, 25.9, 18.5.

HRMS (ESI) m/z: $[M+Na]^+$ Calculated for $C_{11}H_{13}INaO^+$ 310.9903; Found 310.9902.

The spectral data are consistent with those reported in the literature.[31]

*1-(Cinnamyloxy)-2-iodobenzene (**SI-8**)*

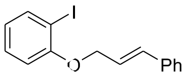

1-(Cinnamyloxy)-2-iodobenzene (**SI-8**) was prepared according to the general procedure A using 2-iodophenol (1.10 g, 5.0 mmol) and (*E*)-(3-bromoprop-1-en-1-yl)benzene (1.18 g, 6.0 mmol). Flash column chromatography on silica gel (cyclohexane) of the crude afforded the product **SI-8** as a yellow oil (1.68 g, 89%).

^{1}H NMR (400 MHz, $CDCl_3$) δ 7.80 (dd, *J* = 7.8, 1.6 Hz, 1H), 7.45 – 7.41 (m, 2H), 7.36 – 7.27 (m, 4H), 6.88 (dd, *J* = 8.2, 1.4 Hz, 1H), 6.82 (dt, *J* = 16.0, 1.7 Hz, 1H), 6.73 (ddd, *J* = 7.8, 7.4, 1.4 Hz, 1H), 6.42 (dt, *J* = 16.0, 5.4 Hz, 1H), 4.77 (dd, *J* = 5.4, 1.6 Hz, 2H).

^{13}C NMR (101 MHz, $CDCl_3$) δ 157.4, 139.7, 136.6, 133.1, 129.6, 128.8 (2C), 128.1, 126.8 (2C), 124.1, 122.9, 112.9, 87.0, 69.9.

HRMS (ESI) m/z: $[M+Na]^+$ Calculated for $C_{15}H_{13}INaO^+$ 358.9903; Found 358.9898.

The spectral data are consistent with those reported in the literature. [32]

*Methyl 4-(allyloxy)-3-iodobenzoate (**SI-9**)*

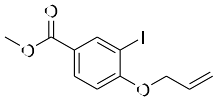

Methyl 4-(allyloxy)-3-iodobenzoate (**SI-9**) was prepared according to the general procedure A using methyl 4-hydroxy-3-iodobenzoate (1.39 g, 5.0 mmol) and 3-bromoprop-1-ene (726 mg, 519 µL, 6.0 mmol). Flash column chromatography on silica gel (ethyl acetate in cyclohexane) of the crude afforded the product **SI-9** as a white solid (1.19 g, 75%).

^{1}H NMR (400 MHz, $CDCl_3$) δ 8.47 (d, *J* = 2.1 Hz, 1H), 7.99 (dd, *J* = 8.6, 2.1 Hz, 1H), 6.80 (d, *J* = 8.7 Hz, 1H), 6.05 (ddt, *J* = 17.2, 10.6, 4.8 Hz, 1H), 5.53 (dtd, *J* = 17.3, 1.8, 1.3 Hz, 1H), 5.35 (dq, *J* = 10.6, 1.5 Hz, 1H), 4.66 (dt, *J* = 4.9, 1.7 Hz, 2H), 3.89 (s, 3H).

^{13}C NMR (101 MHz, $CDCl_3$) δ 165.7, 160.8, 141.2, 131.9, 131.6, 124.5, 118.3, 111.3, 86.0, 69.9, 52.3.

HRMS (ESI) m/z: $[M+H]^+$ Calculated for $C_{11}H_{12}IO_3^+$ 318.9826; Found 318.9824.

The spectral data are consistent with those reported in the literature. [33]

*2-(Allyloxy)-1-iodo-4-methoxybenzene (**SI-10**)*

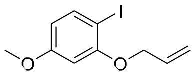

2-(Allyloxy)-1-iodo-4-methoxybenzene (**SI-10**) was prepared according to the general procedure A using 2-iodo-5-methoxyphenol (1.0 g, 4.0 mmol) and 3-bromoprop-1-ene (581 mg, 415 µL, 4.8 mmol). Flash column chromatography on silica gel (cyclohexane) of the crude afforded the product **SI-10** as a colourless oil (1.19 g, 75%).

^{1}H NMR (400 MHz, $CDCl_3$) δ 7.63 (d, J = 8.6 Hz, 1H), 6.42 (d, J = 2.7 Hz, 1H), 6.33 (dd, J = 8.6, 2.7 Hz, 1H), 6.05 (ddt, J = 17.3, 10.6, 4.9 Hz, 1H), 5.52 (dq, J = 17.3, 1.7 Hz, 1H), 5.31 (dq, J = 10.6, 1.5 Hz, 1H), 4.57 (dt, J = 4.8, 1.7 Hz, 2H), 3.78 (s, 3H).

^{13}C NMR (101 MHz, $CDCl_3$) δ 161.4, 158.0, 139.3, 132.6, 117.8, 107.5, 100.7, 75.6, 69.8, 55.7.

HRMS (ESI) m/z: $[M+Na]^+$ Calculated for $C_{10}H_{11}IO_2Na^+$ 312.9696; Found 312.9695.

The spectral data are consistent with those reported in the literature. [34]

*Methyl 3-(allyloxy)-4-iodobenzoate (**SI-11**)*

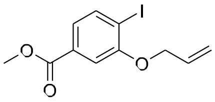

Methyl 3-(allyloxy)-4-iodobenzoate (**SI-11**) was prepared according to the general procedure A using methyl 3-hydroxy-4-iodobenzoate (1.39 g, 5.0 mmol) and 3-bromoprop-1-ene (726 mg, 519 µL, 6.0 mmol). Flash column chromatography on silica gel (cyclohexane) of the crude afforded the product **SI-11** as a white solid (1.26 g, 79%).

^{1}H NMR (400 MHz, $CDCl_3$) δ 7.86 (d, *J* = 8.2 Hz, 1H), 7.43 (d, J = 1.8 Hz, 1H), 7.37 (dd, J = 8.1, 1.8 Hz, 1H), 6.07 (ddt, J = 17.3, 10.6, 4.9 Hz, 1H), 5.60 – 5.50 (m, 1H), 5.34 (dq, J = 10.6, 1.5 Hz, 1H), 4.66 (dt, J = 4.7, 1.6 Hz, 2H), 3.91 (s, 3H).

^{13}C NMR (101 MHz, $CDCl_3$) δ 166.7, 157.4, 139.7, 132.2, 131.7, 123.6, 118.1, 112.7, 93.4, 70.0, 52.5.

HRMS (ESI) m/z: $[M+Na]^+$ Calculated for $C_{11}H_{11}INaO_3^+$ 340.9645; Found 340.9649.

The spectral data are consistent with those reported in the literature. [35]

Synthesis of Benzofurans

*5-Phenylbenzofuran (**4b**)*

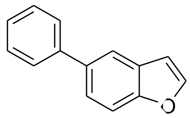

In a 5 mL Schlenk tube 5-bromobenzofuran (99 mg, 63 µL, 0.50 mmol, 1.0 equiv), phenylboronic acid (67 mg, 0.55 mmol, 1.1 equiv), 1,1'-bis(diphenylphosphino)ferrocene-palladium(II) dichloride (15 mg, 0.02 mmol, 0.04 equiv), and potassium phosphate tribasic (318 mg, 1.50 mmol, 3.0 equiv) were dissolved in 1,4-dioxane (2 mL). Then, water (45 mg, 45 µL, 2.50 mmol, 5.0 equiv) was added. The mixture was stirred at 80 °C for 4 h. The mixture was cooled to room temperature, diluted with ethyl acetate (10 mL) and filtered through a plug of celite. The resulting solution was washed with water (3 x 10 mL), followed by brine (10 mL), and the organic phases were collected. The organic phase was dried over Na_2SO_4, filtered, and concentrated under reduced pressure. The crude residue was purified *via* column chromatography on silica gel (ethyl acetate in cyclohexane) to afford the desired product **4b** as a white solid (64 mg, 66%).

^{1}H NMR (400 MHz, $CDCl_3$) δ 7.81 (dd, *J* = 1.8, 0.8 Hz, 1H), 7.68 – 7.62 (m, 3H), 7.61 – 7.52 (m, 2H), 7.50 – 7.43 (m, 2H), 7.39 – 7.33 (m, 1H), 6.83 (dd, *J* = 2.2, 0.9 Hz, 1H).

^{13}C NMR (101 MHz, $CDCl_3$) δ 154.7, 145.7, 141.8, 136.6, 128.9 (2C), 128.1, 127.6 (2C), 127.0, 124.1, 119.8, 111.6, 106.9.

HRMS (EI) m/z: $[M]^+$ Calculated for $C_{14}H_{10}O^+$ 194.0726; Found 194.0722.

The spectral data are consistent with those reported in the literature. [36]

*Methyl benzofuran-5-carboxylate (**4c**)*

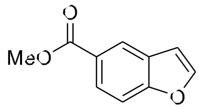

In a round bottom flask benzofuran-5-carboxylic acid (324 mg, 2.0 mmol, 1 equiv) was dissolved in methanol (6.7 mL). Then, one drop of concentrated sulfuric acid was added and the solution stirred 48 h at reflux. After cooling to room temperature, the solvent was removed under reduced pressure, the residue dissolved in DCM and poured in a saturated potassium carbonate solution. The mixture was extracted with DCM, and the organic phase was washed with brine, dried over Na_2SO_4, and concentrated under reduced pressure to obtain the product **4c** as a white solid (334 mg, 95%).

^{1}H NMR (500 MHz, $CDCl_3$) δ 8.35 (dd, *J* = 1.8, 0.6 Hz, 1H), 8.03 (ddd, *J* = 8.7, 1.8, 0.4 Hz, 1H), 7.69 (d, *J* = 2.2 Hz, 1H), 7.53 (dt, *J* = 8.6, 0.9 Hz, 1H), 6.84 (dd, *J* = 2.2, 1.0 Hz, 1H), 3.94 (s, 3H).

^{13}C NMR (126 MHz, $CDCl_3$) δ 167.4, 157.6, 146.4, 127.6, 126.2, 125.3, 123.9, 111.4, 107.3, 52.3.

HRMS (EI) m/z: $[M]^+$ Calculated for $C_{10}H_8O_3^+$ 176.0468; Found 176.0466.

The spectral data are consistent with those reported in the literature. [37]

tert-*Butyl benzofuran-5-ylcarbamate (**4d**)*

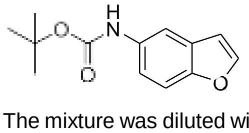

In a 25 round bottom flask benzofuran-5-amine (200 mg, 163 µL, 1.50 mmol, 1 equiv) and di-*tert*-butyl dicarbonate (386 mg, 0.41 mL, 1.77 mmol, 1.18 equiv) were dissolved in THF (4 mL). DIPEA (382 mg, 515 µL, 2.96 mmol, 1.97 equiv) was added dropwise and the solution stirred overnight at rt. The mixture was diluted with water and extracted with DCM (3 x 10 mL). The combined organic phases were dried over anhydrous Na_2SO_4, filtered, and evaporated under reduced pressure. The residue was then purified by column chromatography on silica gel (ethyl acetate in cyclohexane) to obtain the desired product **4d** (298 mg, 85%) as a yellow solid.

^{1}H NMR (500 MHz, $CDCl_3$) δ 7.74 (s, 1H), 7.59 (d, *J* = 2.1 Hz, 1H), 7.42 – 7.36 (m, 1H), 7.13 (dd, *J* = 8.8, 2.3 Hz, 1H), 6.70 (dd, *J* = 2.2, 1.0 Hz, 1H), 6.51 (s, 1H), 1.53 (s, 9H).

^{13}C NMR (126 MHz, $CDCl_3$) δ 153.4, 151.6, 145.9, 133.7, 128.0, 116.7, 111.5, 106.9, 80.5, 28.5 (3C). *(one carbon is obstructed)*

HRMS (ESI) m/z: $[M+Na]^+$ Calculated for $C_{13}H_{15}NNaO_3^+$ 256.0950; Found 256.0944.

The identity of **4d** was unambiguously confirmed by single crystal X-ray analysis.

*6-Phenylbenzofuran (**4e**)*

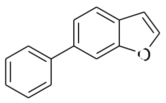

6-Phenylbenzofuran (**4e**) was prepared according to the general procedure B using phenylboronic acid (100 mg, 0.83 mmol). Flash column chromatography on silica gel (cyclohexane) afforded the product **4e** as a yellow oil (44 mg, 30%).

^{1}H NMR (400 MHz, $CDCl_3$) δ 7.78 (dt, *J* = 1.6, 0.8 Hz, 1H), 7.70 – 7.66 (m, 4H), 7.54 (dd, *J* = 8.1, 1.5 Hz, 1H), 7.52 – 7.46 (m, 2H), 7.42 – 7.36 (m, 1H), 6.83 (dd, *J* = 2.2, 1.0 Hz, 1H).

^{13}C NMR (101 MHz, $CDCl_3$) δ 155.7, 145.6, 141.5, 138.2, 128.9 (2C), 127.5 (2C), 127.3, 126.7, 122.5, 121.4, 110.1, 106.6.

HRMS (EI) m/z: $[M]^+$ Calculated for $C_{14}H_{10}O^+$ 194.0726; Found 194.0721.

The spectral data are consistent with those reported in the literature. [38]

*6-(4-(Methylsulfonyl)phenyl)benzofuran (**4h**)*

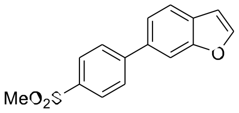

6-(4-(Methylsulfonyl)phenyl)benzofuran (**4h**) was prepared according to the general procedure B using (4-(methylsulfonyl)phenyl)boronic acid (270 mg, 1.35 mmol). Flash column chromatography on silica gel (cyclohexane) afforded the product **4h** as a yellow oil (179 mg, 53%).

^{1}H NMR (500 MHz, $CDCl_3$) δ 8.04 – 8.01 (m, 2H), 7.84 – 7.81 (m, 2H), 7.76 (dt, *J* = 1.6, 0.8 Hz, 1H), 7.71 – 7.69 (m, 2H), 7.51 (dd, *J* = 8.1, 1.6 Hz, 1H), 6.83 (dd, *J* = 2.2, 1.0 Hz, 1H), 3.11 (s, 3H).

^{13}C NMR (126 MHz, $CDCl_3$) δ 155.6, 147.0, 146.4, 139.1, 135.8, 129.3, 128.3 (2C), 128.1 (2C), 122.6, 121.9, 110.5, 106.7, 44.8.

HRMS (EI) m/z: $[M]^+$ Calculated for $C_{15}H_{12}O_3S^+$ 272.0502; Found 272.0498.

*5-(3-Chlorophenyl)benzofuran (**4i**)*

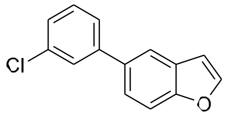

5-(3-Chlorophenyl)benzofuran (**4i**) was prepared according to the general procedure B using (3-chlorophenyl)boronic acid (130 mg, 0.83 mmol). Flash column chromatography on silica gel (cyclohexane) afforded the product **4i** as a transparent oil (36 mg, 21%).

^{1}H NMR (400 MHz, $CDCl_3$) δ 7.78 (dd, *J* = 2.0, 0.7 Hz, 1H), 7.67 (d, *J* = 2.2 Hz, 1H), 7.61 (td, *J* = 1.9, 0.5 Hz, 1H), 7.57 (dt, *J* = 8.6, 0.8 Hz, 1H), 7.52 – 7.46 (m, 2H), 7.38 (td, *J* = 7.8, 0.5 Hz, 1H), 7.32 (ddd, *J* = 8.0, 2.0, 1.3 Hz, 1H), 6.83 (dd, *J* = 2.2, 0.9 Hz, 1H).

^{13}C NMR (101 MHz, $CDCl_3$) δ 154.9, 145.9, 143.6, 135.2, 134.7, 130.1, 128.2, 127.7, 127.0, 125.7, 124.0, 119.9, 111.8, 106.9.

HRMS (EI) m/z: $[M]^+$ Calculated for $C_{14}H_9ClO^+$ 228.0336; Found 228.0332.

*5-(2-(Trifluoromethyl)phenyl)benzofuran (**4j**)*

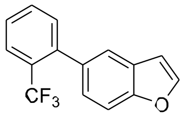

5-(2-(Trifluoromethyl)phenyl)benzofuran (**4j**) was prepared according to the general procedure B using (2-(trifluoromethyl)phenyl)boronic acid (160 mg, 0.83 mmol). Flash column chromatography on silica gel (cyclohexane) afforded the product **4j** as a transparent oil (36 mg, 18%).

^{1}H NMR (400 MHz, $CDCl_3$) δ 7.77 (ddt, *J* = 7.8, 1.1, 0.6 Hz, 1H), 7.68 (d, *J* = 2.2 Hz, 1H), 7.59 – 7.52 (m, 3H), 7.48 (dddd, *J* = 8.7, 6.6, 1.5, 0.8 Hz, 1H), 7.40 – 7.36 (m, 1H), 7.29 – 7.25 (m, 1H), 6.80 (dd, *J* = 2.2, 1.0 Hz, 1H).

^{13}C NMR (101 MHz, $CDCl_3$) δ 154.6, 145.7, 141.9 – 141.7 (m), 134.8, 132.7, 131.3 (q, *J* = 1.2 Hz), 129.3 – 128.4 (m), 127.4, 127.1, 126.2 (q, *J* = 5.4 Hz), 125.7 (q, *J* = 1.6 Hz), 128.6 – 120.0 (m), 121.8 (q, *J* = 1.6 Hz), 110.7, 106.8.

^{19}F NMR (376 MHz, $CDCl_3$) δ -56.78.

HRMS (ESI) m/z: $[M]^+$ Calculated for $C_{15}H_9F_3O^+$ 262.0605; Found 262.0596.

N-*Phenethylbenzofuran-5-carboxamide* ***(4k)***

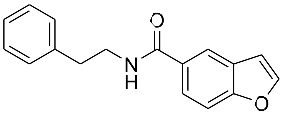

In a 50 mL round bottom flask, benzofuran-5-carboxylic acid (162 mg, 1.0 mmol, 1 equiv), phenethylamine (133 mg, 138 µL, 1.10 mmol, 1.1 equiv), and triethylamine (506 mg, 697 µL, 5.0 mmol, 5 equiv), were dissolved in DMF (17 mL). Propylphosphonic anhydride (1.27 g, 1.19 mL, 50% Wt, 2.0 mmol, 2 equiv) was added carefully. The solution was stirred at room temperature for 24 h. The reaction was then quenched with water (5 mL) and stirred for 5 min. Then, 2M HCl (10 mL) was added and the reaction was extracted with DCM (3 x 20 mL). The organic phases were collected and washed with 2M HCl, 1M NaOH, and brine, dried over anhydrous $MgSO_4$, filtered and the solvent was removed under reduced pressure. The residue was then purified *via* flash column chromatography on silica gel (ethyl acetate in cyclohexane) to afford product **4k** (152 mg, 57%) as a white solid.

^{1}H NMR (400 MHz, $CDCl_3$) δ 7.98 (dd, *J* = 1.9, 0.7 Hz, 1H), 7.67 (d, *J* = 2.2 Hz, 1H), 7.64 (dd, *J* = 8.6, 1.9 Hz, 1H), 7.50 (dt, *J* = 8.6, 0.8 Hz, 1H), 7.37 – 7.31 (m, 2H), 7.28 – 7.23 (m, 3H), 6.81 (dd, *J* = 2.2, 0.9 Hz, 1H), 3.75 (td, *J* = 6.9, 5.9 Hz, 2H), 2.96 (t, *J* = 6.9 Hz, 2H).

^{13}C NMR (126 MHz, $CDCl_3$) δ 167.8, 156.7, 146.4, 139.1, 130.0, 129.0 (2C), 128.9 (2C), 127.7, 126.8, 123.4, 120.7, 111.5, 107.1, 41.4, 35.9.

HRMS (ESI) m/z: $[M+H]^+$ Calculated for $C_{17}H_{16}NO_2^+$ 266.1176; Found 266.1169.

The spectral data are consistent with those reported in the literature. [39]

N-*Phenethylbenzofuran-6-carboxamide* ***(4l)***

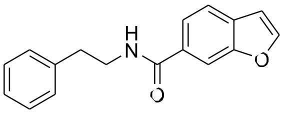

In a 50 mL round bottom flask, benzofuran-6-carboxylic acid (162 mg, 1.0 mmol, 1 equiv), phenethylamine (133 mg, 138 µL, 1.10 mmol, 1.1 equiv), and triethylamine (506 mg, 697 µL, 5.0 mmol, 5 equiv), were dissolved in DMF (17 mL). Propylphosphonic anhydride (1.27 g, 1.19 mL, 50% Wt, 2.0 mmol, 2 equivl) was added carefully. The solution was stirred at room temperature for 24 h. The reaction was then quenched with water (5 mL) and stirred for 5 min. Then, 2M HCl (10 mL) was added and the reaction was extracted with DCM (3 x 20 mL). The organic phases were collected and washed with 2M HCl, 1M NaOH, and brine, dried over anhydrous $MgSO_4$, filtered and the solvent was removed under reduced pressure. The residue was then purified *via* flash column chromatography on silica gel (ethyl acetate in cyclohexane) to afford product **4l** (147 mg, 55%) as a white solid.

^{1}H NMR (400 MHz, $CDCl_3$) δ 7.90 (dt, *J* = 1.5, 0.8 Hz, 1H), 7.72 (d, *J* = 2.2 Hz, 1H), 7.61 (dd, *J* = 8.1, 0.7 Hz, 1H), 7.55 (dd, *J* = 8.1, 1.5 Hz, 1H), 7.39 – 7.31 (m, 2H), 7.29 – 7.18 (m, 3H), 6.80 (dd, *J* = 2.2, 1.0 Hz, 1H), 6.16 (s, 1H), 3.76 (td, *J* = 6.9, 5.9 Hz, 2H), 2.96 (t, *J* = 6.9 Hz, 2H).

^{13}C NMR (126 MHz, $CDCl_3$) δ 167.5, 154.8, 147.4, 139.1, 131.2, 130.5, 129.0 (2C), 128.9 (2C), 126.8, 121.5, 121.3, 110.7, 106.8, 41.4, 35.9.

HRMS (ESI) m/z: $[M+H]^+$ Calculated for $C_{17}H_{16}NO_2^+$ 266.1176; Found 266.1169.

6-Vinylbenzofuran ***(4m)***

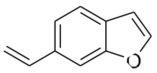

In an argon-filled glovebox, SPhos (18.5 mg, 0.045 mmol, 0.06 equiv) and palladium diacetate (3.37 mg, 0.015 mmol, 0.02 equiv) were weighed into a Schlenk tube. Outside of the glovebox, under nitrogen atmosphere, potassium vinyltrifluoroborate (100 mg, 0.75 mmol, 1.00 equiv), caesium carbonate (733 mg, 2.25 mmol, 3.00 equiv), and 6-bromobenzofuran (148 mg, 0.75 mmol, 1.00 equiv) were added, followed by THF (2.7 mL) and water (0.30 mL). The reaction mixture was stirred at 85 °C for 16h. After cooling to room temperature, water (10 mL) was added and extracted with ethyl acetate (3 x 20 mL). The combined organic layers were washed with brine, dried over $MgSO_4$, and concentrated *in vacuo*. The residue was purified *via* flash column chromatography on silica gel (cyclohexane) to afford product **4m** (61 mg, 47%) as a colorless oil.

^{1}H NMR (400 MHz, $CDCl_3$) δ 7.62 (d, *J* = 2.2 Hz, 1H), 7.57 – 7.51 (m, 2H), 7.34 (dd, *J* = 8.2, 1.4 Hz, 1H), 6.82 (dd, *J* = 17.5, 10.9 Hz, 1H), 6.75 (dd, *J* = 2.2, 1.0 Hz, 1H), 5.79 (dd, *J* = 17.6, 0.8 Hz, 1H), 5.26 (dd, *J* = 10.8, 0.8 Hz, 1H).

^{13}C NMR (101 MHz, $CDCl_3$) δ 155.55, 145.68, 137.12, 134.59, 127.33, 121.51, 121.09, 113.51, 109.12, 106.73.

HRMS (EI) m/z: $[M]^+$ Calculated for $C_{10}H_8O^+$ 144.0570; Found 144.0569.

The spectral data are consistent with those reported in the literature.[60]

Synthesis of 3-substituted Benzofurans

*3-Isopropylbenzofuran (**SI-12**)*

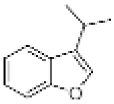

In a 10 mL Schlenk tube was added palladium diacetate (23 mg, 0.1 mmol, 0.1 equiv), 1-iodo-2-((3-methylbut-2-en-1-yl)oxy)benzene (288 mg, 1.0 mmol, 1.0 equiv), and diisopropylethylamine (388 mg, 516 μL, 3.0 mmol, 3.0 equiv) and propionitrile (1 M). The reaction mixture was heated to 100 °C overnight. The reaction was then cooled to room temperature and passed through a pad of celite. The solvent was removed under vacuum. Flash column chromatography on silica gel (cyclohexane) of the crude afforded the product **SI-12** as a colourless oil (75 mg, 47%).

^{1}H NMR (400 MHz, $CDCl_3$) δ 7.60 (ddd, *J* = 7.5, 1.6, 0.7 Hz, 1H), 7.46 (ddd, *J* = 8.1, 1.2, 0.7 Hz, 1H), 7.37 (d, *J* = 1.1 Hz, 1H), 7.30 – 7.25 (m, 1H), 7.23 (td, *J* = 7.4, 1.2 Hz, 1H), 3.10 (heptd, *J* = 6.9, 1.1 Hz, 1H), 1.37 (d, *J* = 6.9 Hz, 6H).

^{13}C NMR (101 MHz, $CDCl_3$) δ 155.7, 139.8, 127.8, 127.5, 124.1, 122.2, 120.3, 111.6, 24.8, 22.6 (2C).

HRMS (EI) m/z: $[M]^+$ Calculated for $C_{11}H_{12}O^+$ 160.0883; Found 160.0883.

The spectral data are consistent with those reported in the literature. [40]

*3-Benzylbenzofuran (**SI-13**)*

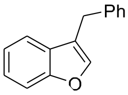

In a 10 mL Schlenk flask under nitrogen atmosphere, bis(acetonitrile)palladium dichloride (13 mg, 0.05 mmol, 0.05 equiv), sodium carbonate (265 mg, 180 μL, 2.5 mmol, 2.5 equiv), sodium formate (68 mg, 38 μL, 1.0 mmol, 1.0 equiv), and tetra(but-1-yl)ammonium chloride (306 mg, 1.10 mmol, 1.1 equiv) were prepared. A deoxygenated solution of 1-(cinnamyloxy)-2-iodobenzene (336 mg, 1.0 mmol. 1.0 equiv) in DMF (0.5 M) was then added. The crude was extracted with ethyl acetate. Flash column chromatography on silica gel (cyclohexane) of the crude afforded the product **SI-13** as a yellow solid (118 mg, 57%).

^{1}H NMR (400 MHz, $CDCl_3$) δ 7.46 (dt, *J* = 8.2, 0.9 Hz, 1H), 7.41 (ddd, *J* = 7.7, 1.4, 0.7 Hz, 1H), 7.38 (t, *J* = 1.2 Hz, 1H), 7.35 – 7.19 (m, 6H), 7.18 (ddd, *J* = 7.7, 7.2, 1.0 Hz, 1H), 4.03 (d, *J* = 1.2 Hz, 2H).

^{13}C NMR (101 MHz, $CDCl_3$) δ 155.7, 142.3, 139.3, 128.8 (2C), 128.7 (2C), 128.2, 126.5, 124.4, 122.5, 120.0, 119.8, 111.6, 30.1.

HRMS (EI) m/z: $[M]^+$ Calculated for $C_{15}H_{12}O^+$ 208.0883; Found 208.0880.

The spectral data are consistent with those reported in the literature. [41]

*3-Phenylbenzofuran (**1b**)*

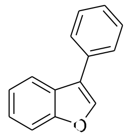

In a 25-mL Schlenk tube under nitrogen atmosphere, palladium diacetate (9.1 mg, 0.04 mmol, 0.02 equiv), tri-*tert*-butylphosphonium tetrafluoroborate (15 mg, 0.05 mmol, 0.03 equiv), phenylboronic acid (297 mg, 2.44 mmol, 1.2 equiv), and 3-bromobenzofuran (400 mg, 2.03 mmol, 1.0 equiv) were dissolved in 5.5 mL degassed *n*-butanol and the mixture was stirred at room temperature for 15 minutes. Then, a degassed solution of sodium hydroxide (138 mg, 3.45 mmol, 1.7 equiv) in 1.4 mL water was added and the reaction mixture was stirred at room temperature overnight. After completion of the reaction, water (15 mL) was added, followed by extraction with DCM (3x20 mL). The combined organic phases were dried over $MgSO_4$, filtered and concentrated under reduced pressure. Purification of the crude *via* flash column chromatography on silica gel (cyclohexane) afforded the product **1b** as yellow oil (353 mg, 90%).

^{1}H NMR (400 MHz, $CDCl_3$) δ 7.89-7.87 (m, 1H), 7.83 (s, 1H), 7.75 – 7.63 (m, 2H), 7.63 – 7.56 (m, 1H), 7.56 – 7.45 (m, 2H), 7.44 – 7.31 (m, 3H).

HRMS (EI) m/z: $[M]^+$ Calculated for $C_{14}H_{10}O^+$ 194.0726; Found 194.0723.

The spectral data are consistent with those reported in the literature. [42,43]

*6-Methoxy-3-methylbenzofuran (**1d**)*

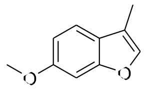

To a 10 mL Schlenk flask under nitrogen atmosphere, palladium diacetate (23 mg, 0.1 mmol, 0.1 equiv), sodium carbonate (265 mg, 2.5 mmol, 2.5 equiv), sodium formate (68 mg, 1.0 mmol, 1.0 equiv), and tetra(but-1-yl)ammonium chloride (306 mg, 1.1 mmol, 1.1 equiv) were added. A deoxygenated solution of 2-(allyloxy)-1-iodo-4-methoxybenzene (290 mg, 1.0 mmol, 1.0 equiv) in DMF (2 mL) was added. Flash column chromatography on silica gel (cyclohexane) of the crude afforded product **1d** as a white solid (40.3 mg, 25%).

^{1}H NMR (400 MHz, $CDCl_3$) δ 7.37 (d, J = 8.5 Hz, 1H), 7.32 (q, J = 1.3 Hz, 1H), 6.99 (dd, J = 2.2, 0.5 Hz, 1H), 6.88 (dd, J = 8.5, 2.2 Hz, 1H), 3.85 (s, 3H), 2.21 (d, J = 1.3 Hz, 3H).

^{13}C NMR (101 MHz, $CDCl_3$) δ 158.1, 156.4, 140.6, 122.6, 119.6, 115.6, 111.4, 96.1, 55.9, 8.1.

HRMS (EI) m/z: $[M]^+$ Calculated for $C_{10}H_{10}O_2^+$ 162.0681; Found 162.0675.

The spectral data are consistent with those reported in the literature. [41]

*Methyl 3-methylbenzofuran-5-carboxylate (**1e**)*

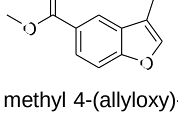

To a 10 mL Schlenk flask under nitrogen atmosphere, palladium diacetate (23 mg, 0.10 mmol, 0.05 equiv), sodium carbonate (530 mg, 5.0 mmol, 2.5 equiv), sodium formate (136 mg, 2.0 mmol, 1.0 equiv), and tetra(but-1-yl)ammonium chloride (611 mg, 2.2 mmol, 1.1 equiv) were added. A deoxygenated solution of methyl 4-(allyloxy)-3-iodobenzoate (636 mg, 2.0 mmol, 1.0 equiv) in DMF (2 mL) was added. The reaction was heated to 80°C for 48h. Flash column chromatography on silica gel (ethyl acetate in cyclohexane) of the crude afforded product **1e** as a white solid (99.6 mg, 26%).

^{1}H NMR (400 MHz, $CDCl_3$) δ 8.28 (dd, J = 1.8, 0.7 Hz, 1H), 8.02 (ddd, J = 8.7, 1.8, 0.4 Hz, 1H), 7.50 – 7.43 (m, 2H), 3.95 (s, 3H), 2.28 (d, J = 1.4 Hz, 3H).

^{13}C NMR (101 MHz, $CDCl_3$) δ 167.6, 158.0, 142.8, 129.2, 126.1, 124.8, 122.2, 116.5, 111.3, 52.2, 8.0.

HRMS (ESI) m/z: $[M+Na]^+$ Calculated for $C_{11}H_{10}NaO_3^+$ 213.0522; Found 213.0519.

The spectral data are consistent with those reported in the literature. [44]

*Methyl 3-methylbenzofuran-6-carboxylate (**1f**)*

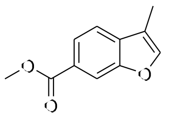

In a 10 mL Schlenk flask under nitrogen atmosphere, palladium diacetate (34 mg, 0.15 mmol, 0.05 equiv), sodium carbonate (795 mg, 7.5 mmol, 2.5 equiv), and tetra(but-1-yl)ammonium chloride (917 mg, 3.3 mmol, 1.1 equiv) were prepared. A deoxygenated solution of methyl 3-(allyloxy)-4-iodobenzoate (954 mg, 3.0 mmol, 1.0 equiv) in DMF (6 mL) was added. The reaction was heated to 80°C for 48h. Flash column chromatography on silica gel (ethyl acetate in cyclohexane) of the crude afforded the product **1f** as a white solid (258 mg, 45%).

^{1}H NMR (400 MHz, $CDCl_3$) δ 8.15 (dd, J = 1.4, 0.6 Hz, 1H), 7.96 (dd, J = 8.2, 1.4 Hz, 1H), 7.58 – 7.53 (m, 2H), 3.95 (s, 3H), 2.27 (d, J = 1.3 Hz, 3H).

^{13}C NMR (101 MHz, $CDCl_3$) δ 167.6, 154.9, 144.5, 133.4, 126.3, 123.8, 119.2, 116.1, 113.2, 52.3, 8.0.

HRMS (ESI) m/z: $[M+H]^+$ Calculated for $C_{11}H_{11}O_3^+$ 191.0703; Found 191.0703.

*3-Methyl-5-phenylbenzofuran (**1g**)*

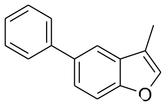

To a 10 mL Schlenk flask under nitrogen atmosphere, tetrakis(triphenylphosphine)palladium(0) (11.0 mg, 9.50 µmol, 0.019 equiv), sodium carbonate (132 mg, 1.25 mmol, 2.5 equiv), and phenylboronic acid (318 mg, 0.62 mmol, 1.25 equiv) were added. A deoxygenated solution of 5-bromo-3-methylbenzofuran (69.4 µL, 106 mg, 0.500 mmol, 1.0 equiv) in water (1.75 mL) and DME (0.75 mL) was added. The reaction mixture was refluxed for 24 hours. After completion of the reaction, the reaction mixture was layer separated with ethyl acetate, and the organic layer was concentrated at a reduced pressure. Flash column chromatography on silica gel (ethyl acetate in cyclohexane) of the crude afforded the product **1g** as a white solid (99.6 mg, 26%).

^{1}H NMR (400 MHz, $CDCl_3$) δ 7.71 (dd, J = 1.6, 1.0 Hz, 1H), 7.65 – 7.62 (m, 2H), 7.52 – 7.50 (m, 2H), 7.48 – 7.43 (m, 3H), 7.37 – 7.32 (m, 1H), 2.29 (d, J = 1.4 Hz, 3H).

^{13}C NMR (101 MHz, $CDCl_3$) δ 155.0, 142.2, 142.0, 136.2, 129.7, 128.9 (2C), 127.6 (2C), 127.0, 123.9, 118.1, 116.0, 111.6, 8.1.

HRMS (EI) m/z: $[M]^+$ Calculated for $C_{15}H_{12}O^+$ 208.0883; Found 208.0880.

The spectral data are consistent with those reported in the literature. [45]

*3-Methyl-6-phenylbenzofuran (**1h**)*

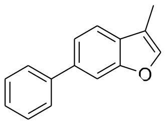

3-Methyl-6-phenylbenzofuran (**1h**) was prepared according to the general procedure C using 6-bromo-3-methylbenzofuran (106 mg, 0.50 mmol, 1.0 equiv) and phenylboronic acid (61 mg, 0.50 mmol, 1.0 equiv). Flash column chromatography on silica gel (ethyl acetate in cyclohexane) of the crude afforded the product **1h** as a white solid (72.2 mg, 69%).

^{1}H NMR (400 MHz, $CDCl_3$) δ 7.70 – 7.63 (m, 3H), 7.58 (dd, *J* = 8.0, 0.7 Hz, 1H), 7.51 (dd, *J* = 8.1, 1.5 Hz, 1H), 7.49 – 7.43 (m, 3H), 7.35 (ddt, *J* = 8.1, 6.7, 1.3 Hz, 1H), 2.28 (d, *J* = 1.3 Hz, 3H).

^{13}C NMR (101 MHz, $CDCl_3$) δ 156.0, 142.1, 141.6, 138.0, 128.9 (2C), 128.4, 127.6 (2C), 127.2, 122.0, 119.7, 115.7, 110.0, 8.1.

HRMS (EI) m/z: $[M]^+$ Calculated for $C_{15}H_{12}O^+$ 208.0883; Found 208.0879.

The spectral data are consistent with those reported in the literature. [45]

*6-(3-Chlorophenyl)-3-methylbenzofuran (**1i**)*

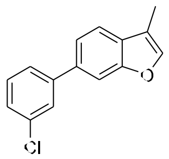

6-(3-Chlorophenyl)-3-methylbenzofuran (**1i**) was prepared according to the general procedure C using 6-bromo-3-methylbenzofuran (106 mg, 0.50 mmol, 1.0 equiv) and (3-chlorophenyl)boronic acid (78 mg, 0.50 mmol, 1.0 equiv). Flash column chromatography on silica gel (ethyl acetate in cyclohexane) of the crude afforded the product **1i** as a white solid (77 mg, 63%).

^{1}H NMR (400 MHz, $CDCl_3$) δ 7.45 (dd, J = 1.5, 0.6 Hz, 1H), 7.45 – 7.43 (m, 1H), 7.39 (dd, J = 8.0, 0.7 Hz, 1H), 7.32 (ddd, J = 7.6, 1.8, 1.2 Hz, 1H), 7.29 – 7.25 (m, 2H), 7.18 (td, J = 7.8, 0.5 Hz, 1H), 7.13 (ddd, J = 8.0, 2.0, 1.2 Hz, 1H), 2.08 (d, J = 1.4 Hz, 3H).

^{13}C NMR (101 MHz, $CDCl_3$) δ 156.0, 143.4, 142.4, 136.5, 134.8, 130.1, 128.9, 127.6, 127.2, 125.7, 121.9, 119.8, 115.8, 110.1, 8.1.

HRMS (EI) m/z: $[M]^+$ Calculated for $C_{15}H_{11}ClO^+$ 242.0493; Found 242.0487.

*6-(4-chlorophenyl)-3-methylbenzofuran (**1j**)*

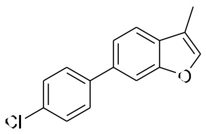

6-(4-chlorophenyl)-3-methylbenzofuran (**1j**) was prepared according to the general procedure C using 6-bromo-3-methylbenzofuran (158 mg, 0.75 mmol, 1.5 equiv) and (4-chlorophenyl)boronic acid (78 mg, 0.50 mmol, 1.0 equiv). Flash column chromatography on silica gel (cyclohexane) of the crude afforded the product **1j** as a white solid (88.7 mg, 73%).

^{1}H NMR (400 MHz, $CDCl_3$) δ 7.63 (dd, *J* = 1.6, 0.6 Hz, 1H), 7.59 – 7.55 (m, 3H), 7.47 – 7.44 (m, 2H), 7.43 – 7.40 (m, 2H), 2.27 (d, *J* = 1.3 Hz, 3H).

^{13}C NMR (101 MHz, $CDCl_3$) δ 156.0, 142.3, 140.0, 136. 7, 133.3, 129.1 (2C), 128.7 (2C), 128.7, 121.8, 119.8, 115.8, 109.9, 8.1.

HRMS (ESI) m/z: $[M+H]^+$ Calculated for $C_{15}H_{12}ClO^+$ 243.0571; Found 243.0572.

*3-methyl-6-(2-(trifluoromethyl)phenyl)benzofuran (**1k**)*

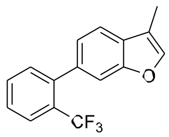

3-methyl-6-(2-(trifluoromethyl)phenyl)benzofuran (**1k**) was prepared according to the general procedure C using 6-bromo-3-methylbenzofuran (211 mg, 1.0 mmol, 1.0 equiv) and (2-(trifluoromethyl)phenyl)boronic acid (190 mg, 1.0 mmol, 1.0 equiv). Flash column chromatography on silica gel (ethyl acetate in cyclohexane) of the crude afforded the product **1k** as a colourless oil (106.8 mg, 39%).

^{1}H NMR (400 MHz, $CDCl_3$) δ 7.76 (ddq, J = 7.8, 1.5, 0.5 Hz, 1H), 7.59 – 7.52 (m, 2H), 7.50 – 7.42 (m, 3H), 7.38 (ddt, J = 7.6, 1.4, 0.7 Hz, 1H), 7.22 (ddq, J = 8.0, 1.3, 0.6 Hz, 1H), 2.29 (d, J = 1.3 Hz, 3H).

^{13}C NMR (101 MHz, $CDCl_3$) δ 154.9, 142.2, 141.6, 136.1, 132.6, 131.4, 128.6, 127.4, 126.2 (q, J = 5.4 Hz), 125.7, 123.8 (d, J = 1.7 Hz), 123.0, 118.7, 115.8, 112.3 – 111.6 (m), 8.1.

^{19}F NMR (376 MHz, $CDCl_3$) δ -56.77.

HRMS (ESI) m/z: $[M+Na]^+$ Calculated for $C_{16}H_{11}F_3NaO^+$ 299.0654; Found 299.0655.

3-*methyl-6-(phenylethynyl)benzofuran* ***(1l)***

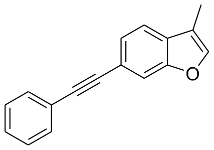

To a 10 mL schlenk flask under nitrogen atmosphere, 6-bromo-3-methylbenzofuran (106 mg, 1.5 mmol, 1.0 equiv), ethynylbenzene (325 mg, 0.35 mL, 3.12 mmol, 2.08 equiv), tetrakis(triphenylphosphine)palladium(0) (90 mg, 0.078 mmol, 0.052 equiv), cuprous iodide (30 mg, 0.16 mmol, 0.10 equiv) and triethylamine (4.55 g, 6.3 mL, 45.0 mmol, 30 equiv) were added. The mixture was heated at 90 °C for 24 h under nitrogen. After cooling, the reaction mixture was diluted with Et_2O, filtered through a pad of celite and washed with brine. The organic layer was dried over Na_2SO_4, filtered and concentrated in vacuo. Flash column chromatography on silica gel (cyclohexane) of the crude afforded the product **1l** as an orange solid (96 mg, 28%).

^{1}H NMR (400 MHz, $CDCl_3$) δ 7.76 (dd, *J* = 1.3, 0.7 Hz, 1H), 7.70 – 7.66 (m, 2H), 7.59 (dd, *J* = 8.0, 0.7 Hz, 1H), 7.56 – 7.54 (m, 2H), 7.52 – 7.43 (m, 3H), 2.35 (d, *J* = 1.3 Hz, 3H).

^{13}C NMR (126 MHz, $CDCl_3$) δ 155.0, 142.9, 131.7 (2C), 129.5, 128.5 (2C), 128.3, 126.3, 123.6, 119.4, 119.0, 116.0, 114.7, 90.1, 89.1, 8.00.

HRMS (EI) m/z: $[M+H]^+$ Calculated for $C_{17}H_{13}O^+$ 233.0961; Found 233.0961.

3-(3-Methylbenzofuran-5-yl)pyridine ***(1m)***

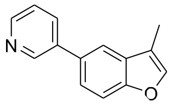

3-(3-methylbenzofuran-5-yl)pyridine (**1m**) was prepared according to the general procedure C using 5-bromo-3-methylbenzofuran (258 mg, 1.2 mmol, 1.0 equiv) and pyridin-3-ylboronic acid (150 mg, 1.2 mmol, 1.0 equiv). Flash column chromatography on silica gel (ethyl acetate in cyclohexane) of the crude afforded the product **1m** as yellow solid (205 mg, 80%).

^{1}H NMR (400 MHz, $CDCl_3$) δ 8.90 – 8.88 (m, 1H), 8.60 – 8.58 (m, 1H), 7.94 – 7.90 (m, 1H), 7.71 – 7.69 (m, 1H), 7.56 – 7.53 (m, 1H), 7.50 – 7.46 (m, 2H), 7.41 – 7.36 (m, 1H), 2.29 (s, 3H).

^{13}C NMR (101 MHz, $CDCl_3$) δ 155.4, 148.6, 148.1, 142.5, 137.5, 134.9, 132.6, 130.0, 123.7, 123.7, 118.3, 116.0, 112.0, 8.0.

HRMS (EI) m/z: $[M]^+$ Calculated for $C_{14}H_{11}NO^+$ 209.0835; Found 209.0833.

3-(3-Methylbenzofuran-6-yl)pyridine ***(1n)***

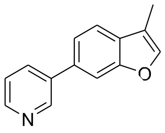

3-(3-methylbenzofuran-6-yl)pyridine (**1n**) was prepared according to the general procedure C using 6-bromo-3-methylbenzofuran (258 mg, 1.2 mmol, 1.0 equiv) and pyridin-3-ylboronic acid (150 mg, 1.2 mmol, 1.0 equiv). Flash column chromatography on silica gel (ethyl acetate in cyclohexane) of the crude afforded the product **1n** as colorless solid (195 mg, 76%).

^{1}H NMR (400 MHz, $CDCl_3$) δ 9.07 – 8.74 (m, 1H), 8.59 (dd, *J* = 4.9, 1.6 Hz, 1H), 8.22 – 7.82 (m, 1H), 7.83 – 7.54 (m, 2H), 7.61 – 7.44 (m, 2H), 7.43 – 7.30 (m, 1H), 2.28 (d, *J* = 1.3 Hz, 3H).

^{13}C NMR (101 MHz, $CDCl_3$) δ 156.0, 148.4, 148.1, 142.6, 137.2, 135.0, 134.2, 129.2, 123.8, 121.8, 120.2, 115.8, 110.1, 8.1.

HRMS (EI) m/z: $[M]^+$ Calculated for $C_{14}H_{11}NO^+$ 209.0835; Found 209.0832.

3-Methyl-5-vinylbenzofuran ***(1o)***

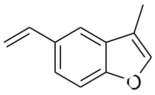

In an argon-filled glovebox, SPhos (18.5 mg, 0.045 mmol, 0.06 equiv) and palladium diacetate (3.37 mg, 0.015 mmol, 0.02 equiv) were weighed into a Schlenk tube. Outside of the glovebox, under nitrogen atmosphere, potassium vinyltrifluoroborate (100 mg, 0.75 mmol, 1.00 equiv), caesium carbonate (733 mg, 2.25 mmol, 3.00 equiv), and 5-bromo-3-methylbenzofuran (158 mg, 0.75 mmol, 1.00 equiv) were added, followed by THF (2.7 mL) and water (0.30 mL). The reaction mixture was stirred at 85 °C for 16h. After cooling to room temperature, water (10 mL) was added and extracted with ethyl acetate (3 x 20 mL). The combined organic layers were washed with brine, dried over $MgSO_4$, and concentrated *in vacuo*. The residue was purified *via* flash column chromatography on silica gel (cyclohexane) to afford product **1o** (75 mg, 60%) as a colorless oil.

^{1}H NMR (400 MHz, $CDCl_3$) δ 7.54 (d, *J* = 1.5 Hz, 1H), 7.44 – 7.30 (m, 3H), 6.84 (ddd, *J* = 17.6, 10.9, 1.8 Hz, 1H), 5.74 (ddd, *J* = 16.9, 1.9, 0.8 Hz, 1H), 5.22 (ddd, *J* = 10.9, 1.8, 0.8 Hz, 1H), 2.25 (d, *J* = 1.3 Hz, 3H).

^{13}C NMR (101 MHz, $CDCl_3$) δ 155.26, 142.06, 137.27, 132.43, 129.42, 122.66, 117.35, 115.90, 112.60, 111.40, 8.02.

HRMS (EI) m/z: $[M]^+$ Calculated for $C_{11}H_{10}O^+$ 158.0726; Found 158.0722.

3-Methyl-6-vinylbenzofuran ***(1p)***

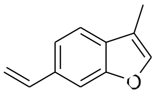

In an argon-filled glovebox, SPhos (18.5 mg, 0.045 mmol, 0.06 equiv) and palladium diacetate (3.37 mg, 0.015 mmol, 0.02 equiv) were weighed into a Schlenk tube. Outside of the glovebox, under nitrogen atmosphere, potassium vinyltrifluoroborate (100 mg, 0.75 mmol, 1.00 equiv), caesium carbonate (733 mg, 2.25 mmol, 3.00 equiv), and 6-bromo-3-methylbenzofuran (158 mg, 0.75 mmol, 1.00 equiv) were added, followed by THF (2.7 mL) and water (0.30 mL). The reaction mixture was stirred at 85 °C for 16h. After cooling to room temperature, water (10 mL) was added and extracted with ethyl acetate (3 x 20 mL). The combined organic layers were washed with brine, dried over $MgSO_4$, and concentrated *in vacuo*. The residue was purified *via* flash column chromatography on silica gel (cyclohexane) to afford product **1p** (77 mg, 65%) as a colorless oil.

^{1}H NMR (400 MHz, $CDCl_3$) δ 7.49 (dd, *J* = 1.4, 0.6 Hz, 1H), 7.46 (dd, *J* = 8.1, 0.7 Hz, 1H), 7.40 (q, *J* = 1.3 Hz, 1H), 7.35 – 7.31 (m, 1H), 7.05 – 6.37 (m, 1H), 6.12 – 5.37 (m, 1H), 5.25 (dd, *J* = 10.8, 0.9 Hz, 1H), 2.24 (d, *J* = 1.3 Hz, 4H).

^{13}C NMR (101 MHz, $CDCl_3$) δ 155.72, 142.05, 137.10, 134.29, 128.86, 120.91, 119.22, 115.71, 113.18, 108.89, 7.91.

HRMS (EI) m/z: $[M]^+$ Calculated for $C_{11}H_{10}O^+$ 158.0726; Found 158.0723.

C-to-N Atom Swap

General Procedure D to Access Benzisoxazoles from Benzofurans

In an 8-mL screw-cap vial equipped with a magnetic stirring bar and an oxygen balloon, benzofuran (0.25 mmol, 1 equiv), $Ru(phen)_3Cl_2$ • x H_2O (5 mg, 7.5 µmol, 3 mol%) and DIPEA (6.5 mg, 8.7 µL, 0.05 mmol, 0.2 equiv) were dissolved in acetonitrile (625 µL). Then, water (270 µL, 15 mmol, 60 equiv) was added to the reaction mixture. The vial was placed in the photoreactor (EvoluChem 450PF LED, 18W) and stirred at 35° C for 16h. Then, water (355 µL; total concentration = 0.2 M with ACN:H_2O 1:1) and HOSA (42 mg, 0.375 mmol, 1.5 equiv) were added at 0 °C. The mixture was stirred for 1h before $NaHCO_3$ (53 mg, 0.625 mmol, 2.5 equiv) was added and the mixture was stirred at room temperature for 1h. The crude was purified by flash chromatography on silica gel with ethyl acetate in cyclohexane as eluent.

General Procedure E to Access Benzoxazoles from 3-Substituted Benzofurans

In an 8-mL screw-cap vial equipped with a magnetic stirring bar and an oxygen balloon, benzofuran (0.25 mmol, 1.0 equiv), $Ru(phen)_3Cl_2 \cdot x\, H_2O$ (2.7 mg, 0.004 mmol, 1.5 mol%) and DIPEA (22 µL, 0.13 mmol, 0.5 equiv) were dissolved in acetonitrile (2.5 mL). The vial was placed in the photoreactor (EvoluChem 450PF LED, 18W) and stirred at 35° C for 16h. Then, the vial was taken out of the photoreactor, followed by addition of hydroxylamine-O-sulfonic acid (85 mg, 0.75 mmol, 3.0 equiv) and methanesulfonic acid (51 µL, 0.75 mmol, 3.0 equiv). The reaction mixture was stirred at 90 °C for 3h. After cooling to room temperature, sat. $NaHCO_3$ solution was added, extracted with DCM (3x), and the combined organic phases were dried over $MgSO_4$, filtered and concentrated under reduced pressure. The crude was purified *via* flash column chromatography on silica gel using ethyl acetate in cyclohexane as eluent.

General Procedure F to Access Benzisoxazoles from 3-Substituted Benzofurans

In an 8-mL screw-cap vial equipped with a magnetic stirring bar and an oxygen balloon, benzofuran (0.25 mmol, 1.0 equiv), $Ru(phen)_3Cl_2 \cdot x\, H_2O$ (2.7 mg, 0.004 mmol, 1.5 mol%) and DIPEA (22 µL, 0.13 mmol, 0.5 equiv) were dissolved in acetonitrile (2.5 mL). The vial was placed in the photoreactor (EvoluChem 450PF LED, 18W) and stirred at 35° C for 16h. Then, the solvent was removed under reduced pressure, 7M NH_3 in methanol (360 µL, 2.5 mmol, 10 equiv) was added and the reaction mixture was stirred at room temperature for 3h. The crude mixture was concentrated under reduced pressure and redissolved in tetrahydrofuran (2.5 mL), followed by addition of K_2CO_3 (69 mg, 0.50 mmol, 2.0 equiv) and N-chlorosuccinimide (50 mg, 0.38 mmol, 1.5 equiv). The reaction mixture was stirred at room temperature for 16h. Then, water was added, extracted with DCM (3x), and the combined organic phases were dried over $MgSO_4$, filtered and concentrated under reduced pressure. The crude was purified by flash column chromatography on silica gel using ethyl acetate in cyclohexane as eluent.

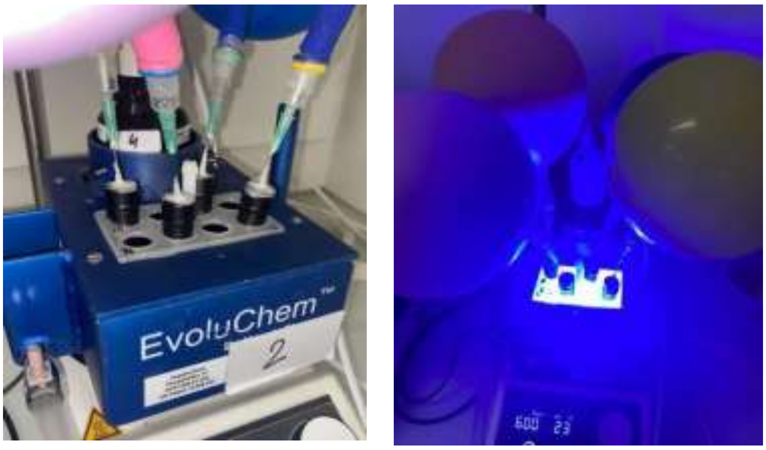

Figure S1. Photochemical setup used for general procedures D to F.

Benzisoxazoles from Benzofurans

*Benzo[*d*]isoxazole (**5a**)*

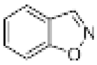

Benzo[*d*]isoxazole (**5a**) was prepared according to the general procedure using benzofuran (**1a**) (30 mg, 0.25 mmol). Flash column chromatography on silica gel (ethyl acetate in cyclohexane) of the crude reaction mixture afforded the product **5a** as yellow oil (3 mg, 10%). The low isolated yield was attributed to the volatility of **5a**.

^{1}H NMR (400 MHz, $CDCl_3$) δ 8.72 (d, J = 1.1 Hz, 1H), 7.75 (dt, J = 7.9, 1.0 Hz, 1H), 7.72 – 7.62 (m, 1H), 7.61 – 7.50 (m, 1H), 7.34 (ddd, J = 7.9, 6.9, 1.0 Hz, 1H).

The spectral data are consistent with the commercially available authentic sample.

5-*Phenylbenzo[*d*]isoxazole (5b)*

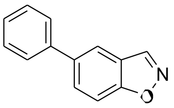

5-Phenylbenzo[*d*]isoxazole (**5b**) was prepared according to the general procedure D using 5-phenylbenzofuran (**4b**) (49 mg, 0.25 mmol). Flash column chromatography on silica gel (ethyl acetate in cyclohexane) of the crude reaction mixture afforded the product **5b** as a yellow oil (36 mg, 73%).

^{1}H NMR (500 MHz, $CDCl_3$) δ 8.76 (d, *J* = 1.1 Hz, 1H), 7.90 (dd, *J* = 1.8, 0.8 Hz, 1H), 7.81 (dd, *J* = 8.7, 1.8 Hz, 1H), 7.68 (dt, *J* = 8.7, 0.9 Hz, 1H), 7.62 – 7.58 (m, 2H), 7.50 – 7.45 (m, 2H), 7.42 – 7.37 (m, 1H).

^{13}C NMR (126 MHz, $CDCl_3$) δ 161.9, 146.6, 140.5, 137.8, 130.2, 129.1 (2C), 127.7, 127.6 (2C), 122.2, 120.2, 110.1.

HRMS (EI) m/z: $[M]^+$ Calculated for $C_{13}H_9NO^+$ 195.0679; Found 195.0676.

*Methyl benzo[*d*]isoxazole-5-carboxylate (5c)*

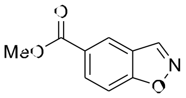

Methyl benzo[*d*]isoxazole-5-carboxylate (**5c**) was prepared according to the general procedure D using methyl benzofuran-5-carboxylate (**4c**) (44 mg, 0.25 mmol). Flash column chromatography on silica gel (ethyl acetate in cyclohexane) of the crude reaction mixture afforded the product **5c** as a yellow oil (22 mg, 50%). 21% starting material was recovered.

^{1}H NMR (400 MHz, CDCl3) δ 8.79 (d, J = 1.1 Hz, 1H), 8.49 (dd, J = 1.6, 0.8 Hz, 1H), 8.27 (dd, J = 8.8, 1.6 Hz, 1H), 7.65 (dt, J = 8.9, 0.9 Hz, 1H), 3.96 (s, 3H).

^{13}C NMR (101 MHz, $CDCl_3$) δ 166.3, 164.4, 146.8, 131.5, 126.6, 125.1, 121.8, 109.8, 52.6.

HRMS (EI) m/z: $[M]^+$ Calculated for $C_9H_7NO_3^+$ 177.0420; Found 177.0419.

tert-*Butyl benzo[*d*]isoxazol-5-ylcarbamate (5d)*

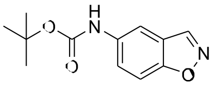

Tert-Butyl benzo[*d*]isoxazol-5-ylcarbamate (**5d**) was prepared according to the general procedure D using *tert*-butyl benzofuran-5-ylcarbamate (**4d**) (58 mg, 0.25 mmol). Flash column chromatography on silica gel (ethyl acetate in cyclohexane) of the crude reaction mixture afforded the product **5d** as a yellow oil (18 mg, 31%). 26% starting material was recovered.

^{1}H NMR (400 MHz, $CDCl_3$) δ 8.63 (d, *J* = 1.0 Hz, 1H), 7.96 (s, 1H), 7.52 (dt, *J* = 8.9, 0.9 Hz, 1H), 7.36 (dd, *J* = 8.9, 2.1 Hz, 1H), 6.67 (s, 1H), 1.53 (s, 9H).

^{13}C NMR (101 MHz, $CDCl_3$) δ 159.0, 153.1, 146.4, 134.7, 122.9, 122.0, 110.9, 110.0, 81.1, 28.5 (3C).

HRMS (ESI) m/z: $[M+H]^+$ Calculated for $C_{12}H_{15}N_2O_3^+$ 235.1077; Found 235.1077.

6-*Phenylbenzo[*d*]isoxazole (5e)*

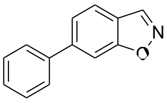

6-Phenylbenzo[*d*]isoxazole (**5e**) was prepared according to the general procedure D using 6-phenylbenzofuran (**4e**) (29 mg, 0.15 mmol). Flash column chromatography on silica gel (ethyl acetate in cyclohexane) of the crude reaction mixture afforded the product **5e** as a yellow oil (23 mg, 78%).

^{1}H NMR (400 MHz, $CDCl_3$) δ 8.73 (d, *J* = 1.1 Hz, 1H), 7.82 – 7.76 (m, 2H), 7.68 – 7.63 (m, 2H), 7.58 (dd, *J* = 8.2, 1.4 Hz, 1H), 7.52 – 7.47 (m, 2H), 7.45 – 7.39 (m, 1H).

^{13}C NMR ^{13}C NMR (101 MHz, $CDCl_3$) δ 163.2, 146.2, 144.02, 140.3, 129.2 (2C), 128.3, 127.8 (2C), 123.9, 122.1, 120.5, 108.1.

HRMS (EI) m/z: $[M]^+$ Calculated for $C_{13}H_9NO^+$ 195.0679; Found 195.0676.

*6-Methoxybenzo[*d*]isoxazole (**5f**)*

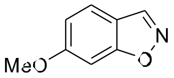

6-Methoxybenzo[*d*]isoxazole (**5f**) was prepared according to the general procedure D using 6-methoxybenzofuran (**4f**) (37 mg, 32 µL, 0.25 mmol). Flash column chromatography on silica gel (ethyl acetate in cyclohexane) of the crude reaction mixture afforded the product **5f** as a yellow oil (6.5 mg, 17%).

^{1}H NMR (500 MHz, $CDCl_3$) δ 8.58 (d, *J* = 1.1 Hz, 1H), 7.56 (dd, *J* = 8.7, 0.5 Hz, 1H), 7.10 – 7.02 (m, 1H), 6.94 (dd, *J* = 8.7, 2.1 Hz, 1H), 3.90 (s, 3H).

^{13}C NMR (126 MHz, $CDCl_3$) δ 164.3, 162.5, 146.0, 122.2, 114.9, 114.8, 92.5, 55.9.

HRMS (ESI) m/z: $[M+H]^+$ Calculated for $C_8H_8NO_2^+$ 150.0550; Found 150.0548.

The spectral data are consistent with those reported in the literature. [46]

*4-Methoxy-7*H*-chromeno[6,7-*d*]isoxazol-7-one (**5g**)*

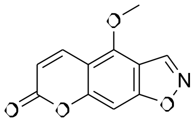

4-Methoxy-7*H*-chromeno[6,7-*d*]isoxazol-7-one (**5g**) was prepared according to the general procedure D using bergapten (**4g**) (54 mg, 0.25 mmol) in 0.1 M solvent. Flash column chromatography on silica gel (ethyl acetate in cyclohexane) of the crude reaction mixture afforded the product **5g** as a yellow oil (28 mg, 52%). 18% starting material was recovered.

^{1}H NMR (600 MHz, $CDCl_3$) δ 8.93 (d, *J* = 1.2 Hz, 1H), 8.14 (dd, *J* = 9.8, 0.7 Hz, 1H), 7.15 (dd, *J* = 1.2, 0.7 Hz, 1H), 6.31 (d, *J* = 9.8 Hz, 1H), 4.38 (s, 3H).

^{13}C NMR (151 MHz, $CDCl_3$) δ 164.9, 160.4, 156.8, 150.9, 144.6, 138.7, 113.3, 106.9, 105.9, 90.8, 60.1.

HRMS (ESI) m/z: $[M+H]^+$ Calculated for $C_{11}H_8NO_4^+$ 218.0448; Found 218.0451.

*6-(4-(Methylsulfonyl)phenyl)benzo[*d*]isoxazole (**5h**)*

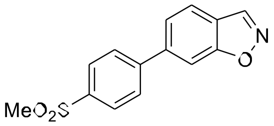

6-(4-(Methylsulfonyl)phenyl)benzo[*d*]isoxazole (**5h**) was prepared according to the general procedure D using 6-(4-(methylsulfonyl)phenyl)benzofuran (**4h**) (68 mg, 0.25 mmol). Flash column chromatography on silica gel (ethyl acetate in cyclohexane) of the crude reaction mixture afforded the product **5h** as a yellow oil (36 mg, 53%).

^{1}H NMR (500 MHz, CD_3CN) δ 8.93 (d, *J* = 1.1 Hz, 1H), 8.05 – 8.03 (m, 2H), 7.98 – 7.94 (m, 4H), 7.71 (dd, *J* = 8.3, 1.5 Hz, 1H), 3.12 (s, 3H).

^{13}C NMR (126 MHz, CD_3CN) δ 163.6, 147.7, 146.1, 142.5, 141.5, 129.6 (2C), 128.9 (2C), 124.8, 124.1, 122.6, 109.2, 44.5.

HRMS (EI) m/z: $[M]^+$ Calculated for $C_{14}H_{11}NO_3S^+$ 273.0454; Found 273.0452.

*5-(3-Chlorophenyl)benzo[*d*]isoxazole (**5i**)*

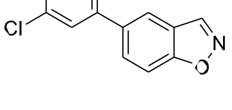

5-(3-Chlorophenyl)benzo[*d*]isoxazole (**5i**) was prepared according to the general procedure D using 5-(3-chlorophenyl)benzofuran (**4i**) (22 mg, 0.098 mmol). Flash column chromatography on silica gel (ethyl acetate in cyclohexane) of the crude reaction mixture afforded the product **5i** as a yellow oil (13 mg, 59%).

^{1}H NMR (400 MHz, $CDCl_3$) δ 8.76 (d, *J* = 1.0 Hz, 1H), 7.88 (dd, *J* = 1.8, 0.8 Hz, 1H), 7.77 (dd, *J* = 8.7, 1.8 Hz, 1H), 7.69 (ddd, *J* = 8.7, 1.1, 0.8 Hz, 1H), 7.59 (td, *J* = 1.9, 0.5 Hz, 1H), 7.47 (ddd, *J* = 7.5, 1.8, 1.4 Hz, 1H), 7.42 – 7.38 (m, 1H), 7.36 (ddd, *J* = 7.9, 2.0, 1.4 Hz, 1H).

^{13}C NMR (101 MHz, $CDCl_3$) δ 162.2, 146.5, 142.3, 136.4, 135.0, 130.3, 130.0, 127.7 (2C), 125.7, 122.3, 120.4, 110.3.

HRMS (EI) m/z: $[M]^+$ Calculated for $C_{13}H_8ClNO^+$ 229.0289; Found 229.0284.

*5-(2-(Trifluoromethyl)phenyl)benzo[*d*]isoxazole* ***(5j)***

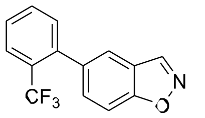

5-(2-(Trifluoromethyl)phenyl)benzo[*d*]isoxazole (**5j**) was prepared according to the general procedure D using 5-(2-(trifluoromethyl)phenyl)benzofuran (**4j**) (26 mg, 0.098 mmol). Flash column chromatography on silica gel (ethyl acetate in cyclohexane) of the crude reaction mixture afforded the product **5j** as a yellow oil (18 mg, 72%).

^{1}H NMR (400 MHz, $CDCl_3$) δ 8.74 (d, *J* = 1.1 Hz, 1H), 7.80 – 7.77 (m, 1H), 7.68 (dt, *J* = 1.7, 0.7 Hz, 1H), 7.65 (dt, *J* = 8.6, 0.9 Hz, 1H), 7.59 (tdt, *J* = 7.5, 1.2, 0.6 Hz, 1H), 7.55 – 7.49 (m, 2H), 7.35 (ddt, *J* = 7.5, 1.4, 0.7 Hz, 1H).

^{13}C NMR (126 MHz, $CDCl_3$) δ 161.9, 146.5, 140.3 (q, *J* = 2.0 Hz), 135.8, 132.4, 131.6 (d, *J* = 1.2 Hz), 131.6 (d, *J* = 1.6 Hz), 128.9 (q, *J* = 29.8 Hz), 128.0, 126.4 (q, *J* = 5.3 Hz), 124.2 (q, *J* = 273.9 Hz), 122.3 (d, *J* = 1.7 Hz), 121.3, 109.2.

^{19}F NMR (376 MHz, $CDCl_3$) δ -56.84.

HRMS (EI) m/z: $[M]^+$ Calculated for $C_{14}H_8F_3NO^+$ 263.0552; Found 263.0548.

N*-Phenethylbenzo[*d*]isoxazole-5-carboxamide* ***(5k)***

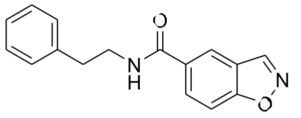

N-Phenethylbenzo[*d*]isoxazole-5-carboxamide (**5k**) was prepared according to the general procedure D using *N*-phenethylbenzofuran-5-carboxamide (**4k**) (66 mg, 0.25 mmol). Flash column chromatography on silica gel (ethyl acetate in cyclohexane) of the crude reaction mixture afforded the product **5k** as a yellow oil (45 mg, 68%). 10% starting material was recovered.

^{1}H NMR (400 MHz, CD_3CN) δ 8.92 (d, *J* = 1.0 Hz, 1H), 8.21 (dd, *J* = 1.8, 0.8 Hz, 1H), 7.99 (dd, *J* = 8.8, 1.7 Hz, 1H), 7.67 (dt, *J* = 8.8, 0.9 Hz, 1H), 7.34 – 7.25 (m, 4H), 7.21 (tq, *J* = 5.8, 1.9 Hz, 1H), 3.61 (ddd, *J* = 7.4, 6.8, 5.8 Hz, 2H), 2.91 (t, *J* = 7.2 Hz, 2H).

^{13}C NMR (101 MHz, CD_3CN) δ 167.0, 164.1, 148.2, 140.6, 132.3, 130.4, 129.8 (2C), 129.4 (2C), 127.2, 123.1, 122.6, 110.3, 42.1, 36.3.

HRMS (ESI) m/z: $[M+H]^+$ Calculated for $C_{16}H_{15}N_2O_2^+$ 267.1128; Found 267.1127.

N*-Phenethylbenzo[*d*]isoxazole-6-carboxamide* ***(5l)***

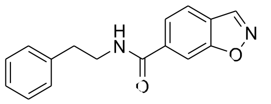

N-Phenethylbenzo[*d*]isoxazole-6-carboxamide (**5l**) was prepared according to the general procedure D using *N*-phenethylbenzofuran-6-carboxamide (**4l**) (66 mg, 0.25 mmol). Flash column chromatography on silica gel (ethyl acetate in cyclohexane) of the crude reaction mixture afforded the product **5l** as a yellow oil (36 mg, 55%). 29% starting material was recovered.

^{1}H NMR (400 MHz, CD_3CN) δ 8.92 (d, *J* = 1.1 Hz, 1H), 7.99 (q, *J* = 1.1 Hz, 1H), 7.87 (dd, *J* = 8.3, 0.8 Hz, 1H), 7.71 (dd, *J* = 8.2, 1.3 Hz, 1H), 7.34 – 7.20 (m, 5H), 3.62 (ddd, *J* = 7.4, 6.7, 5.9 Hz, 2H), 2.92 (t, *J* = 7.2 Hz, 2H).

^{13}C NMR (101 MHz, CD_3CN) δ 167.1, 162.8, 147.8, 140.6, 138.1, 129.9 (2C), 129.4 (2C), 127.2, 124.5, 123.7, 123.7, 109.2, 42.2, 36.2.

HRMS (ESI) m/z: $[M+H]^+$ Calculated for $C_{16}H_{15}N_2O_2^+$ 267.1128; Found 267.1128.

6-Vinylbenzo[*d*]isoxazole ***(5m)***

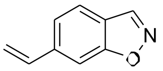

6-Vinylbenzo[*d*]isoxazole (**5m**) was prepared according to the general procedure D using 6-vinylbenzofuran (**4m**) (29 mg, 0.20 mmol). Flash column chromatography on silica gel (ethyl acetate in cyclohexane) of the crude reaction mixture afforded the product **5m** as a yellow oil (7 mg, 24%).

^{1}H NMR (500 MHz, $CDCl_3$) δ 8.67 (d, *J* = 1.1 Hz, 1H), 7.67 (dt, *J* = 8.3, 0.5 Hz, 1H), 7.62 – 7.57 (m, 1H), 7.42 (ddd, *J* = 8.3, 1.4, 0.5 Hz, 1H), 6.85 (ddd, *J* = 17.6, 10.9, 0.5 Hz, 1H), 5.91 (dd, *J* = 17.5, 0.6 Hz, 1H), 5.43 (dd, *J* = 10.9, 0.6 Hz, 1H).

^{13}C NMR (126 MHz, $CDCl_3$) δ 163.10, 146.17, 140.21, 136.30, 122.48, 121.89, 120.95, 116.89, 107.28.

HRMS (ESI) m/z: $[M+H]^+$ Calculated for $C_9H_8NO^+$ 146.0600; Found 146.0601.

Benzoxazoles from 3-Substituted Benzofurans

2-*Methylbenzo*[d]*oxazole* ***(2a)***

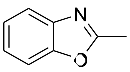

2-Methylbenzo[*d*]oxazole (**2a**) was prepared according to the general procedure E using 3-methylbenzofuran (**1a**) (33.0 mg, 31.6 µL, 0.25 mmol). Flash column chromatography on silica gel (ethyl acetate in cyclohexane) of the crude reaction mixture afforded the product **2a** as a yellow oil (16 mg, 48%).

^{1}H NMR (400 MHz, $CDCl_3$) δ 7.68 – 7.62 (m, 1H), 7.49 – 7.44 (m, 1H), 7.33 – 7.26 (m, 2H), 2.64 (s, 3H).

^{13}C NMR (101 MHz, $CDCl_3$) δ 164.0, 151.1, 141.7, 124.6, 124.2, 119.6, 110.3, 14.7.

HRMS (ESI) m/z: $[M+H]^+$ Calculated for $C_8H_8NO^+$ 134.0606; Found 134.0602.

The spectral data are consistent with those reported in the literature. [47]

2-Phenylbenzo[d]*oxazole* ***(2b)***

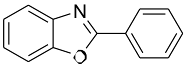

2-Phenylbenzo[*d*]oxazole (**2b**) was prepared according to the general procedure E using 3-phenylbenzofuran (**1b**) (49 mg, 0.25 mmol). Flash column chromatography on silica gel (ethyl acetate in cyclohexane) of the crude reaction mixture afforded the product **2b** as yellow solid (11 mg, 23%).

^{1}H NMR (400 MHz, $CDCl_3$) δ 8.30 – 8.24 (m, 2H), 7.83 – 7.74 (m, 1H), 7.62 – 7.56 (m, 1H), 7.56 – 7.50 (m, 3H), 7.40 – 7.33 (m, 2H).

^{13}C NMR (101 MHz, $CDCl_3$) δ 163.2, 150.9, 142.3, 131.7, 129.1 (2C), 127.8 (2C), 127.3, 125.3, 124.7, 120.2, 110.8.

HRMS (ESI) m/z: $[M+H]^+$ Calculated for $C_{13}H_{10}NO^+$ 196.0757; Found 196.0756.

The spectral data are consistent with those reported in the literature. [48]

6-Bromo-2-methylbenzo[d]oxazole ***(2c)***

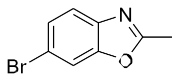

6-Bromo-2-methylbenzo[*d*]oxazole (**2c**) was prepared according to the general procedure E using 6-bromo-2-methylbenzo[*d*]oxazole (**1c**) (53 mg, 0.25 mmol). Flash column chromatography on silica gel (ethyl acetate in cyclohexane) of the crude reaction mixture afforded the product **2c** as a colorless solid (30 mg, 57%).

^{1}H NMR (400 MHz, $CDCl_3$) δ 7.64 (dd, J = 1.8, 0.5 Hz, 1H), 7.51 (dd, J = 8.4, 0.5 Hz, 1H), 7.42 (dd, J = 8.4, 1.8 Hz, 1H), 2.63 (s, 3H).

^{13}C NMR (101 MHz, $CDCl_3$) δ 164.9, 151.9, 141.2, 128.0, 120.9, 117.9, 114.3, 15.0.

HRMS (ESI) m/z: $[M+H]^+$ Calculated for $C_8H_7BrNO^+$ 211.9706; Found 211.9705.

The spectral data are consistent with those reported in the literature. [49]

6-Methoxy-2-methylbenzo[d]oxazole ***(2d)***

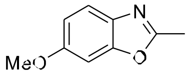

6-Methoxy-2-methylbenzo[*d*]oxazole (**2d**) was prepared according to the general procedure E using 6-methoxy-2-methylbenzofuran (**1d**) (41 mg, 0.25 mmol). Flash column chromatography on silica gel (ethyl acetate in cyclohexane) of the crude reaction mixture afforded the product **2d** as a yellow oil (14 mg, 34%).

^{1}H NMR (400 MHz, $CDCl_3$) δ 7.50 (d, *J* = 8.7 Hz, 1H), 7.00 (d, *J* = 2.4 Hz, 1H), 6.89 (dd, *J* = 8.7, 2.4 Hz, 1H), 3.84 (s, 3H), 2.59 (s, 3H).

^{13}C NMR (101 MHz, $CDCl_3$) δ 162.6, 157.5, 151.6, 134.9, 119.1, 111.8, 95.2, 55.7, 14.3.

HRMS (EI) m/z: $[M]^+$ Calculated for $C_9H_9NO_2^+$ 163.0628; Found 163.0627.

The spectral data are consistent with those reported in the literature. [46]

Methyl 2-methylbenzo[d]oxazole-5-carboxylate ***(2e)***

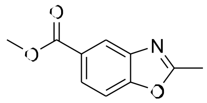

Methyl 3-methylbenzo[*d*]isoxazole-5-carboxylate (**2e**) was prepared according to the general procedure using methyl 3-methylbenzofuran-5-carboxylate (**1e**) (19 mg, 0.10 mmol). Flash column chromatography on silica gel (ethyl acetate in cyclohexane) of the crude reaction mixture afforded the product **2e** as a colorless solid (11 mg, 55%).

^{1}H NMR (400 MHz, $CDCl_3$) δ 8.34 (dd, J = 1.7, 0.6 Hz, 1H), 8.06 (dd, J = 8.5, 1.7 Hz, 1H), 7.50 (dd, J = 8.5, 0.6 Hz, 1H), 3.95 (s, 3H), 2.67 (s, 3H).

^{13}C NMR (101 MHz, $CDCl_3$) δ 166.9, 165.4, 154.1, 141.7, 126.8, 126.7, 121.6, 110.2, 52.4, 14.7.

HRMS (ESI) m/z: $[M+H]^+$ Calculated for $C_{10}H_{10}NO_3^+$ 192.0655; Found 192.0658.

The spectral data are consistent with those reported in the literature. [50]

Methyl 2-methylbenzo[d]oxazole-6-carboxylate ***(2f)***

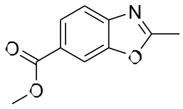

Methyl 3-methylbenzo[*d*]isoxazole-6-carboxylate (**2f**) was prepared according to the general procedure using methyl 3-methylbenzofuran-6-carboxylate (**1f**) (19 mg, 0.10 mmol). Flash column chromatography on silica gel (ethyl acetate in cyclohexane) of the crude reaction mixture afforded the product **2f** as a colorless solid (9 mg, 44%).

¹H NMR (400 MHz, $CDCl_3$) δ 8.16 (dd, *J* = 1.6, 0.6 Hz, 1H), 8.04 (dd, *J* = 8.3, 1.5 Hz, 1H), 7.67 (dd, *J* = 8.3, 0.6 Hz, 1H), 3.95 (s, 3H), 2.68 (s, 3H).

¹³C NMR (101 MHz, $CDCl_3$) δ 166.9, 166.9, 150.8, 145.6, 126.8, 126.1, 119.2, 112.1, 52.5, 14.9.

HRMS (ESI) m/z: $[M+H]^+$ Calculated for $C_{10}H_{10}NO_3^+$ 192.0655; Found 192.0654.

The spectral data are consistent with those reported in the literature. [51]

*2-Methyl-5-phenylbenzo[*d*]oxazole (**2g**)*

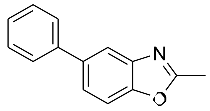

2-Methyl-5-phenylbenzo[*d*]oxazole (**2g**) was prepared according to the general procedure E using 3-methyl-5-phenylbenzofuran (**1g**) (43 mg, 0.25 mmol). Flash column chromatography on silica gel (ethyl acetate in cyclohexane) of the crude reaction mixture afforded the product **2g** as yellow oil (22 mg, 48%).

¹H NMR (400 MHz, $CDCl_3$) δ 7.84 (t, *J* = 1.2 Hz, 1H), 7.63 – 7.59 (m, 2H), 7.52 (d, *J* = 1.2 Hz, 2H), 7.49 – 7.43 (m, 2H), 7.39 – 7.33 (m, 1H), 2.67 (s, 3H).

¹³C NMR (101 MHz, $CDCl_3$) δ 164.7, 150.7, 142.1, 141.2, 138.3, 129.0 (2C), 127.6 (2C), 127.4, 124.3, 118.1, 110.4, 14.8.

HRMS (ESI) m/z: $[M+H]^+$ Calculated for $C_{14}H_{12}NO^+$ 210.0913; Found 210.0908.

The spectral data are consistent with those reported in the literature. [52]

*2-Methyl-6-phenylbenzo[*d*]oxazole (**2h**)*

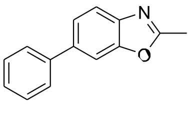

2-Methyl-6-phenylbenzo[*d*]oxazole (**2h**) was prepared according to the general procedure using 3-methyl-6-phenylbenzofuran (**1h**) (52 mg, 0.25 mmol). Flash column chromatography on silica gel (ethyl acetate in cyclohexane) of the crude reaction mixture afforded the product **2h** as orange solid (30 mg, 57%).

¹H NMR (400 MHz, $CDCl_3$) δ 7.71 (dd, J = 8.2, 0.6 Hz, 1H), 7.69 (dd, J = 1.6, 0.6 Hz, 1H), 7.63 – 7.60 (m, 2H), 7.55 (dd, J = 8.2, 1.7 Hz, 1H), 7.47 (m, 2H), 7.39 – 7.35 (m, 1H), 2.69 (s, 3H).

¹³C NMR (101 MHz, $CDCl_3$) δ 164.6, 151.7, 141.0, 138.8, 129.1 (2C), 127.6 (2C), 124.0, 119.4, 109.0, 14.7. *(two carbons are obstructed)*

HRMS (EI) m/z: $[M]^+$ Calculated for $C_{14}H_{11}NO^+$ 209.0835; Found 209.0833.

The spectral data are consistent with those reported in the literature. [53]

*6-(3-Chlorophenyl)-2-methylbenzo[*d*]oxazole (**2i**)*

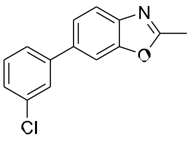

6-(3-Chlorophenyl)-2-methylbenzo[*d*]oxazole (**2i**) was prepared according to the general procedure E using 6-(3-chlorophenyl)-3-methylbenzofuran (**1i**) (61 mg, 0.25 mmol). Flash column chromatography on silica gel (ethyl acetate in cyclohexane) of the crude reaction mixture afforded the product **2i** as a colorless solid (31 mg, 51%).

¹H NMR (400 MHz, $CDCl_3$) δ 7.69 (dd, J = 8.3, 0.6 Hz, 1H), 7.64 (dd, J = 1.7, 0.6 Hz, 1H), 7.59 (td, J = 1.9, 0.5 Hz, 1H), 7.52 – 7.46 (m, 2H), 7.38 (td, J = 7.7, 0.5 Hz, 1H), 7.33 (ddd, J = 7.9, 2.0, 1.3 Hz, 1H), 2.67 (s, 3H).

¹³C NMR (101 MHz, $CDCl_3$) δ 164.8, 151.7, 142.9, 141.5, 137.1, 134.9, 130.2, 127.7, 127.5, 125.7, 123.8, 119.7, 109.0, 14.8.

HRMS (ESI) m/z: $[M+H]^+$ Calculated for $C_{14}H_{11}ClNO^+$ 244.0524; Found 244.0522.

6-(4-Chlorophenyl)-2-methylbenzo[d]oxazole ***(2j)***

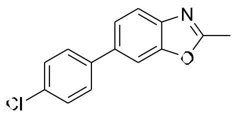

6-(4-Chlorophenyl)-2-methylbenzo[*d*]oxazole (**2j**) was prepared according to the general procedure E using 6-(4-chlorophenyl)-2-methylbenzofuran (**1j**) (24 mg, 0.10 mmol). Flash column chromatography on silica gel (ethyl acetate in cyclohexane) of the crude reaction mixture afforded the product **2j** as a colorless solid (15 mg, 62%).

^{1}H NMR (400 MHz, $CDCl_3$) δ 7.69 (dd, *J* = 8.3, 0.6 Hz, 1H), 7.63 (dd, *J* = 1.7, 0.6 Hz, 1H), 7.58 – 7.51 (m, 2H), 7.49 (dd, *J* = 8.2, 1.7 Hz, 1H), 7.45 – 7.39 (m, 2H), 2.66 (s, 3H).

^{13}C NMR (101 MHz, $CDCl_3$) δ 164.7, 151.8, 141.3, 139.5, 137.3, 133.7, 129.2 (2C), 128.8 (2C), 123.7, 119.7, 108.8, 14.8.

HRMS (ESI) m/z: $[M+H]^+$ Calculated for $C_{14}H_{11}ClNO^+$ 244.0524; Found 244.0523.

*2-Methyl-6-(2-(trifluoromethyl)phenyl)benzo[*d*]oxazole* ***(2k)***

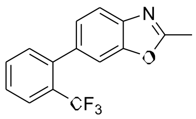

2-Methyl-6-(2-(trifluoromethyl)phenyl)benzo[*d*]oxazole (**2k**) was prepared according to the general procedure E using 2-methyl-6-(2-(trifluoromethyl)phenyl)benzofuran (**1k**) (69 mg, 0.25 mmol). Flash column chromatography on silica gel (ethyl acetate in cyclohexane) of the crude reaction mixture afforded the product **2k** as a a yellow oil (29 mg, 41%).

^{1}H NMR (400 MHz, $CDCl_3$) δ 7.76 (ddt, *J* = 7.9, 1.2, 0.6 Hz, 1H), 7.66 (dd, *J* = 8.1, 0.6 Hz, 1H), 7.57 (tdq, *J* = 7.6, 1.3, 0.6 Hz, 1H), 7.49 (dddt, *J* = 8.5, 6.7, 1.4, 0.9 Hz, 1H), 7.45 (dt, *J* = 1.6, 0.6 Hz, 1H), 7.36 (ddt, *J* = 7.6, 1.4, 0.7 Hz, 1H), 7.26 (ddd, *J* = 8.2, 1.6, 0.6 Hz, 1H), 2.67 (s, 3H).

^{13}C NMR (101 MHz, $CDCl_3$) δ 164.7, 150.6, 141.1, 141.0 (q, *J* = 1.9 Hz), 136.6, 132.4, 131.5 (d, *J* = 1.2 Hz), 128.8 (q, *J* = 29.7 Hz), 127.7, 126.3 (q, *J* = 5.3 Hz), 125.6, 124.3 (q, *J* = 275.9 Hz), 118.6, 111.1 (q, *J* = 1.8 Hz), 14.7.

^{19}F NMR (376 MHz, $CDCl_3$) δ -56.78.

HRMS (ESI) m/z: $[M+H]^+$ Calculated for $C_{15}H_{11}F_3NO^+$ 278.0787; Found 278.0786.

*2-Methyl-6-(phenylethynyl)benzo[*d*]oxazole* ***(2l)***

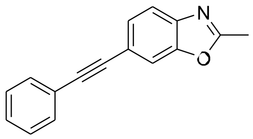

2-Methyl-6-(phenylethynyl)benzo[*d*]oxazole (**2l**) was prepared according to the general procedure E using 2-methyl-6-(phenylethynyl)benzofuran (**1l**) (23 mg, 0.10 mmol). Flash column chromatography on silica gel (0–20% ethyl acetate in cyclohexane) of the crude reaction mixture afforded the product **2l** as a yellow solid (11 mg, 47%).

^{1}H NMR (400 MHz, $CDCl_3$) δ 7.65 (dd, *J* = 1.4, 0.7 Hz, 1H), 7.61 (dd, *J* = 8.2, 0.6 Hz, 1H), 7.56 – 7.54 (m, 2H), 7.49 (dd, *J* = 8.2, 1.4 Hz, 1H), 7.39 – 7.33 (m, 3H), 2.66 (s, 3H).

^{13}C NMR (101 MHz, $CDCl_3$) δ 165.2, 150.8, 141.7, 131.8 (2C), 128.5 (2C), 128.5, 128.4, 123.2, 119.8, 119.4, 113.5, 89.5, 89.3, 14.7.

HRMS (EI) m/z: $[M]^+$ Calculated for $C_{16}H_{11}NO^+$ 233.0835; Found 233.0832.

Benzisoxazoles from 3-Substituted Benzofurans

*3-Methylbenzo[d]isoxazole (**3a**)*

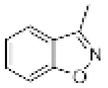

3-Methylbenzo[*d*]isoxazole (**3a**) was prepared according to the general procedure F using 3-methylbenzofuran (**1a**) (33 mg, 0.25 mmol). Flash column chromatography on silica gel (ethyl acetate in cyclohexane) of the crude reaction mixture afforded the product **3a** as yellow oil (17 mg, 51%).

^{1}H NMR (400 MHz, $CDCl_3$) δ 7.64 (dt, *J* = 7.9, 1.0 Hz, 1H), 7.55 (d, *J* = 1.0 Hz, 1H), 7.54 (dd, *J* = 2.0, 1.0 Hz, 1H), 7.31 (ddd, *J* = 7.9, 5.0, 3.0 Hz, 1H), 2.59 (s, 3H).

^{13}C NMR (101 MHz, $CDCl_3$) δ 162.9, 155.1, 129.9, 123.3, 122.4, 121.3, 109.9, 10.2.

HRMS (ESI) m/z: $[M+H]^+$ Calculated for $C_8H_8NO^+$ 134.0600; Found 134.0600.

The spectral data are consistent with those reported in the literature. [54]

*3-Phenylbenzo[d]isoxazole (**3b**)*

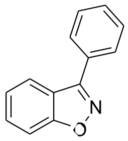

3-Phenylbenzo[*d*]isoxazole (**3b**) was prepared according to the general procedure F using 3-phenylbenzofuran (**1b**) (49 mg, 0.25 mmol). Flash column chromatography on silica gel (ethyl acetate in cyclohexane) of the crude reaction mixture afforded the product **3b** as yellow oil (30 mg, 43%).

^{1}H NMR (400 MHz, $CDCl_3$) δ 8.04 – 7.96 (m, 2H), 7.94 (dt, *J* = 8.0, 1.0 Hz, 1H), 7.70 – 7.62 (m, 1H), 7.62 – 7.54 (m, 4H), 7.39 (ddd, *J* = 8.0, 6.9, 1.0 Hz, 1H).

^{13}C NMR (101 MHz, $CDCl_3$) δ 164.0, 157.5, 130.4, 129.9, 129.3 (2C), 129.1, 128.3 (2C), 124.0, 122.4, 120.7, 110.4.

HRMS (ESI) m/z: $[M+H]^+$ Calculated for $C_{13}H_{10}NO^+$ 196.0757; Found 196.0756.

The spectral data are consistent with those reported in the literature. [55]

*6-Bromo-3-methylbenzo[d]isoxazole (**3c**)*

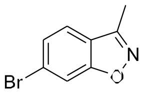

6-Bromo-3-methylbenzo[*d*]isoxazole (**3c**) was prepared according to the general procedure F using 6-bromo-3-methylbenzofuran (**1c**) (53 mg, 0.25 mmol). Flash column chromatography on silica gel (ethyl acetate in cyclohexane) of the crude reaction mixture afforded the product **3c** as colorless solid (25 mg, 47%).

^{1}H NMR (400 MHz, $CDCl_3$) δ 7.74 (dd, *J* = 1.5, 0.6 Hz, 1H), 7.49 (dd, *J* = 8.3, 0.6 Hz, 1H), 7.43 (dd, *J* = 8.4, 1.5 Hz, 1H), 2.57 (s, 3H).

^{13}C NMR (101 MHz, $CDCl_3$) δ 163.8, 155.4, 127.4, 124.9, 122.5, 121.9, 113.8, 10.4.

HRMS (EI) m/z: $[M]^+$ Calculated for $C_8H_6BrNO^+$ 210.9627; Found 210.9625.

The spectral data are consistent with those reported in the literature. [56]

*6-Methoxy-3-methylbenzo[d]isoxazole (**3d**)*

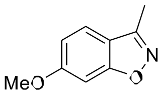

6-Methoxy-3-methylbenzo[*d*]isoxazole (**3d**) was prepared according to the general procedure F using 6-methoxy-3-methylbenzofuran (**1d**) (16 mg, 0.10 mmol). Flash column chromatography on silica gel (ethyl acetate in cyclohexane) of the crude reaction mixture afforded the product **3d** as colorless solid (6 mg, 29%) in 79% purity with 1-(2-hydroxy-4-methoxyphenyl)ethan-1-one as impurity.

^{1}**H NMR** (400 MHz, $CDCl_3$) δ 7.46 (dd, *J* = 8.7, 0.6 Hz, 1H), 6.97 (dd, *J* = 2.1, 0.5 Hz, 1H), 6.91 (dd, *J* = 8.7, 2.1 Hz, 1H), 3.88 (s, 3H), 2.53 (s, 3H).

^{13}**C NMR** (101 MHz, $CDCl_3$) δ 164.7, 162.2, 154.7, 121.4, 115.7, 113.9, 92.6, 55.8, 10.0.

HRMS (ESI) m/z: $[M+H]^+$ Calculated for $C_9H_{10}NO_2^+$ 164.0706; Found 164.0707.

*Methyl 3-methylbenzo[d]isoxazole-5-carboxylate (**3e**)*

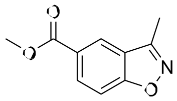

Methyl 3-methylbenzo[*d*]isoxazole-5-carboxylate (**3e**) was prepared according to the general procedure F using methyl 3-methylbenzofuran-5-carboxylate (**1e**) (19 mg, 0.10 mmol). Flash column chromatography on silica gel (ethyl acetate in cyclohexane) of the crude reaction mixture afforded the product **3e** as colorless solid (8 mg, 42%).

^{1}**H NMR** (400 MHz, $CDCl_3$) δ 8.40 (dd, *J* = 1.6, 0.8 Hz, 1H), 8.25 (dd, *J* = 8.8, 1.6 Hz, 1H), 7.58 (dd, *J* = 8.8, 0.7 Hz, 1H), 3.97 (s, 3H), 2.63 (s, 3H).

^{13}**C NMR** (126 MHz, $CDCl_3$) δ 166.5, 165.2, 155.9, 131.3, 126.0, 124.3, 122.8, 110.0, 52.6, 10.2.

HRMS (ESI) m/z: $[M+H]^+$ Calculated for $C_{10}H_{10}NO_3^+$ 192.0655; Found 192.0656.

The spectral data are consistent with those reported in the literature. [57]

*Methyl 3-methylbenzo[d]isoxazole-6-carboxylate (**3f**)*

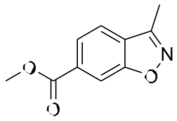

Methyl 3-methylbenzo[*d*]isoxazole-6-carboxylate (**3f**) was prepared according to the general procedure F using methyl 3-methylbenzofuran-6-carboxylate (**1f**) (19 mg, 0.10 mmol). Flash column chromatography on silica gel (ethyl acetate in cyclohexane) of the crude reaction mixture afforded the product **3f** as colorless solid (7.4 mg, 39%).

^{1}**H NMR** (400 MHz, $CDCl_3$) δ 8.23 (dd, *J* = 1.3, 0.8 Hz, 1H), 8.00 (dd, *J* = 8.2, 1.3 Hz, 1H), 7.69 (dd, *J* = 8.3, 0.8 Hz, 1H), 3.98 (s, 3H), 2.62 (s, 3H).

^{13}**C NMR** (101 MHz, $CDCl_3$) δ 166.6, 162.7, 155.2, 131.8, 125.8, 124.4, 121.2, 111.6, 52.8, 10.3.

HRMS (EI) m/z: $[M]^+$ Calculated for $C_{10}H_9NO_3^+$ 191.0577; Found 191.0574.

*3-Methyl-5-phenylbenzo[d]isoxazole (**3g**)*

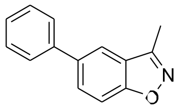

3-Methyl-5-phenylbenzo[*d*]isoxazole (**3g**) was prepared according to the general procedure F using 3-methyl-5-phenylbenzofuran (**1g**) (21 mg, 0.10 mmol). Flash column chromatography on silica gel (ethyl acetate in cyclohexane) of the crude reaction mixture afforded the product **3g** as colorless solid (14 mg, 65%).

^{1}**H NMR** (400 MHz, $CDCl_3$) δ 7.85 – 7.73 (m, 2H), 7.66 – 7.55 (m, 3H), 7.53 – 7.44 (m, 2H), 7.43 – 7.34 (m, 1H), 2.63 (s, 3H).

^{13}**C NMR** (101 MHz, $CDCl_3$) δ 162.5, 155.4, 140.7, 137.3, 129.8, 129.1 (2C), 127.5 (2C), 123.1, 119.4, 110.2, 10.3. *(one carbon is obstructed)*

HRMS (ESI) m/z: $[M+H]^+$ Calculated for $C_{14}H_{12}NO^+$ 210.0913; Found 210.0914.

*3-Methyl-6-phenylbenzo[d]isoxazole **(3h)***

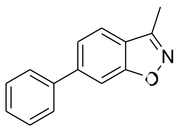

3-Methyl-6-phenylbenzo[*d*]isoxazole (**3h**) was prepared according to the general procedure F using 3-methyl-6-phenylbenzofuran (**1h**) (52 mg, 0.25 mmol). Flash column chromatography on silica gel (ethyl acetate in cyclohexane) of the crude reaction mixture afforded the product **3h** as colorless solid (29 mg, 55%).

^{1}H NMR (400 MHz, $CDCl_3$) δ 7.73 (dd, *J* = 1.4, 0.8 Hz, 1H), 7.70 – 7.61 (m, 3H), 7.55 (dd, *J* = 8.2, 1.4 Hz, 1H), 7.52 – 7.46 (m, 2H), 7.45 – 7.38 (m, 1H), 2.61 (s, 3H).

^{13}C NMR (101 MHz, $CDCl_3$) δ 164.1, 155.4, 144.0, 140.8, 129.4 (2C), 128.6, 128.1 (2C), 123.6, 121.8, 121.7, 108.5, 10.6.

HRMS (EI) m/z: $[M]^+$ Calculated for $C_{14}H_{11}NO^+$ 209.0835; Found 209.0831.

The spectral data are consistent with those reported in the literature. [58]

*6-(3-chlorophenyl)-3-methylbenzo[d]isoxazole **(3i)***

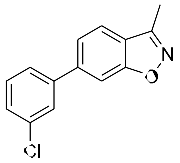

6-(3-chlorophenyl)-3-methylbenzo[*d*]isoxazole (**3i**) was prepared according to the general procedure F using 6-(3-chlorophenyl)-3-methylbenzofuran (**1i**) (24 mg, 0.10 mmol). Flash column chromatography on silica gel (ethyl acetate in cyclohexane) of the crude reaction mixture afforded the product **3i** as colorless solid (14 mg, 56%).

^{1}H NMR (500 MHz, $CDCl_3$) δ 7.70 (dd, *J* = 1.4, 0.8 Hz, 1H), 7.69 (dd, *J* = 8.2, 0.7 Hz, 1H), 7.63 (td, *J* = 1.9, 0.6 Hz, 1H), 7.58 – 7.49 (m, 2H), 7.46 – 7.37 (m, 2H), 2.62 (s, 3H).

^{13}C NMR (126 MHz, $CDCl_3$) δ 163.6, 155.1, 142.3, 142.2, 135.1, 130.4, 128.3, 127.9, 126.0, 123.2, 122.0, 121.6, 108.3, 10.3.

HRMS (EI) m/z: $[M]^+$ Calculated for $C_{14}H_{10}ClNO^+$ 243.0445; Found 243.0443.

*6-(4-Chlorophenyl)-3-methylbenzo[d]isoxazole **(3j)***

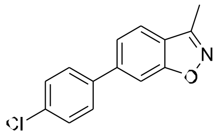

6-(4-Chlorophenyl)-3-methylbenzo[*d*]isoxazole (**3j**) was prepared according to the general procedure F using 6-(4-chlorophenyl)-3-methylbenzofuran (**1j**) (24 mg, 0.10 mmol). Flash column chromatography on silica gel (ethyl acetate in cyclohexane) of the crude reaction mixture afforded the product **3j** as colorless solid (13 mg, 53%).

^{1}H NMR (500 MHz, $CDCl_3$) δ 7.72 – 7.65 (m, 2H), 7.61 – 7.54 (m, 2H), 7.50 (dd, *J* = 8.1, 1.5 Hz, 1H), 7.48 – 7.43 (m, 2H), 2.61 (s, 3H).

^{13}C NMR (126 MHz, $CDCl_3$) δ 163.5, 155.0, 142.3, 138.8, 134.4, 131.0, 129.2, 128.9, 128.6, 122.9, 121.6, 121.4, 108.0, 10.2.

HRMS (EI) m/z: $[M]^+$ Calculated for $C_{14}H_{10}ClNO^+$ 243.0445; Found 243.0443.

The spectral data are consistent with those reported in the literature. [58]

*3-Methyl-6-(2-(trifluoromethyl)phenyl)benzo[d]isoxazole **(3k)***

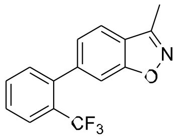

3-Methyl-6-(2-(trifluoromethyl)phenyl)benzo[*d*]isoxazole (**3k**) was prepared according to the general procedure F using 3-methyl-6-(2-(trifluoromethyl)phenyl)benzofuran (**1k**) (28 mg, 0.10 mmol). Flash column chromatography on silica gel (ethyl acetate in cyclohexane) of the crude reaction mixture afforded the product **3k** as yellow oil (15 mg, 54%).

^{1}**H NMR** (500 MHz, $CDCl_3$) δ 7.82 – 7.76 (m, 1H), 7.65 (dd, *J* = 8.1, 0.8 Hz, 1H), 7.62 – 7.58 (m, 1H), 7.56 – 7.53 (m, 1H), 7.52-7.50 (m, 1H), 7.37 – 7.35 (m, 1H), 7.29 – 7.27 (m, 1H), 2.63 (s, 3H).

^{13}**C NMR** (126 MHz, $CDCl_3$) δ 162.6, 155.1, 141.9, 140.3 (q, *J* = 2.1 Hz), 133.0, 131.6 (d, *J* = 1.2 Hz), 128.7 (q, *J* = 30.1 Hz), 128.1, 126.4 (q, *J* = 5.3 Hz), 125.0 (q, *J* = 1.7 Hz), 124.1 (q, *J* = 274.0 Hz), 121.8, 120.4, 110.5 (q, *J* = 1.6 Hz), 10.3.

^{19}**F NMR** (471 MHz, $CDCl_3$) δ -56.82.

HRMS (EI) m/z: $[M]^+$ Calculated for $C_{15}H_{10}F_3NO^+$ 277.0709; Found 277.0705.

*3-Methyl-6-(phenylethynyl)benzo[d]isoxazole (**3l**)*

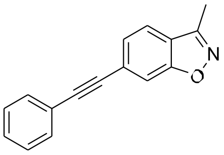

3-Methyl-6-(phenylethynyl)benzo[*d*]isoxazole (**3l**) was prepared according to the general procedure F using 3-methyl-6-(phenylethynyl)benzofuran (**1l**) (23 mg, 0.10 mmol). Flash column chromatography on silica gel (ethyl acetate in cyclohexane) of the crude reaction mixture afforded the product **3l** as yellow oil (20 mg, 43%).

^{1}**H NMR** (500 MHz, $CDCl_3$) δ 7.71 (t, *J* = 1.0 Hz, 1H), 7.60 (dd, *J* = 8.2, 0.8 Hz, 1H), 7.58 – 7.55 (m, 2H), 7.47 (dd, *J* = 8.1, 1.2 Hz, 1H), 7.41 – 7.34 (m, 3H), 2.59 (s, 3H).

^{13}**C NMR** (126 MHz, $CDCl_3$) δ 162.7, 155.1, 131.9 (2C), 128.9, 128.6 (2C), 127.1, 125.2, 122.8, 122.2, 121.1, 112.9, 91.6, 88.8, 10.2.

HRMS (EI) m/z: $[M]^+$ Calculated for $C_{16}H_{11}NO^+$ 233.0835; Found 233.0832.

*3-Methyl-5-(pyridine-3-yl)benzo[d]isoxazole (**3m**)*

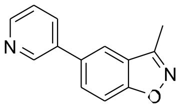

3-Methyl-5-(pyridine-3-yl)benzo[*d*]isoxazole (**3m**) was prepared according to the general procedure F using 3-(3-methylbenzofuran-5-yl)pyridine (**1m**) (21 mg, 0.10 mmol). Flash column chromatography on silica gel (ethyl acetate in cyclohexane) of the crude reaction mixture afforded the product **3m** as colorless solid (11 mg, 52%).

^{1}**H NMR** (400 MHz, $CDCl_3$) δ 8.97 – 8.76 (m, 1H), 8.74 – 8.58 (m, 1H), 7.94 (ddd, *J* = 7.9, 2.4, 1.6 Hz, 1H), 7.80 (dd, *J* = 1.8, 0.8 Hz, 1H), 7.76 (dd, *J* = 8.6, 1.8 Hz, 1H), 7.66 (dd, *J* = 8.6, 0.8 Hz, 1H), 7.44 (ddd, *J* = 7.8, 4.9, 0.8 Hz, 1H), 2.64 (s, 3H).

^{13}**C NMR** (101 MHz, $CDCl_3$) δ 162.9, 155.4, 148.3, 148.1, 136.4, 135.2, 133.6, 129.6, 124.0, 123.4, 119.9, 110.8, 10.3.

HRMS (ESI) m/z: $[M+H]^+$ Calculated for $C_{13}H_{11}N_2O^+$ 211.0866; Found 211.0865.

*3-Methyl-6-(pyridine-3-yl)benzo[d]isoxazole (**3n**)*

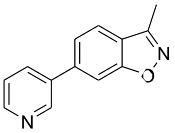

3-Methyl-6-(pyridine-3-yl)benzo[*d*]isoxazole (**3n**) was prepared according to the general procedure F using 3-(3-methylbenzofuran-6-yl)pyridine (**1n**) (21 mg, 0.10 mmol). Flash column chromatography on silica gel (ethyl acetate in cyclohexane) of the crude reaction mixture afforded the product **3n** as colorless solid (10 mg, 48%).

^{1}**H NMR** (500 MHz, $CDCl_3$) δ 8.91 (d, *J* = 2.1 Hz, 1H), 8.66 (dd, *J* = 4.9, 1.6 Hz, 1H), 7.96 (ddd, *J* = 7.9, 2.4, 1.6 Hz, 1H), 7.84 – 7.68 (m, 2H), 7.53 (dd, *J* = 8.3, 1.2 Hz, 1H), 7.44 (ddd, *J* = 8.0, 4.9, 0.8 Hz, 1H), 2.62 (s, 3H).

^{13}**C NMR** (126 MHz, $CDCl_3$) δ 163.6, 155.1, 149.0, 148.4, 140.0, 136.2, 135.3, 124.0, 123.1, 122.3, 122.0, 108.4, 10.3.

HRMS (ESI) m/z: $[M+H]^+$ Calculated for $C_{13}H_{11}N_2O^+$ 211.0866; Found 211.0861.

*3-Methyl-5-vinylbenzo[d]isoxazole (**3o**)*

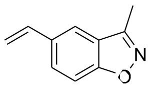

3-Methyl-5-vinylbenzo[*d*]isoxazole (**3o**) was prepared according to the general procedure F using 3-methyl-5-vinylbenzofuran (**1o**) (16 mg, 0.10 mmol). Flash column chromatography on silica gel (ethyl acetate in cyclohexane) of the crude reaction mixture afforded the product **3o** as colorless oil (6 mg, 38%).

^{1}H NMR (500 MHz, $CDCl_3$) δ 7.65 (ddd, *J* = 8.7, 1.7, 0.5 Hz, 1H), 7.63 – 7.57 (m, 1H), 7.50 (dt, *J* = 8.6, 0.6 Hz, 1H), 6.82 (ddd, *J* = 17.6, 10.9, 0.5 Hz, 1H), 5.77 (dd, *J* = 17.5, 0.6 Hz, 1H), 5.29 (dd, *J* = 10.9, 0.6 Hz, 1H), 2.59 (s, 3H).

^{13}C NMR (126 MHz, $CDCl_3$) δ 162.79, 155.31, 136.09, 133.60, 128.22, 122.86, 118.82, 114.10, 110.04, 10.24.

HRMS (EI) m/z: $[M]^+$ Calculated for $C_{10}H_9NO^+$ 159.0679; Found 159.0678.

*3-Methyl-5-vinylbenzo[d]isoxazole (**3p**)*

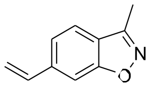

3-Methyl-6-vinylbenzo[*d*]isoxazole (**3p**) was prepared according to the general procedure F using 3-methyl-6-vinylbenzofuran (**1p**) (16 mg, 0.10 mmol). Flash column chromatography on silica gel (ethyl acetate in cyclohexane) of the crude reaction mixture afforded the product **3p** as colorless oil (5 mg, 31%).

^{1}H NMR (500 MHz, $CDCl_3$) δ 7.61 – 7.54 (m, 1H), 7.52 (dt, *J* = 1.3, 0.7 Hz, 1H), 7.39 (ddd, *J* = 8.2, 1.3, 0.5 Hz, 1H), 6.84 (ddd, *J* = 17.5, 10.9, 0.5 Hz, 1H), 5.89 (dd, *J* = 17.5, 0.6 Hz, 1H), 5.41 (dd, *J* = 10.9, 0.6 Hz, 1H), 2.57 (s, 3H).

^{13}C NMR (126 MHz, $CDCl_3$) δ 163.66, 155.02, 139.88, 136.39, 121.94, 121.91, 121.11, 116.58, 107.35, 10.24.

HRMS (ESI) m/z: $[M+H]^+$ Calculated for $C_{10}H_{10}NO^+$ 160.0757; Found 160.0757.

Failed Substrates

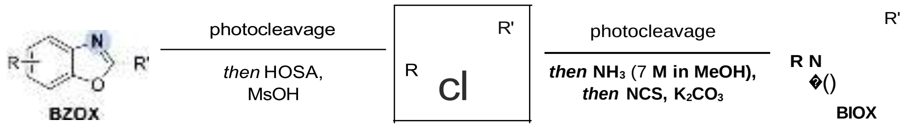

Photocleavage of benzofuran was successful

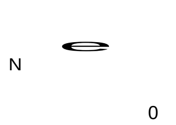

1m

BZOX = no conversion

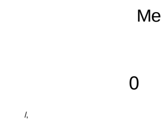

1n

BZOX = no conversion

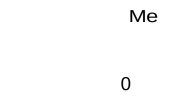

1o

BZOX = no conversion

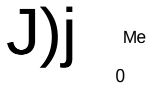

1p

BZOX = no conversion

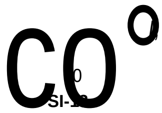

SI-13

BZOX = no conversion
BIOX = no conversion

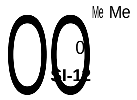

SI-12

BZOX = no conversion
BIOX = no conversion

Photocleavage of benzofuran failed

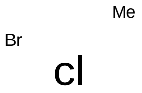

SI-14

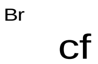

SI-15

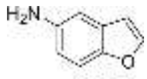

SI-16

NMR Spectra of Starting Materials

NMR Spectra of Alkylated Iodophenols

1- *Iodo-2-((3-methylbut-2-en-1-yl)oxy)benzene (**SI-7**)*

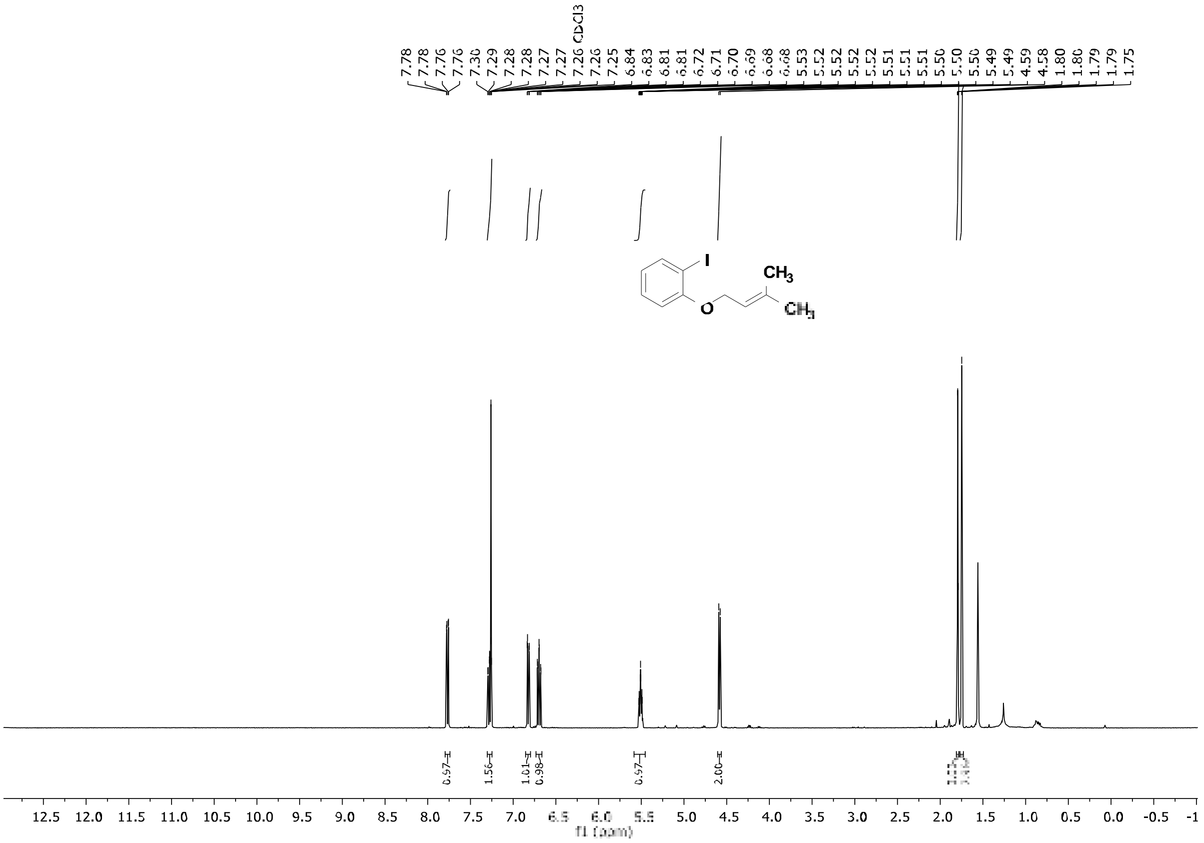

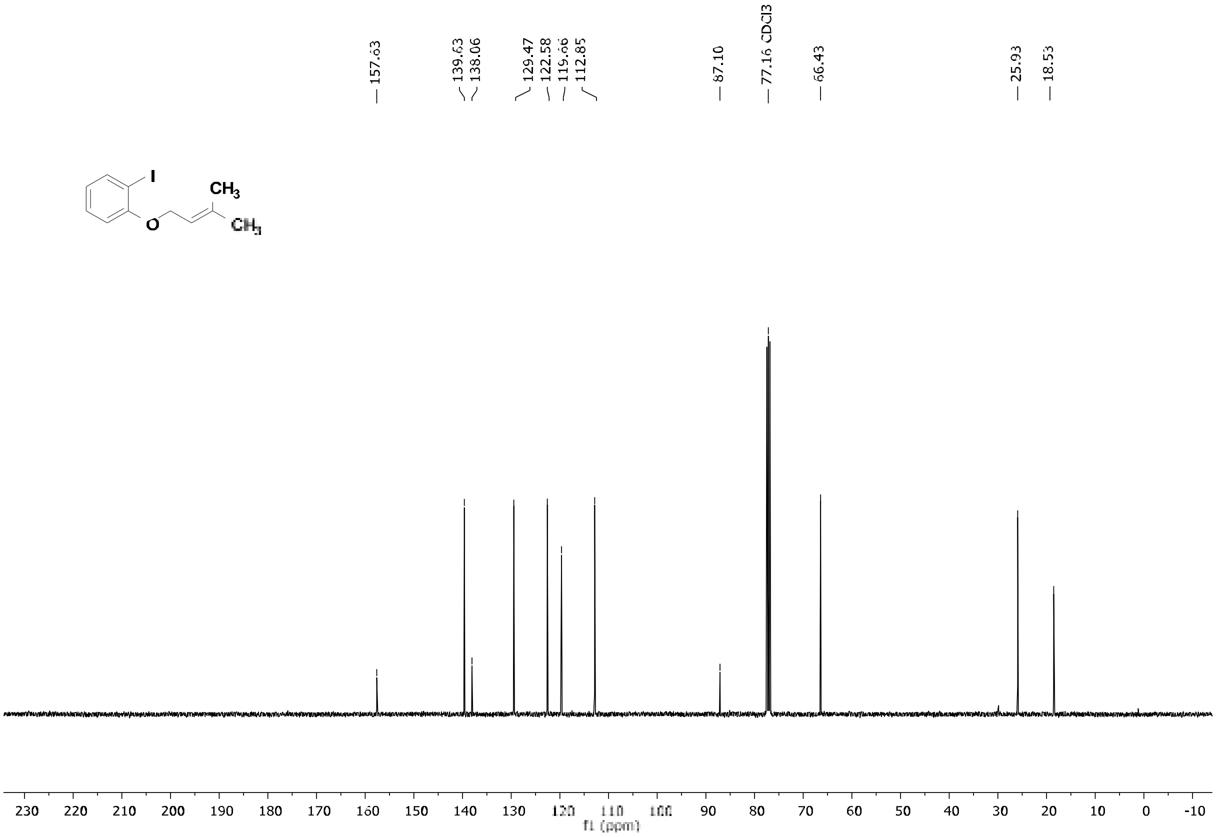

1-(Cinnamyloxy)-2-iodobenzene (**SI-8**)

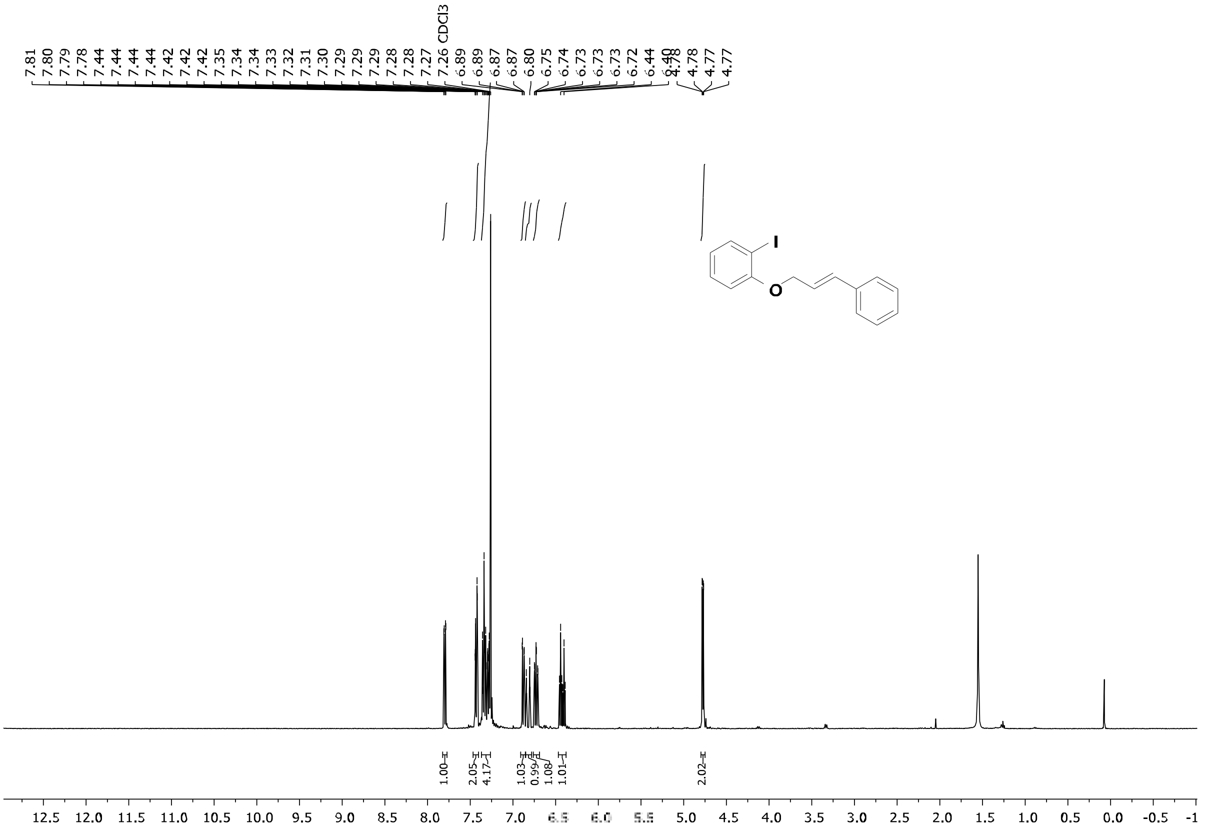

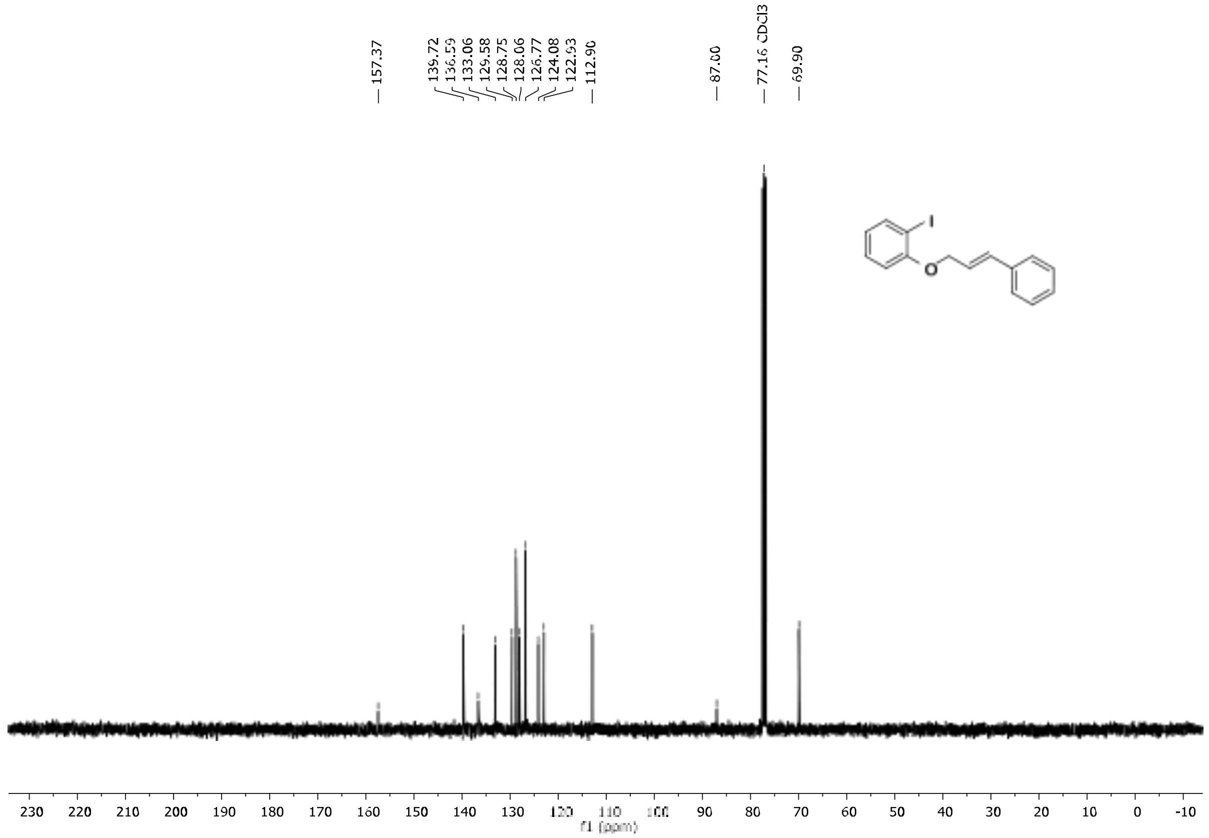

*Methyl 4-(allyloxy)-3-iodobenzoate (**SI-9**)*

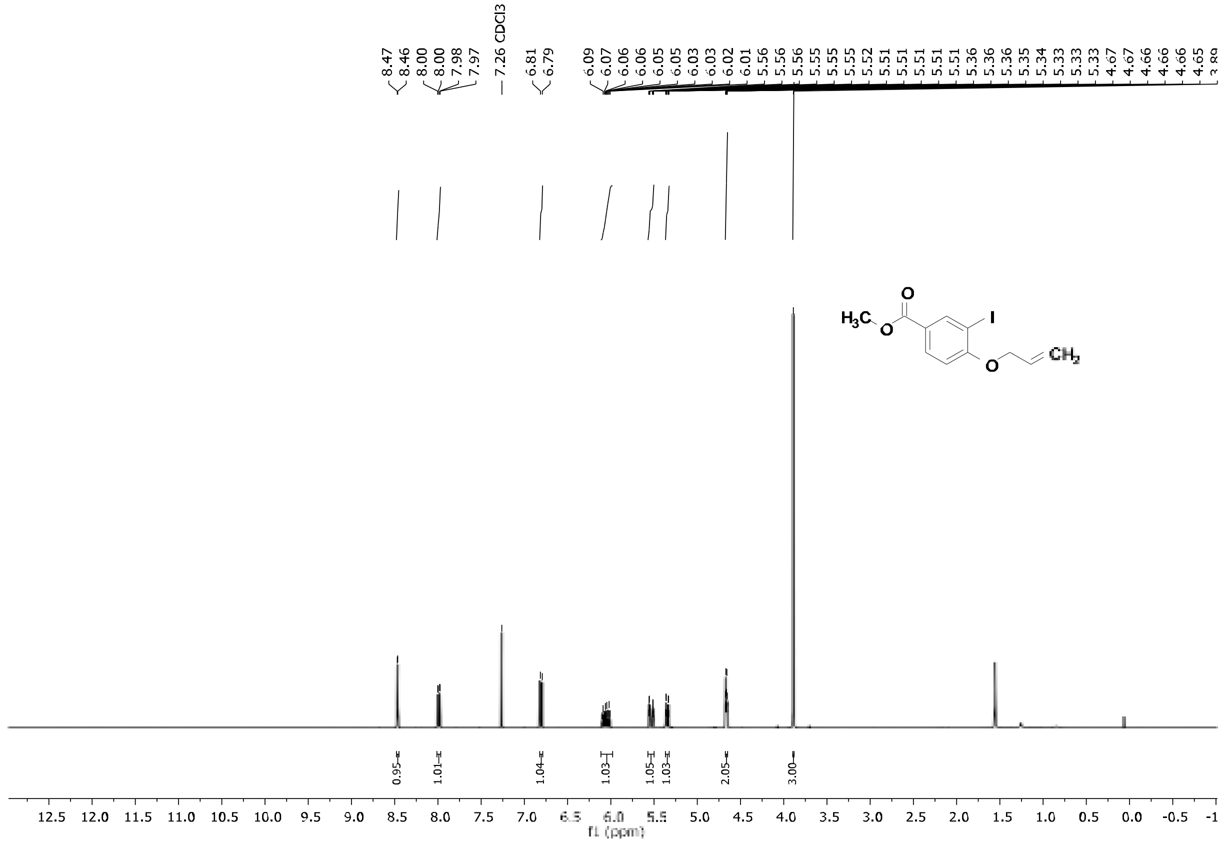

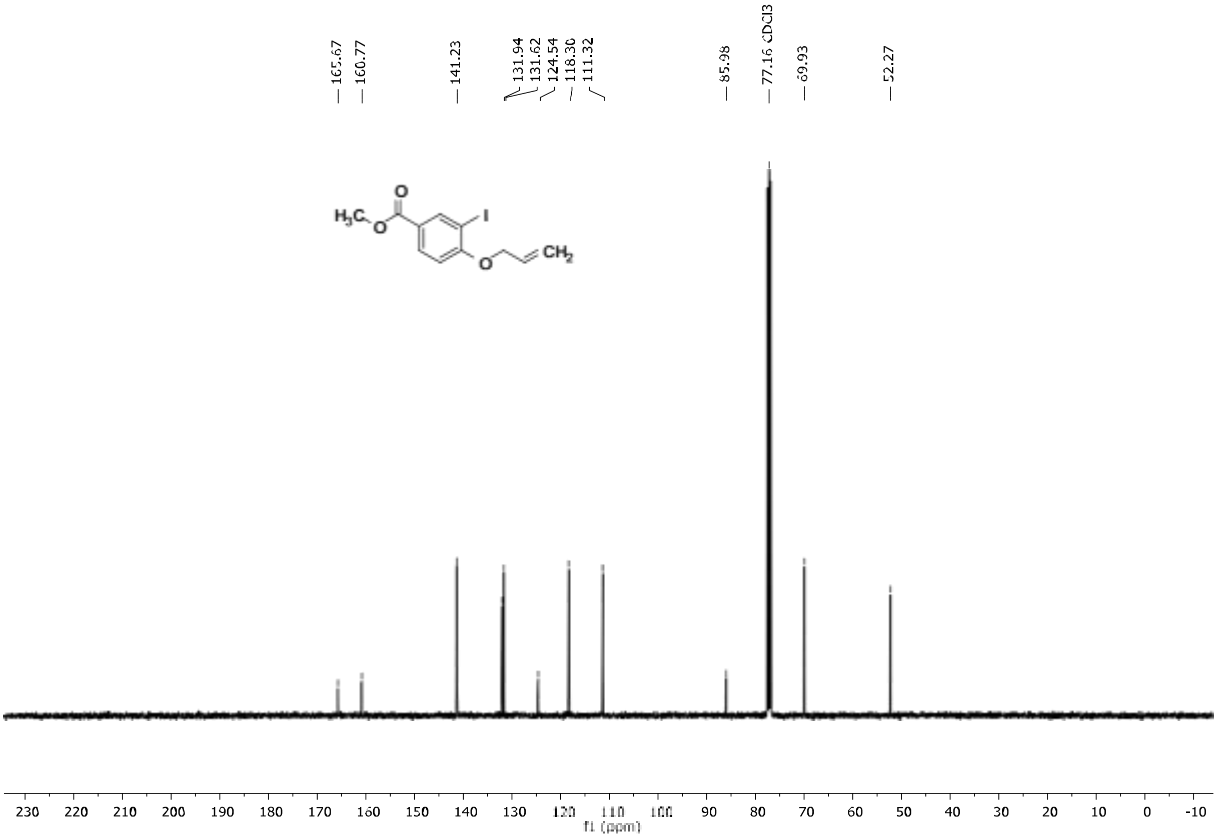

*2-(Allyloxy)-1-iodo-4-methoxybenzene (**SI-10**)*

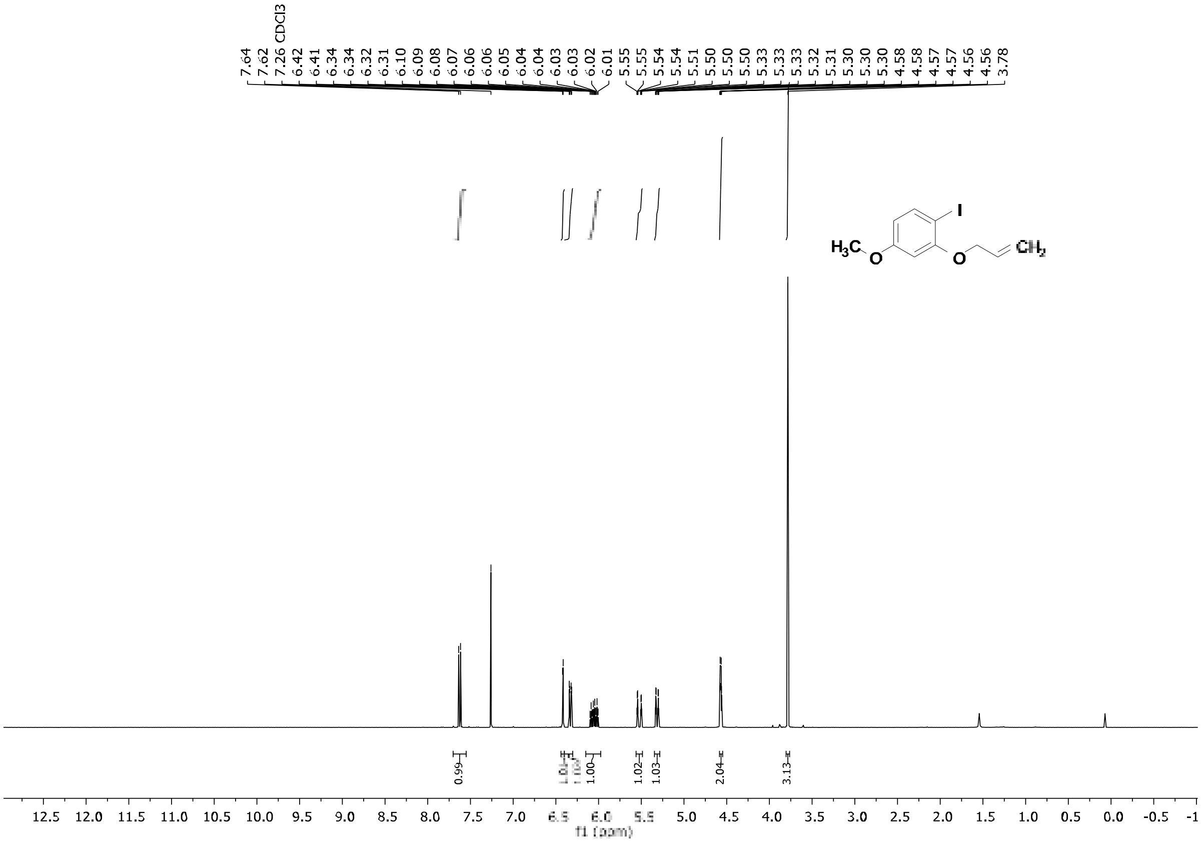

*Methyl 3-(allyloxy)-4-iodobenzoate (**SI-11**)*

CDCl3

7.87
7.85
7.38
7.38
7.36
6.11
6.08
6.08
6.07
6.06
6.05
6.04
5.57
5.57
5.56
5.53
5.52
5.52
5.35
5.32
4.67
4.67
4.66
4.66
4.65
4.65
3.91

1.00
1.00
1.00
0.99
1.01
1.03
1.04

161.36
158.03
139.34
132.63
117.82
107.46
100.74
77.16 CDCl3
75.60
69.81
55.65

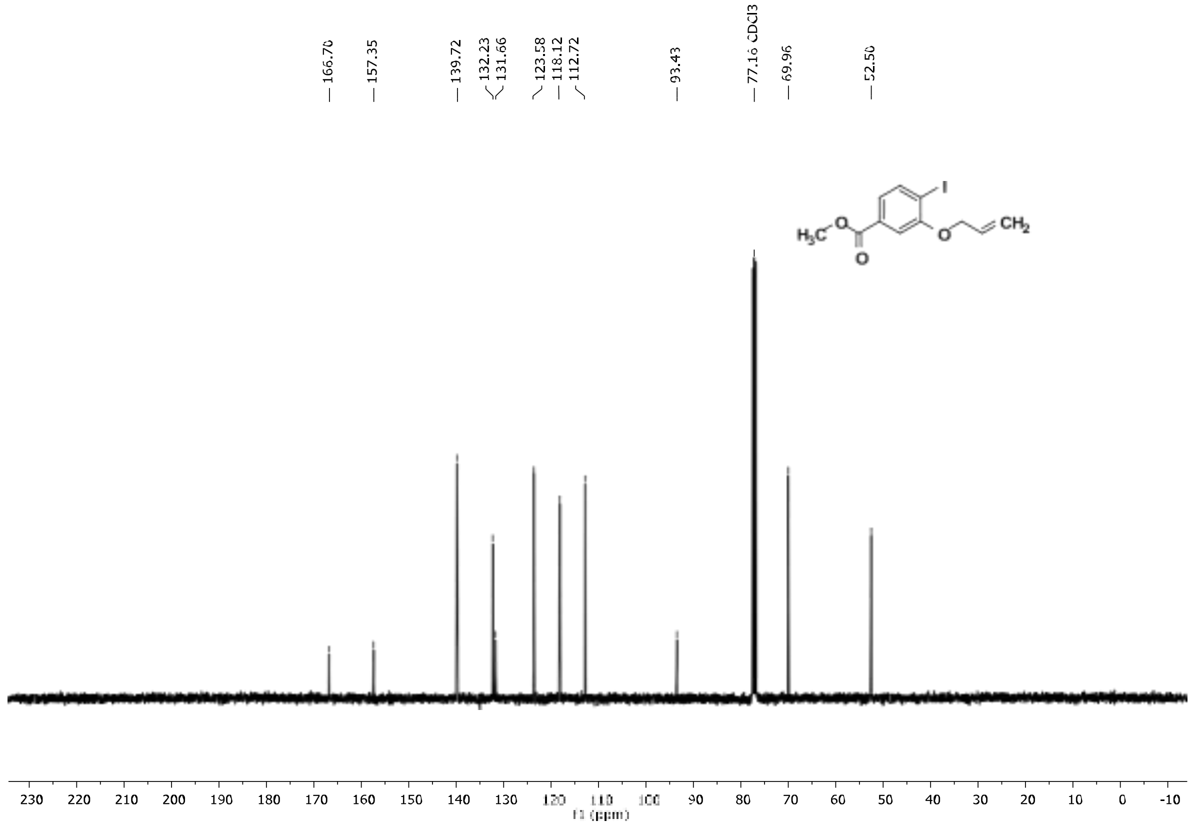

166.70
157.35
139.72
132.23
131.66
123.58
118.12
112.72
93.43
77.16 CDCl3
69.96
52.50
I
H3C
O
O
O
CH2
230 220 210 200 190 180 170 160 150 140 130 120 110 100 90 80 70 60 50 40 30 20 10 0 -10
f1 (ppm)

NMR Spectra of Benzofurans

*5-Phenylbenzofuran (**4b**)*

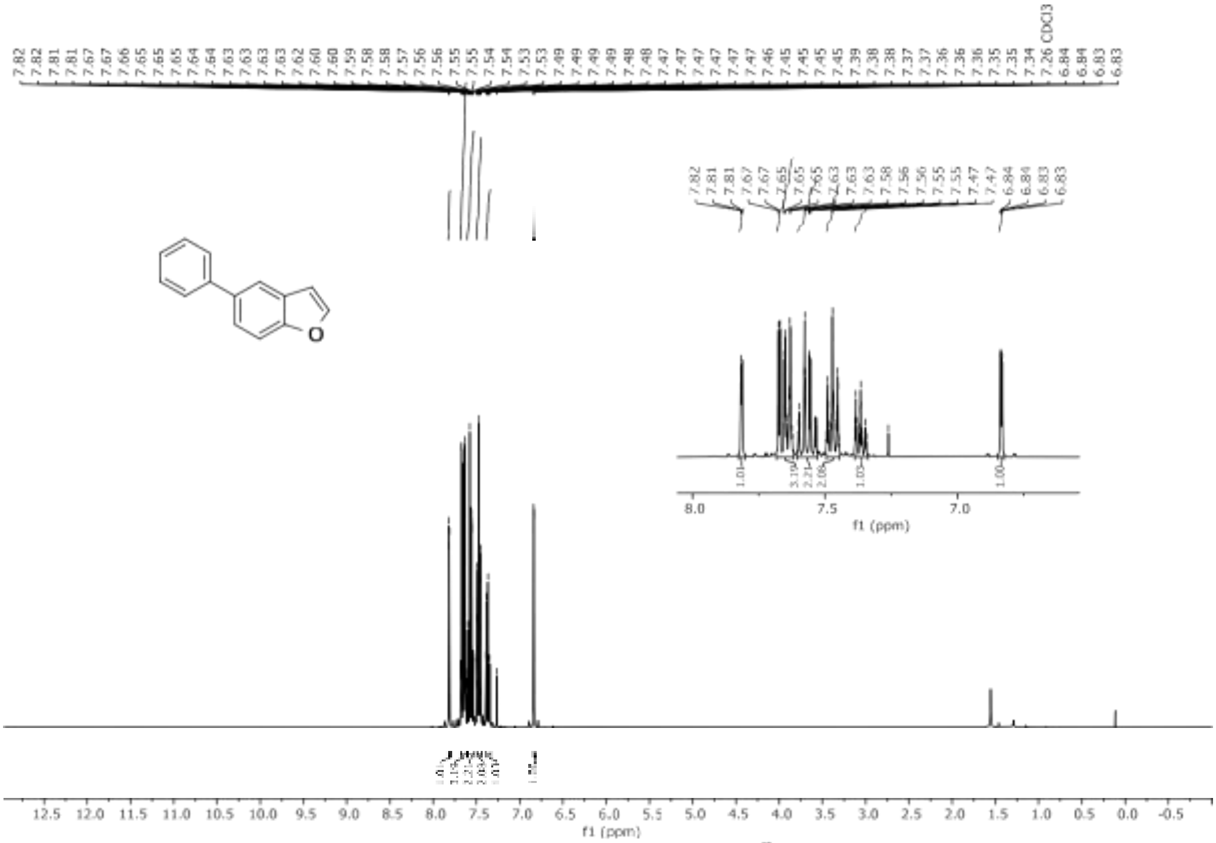

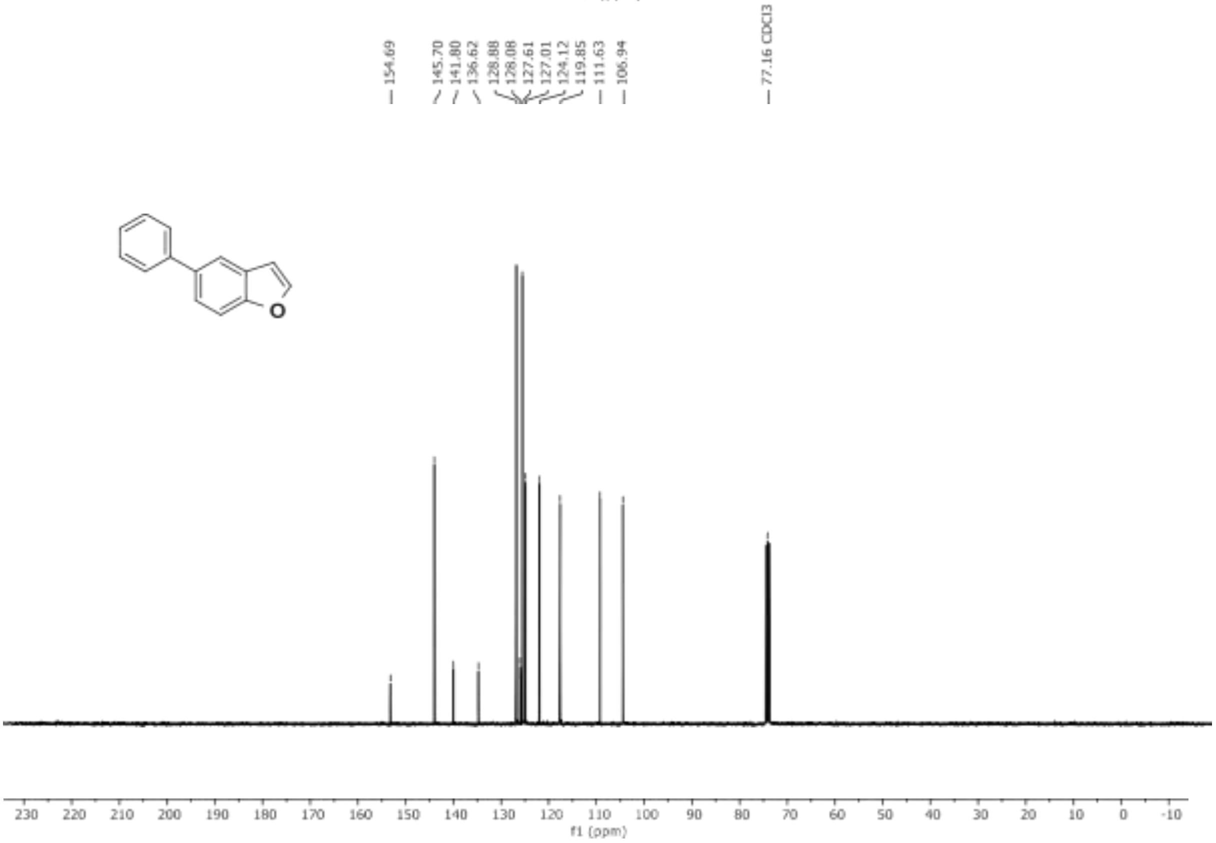

*Methyl benzofuran-5-carboxylate (**4c**)*

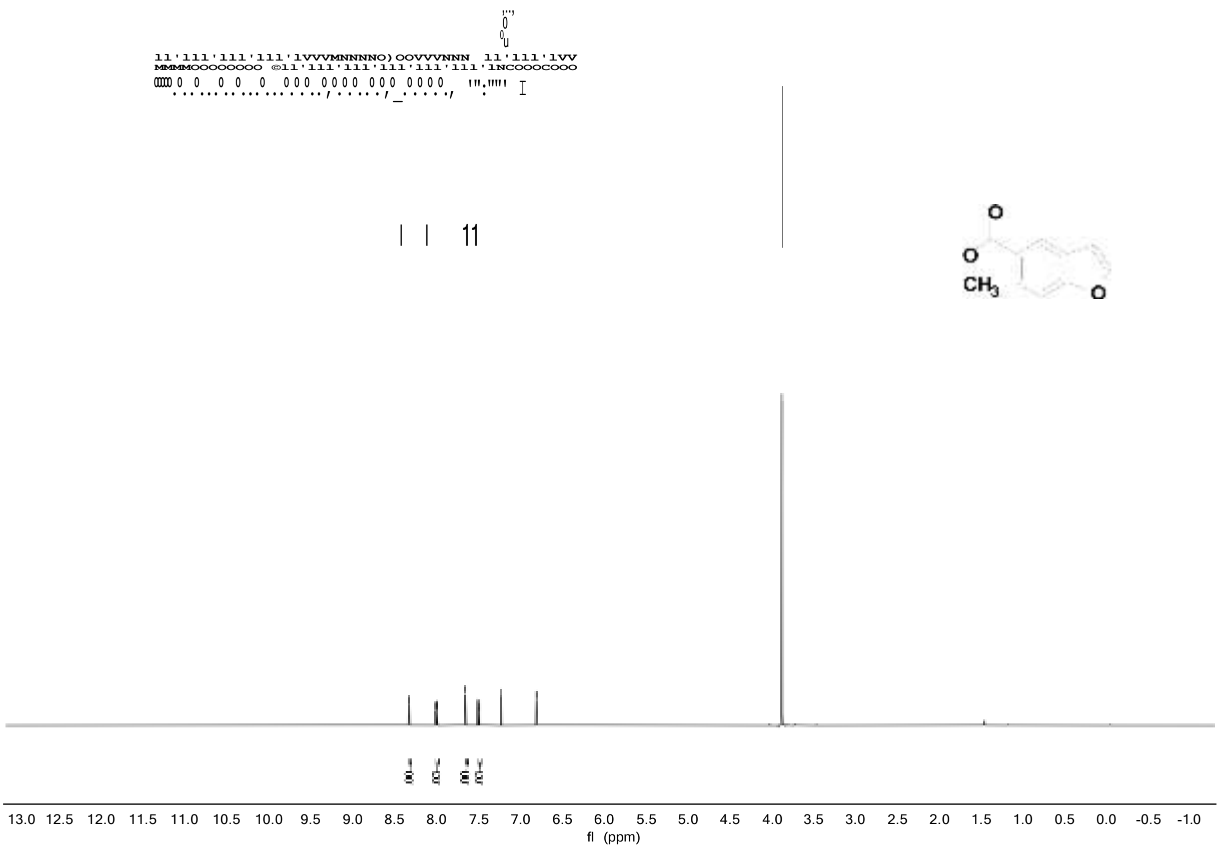

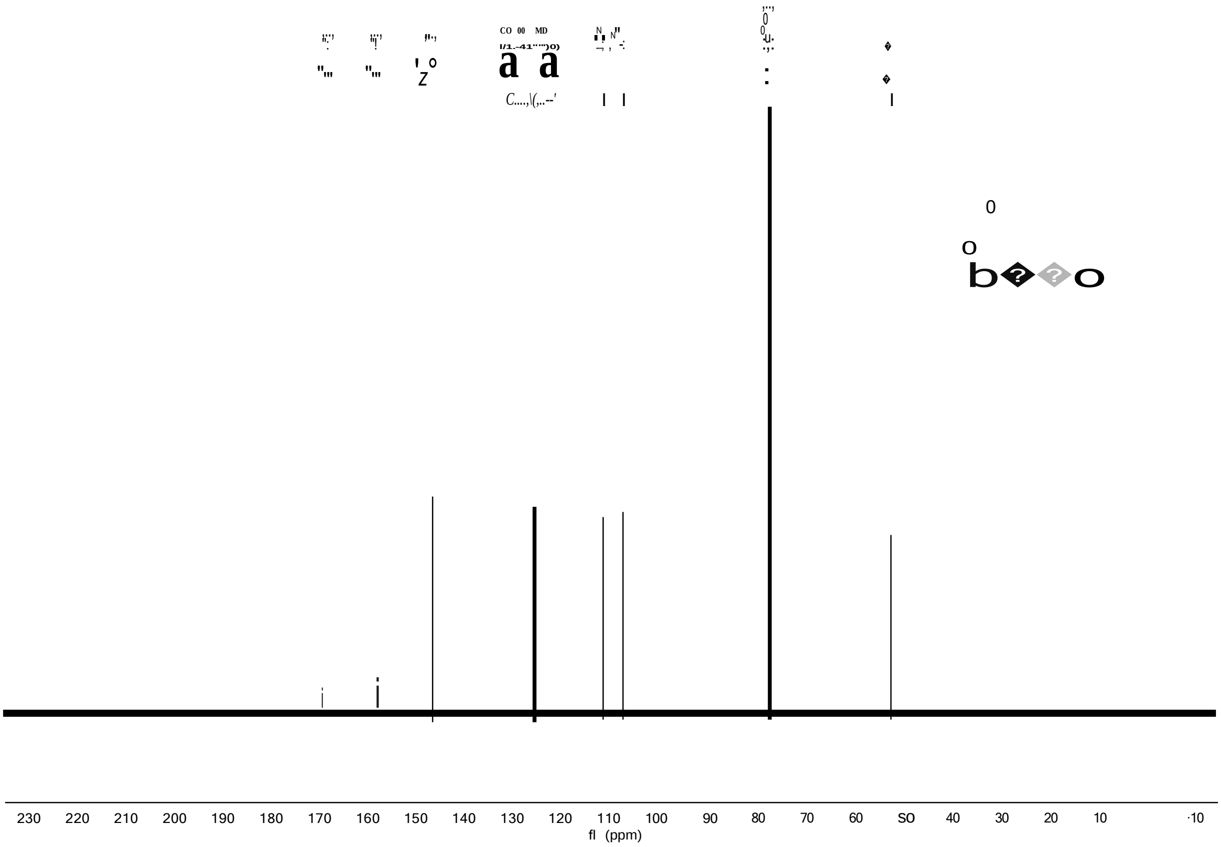

tert-Butyl benzofuran-5-ylcarbamate (**4d**)

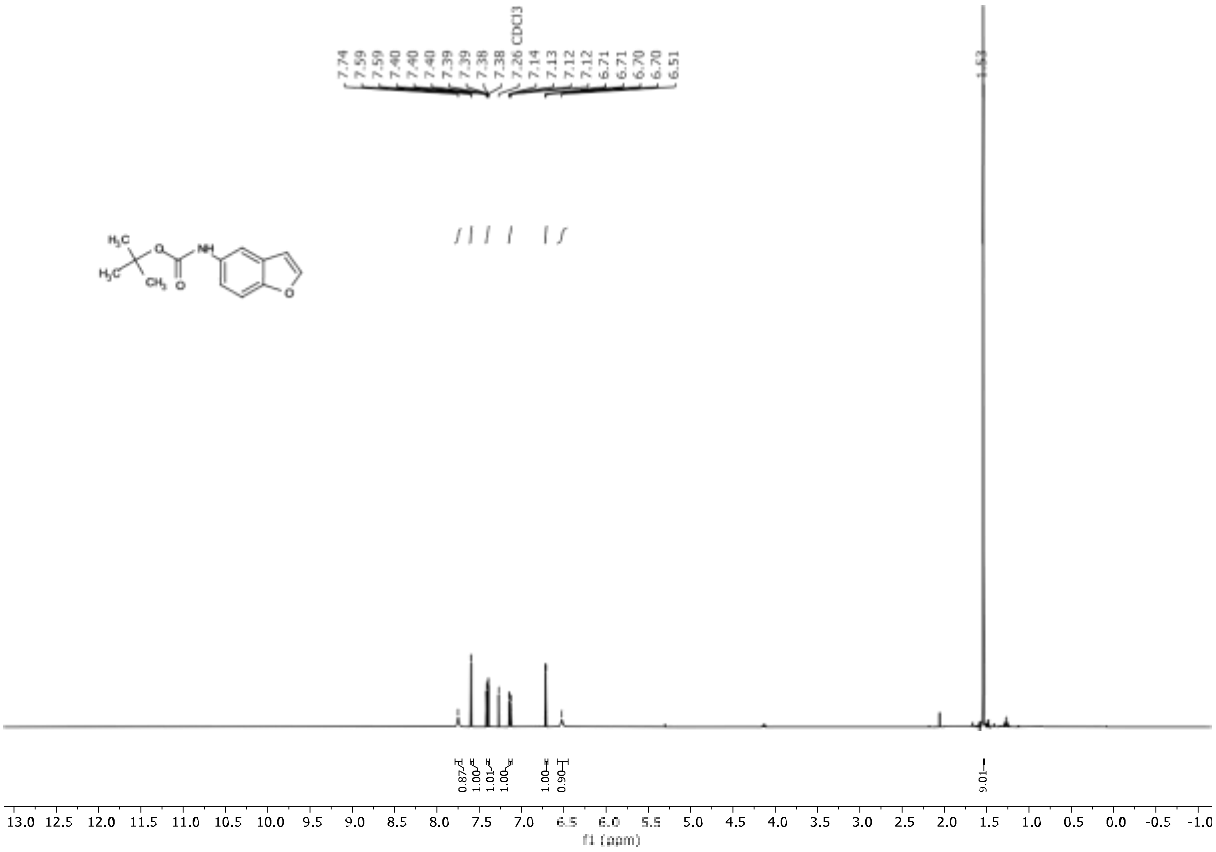

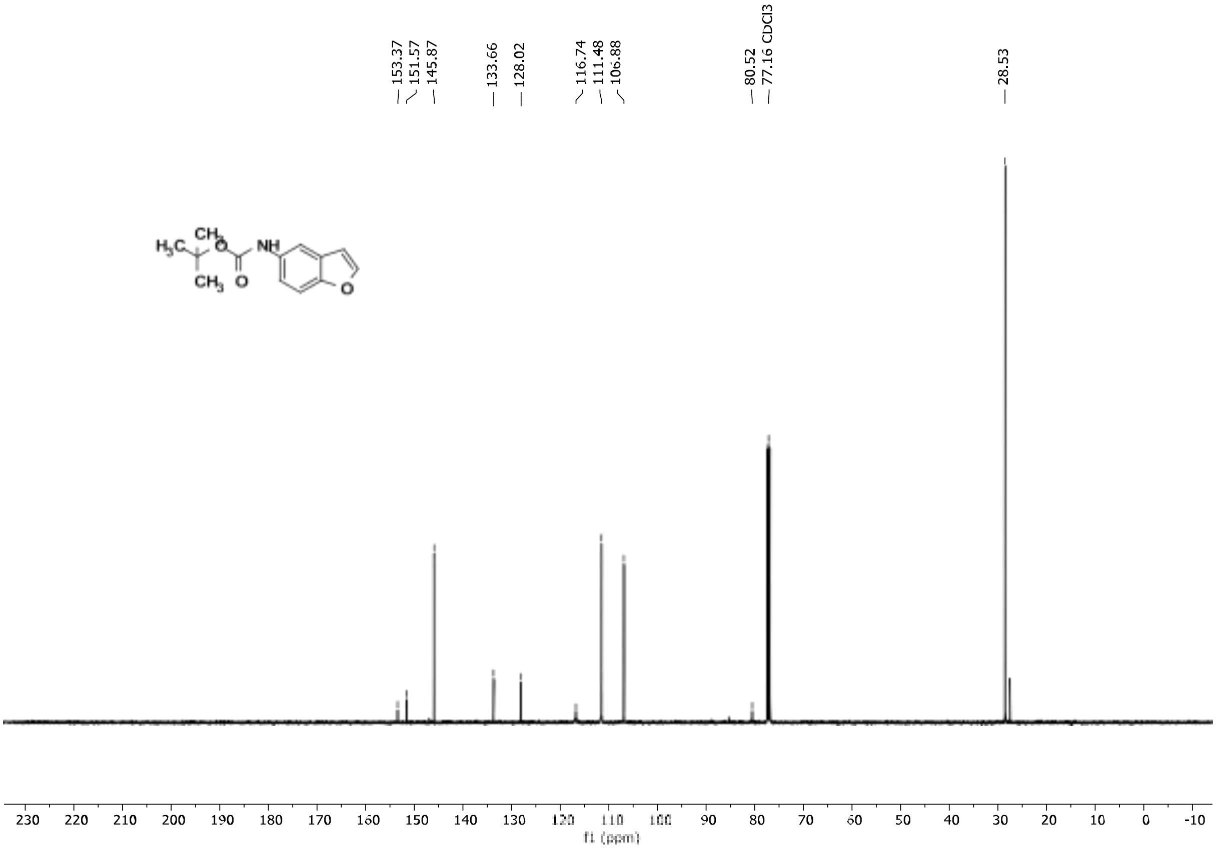

*6-Phenylbenzofuran (**4e**)*

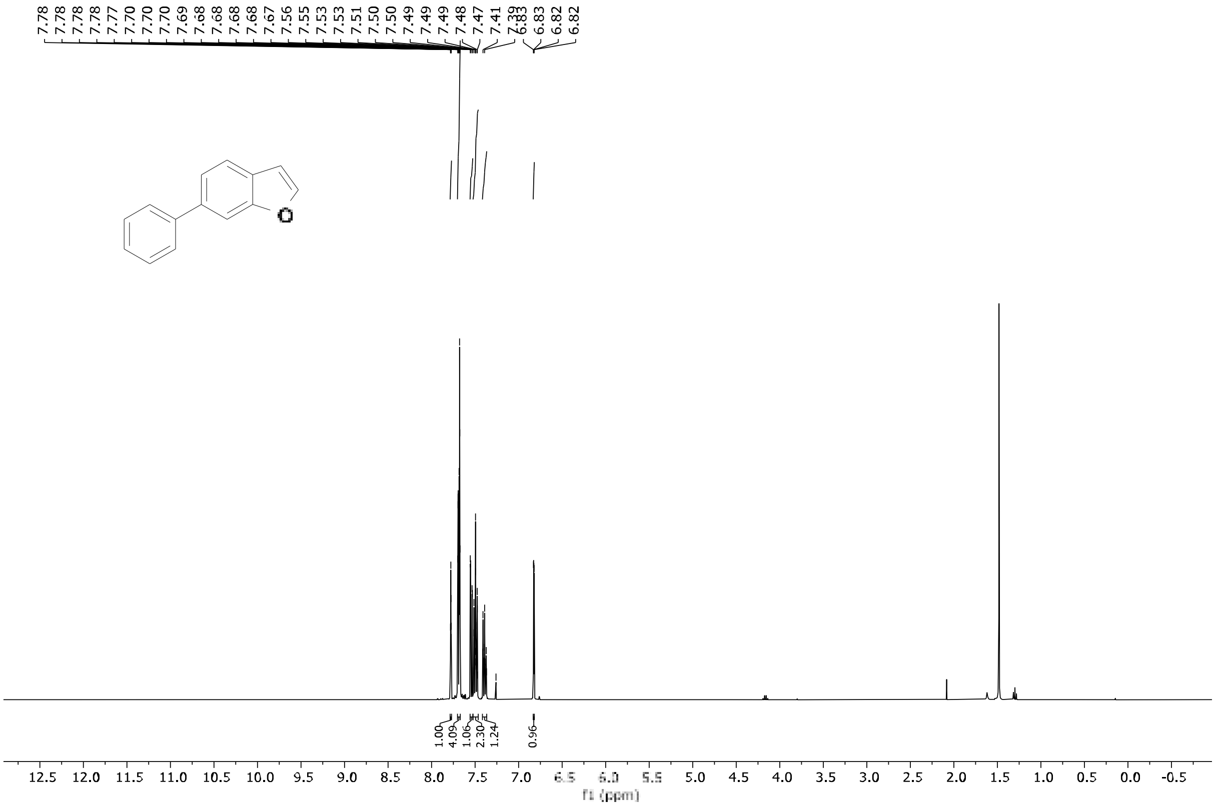

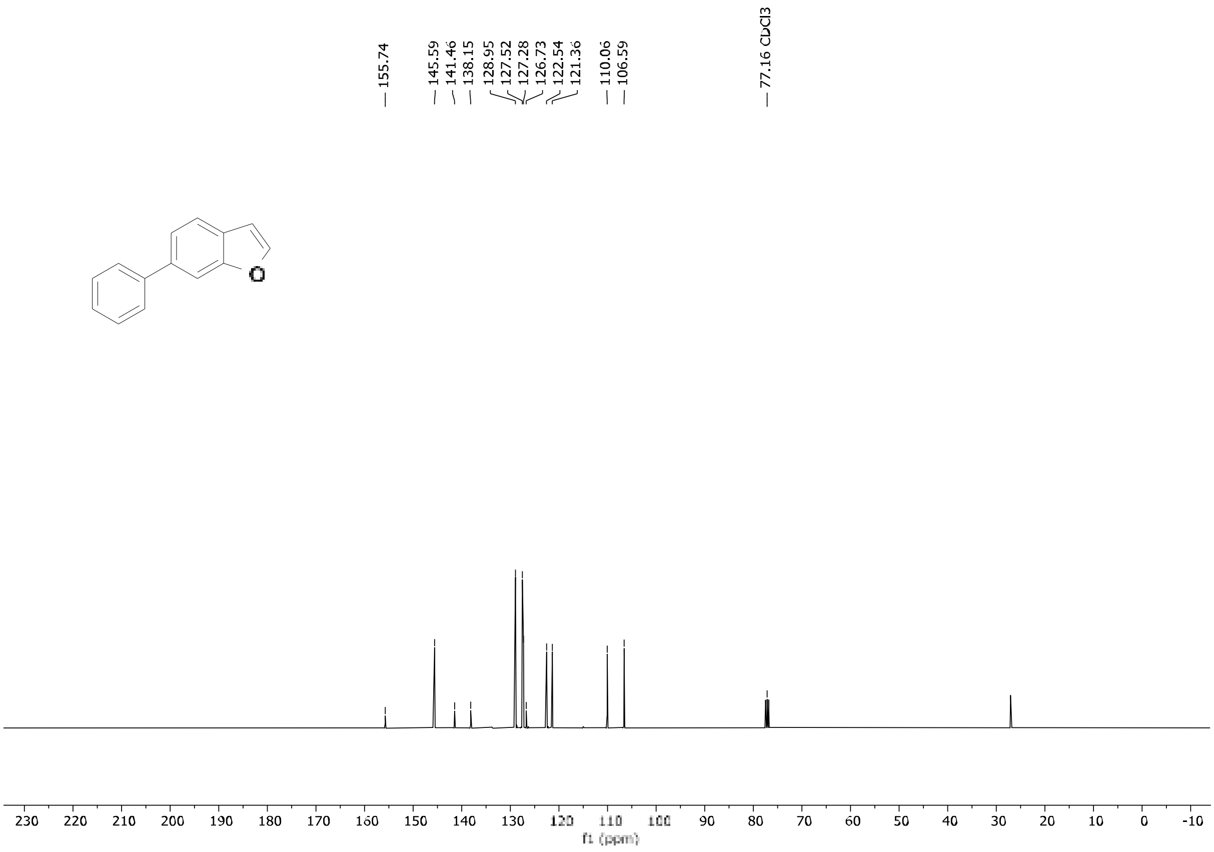

6-(4-(Methylsulfonyl)phenyl)benzofuran (**4h**)

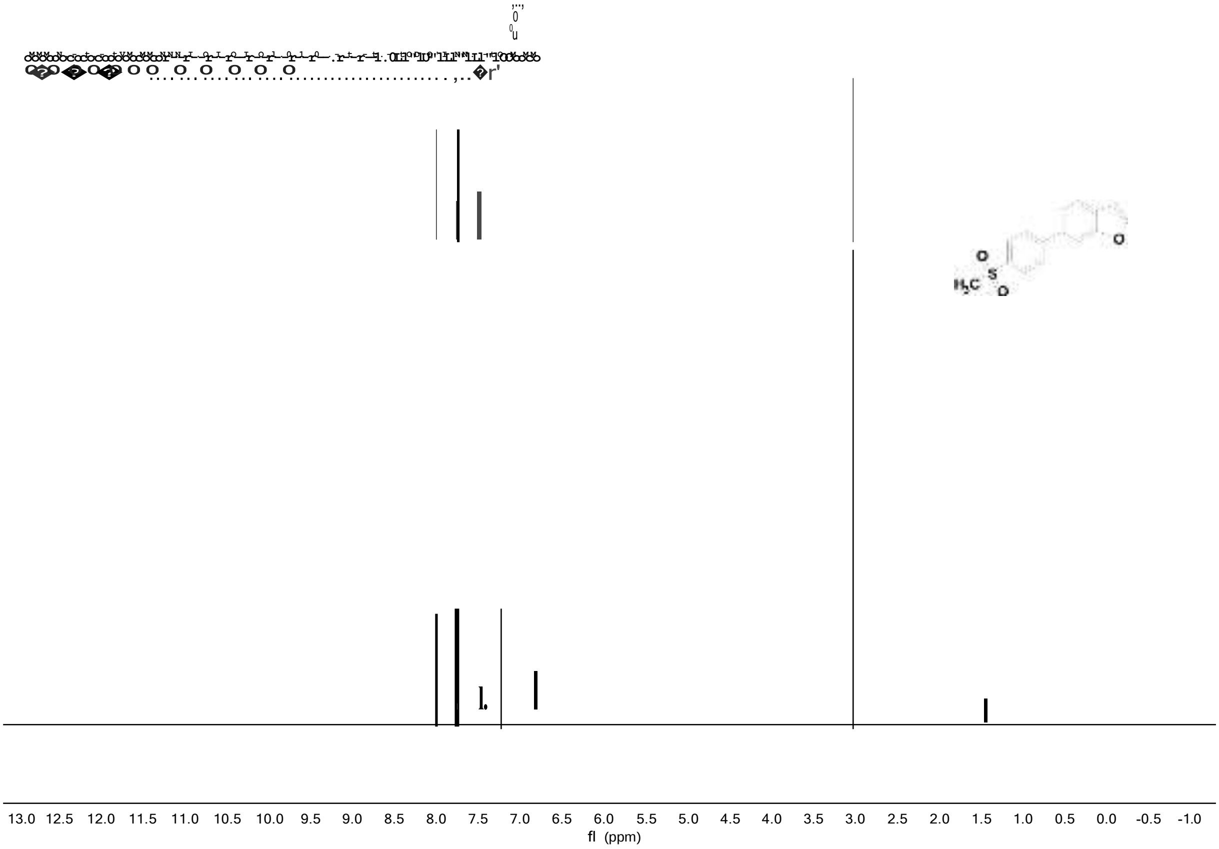

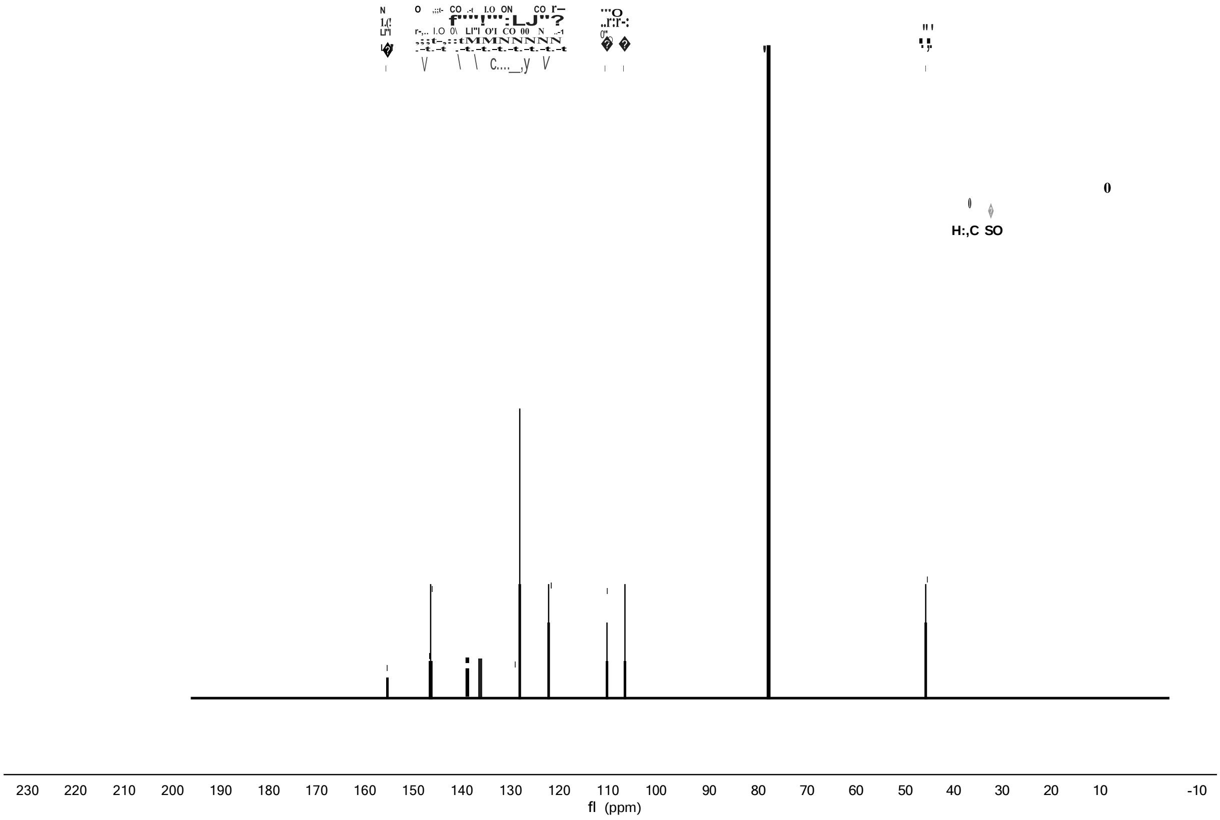

*5-(3-chlorophenyl)benzofuran (**4i**)*

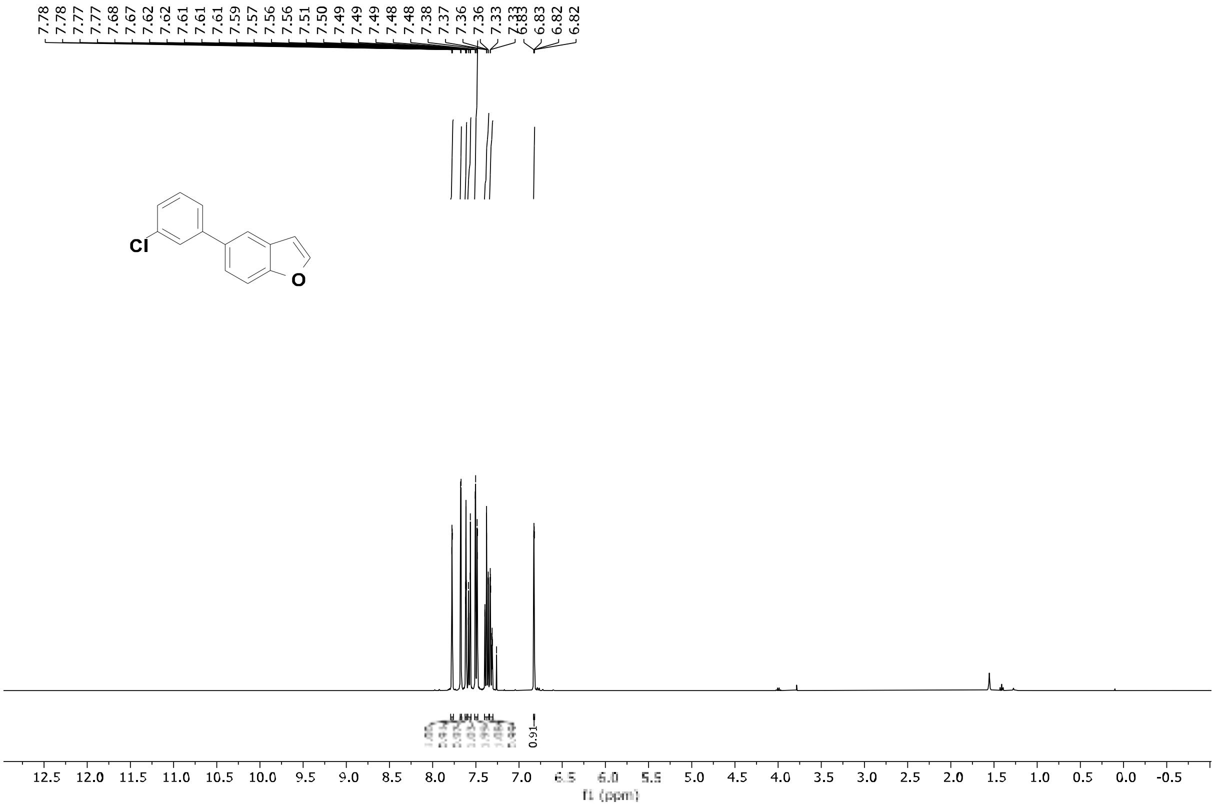

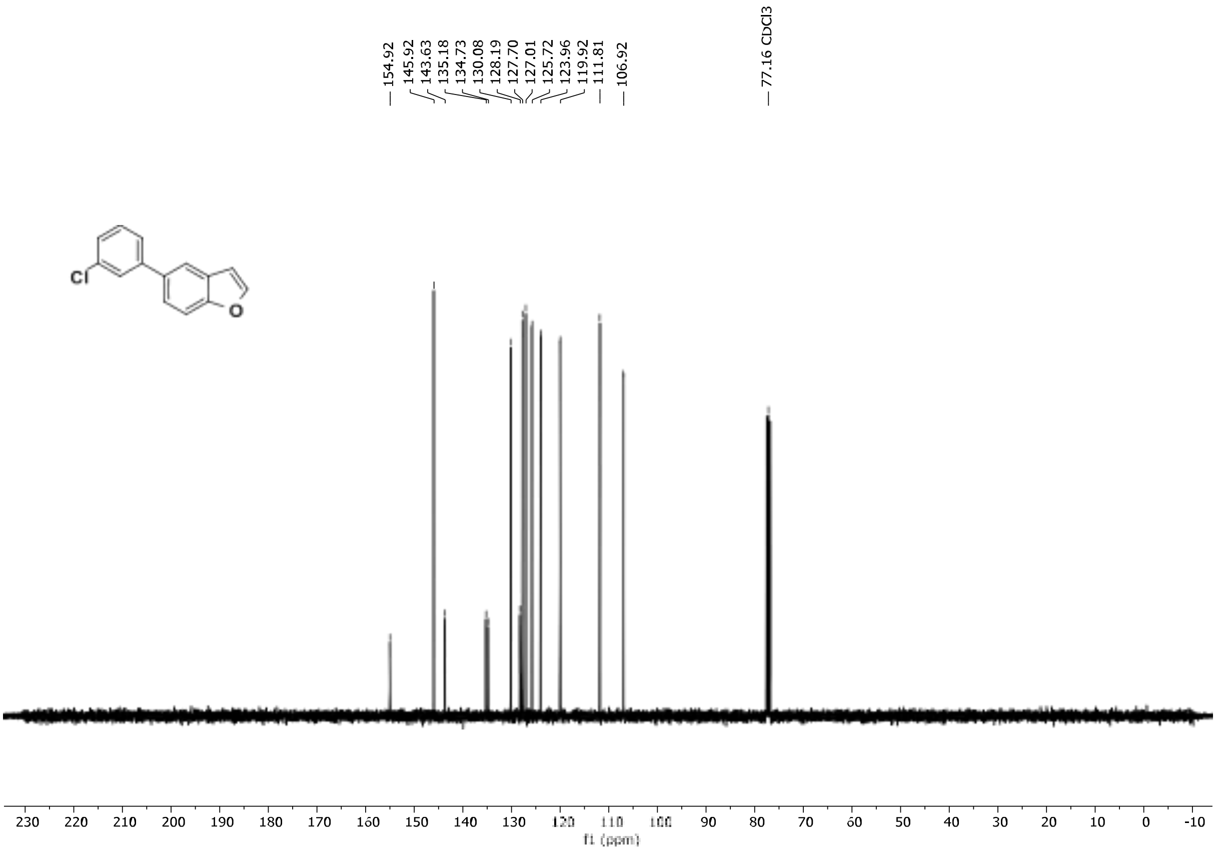

*5-(2-(Trifluoromethyl)phenyl)benzofuran (**4j**)*

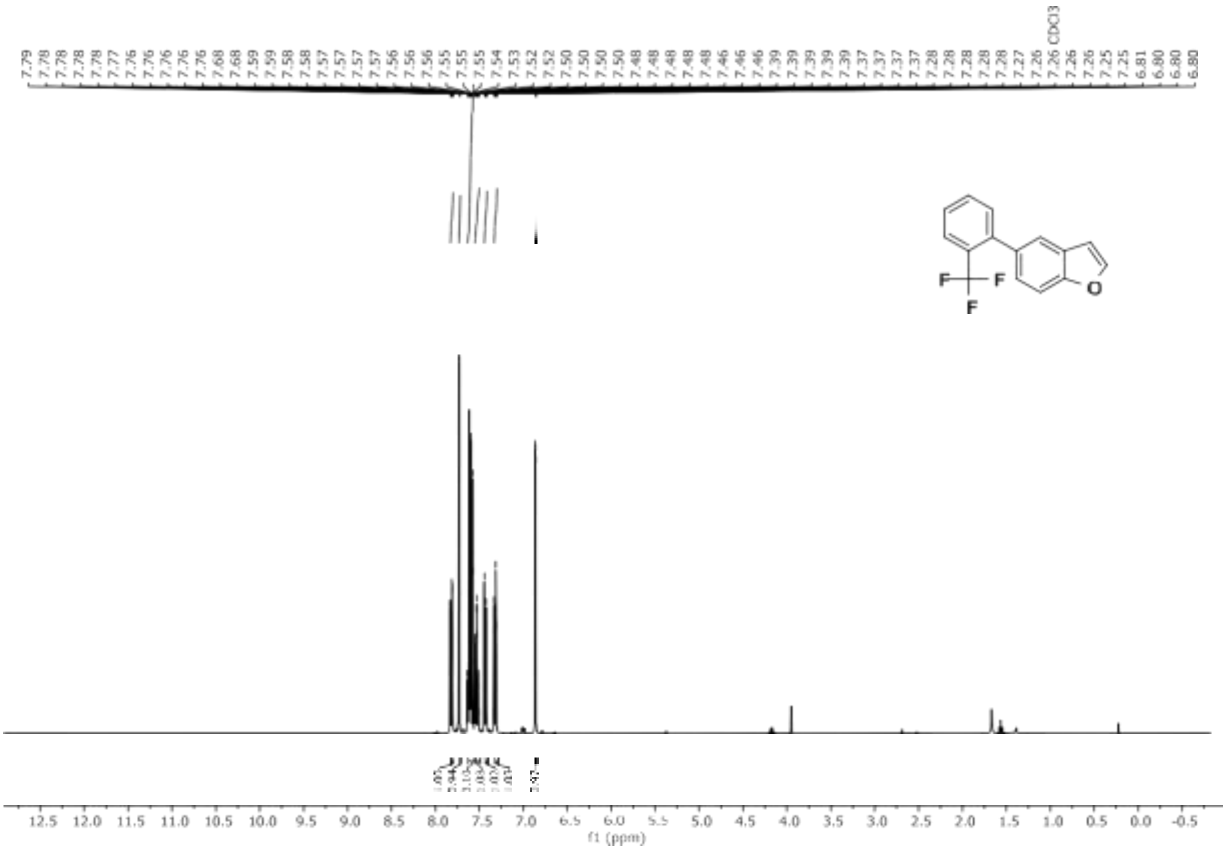

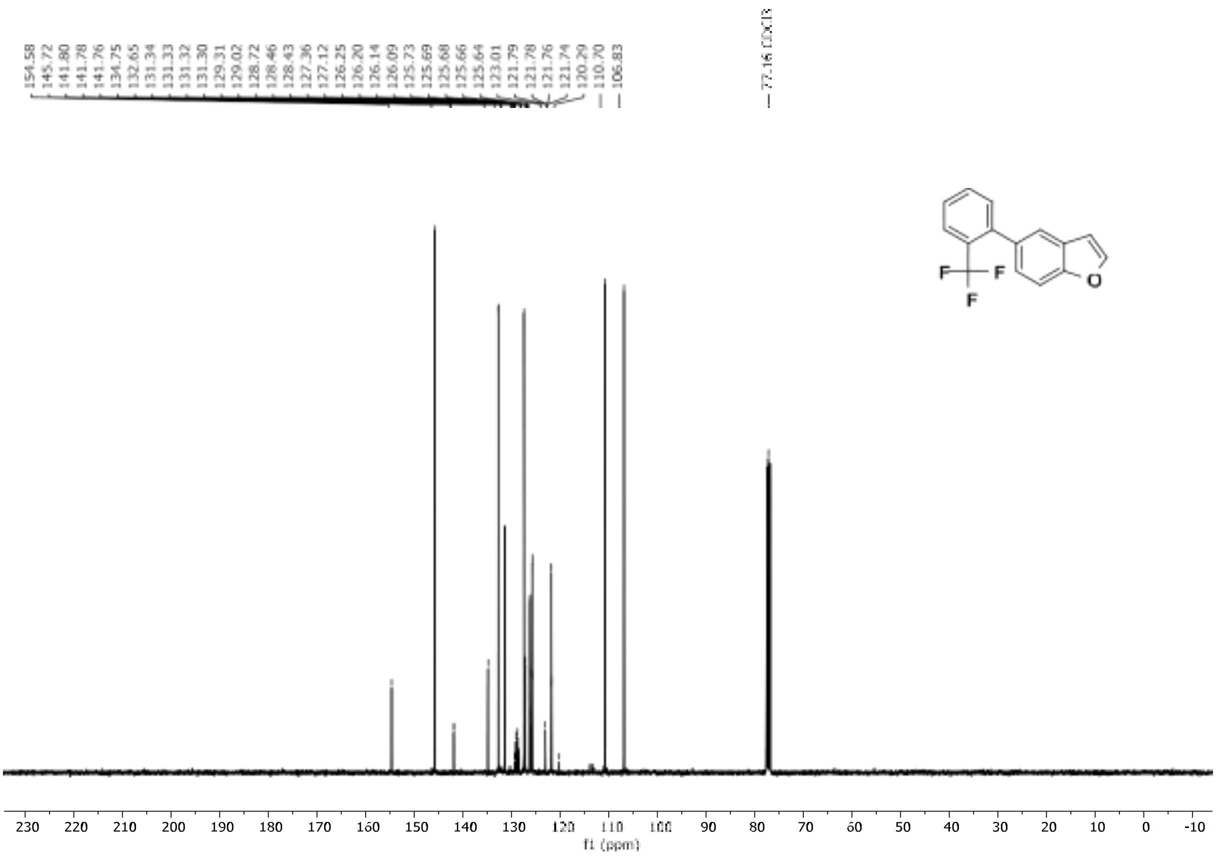

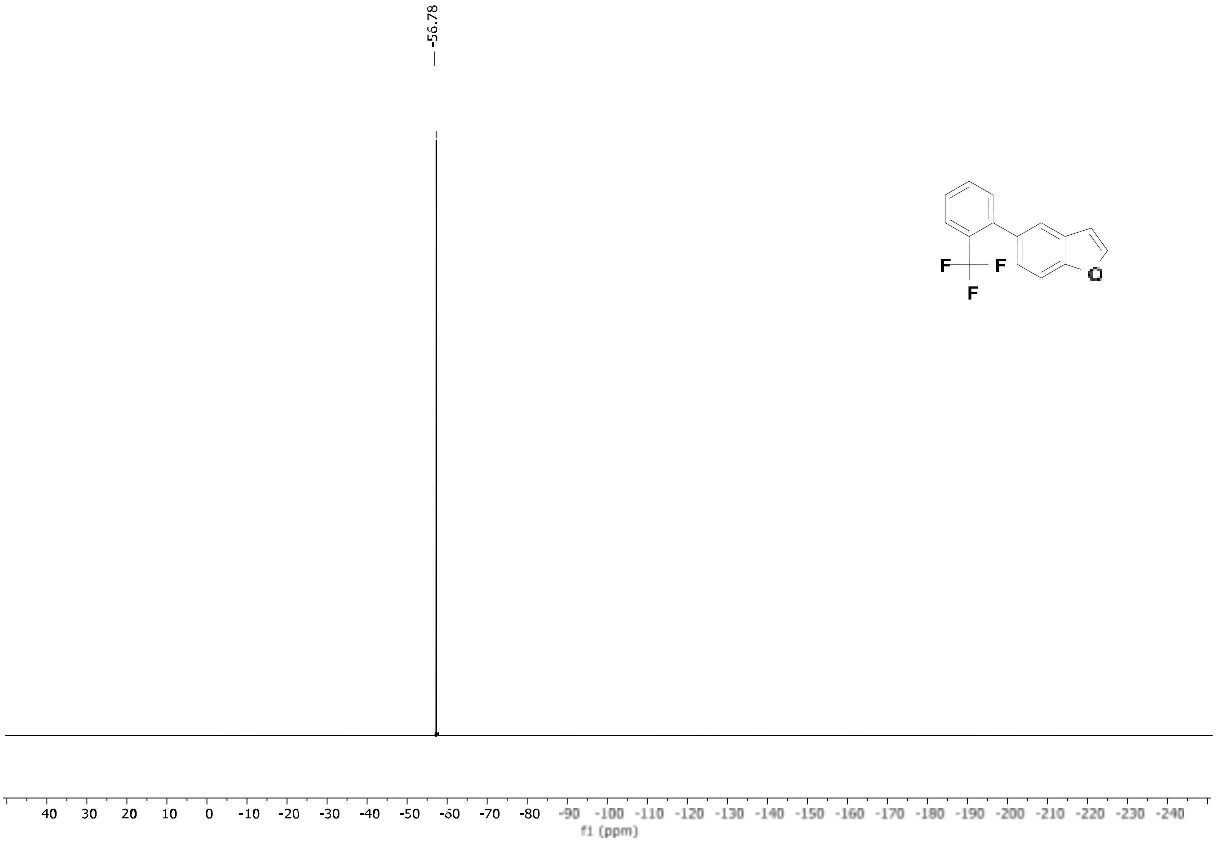
-56.78
F
F
F
O
40 30 20 10 0 -10 -20 -30 -40 -50 -60 -70 -80 -90 -100 -110 -120 -130 -140 -150 -160 -170 -180 -190 -200 -210 -220 -230 -240
f1 (ppm)

N-*Phenethylbenzofuran-5-carboxamide* (**4k**)

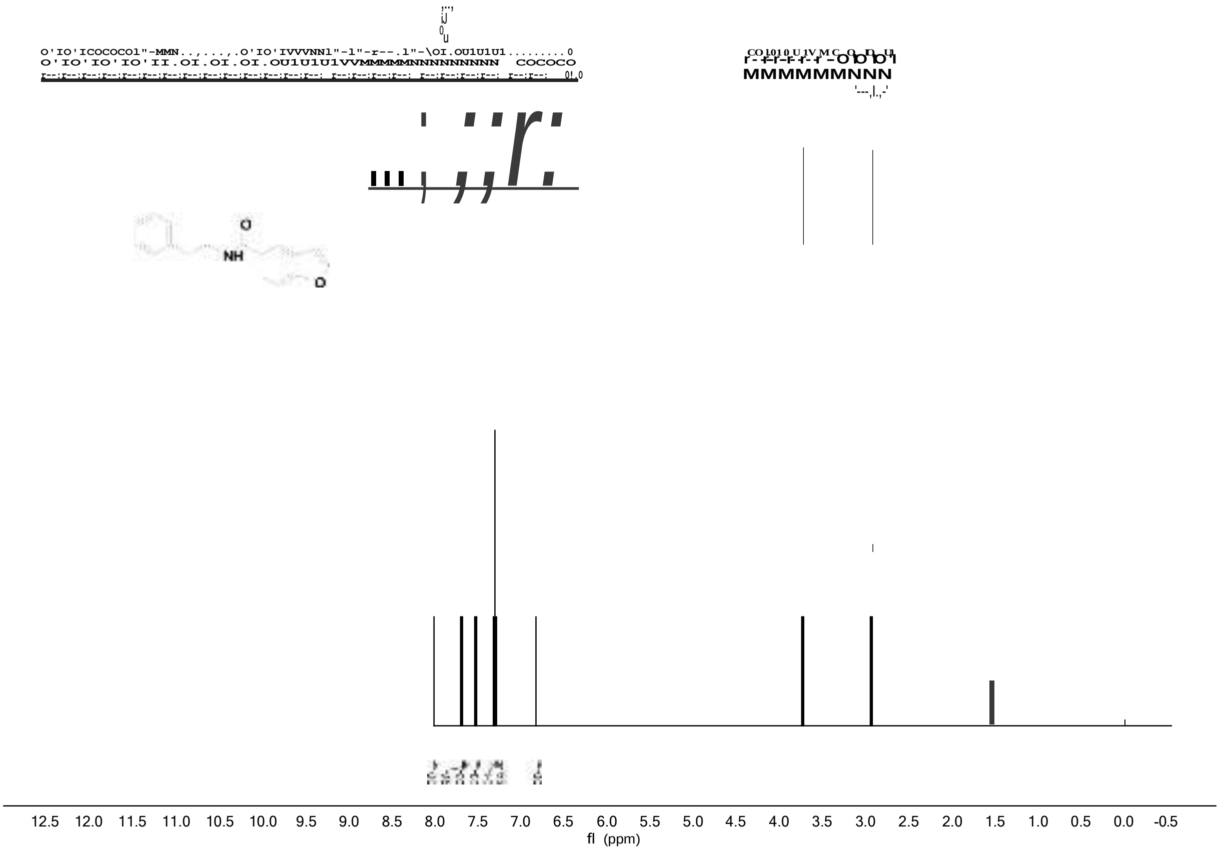

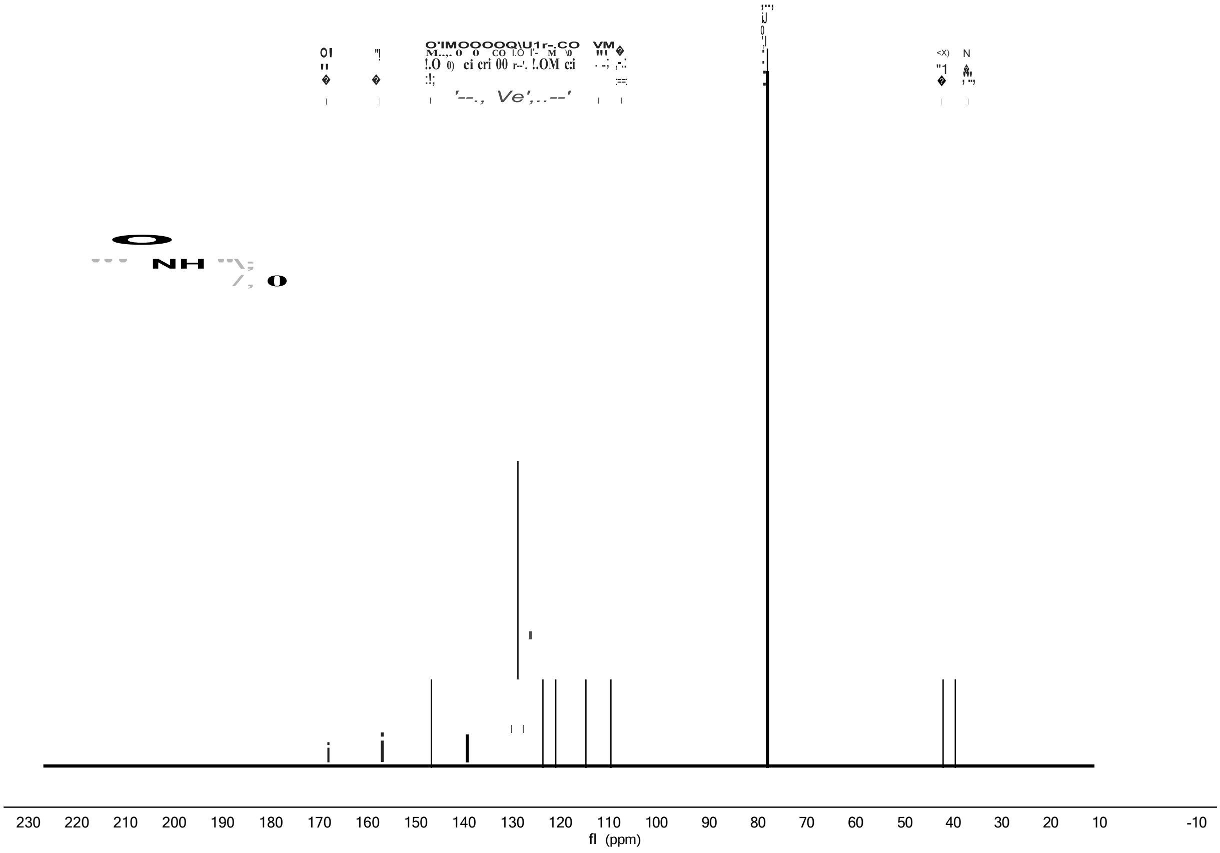

N-*Phenethylbenzofuran-6-carboxamide* (***4l***)

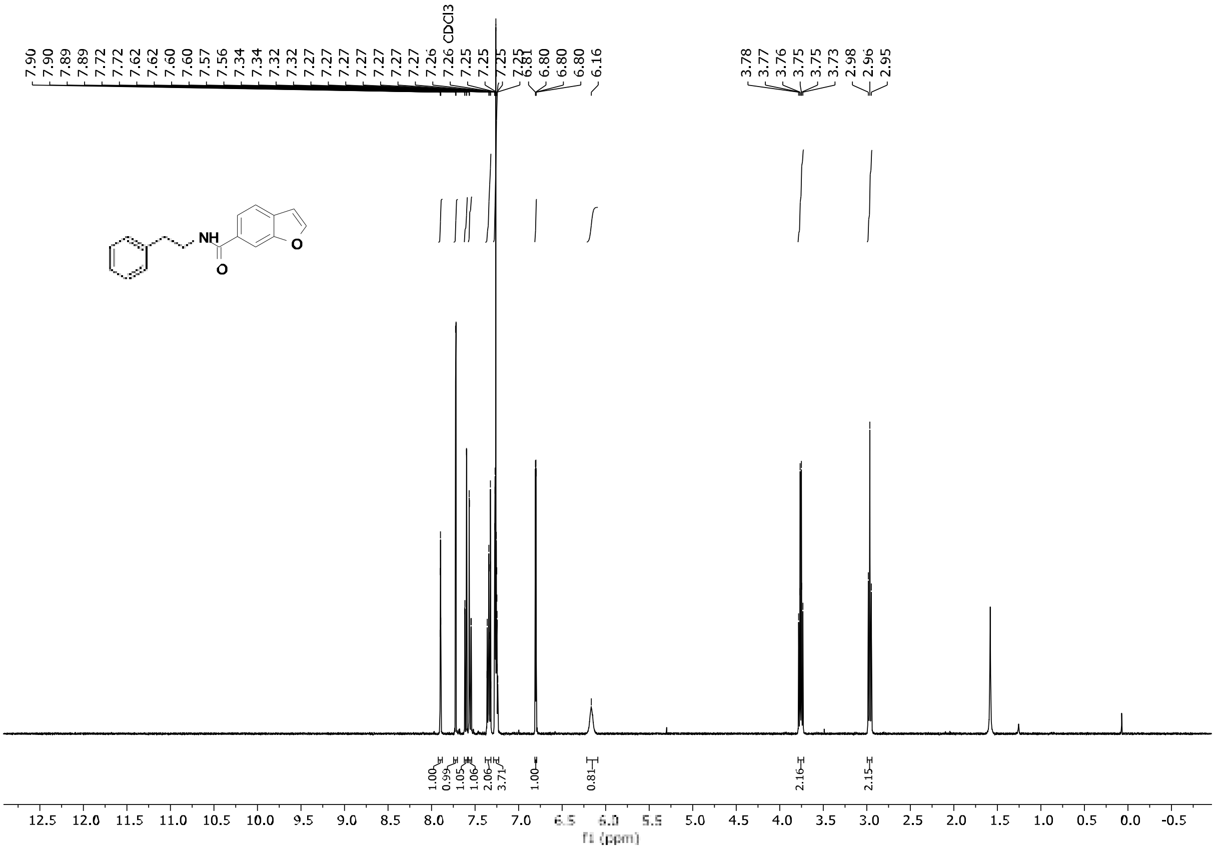

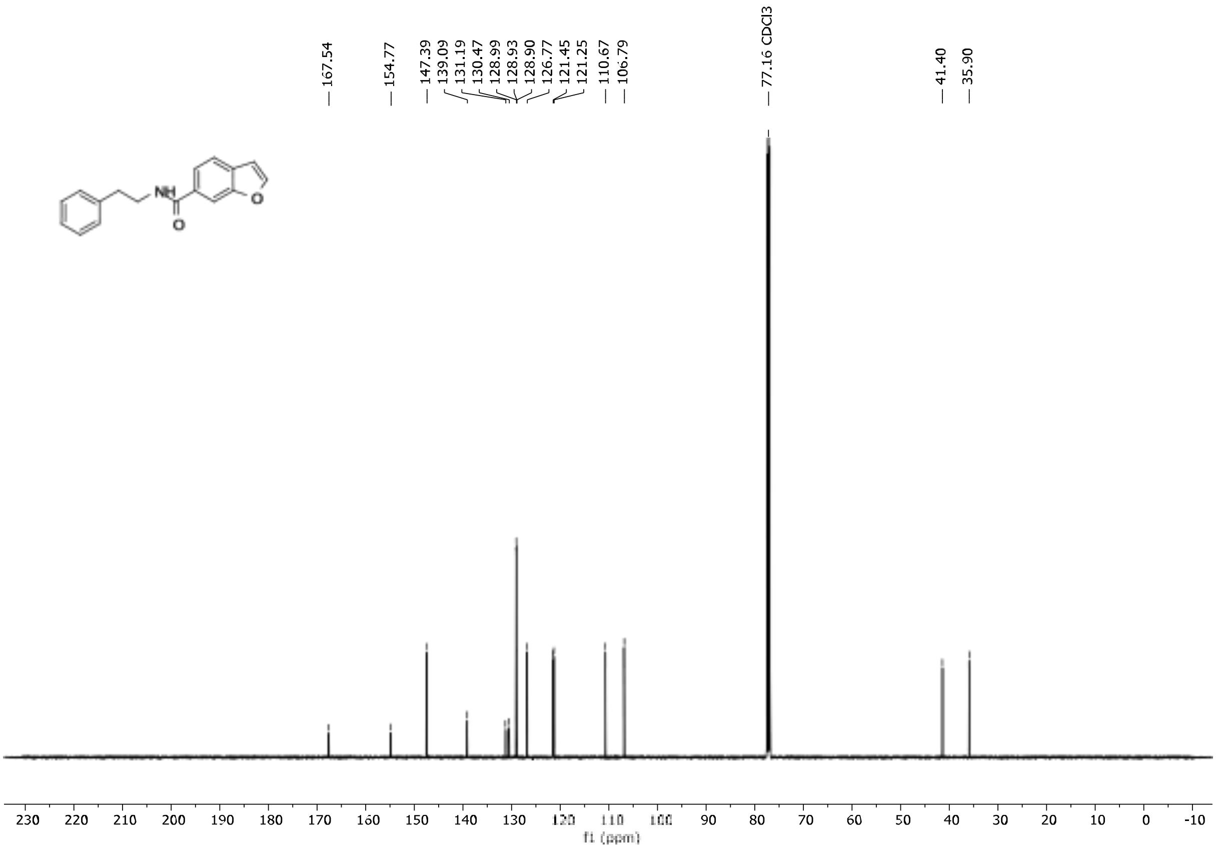

6-Vinylbenzofuran *(4m)*

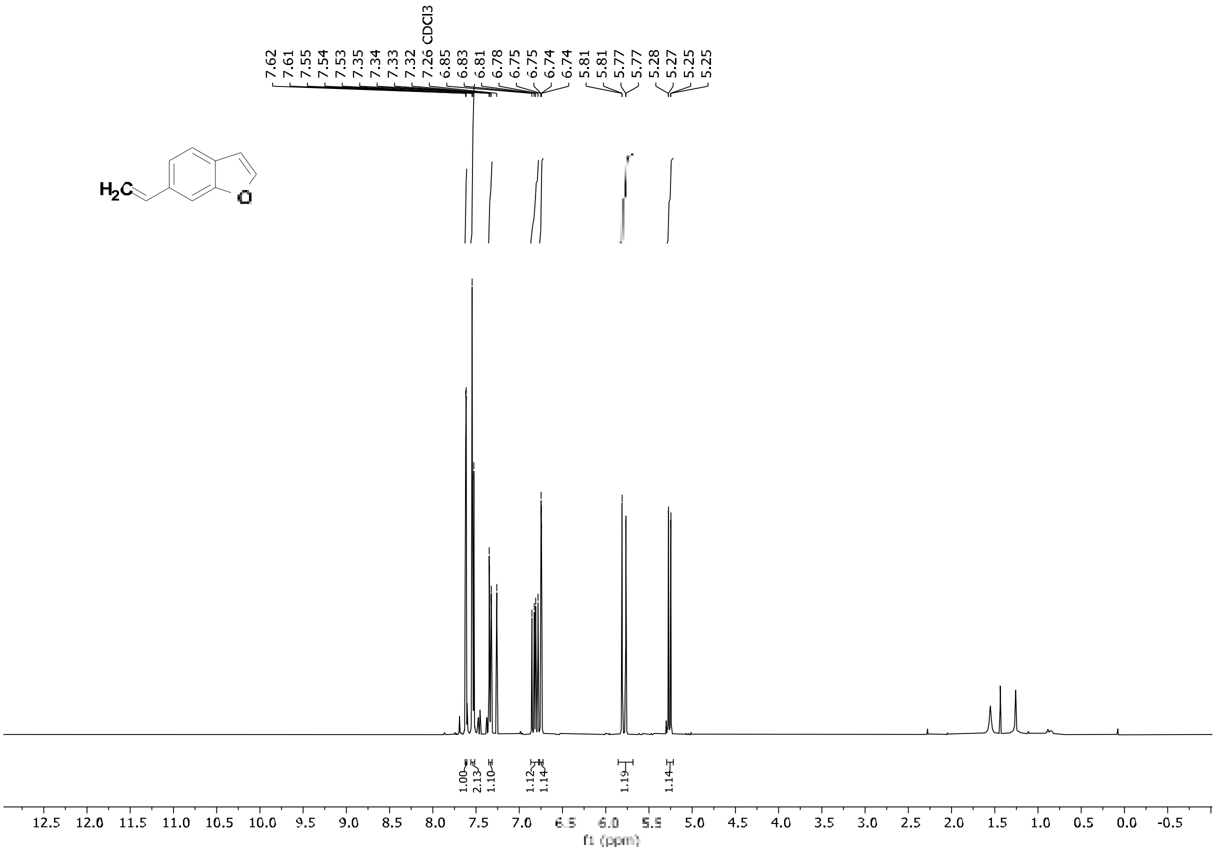

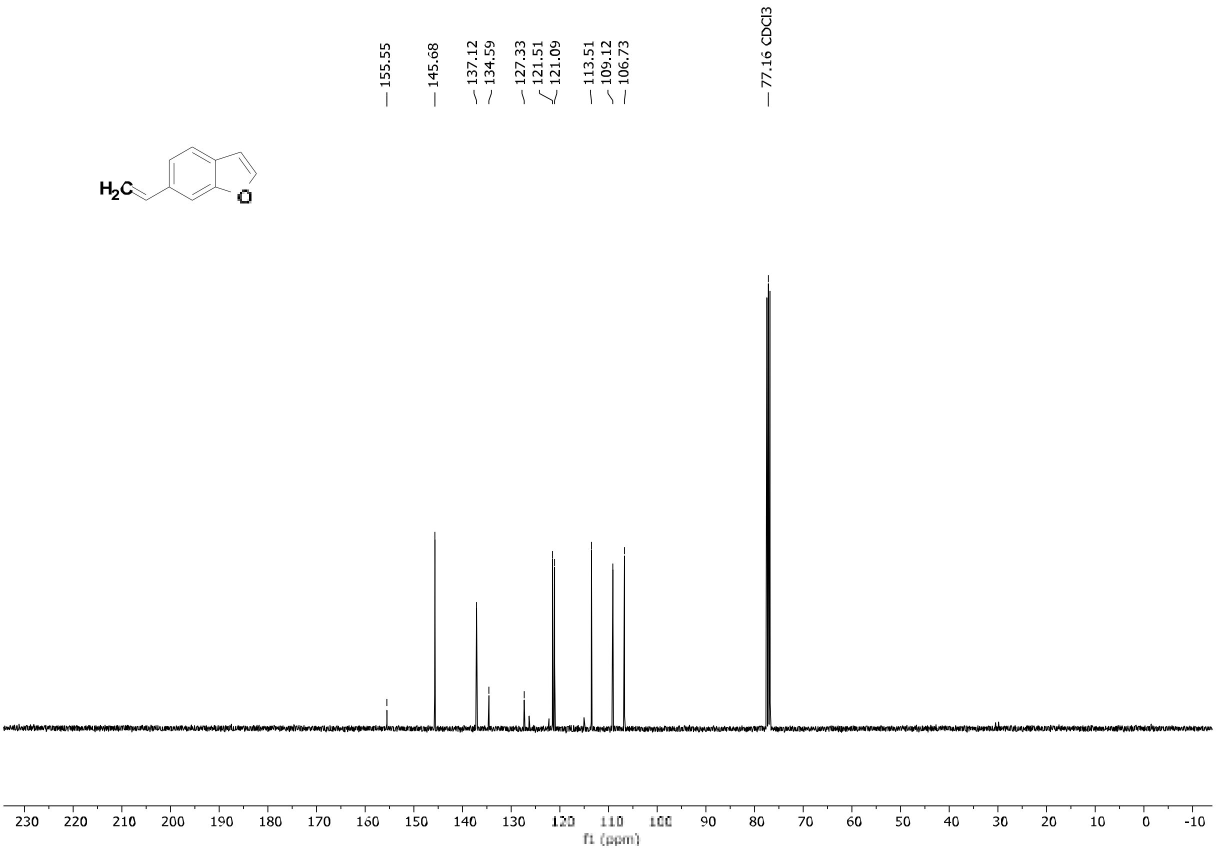

NMR Spectra of 3-substituted Benzofurans

*3-Isopropylbenzofuran (**SI-12**)*

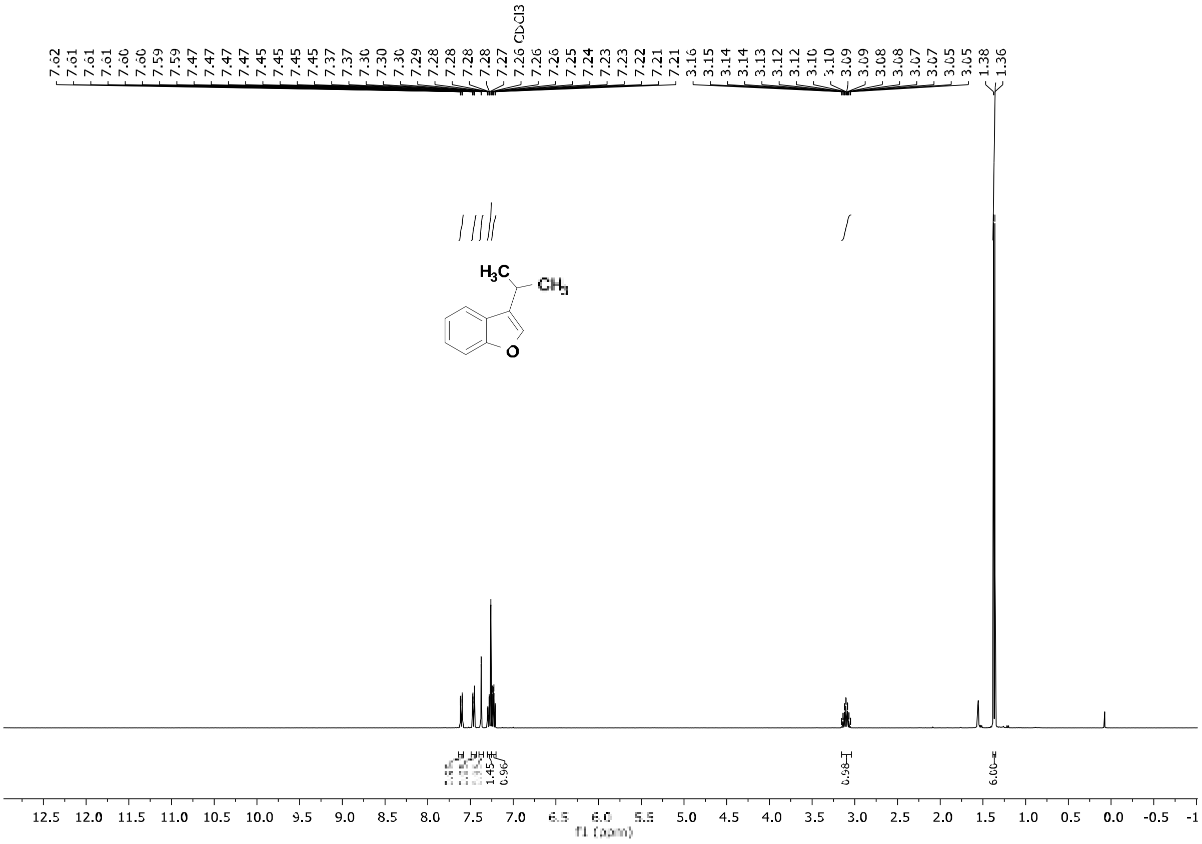

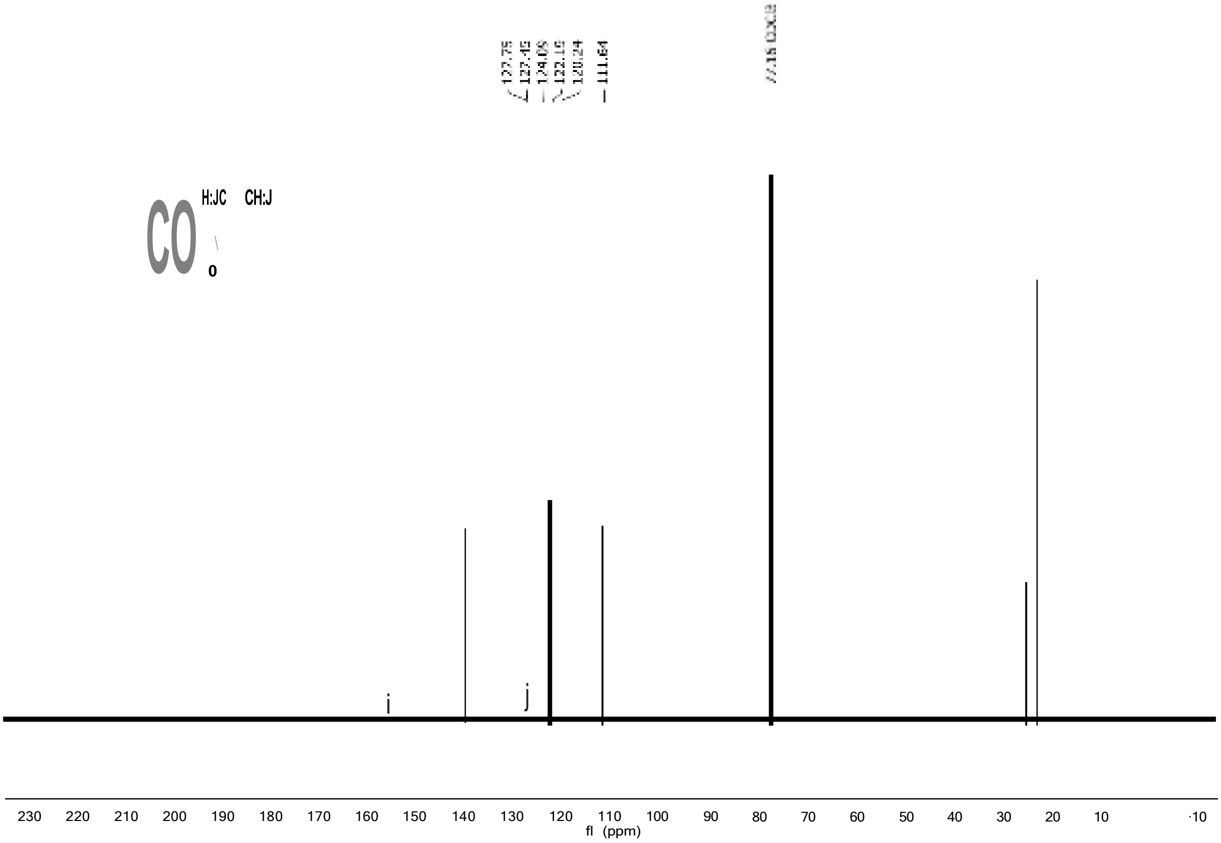

3-Benzylbenzofuran (***SI-13***)

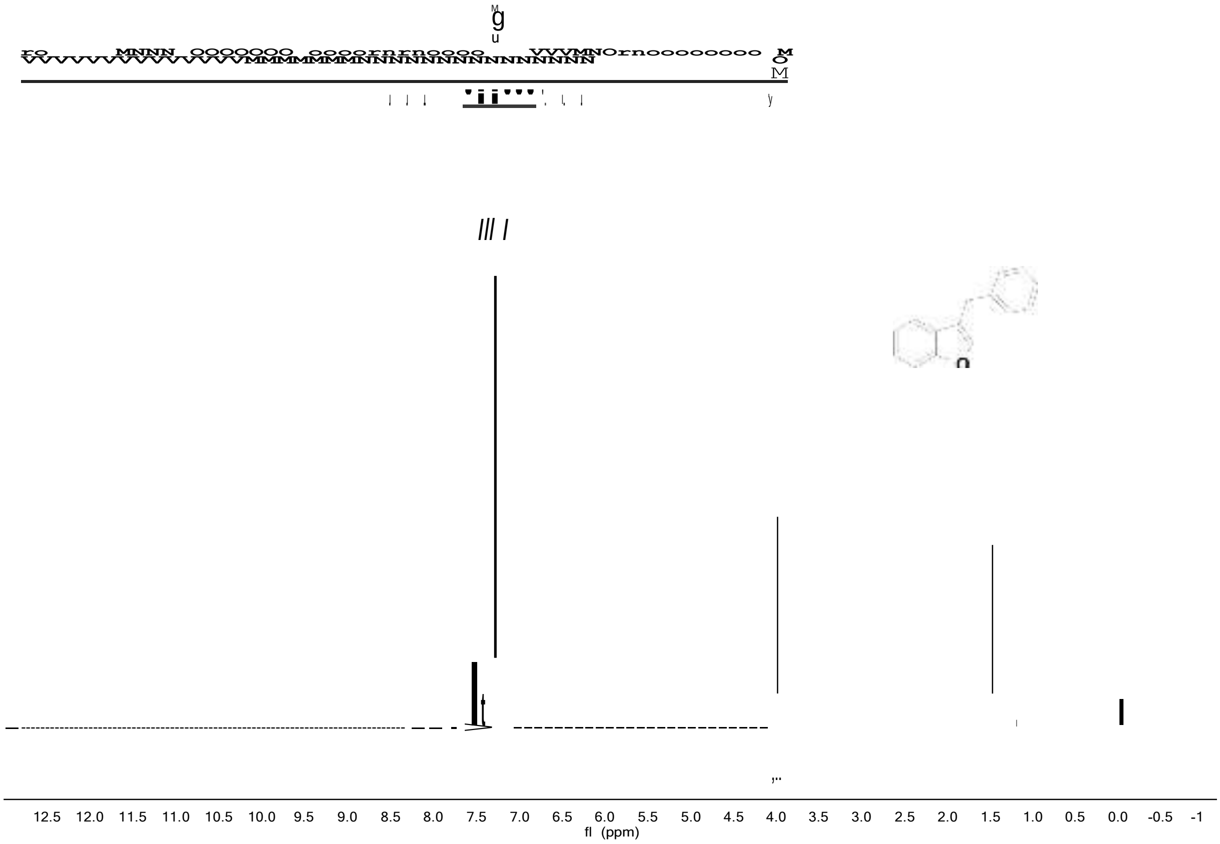

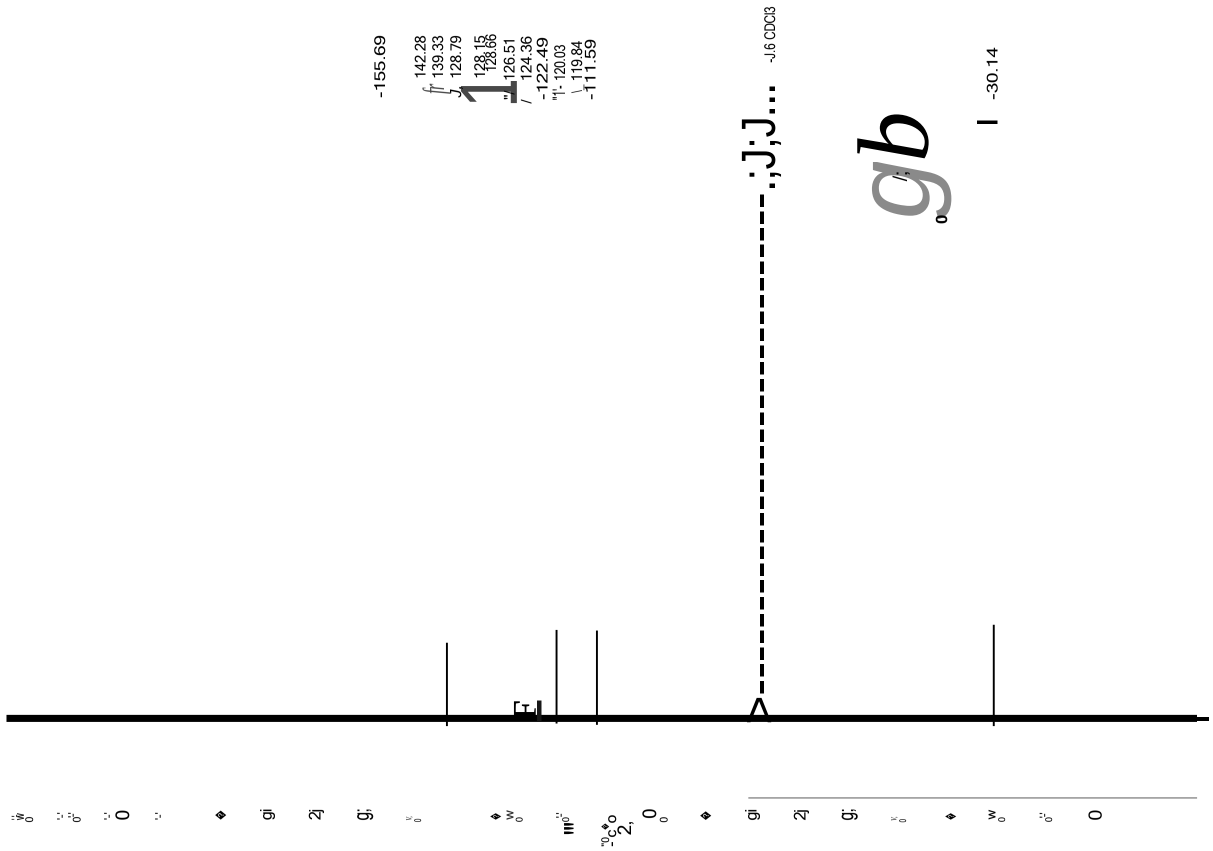

3-Phenylbenzofuran (**1b**)

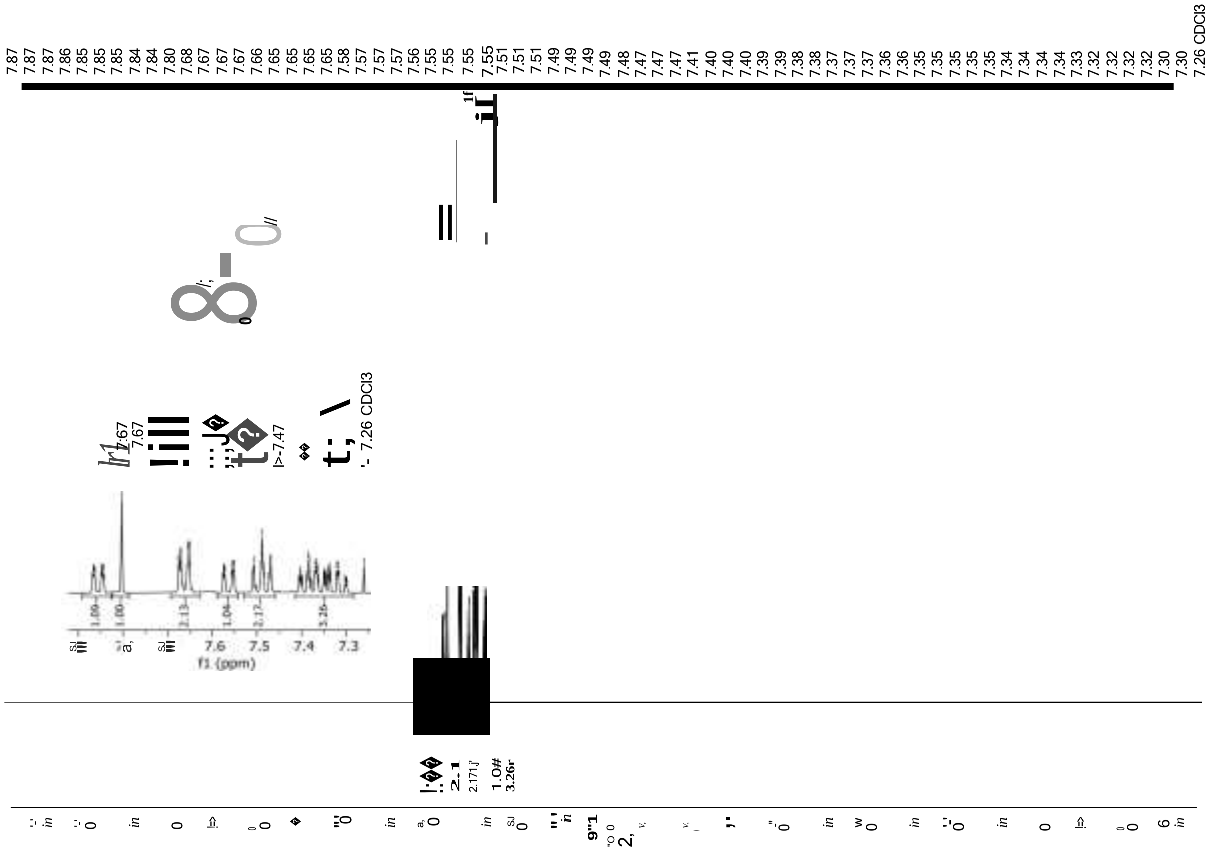

*6-Methoxy-3-methylbenzofuran (**1d**)*

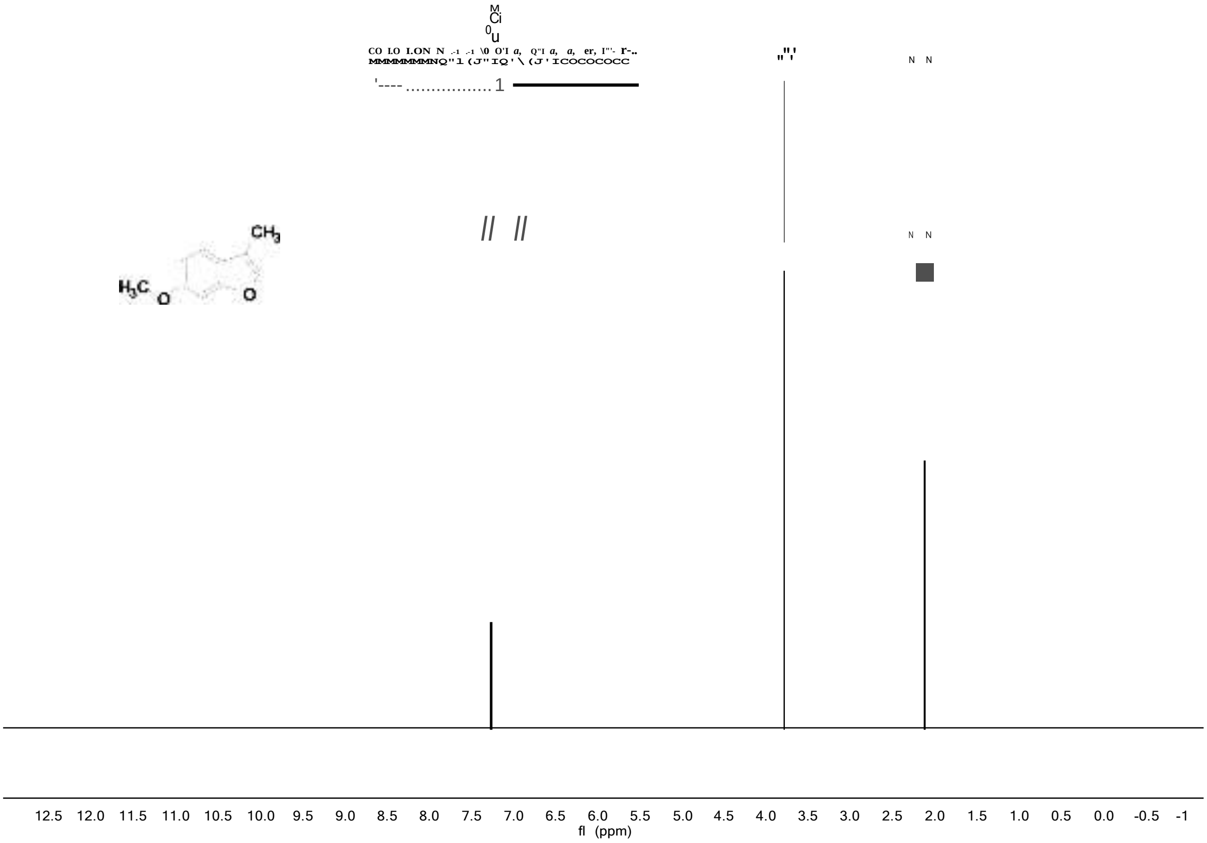

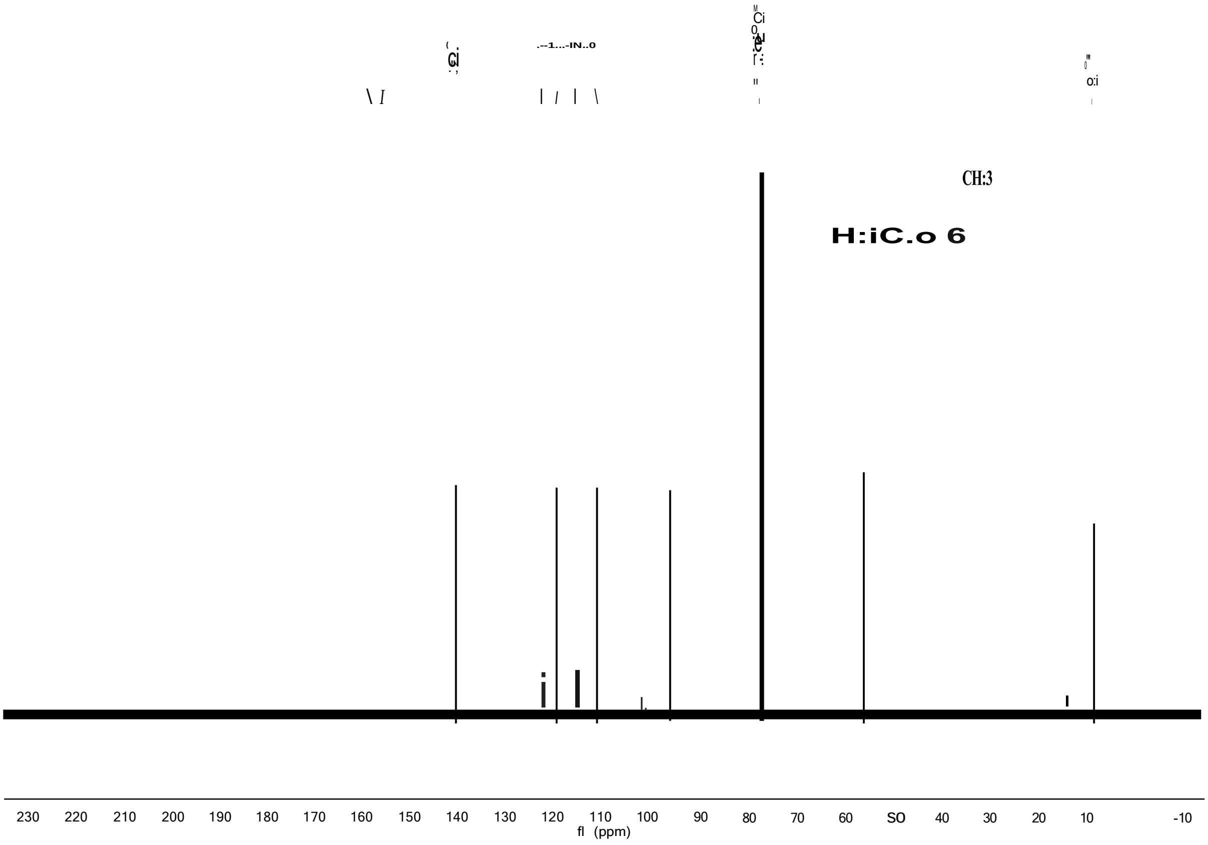

Methyl 3-methylbenzofuran-5-carboxylate (***1e***)

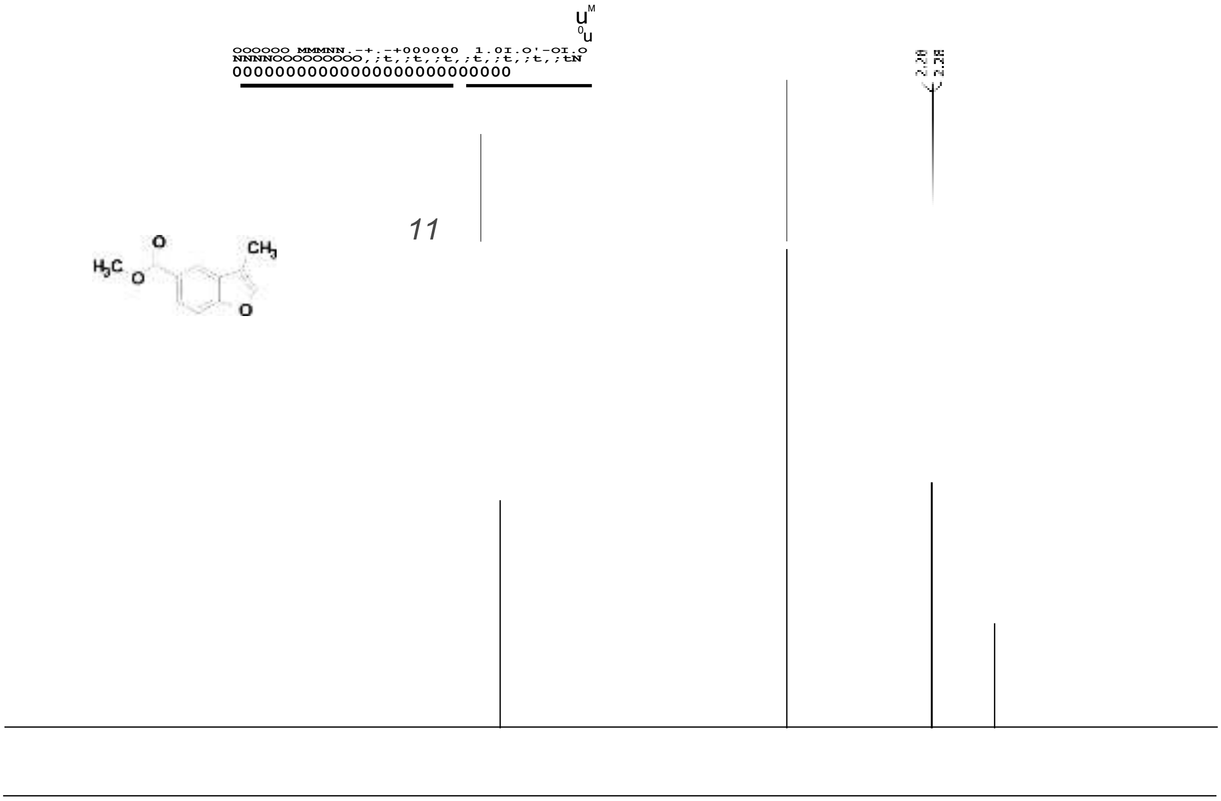

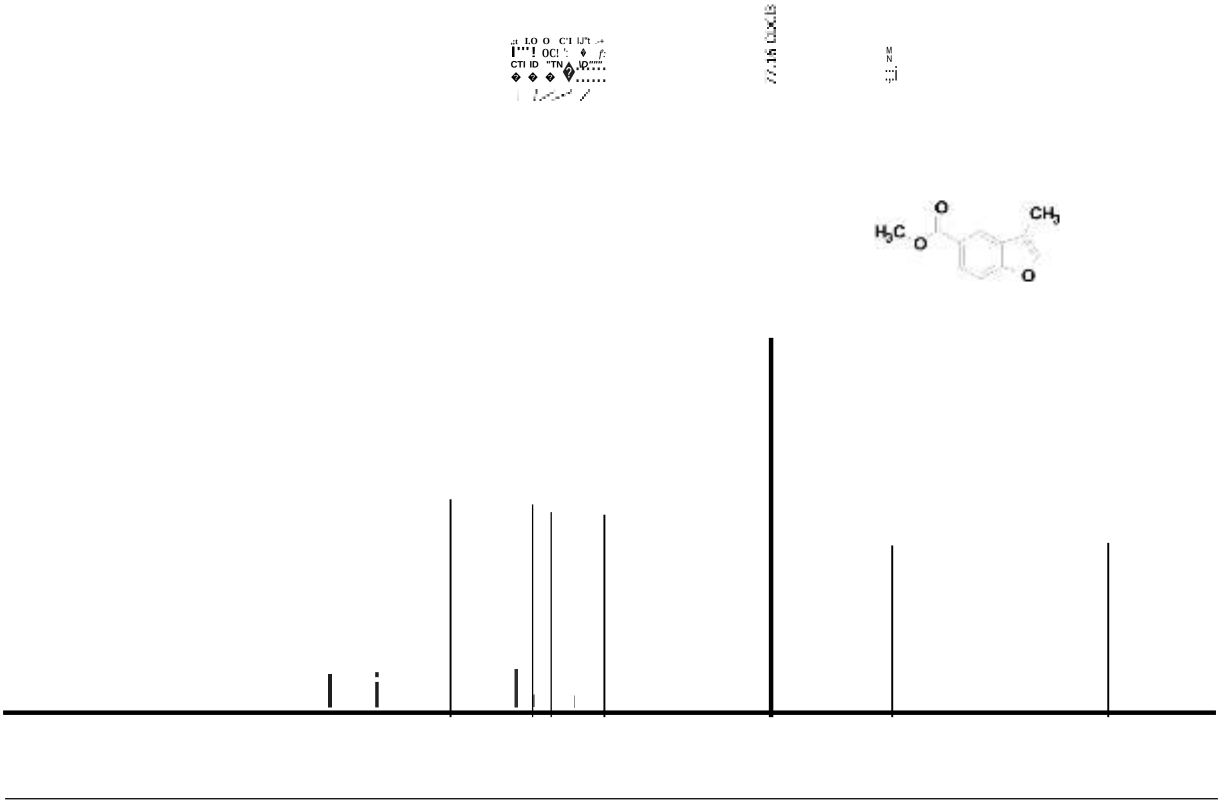

*Methyl 3-methylbenzofuran-6-carboxylate (**1f**)*

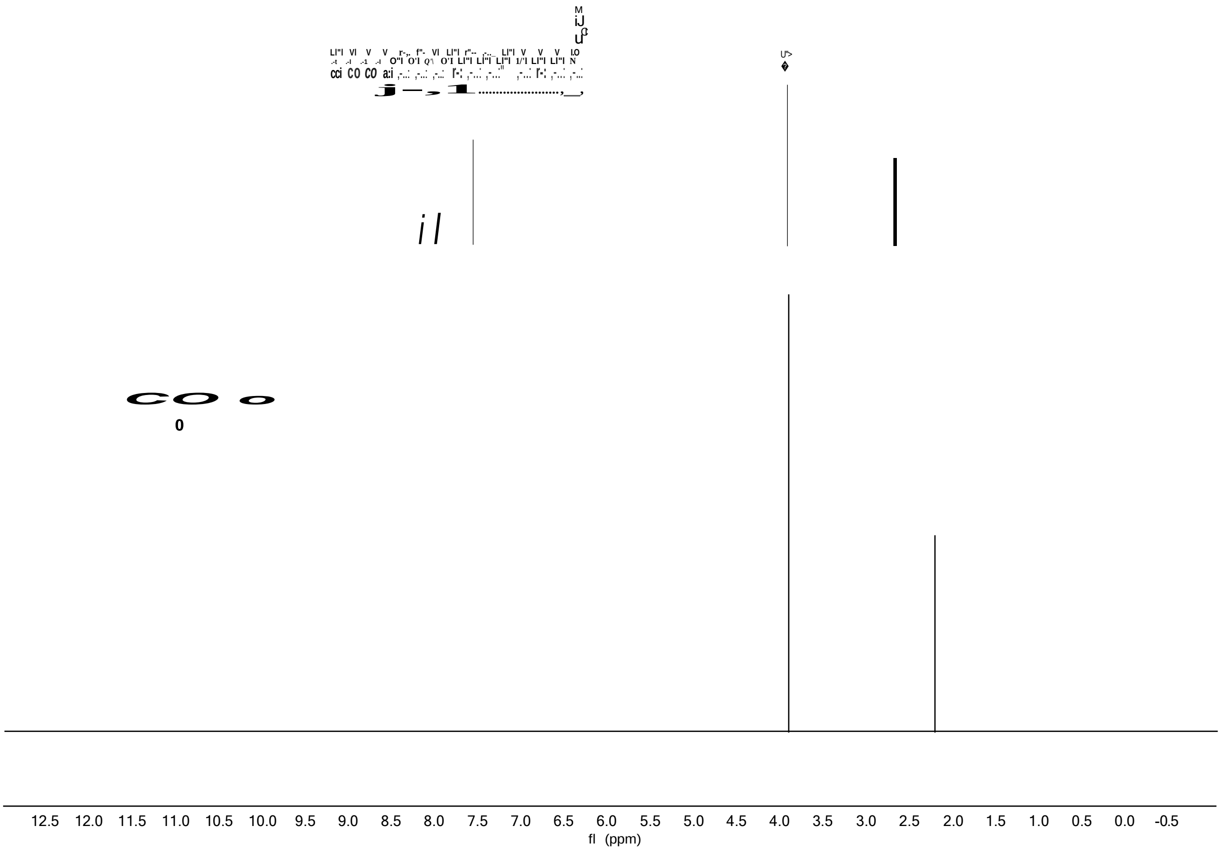

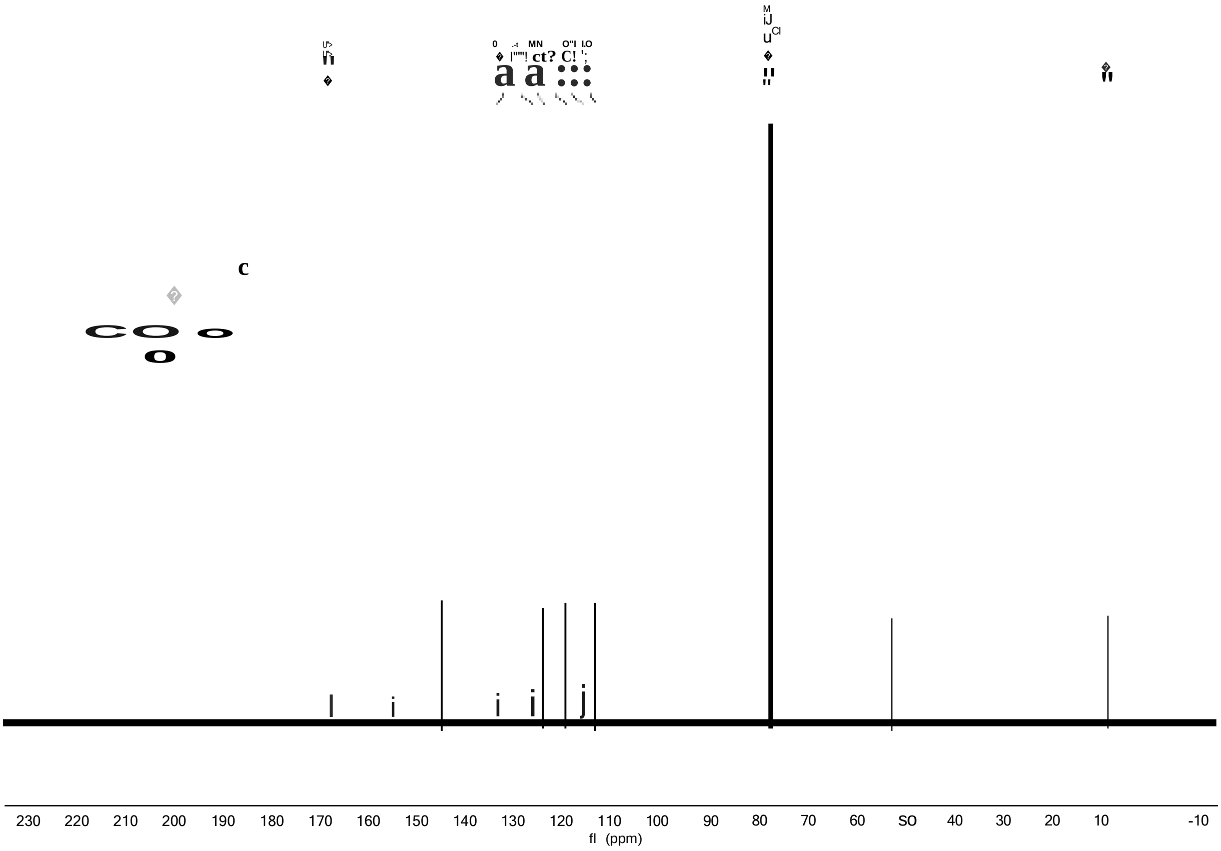

3-Methyl-5-phenylbenzofuran (***1g***)

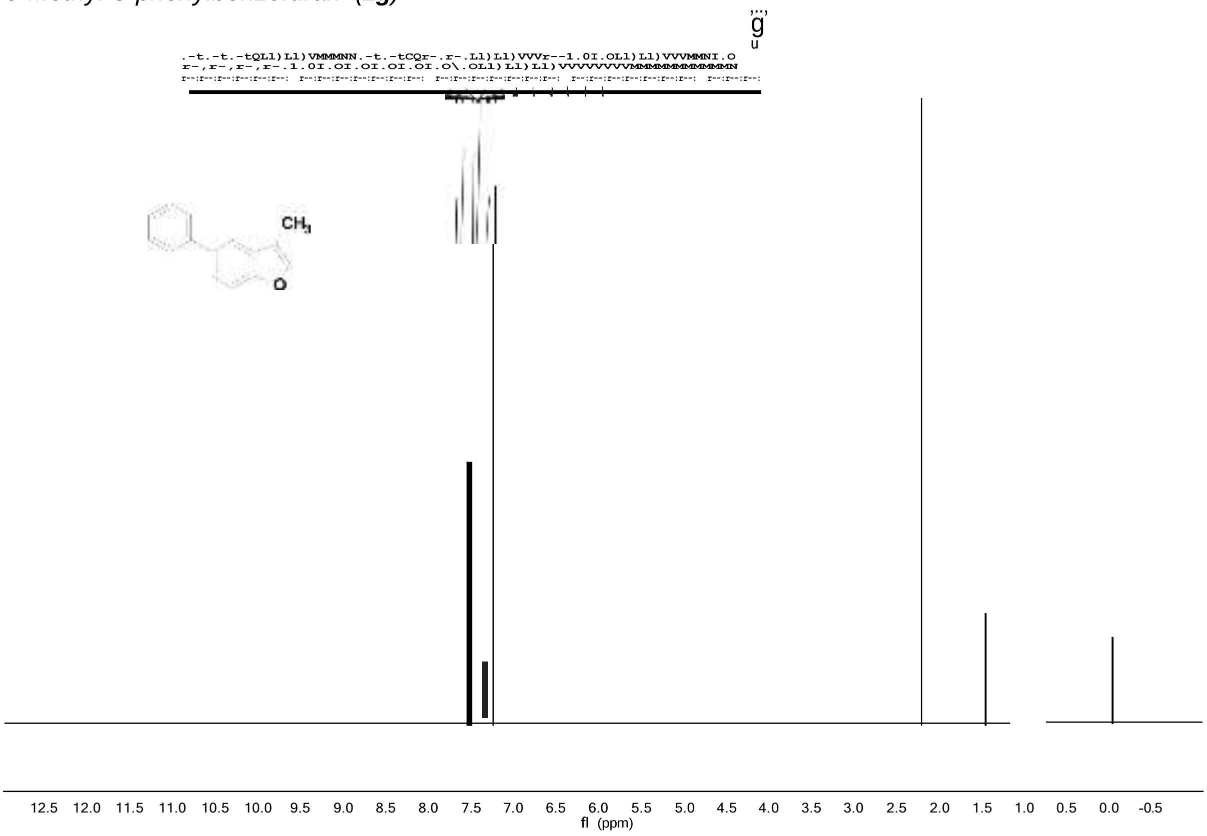

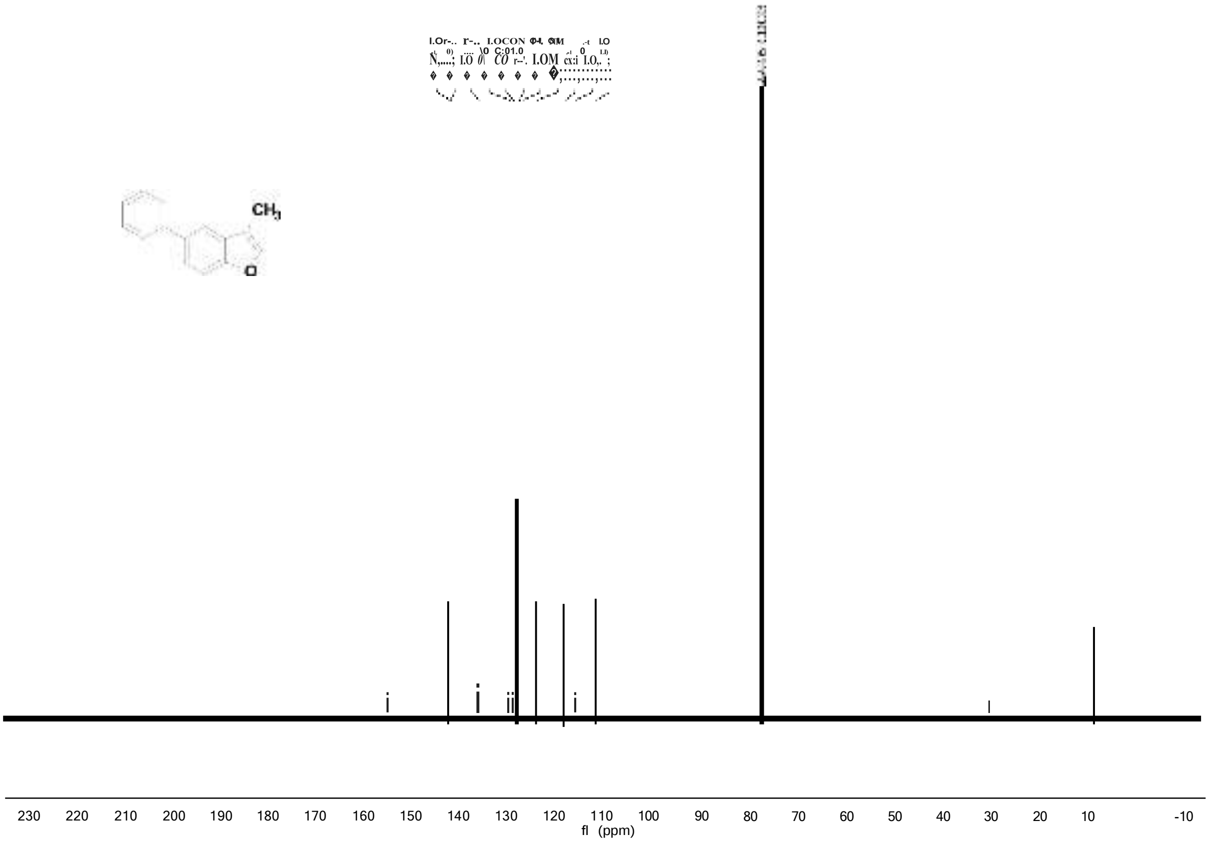

*3-Methyl-6-phenylbenzofuran (**1h**)*

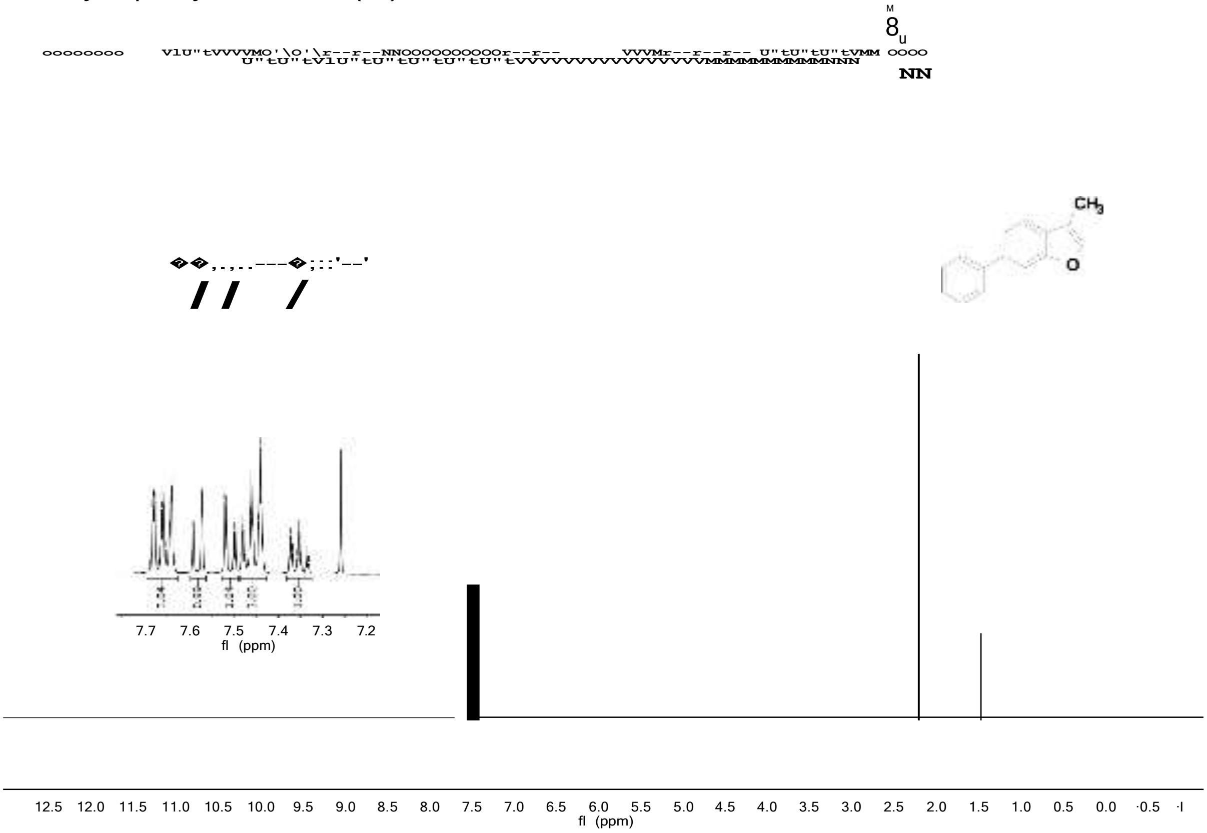

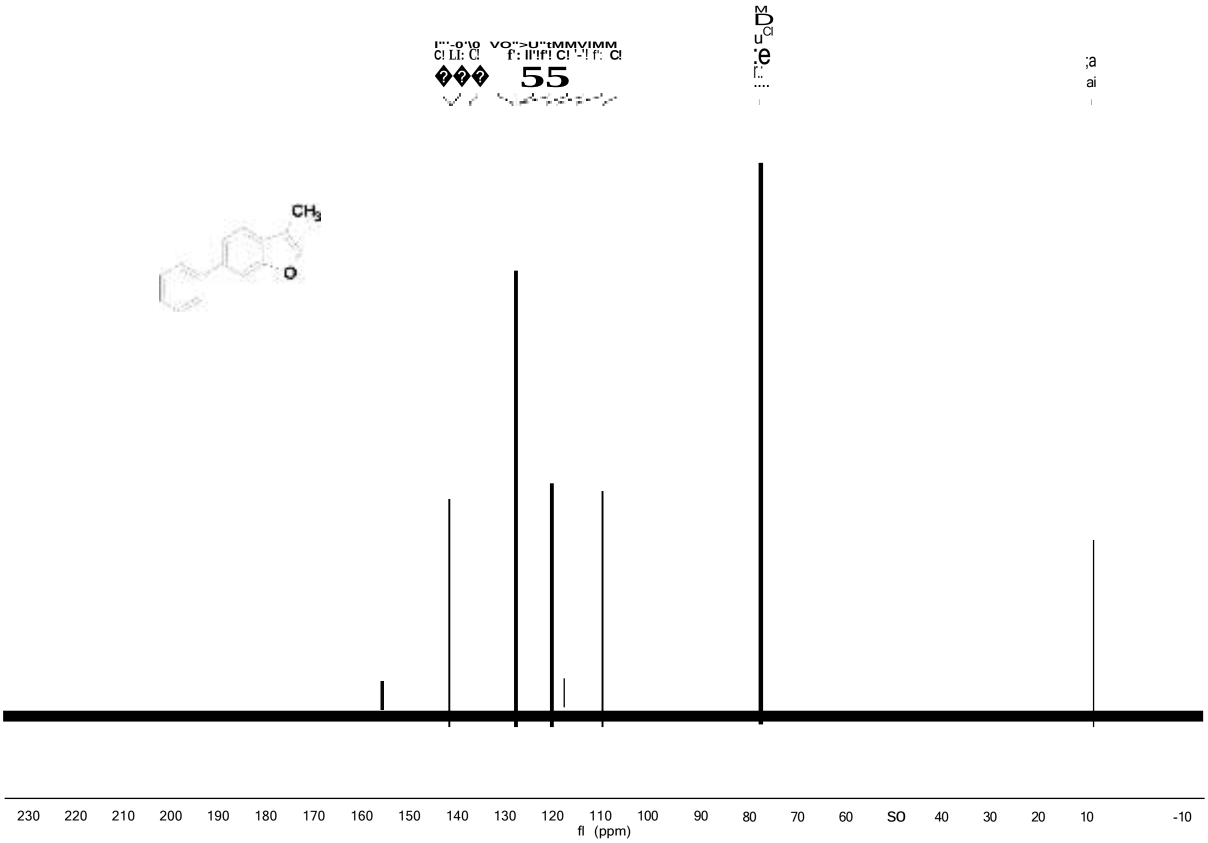

*6-(3-Chlorophenyl)-3-methylbenzofuran (**1i**)*

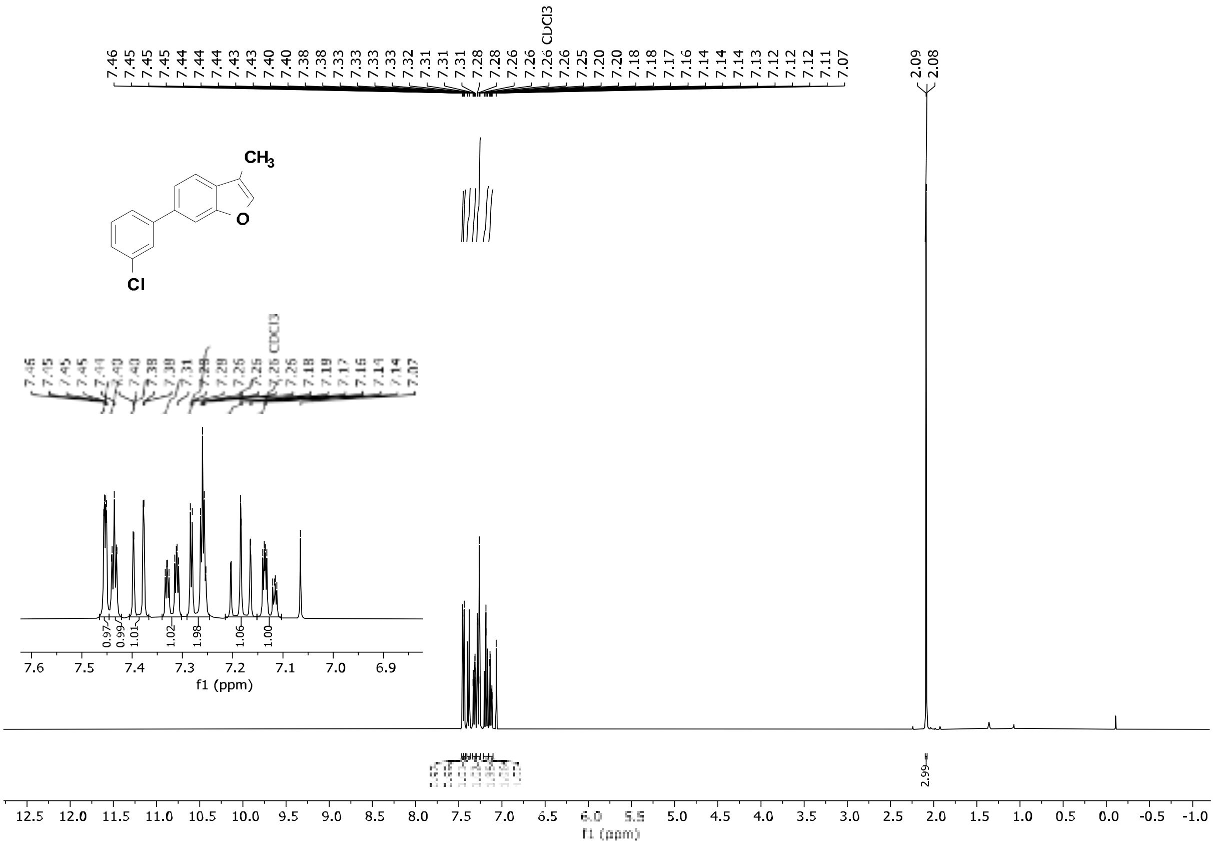

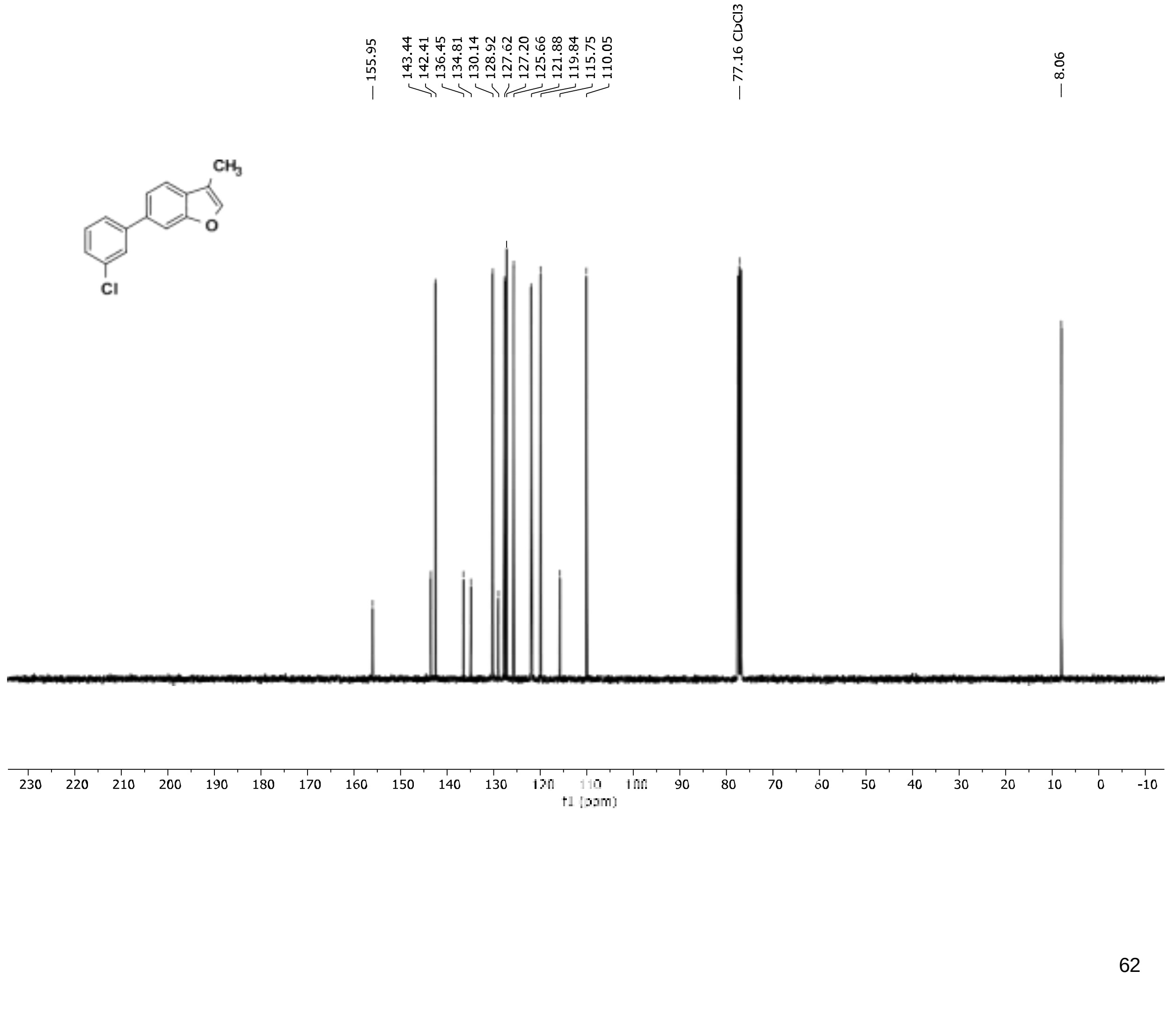

6-(4-chlorophenyl)-3-methylbenzofuran (***1j***)

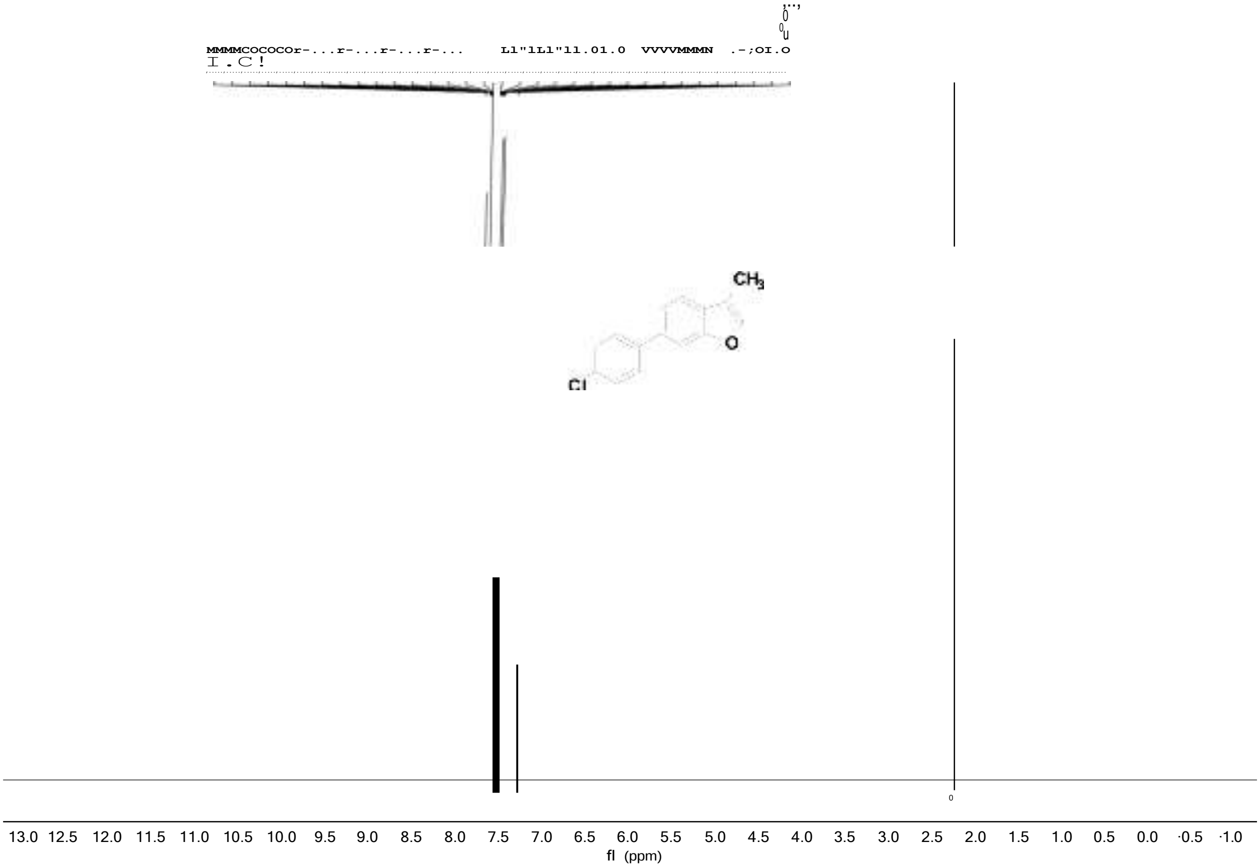

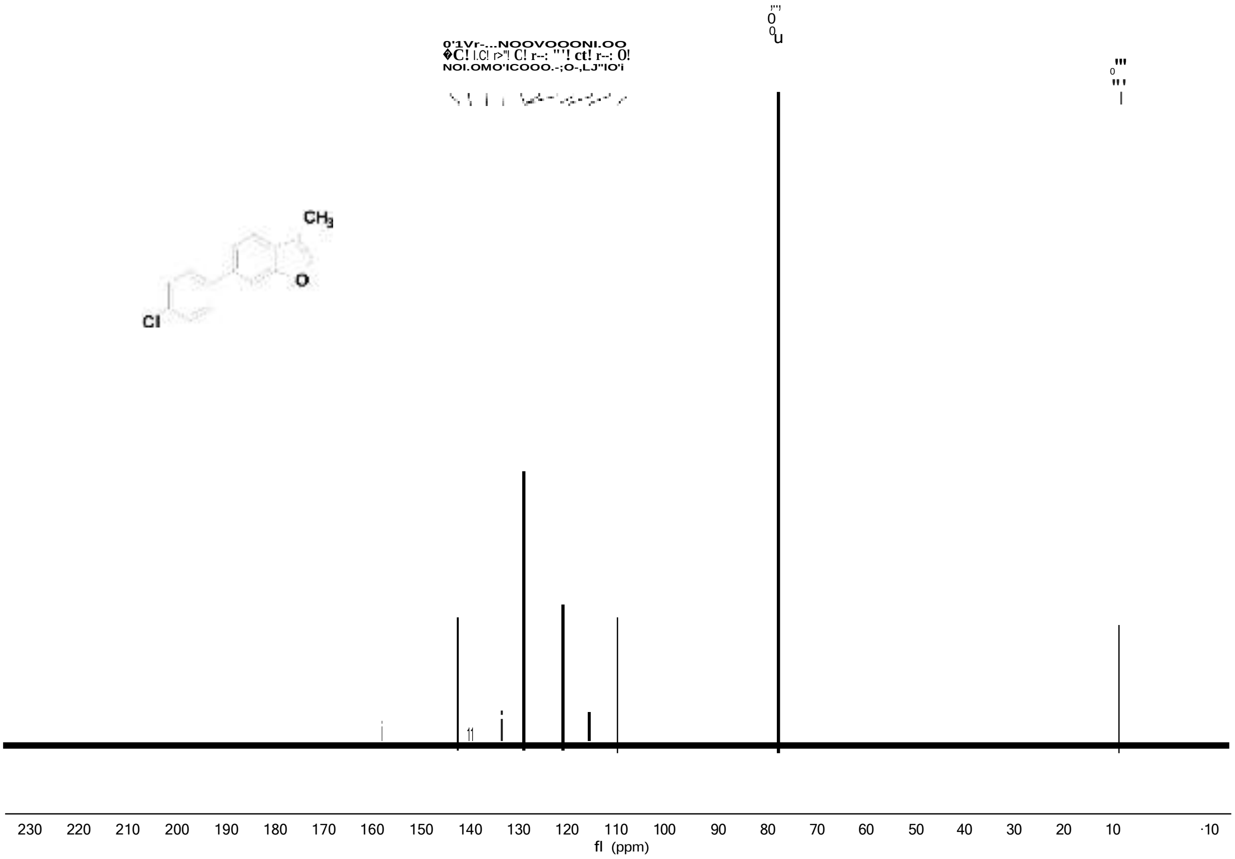

*3-methyl-6-(2-(trifluoromethyl)phenyl)benzofuran (**1k**)*

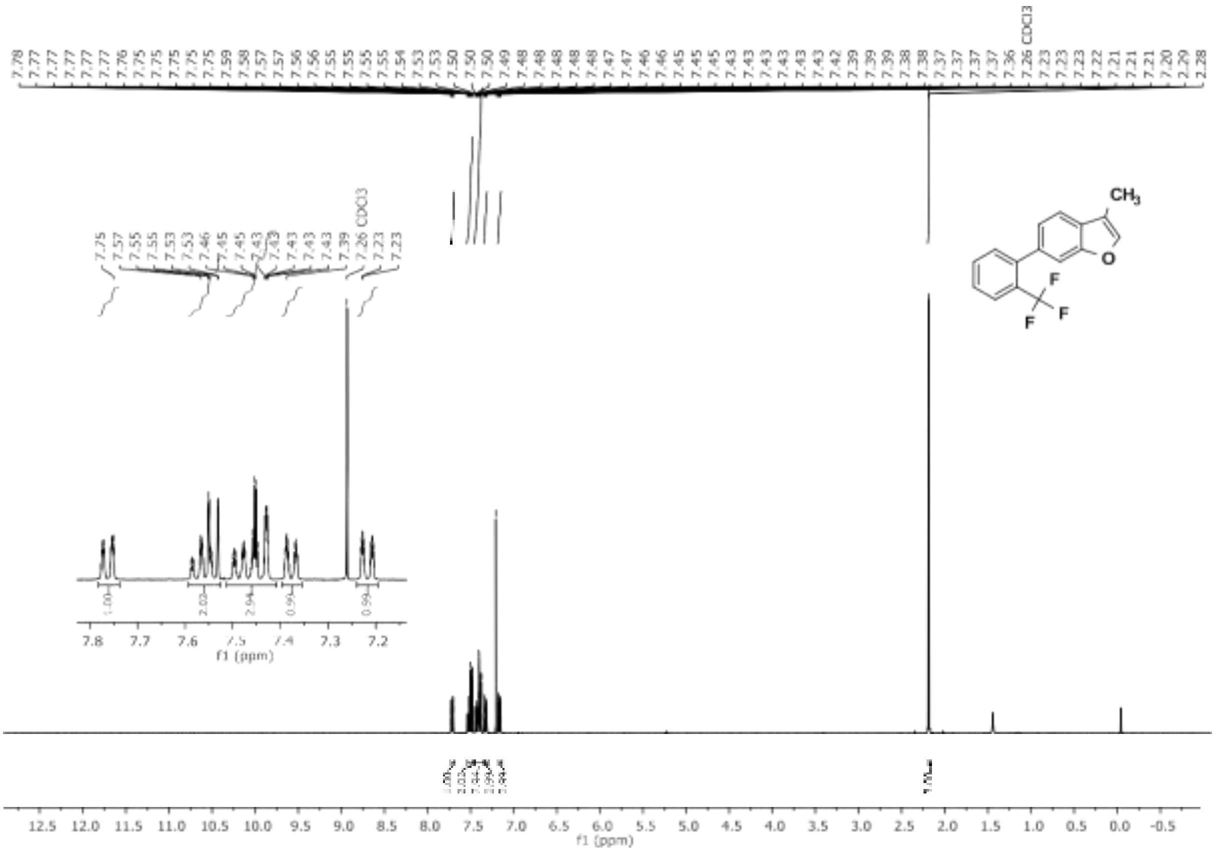

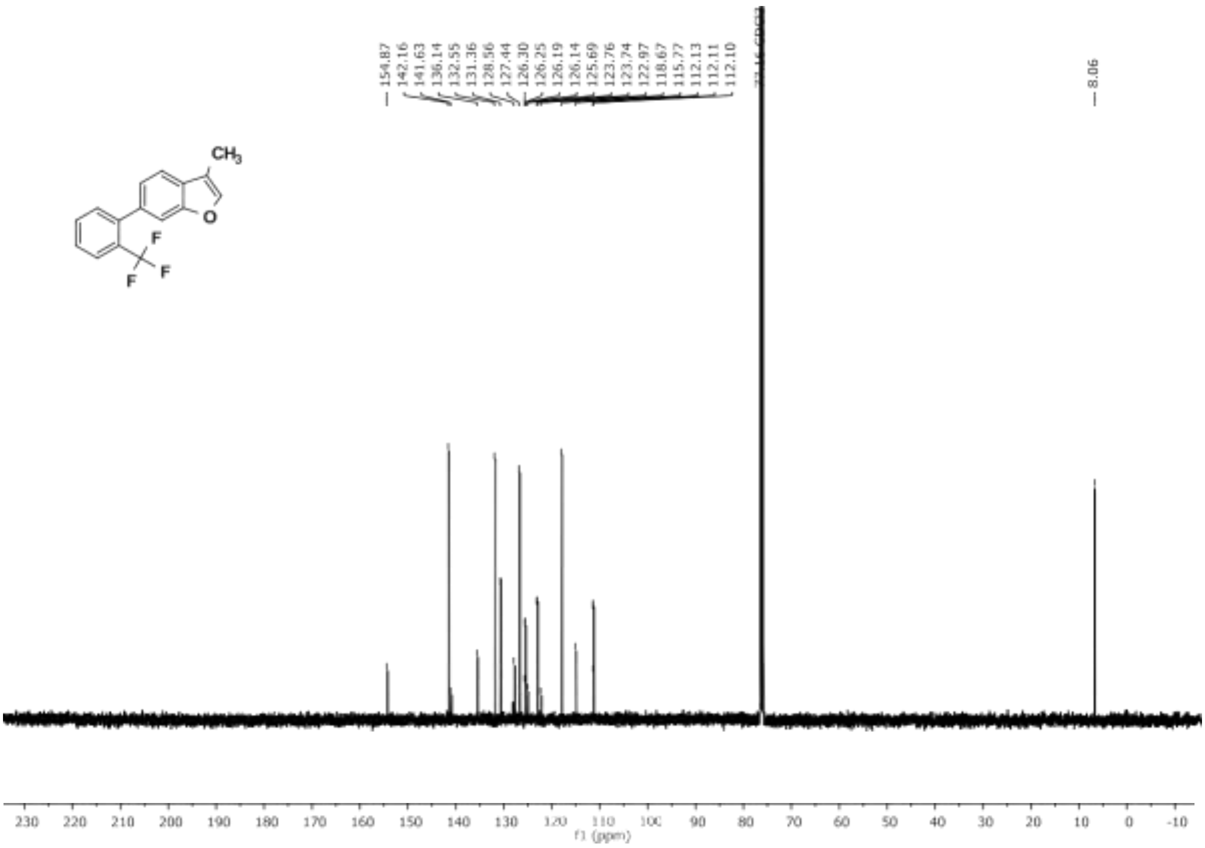

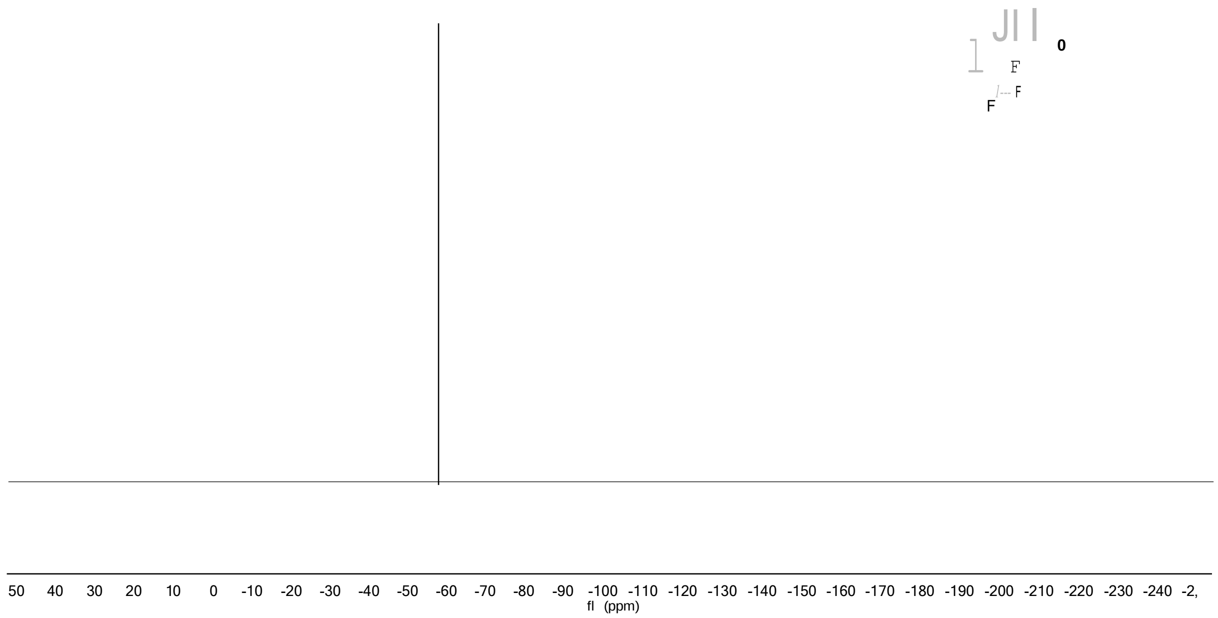

*3-methyl-6-(phenylethynyl)benzofuran (**1l**)*

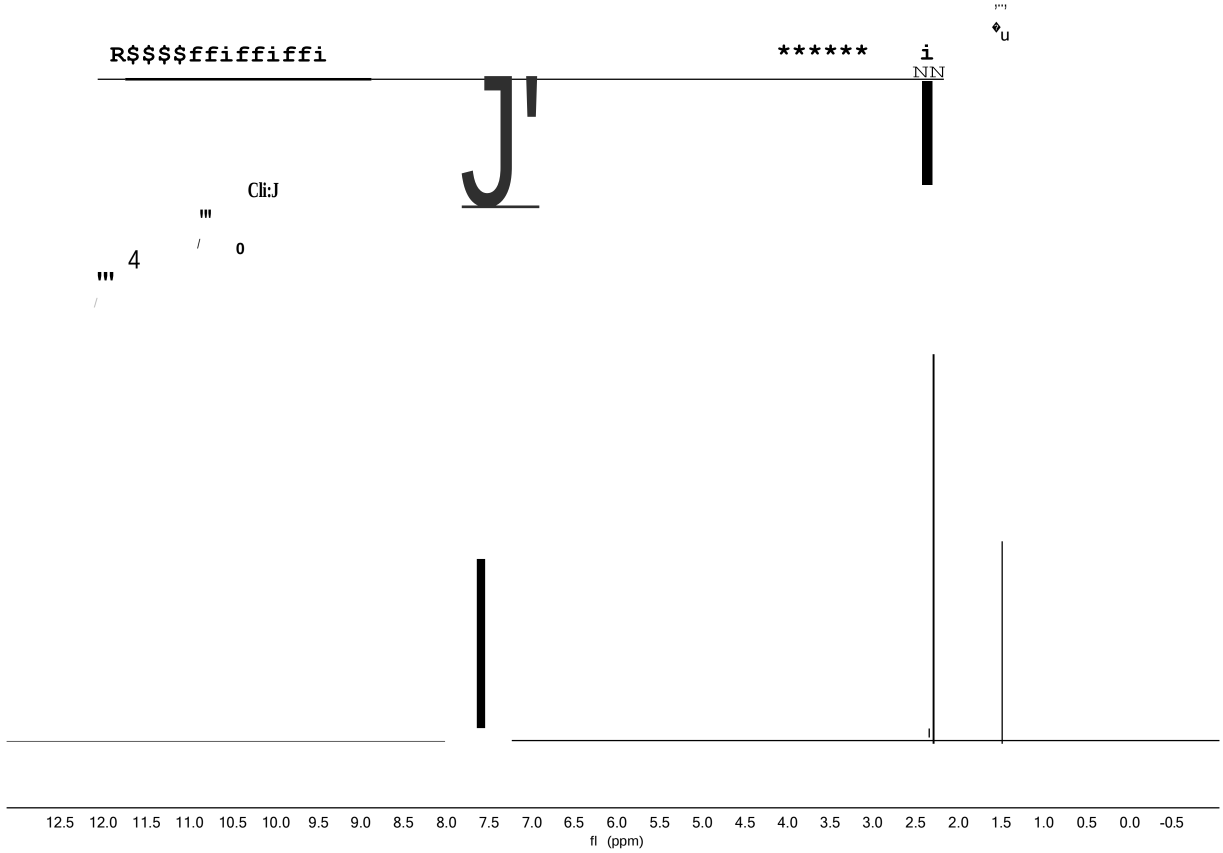

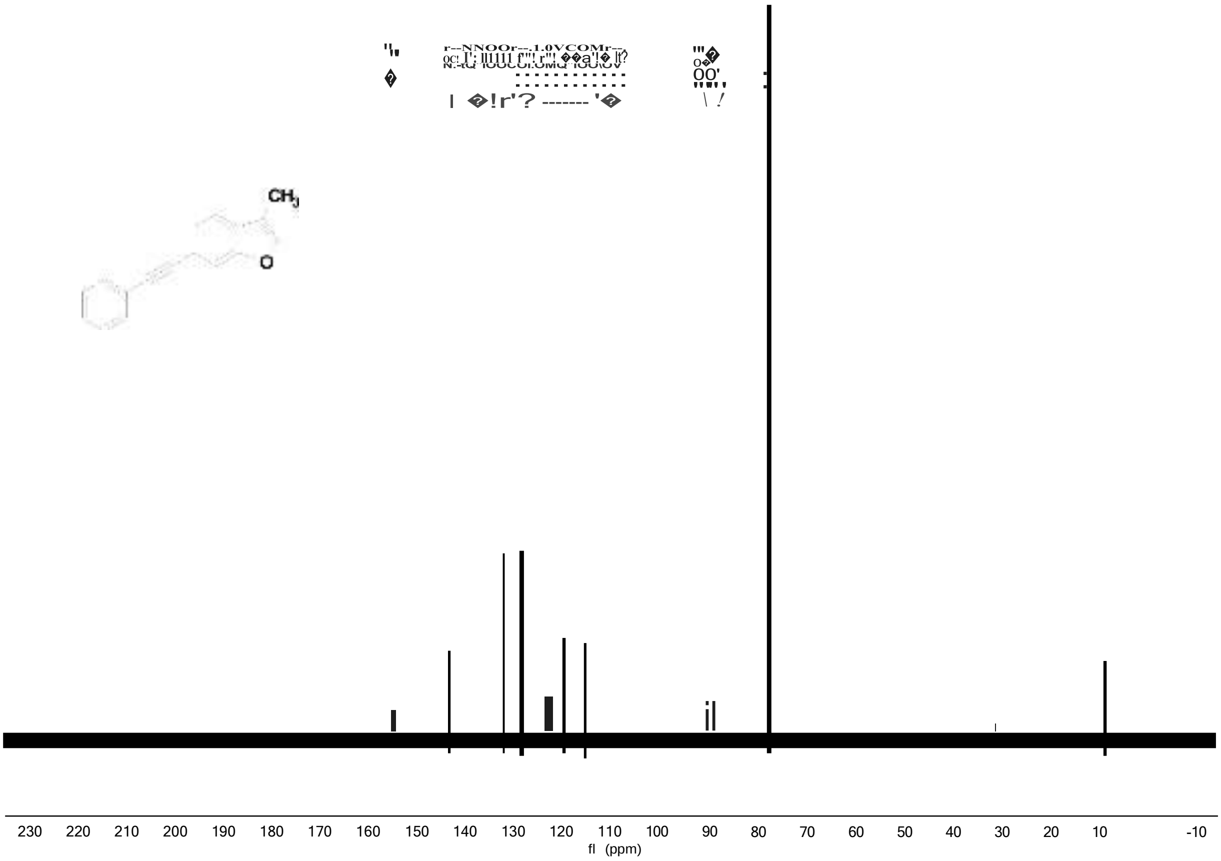

*3-(3-Methylbenzofuran-5-yl)pyridine (**1m**)*

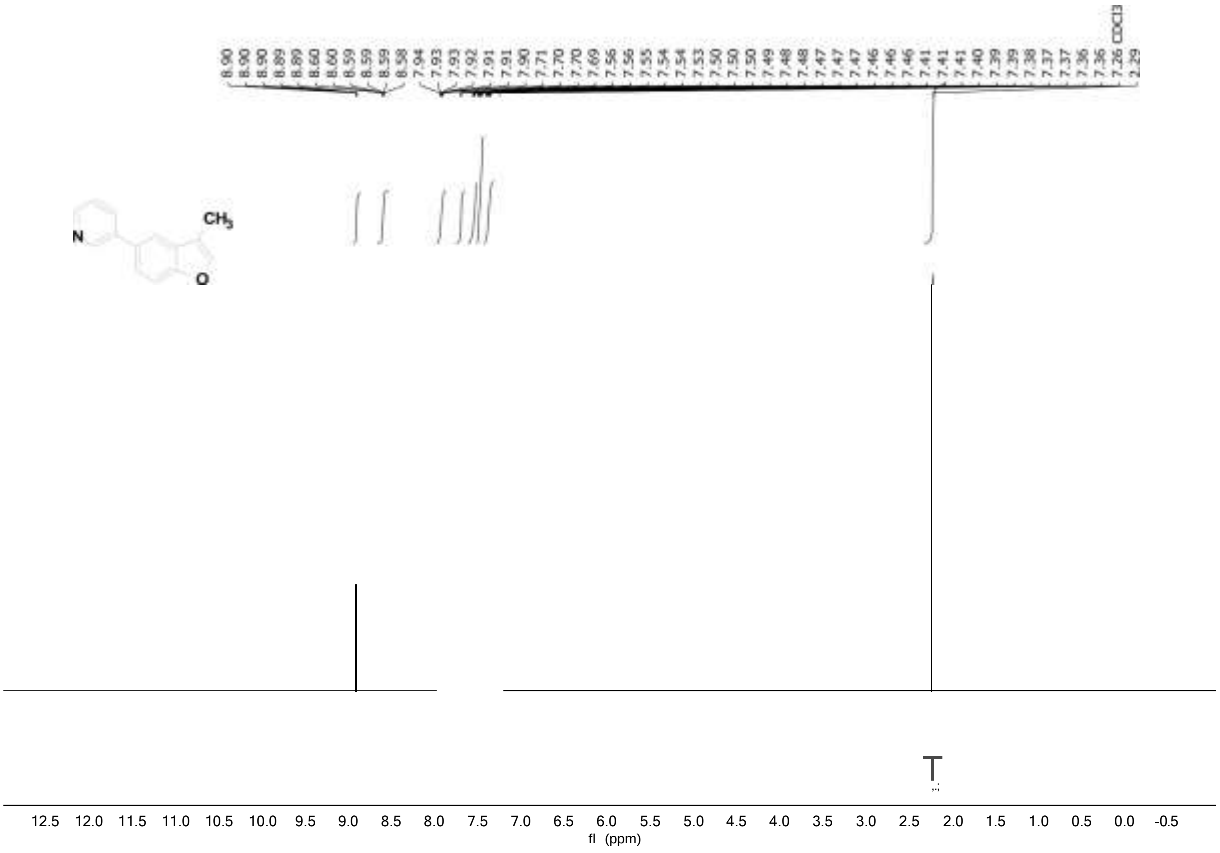

3-(3-Methylbenzofuran-6-yl)pyridine (**1n**)

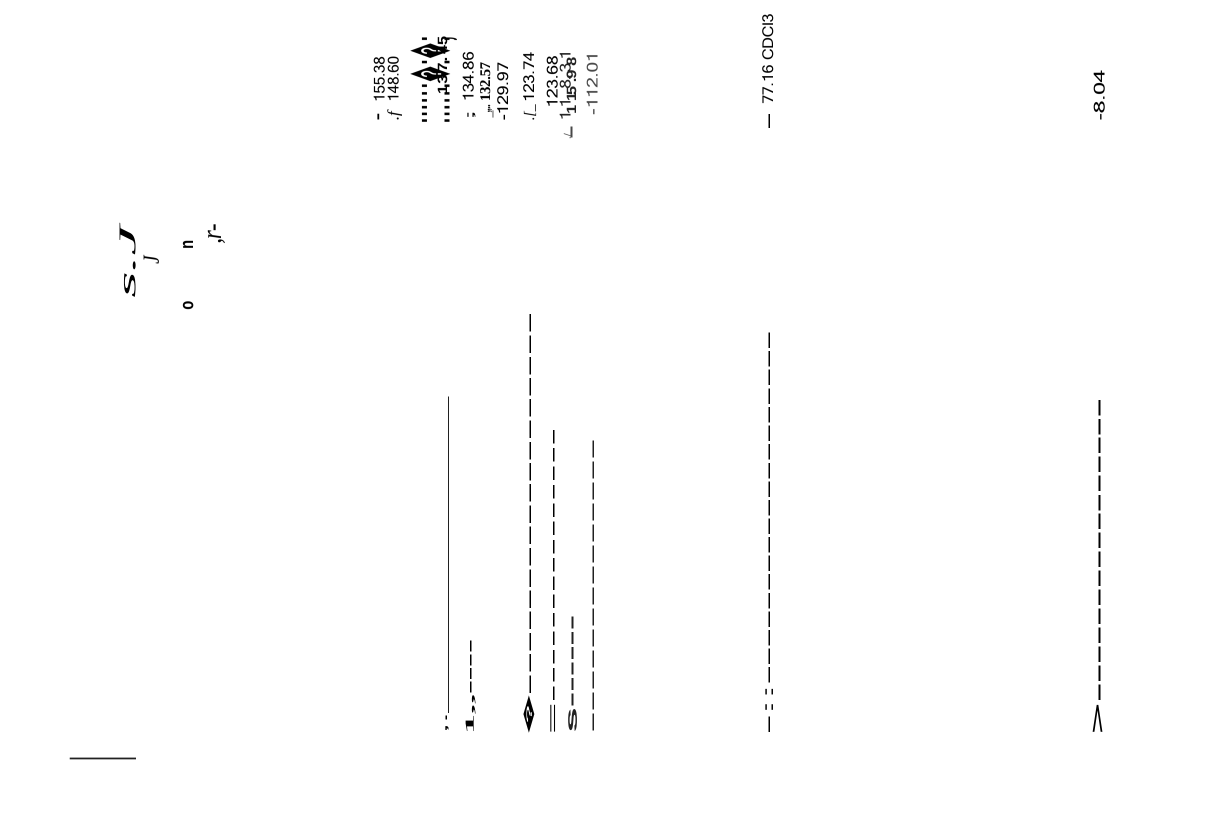

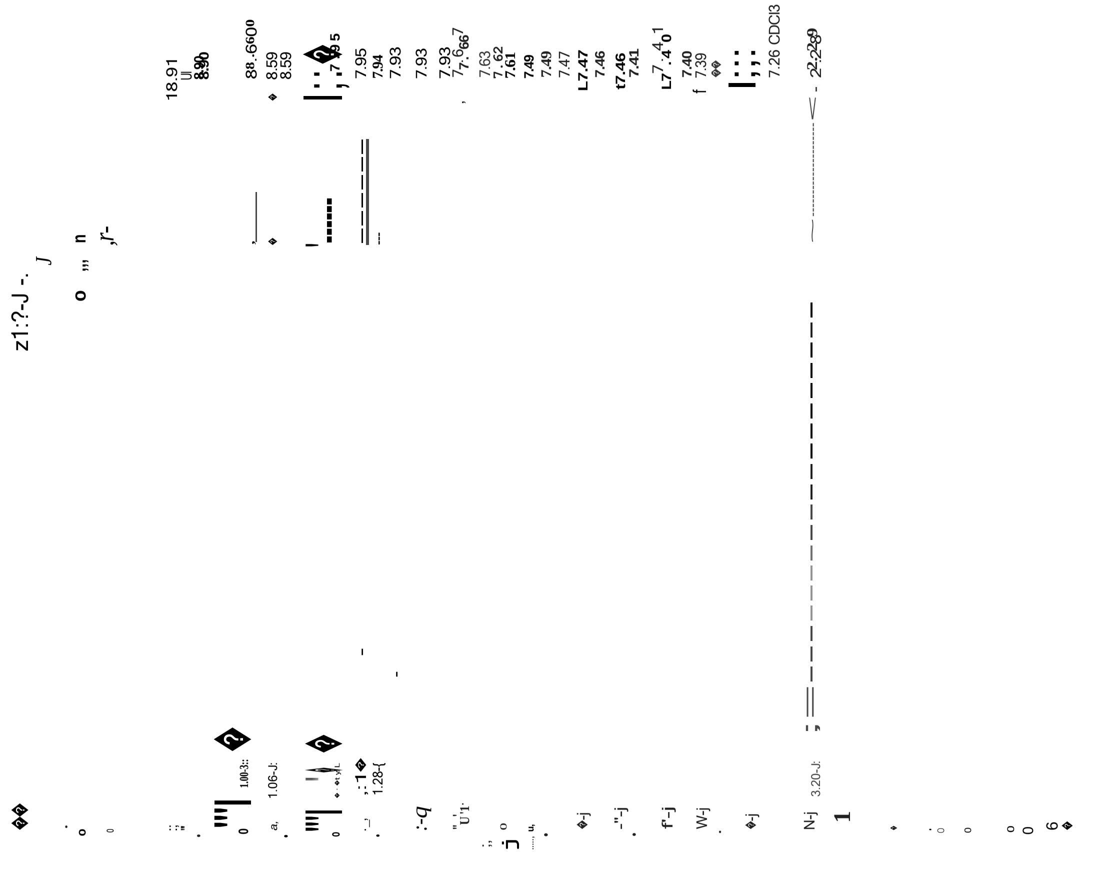

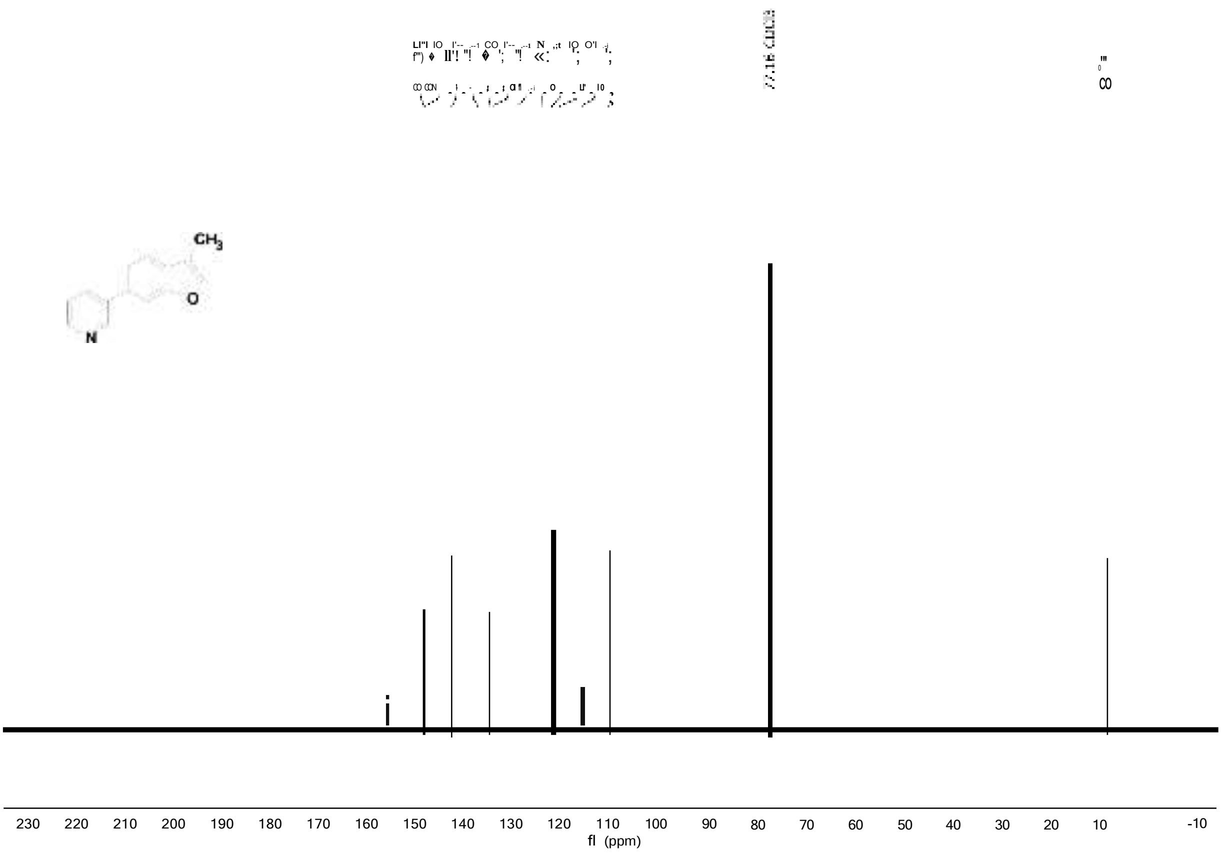

*3-Methyl-5-vinylbenzofuran (**1o**)*

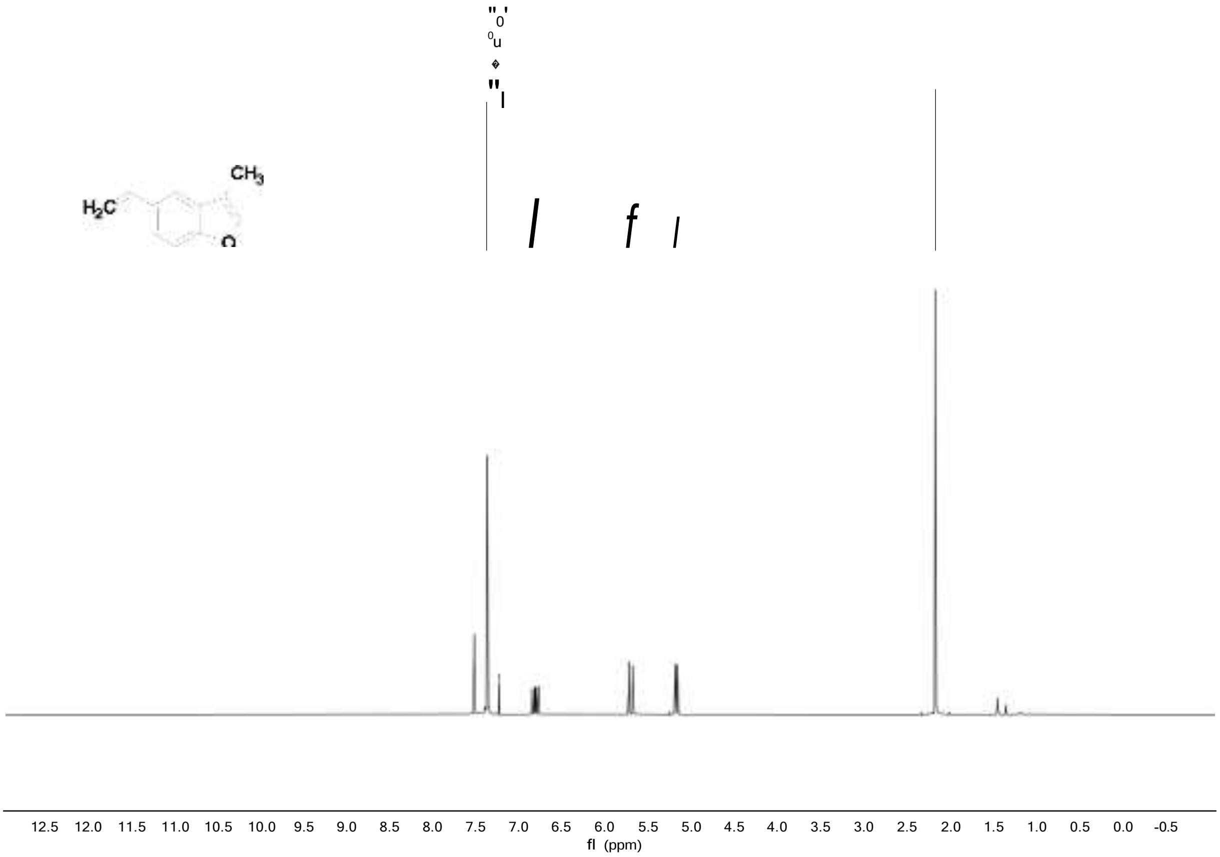

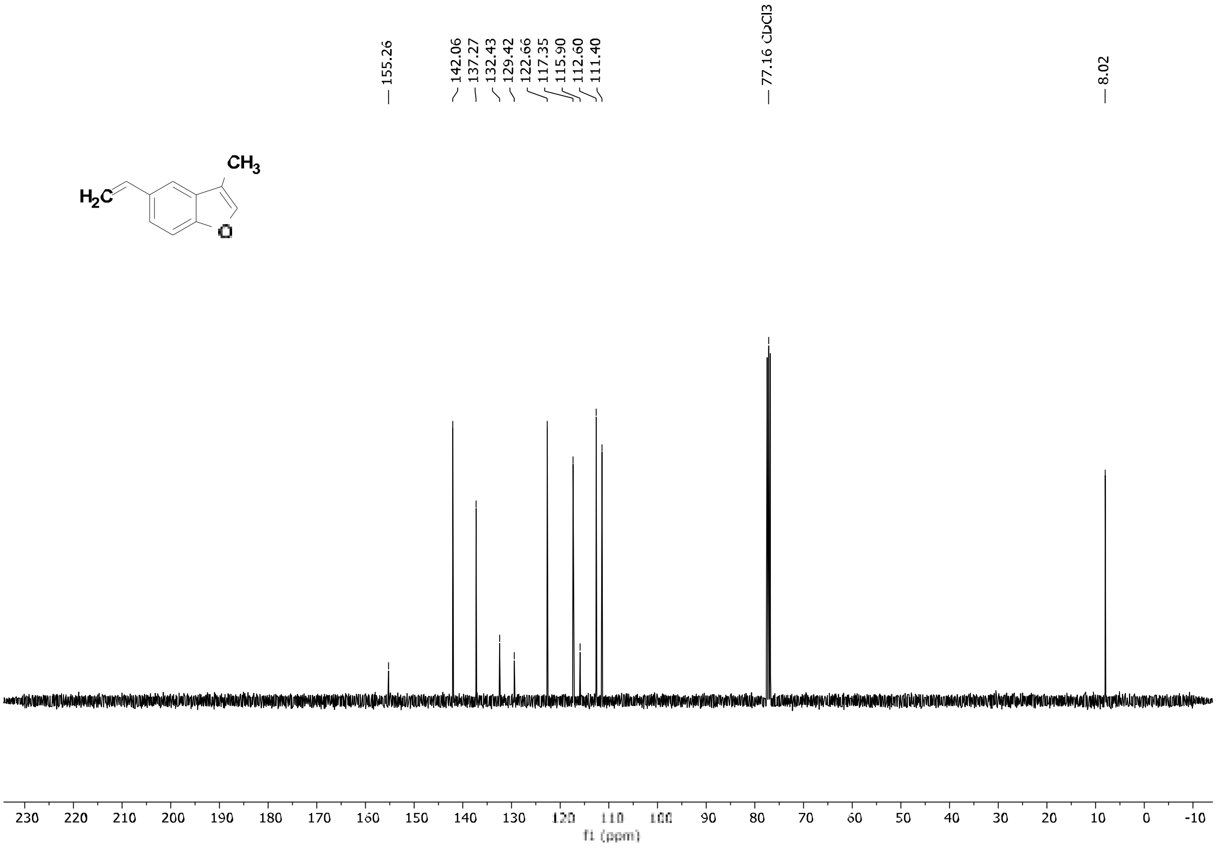

*3-Methyl-6-vinylbenzofuran (**1p**)*

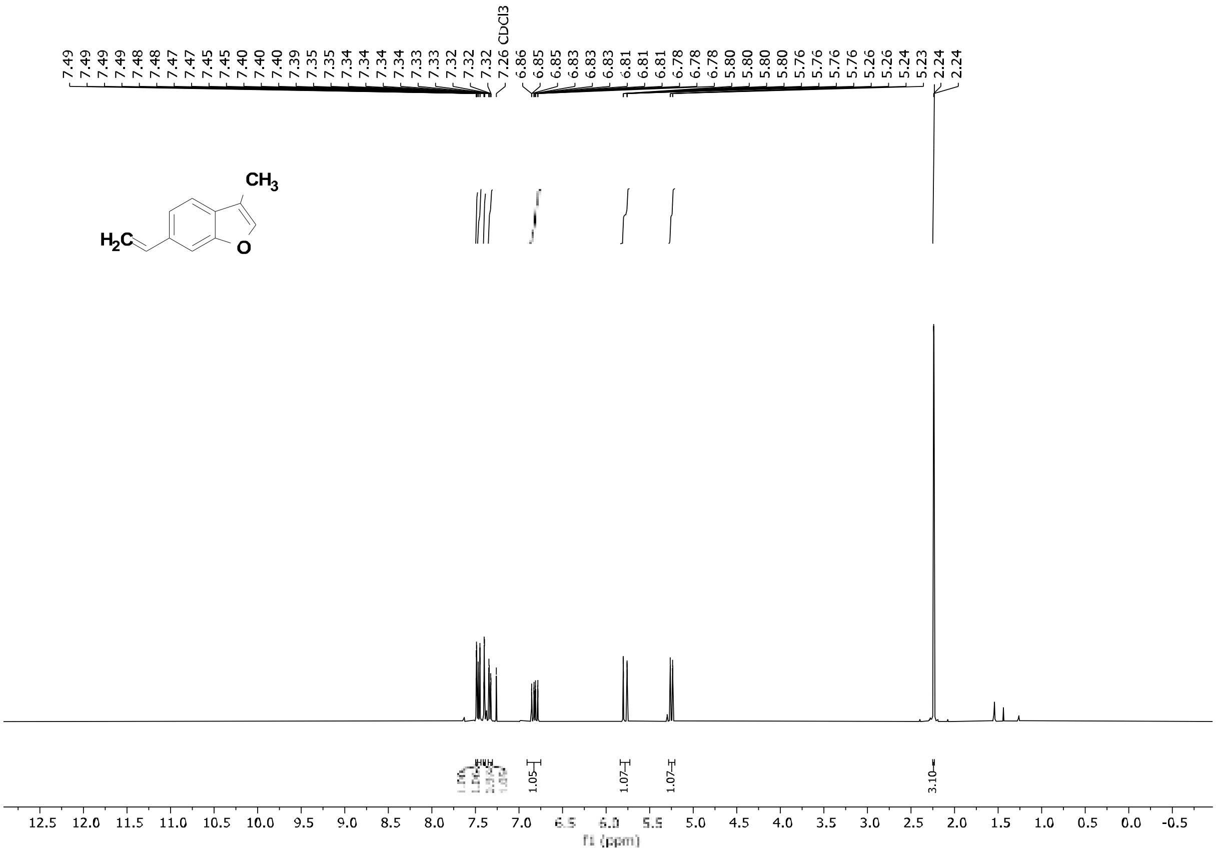

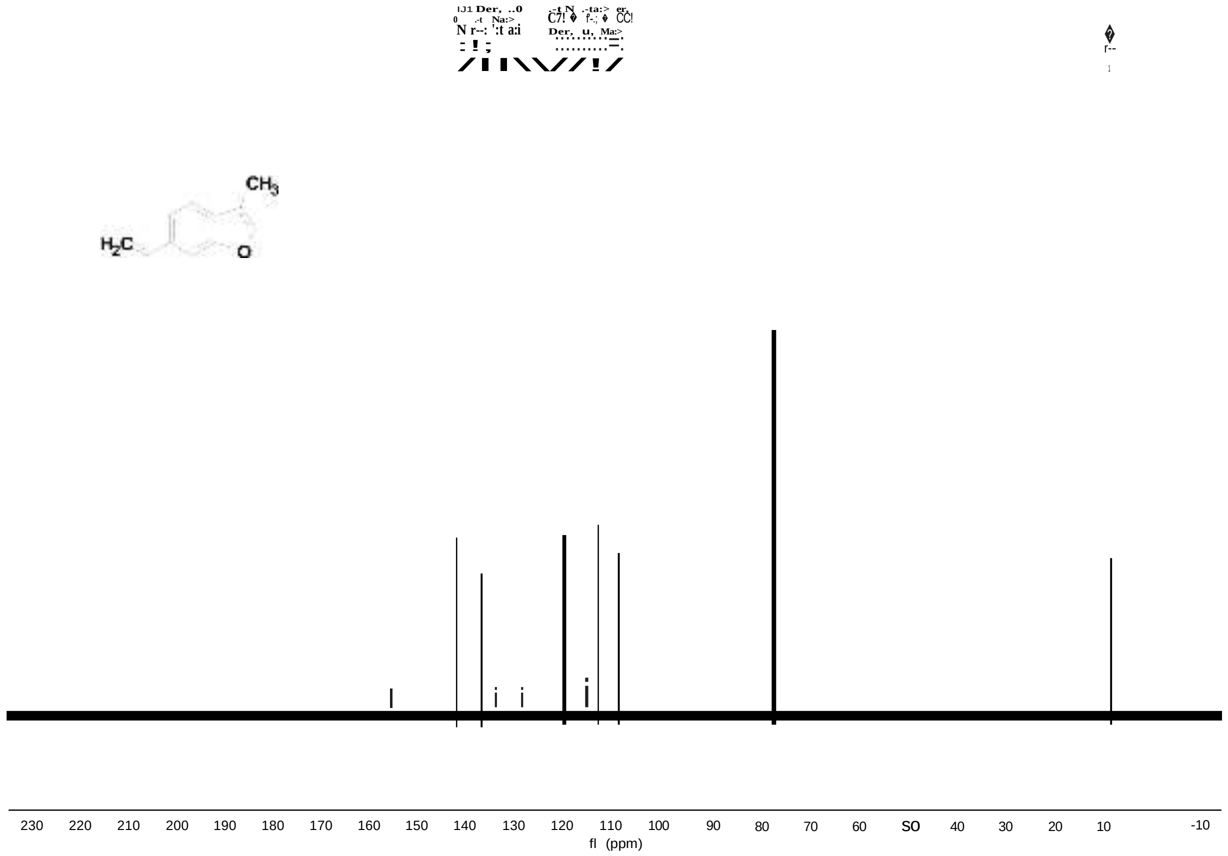
CH3
H2C
O
fl (ppm)

NMR Spectra of Products

NMR Spectra of Benzisoxazoles from Benzofurans

*Benzo[*d*]isoxazole (**5a**)*

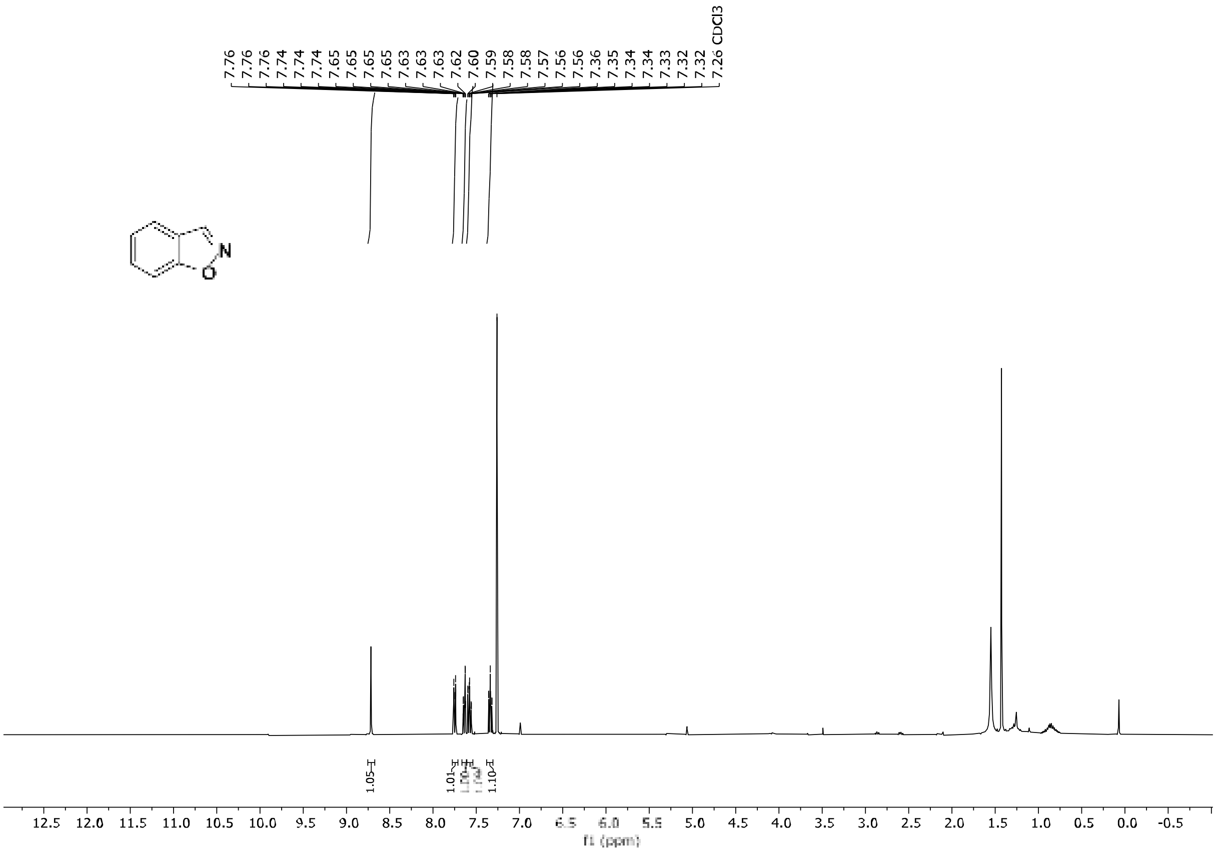

*5-Phenylbenzo[d]isoxazole (**5b**)*

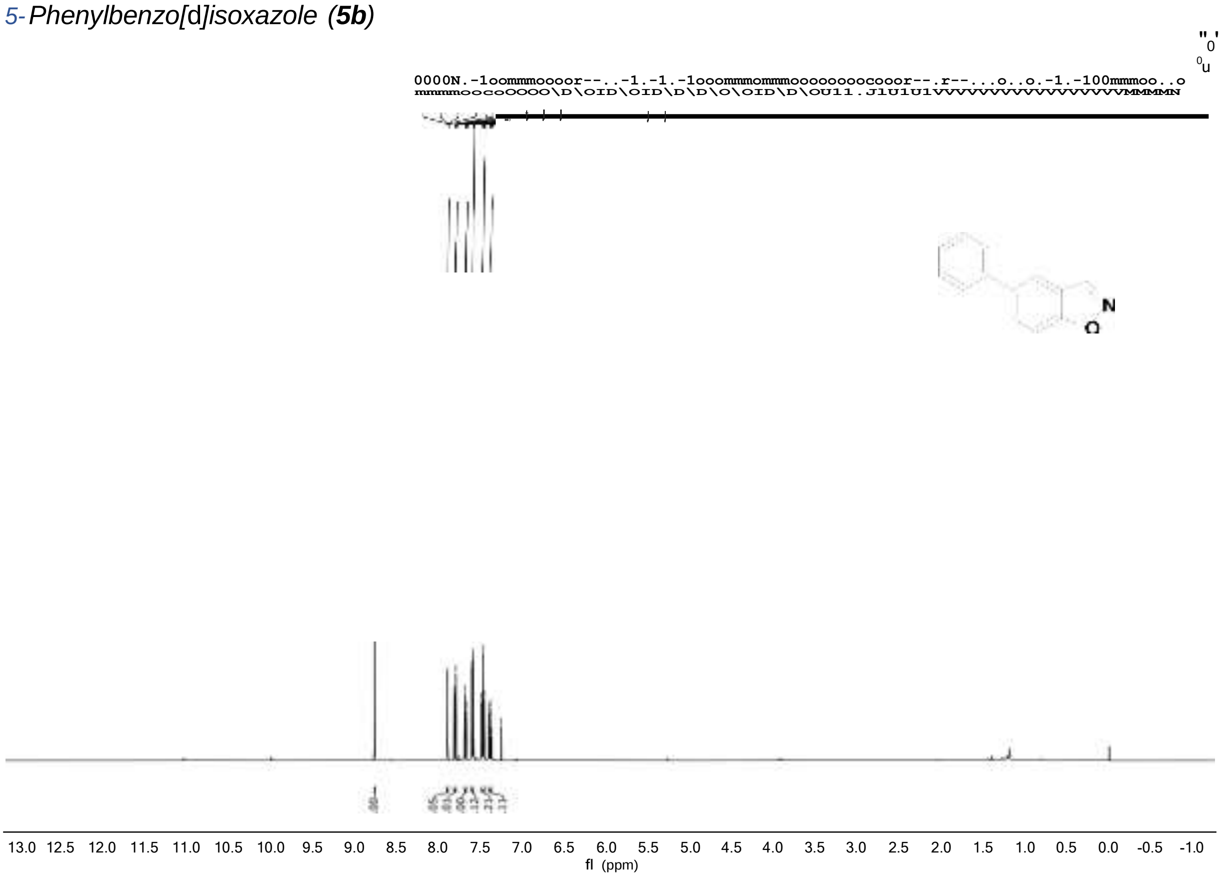

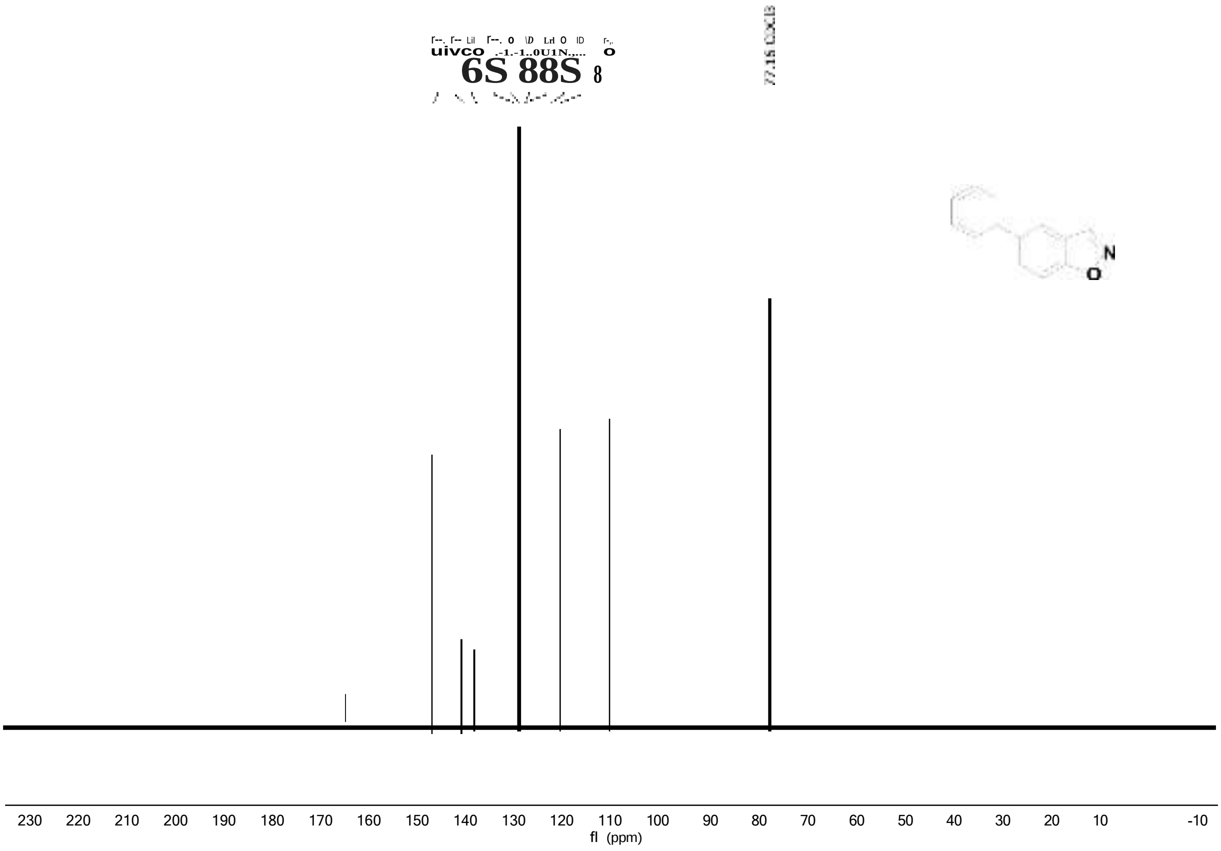

*Methyl benzo[*d*]isoxazole-5-carboxylate (**5c**)*

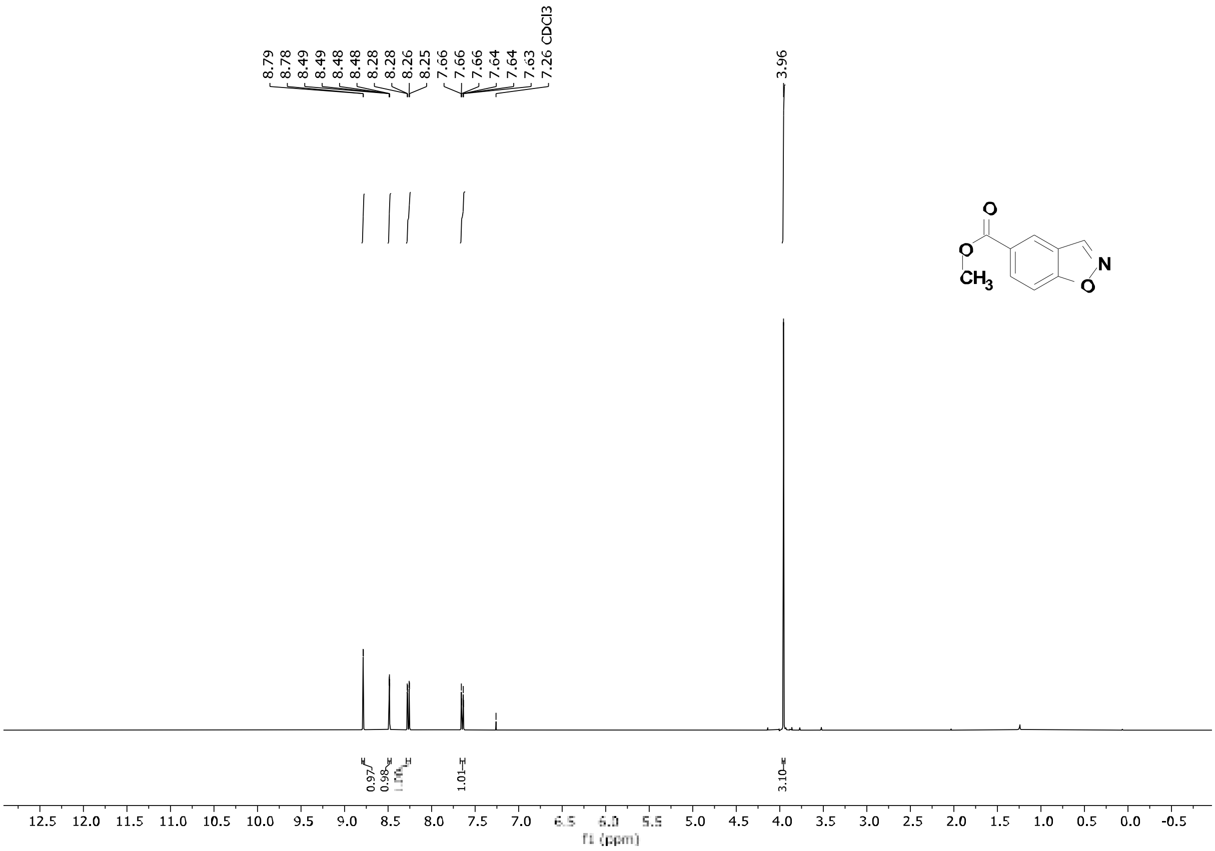

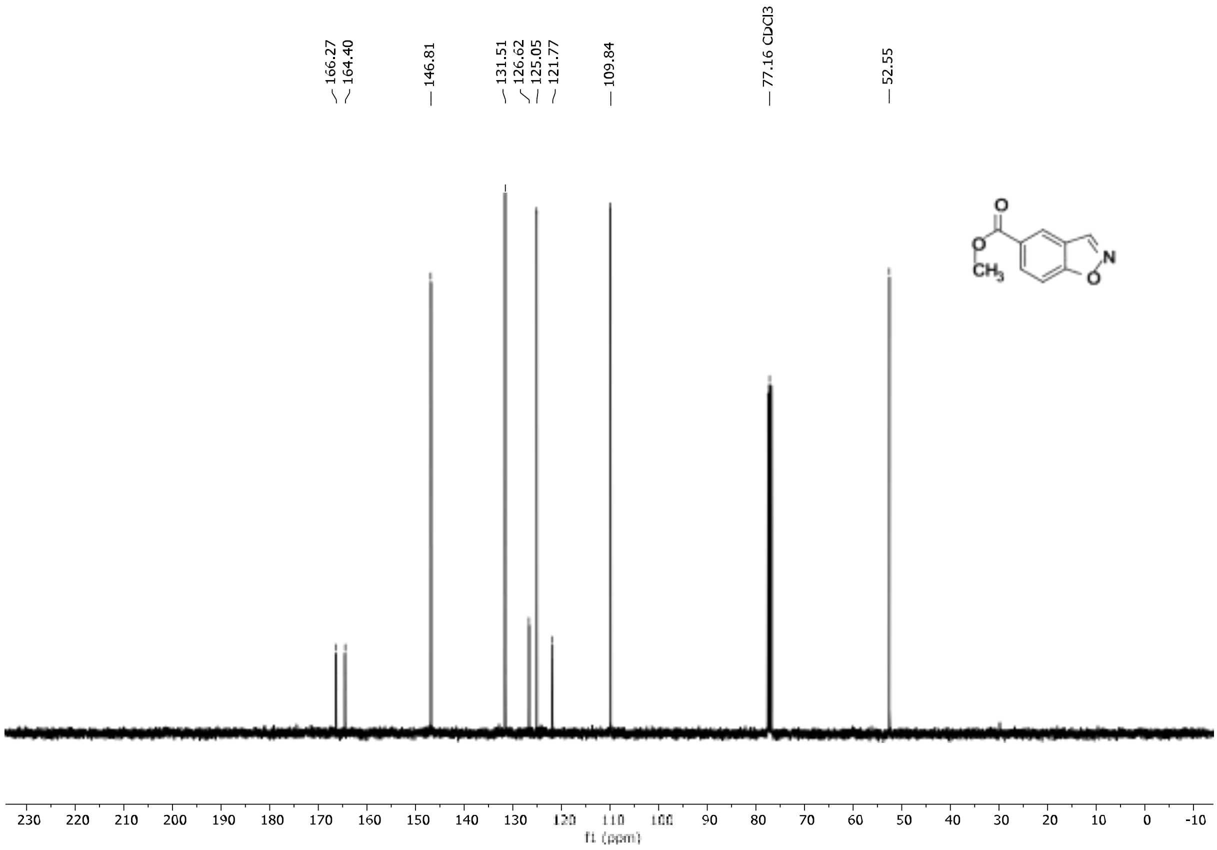

tert-*Butyl benzo[*d*]isoxazol-5-ylcarbamate (***5d***)*

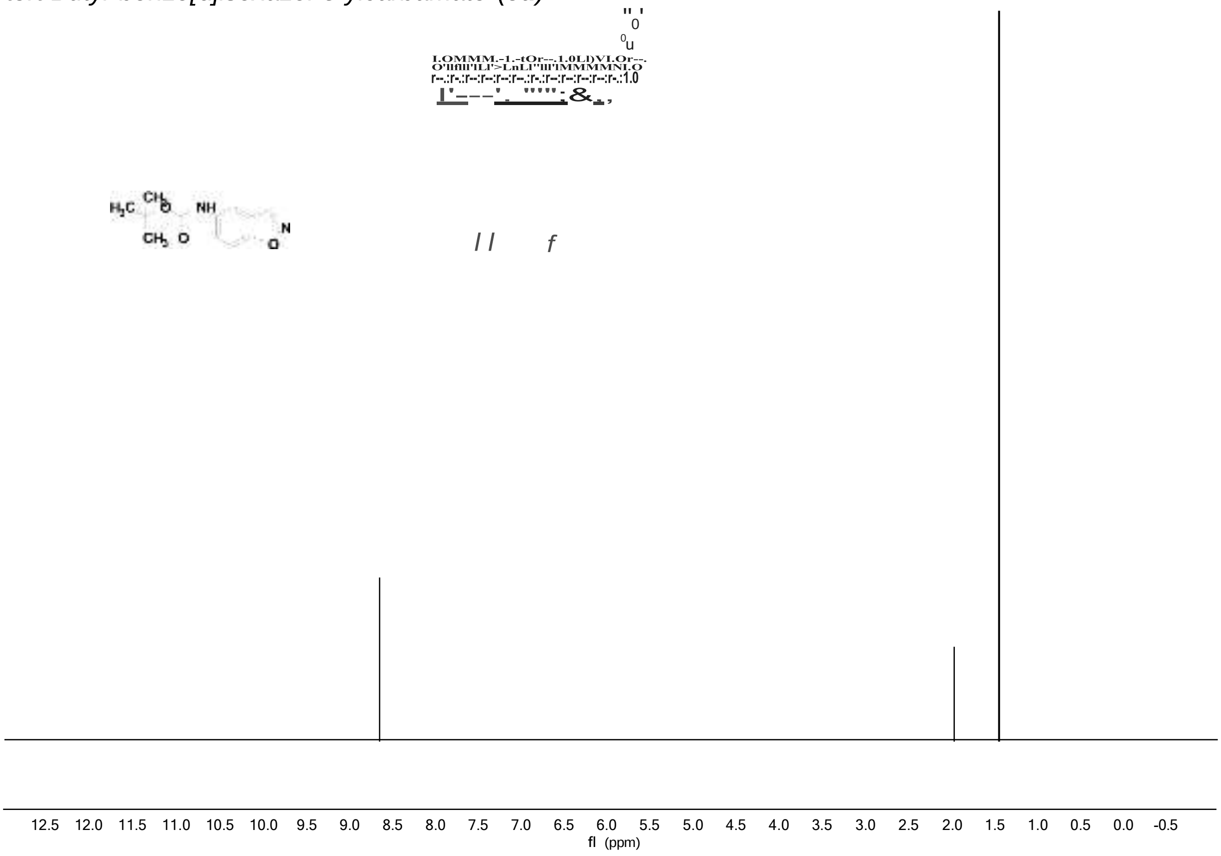

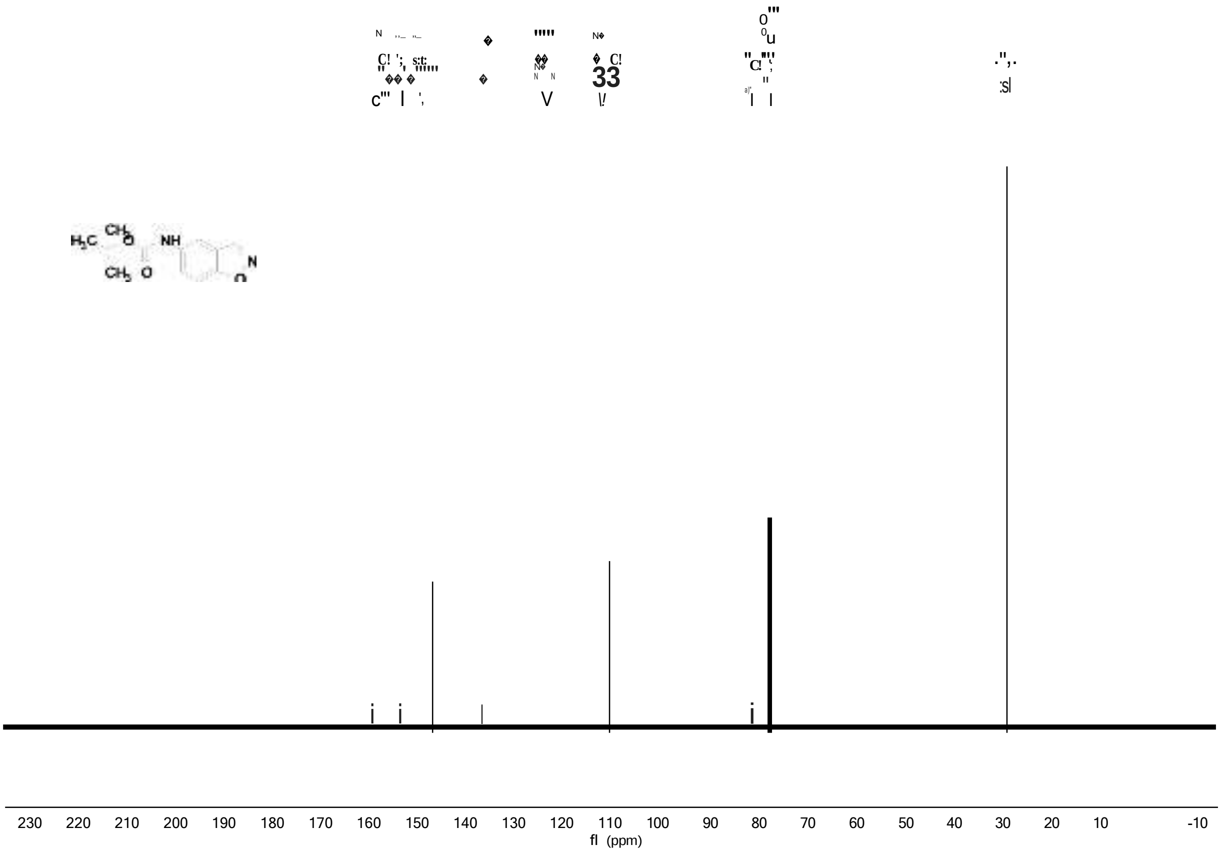

*6-Phenylbenzo[*d*]isoxazole* (***5e***)

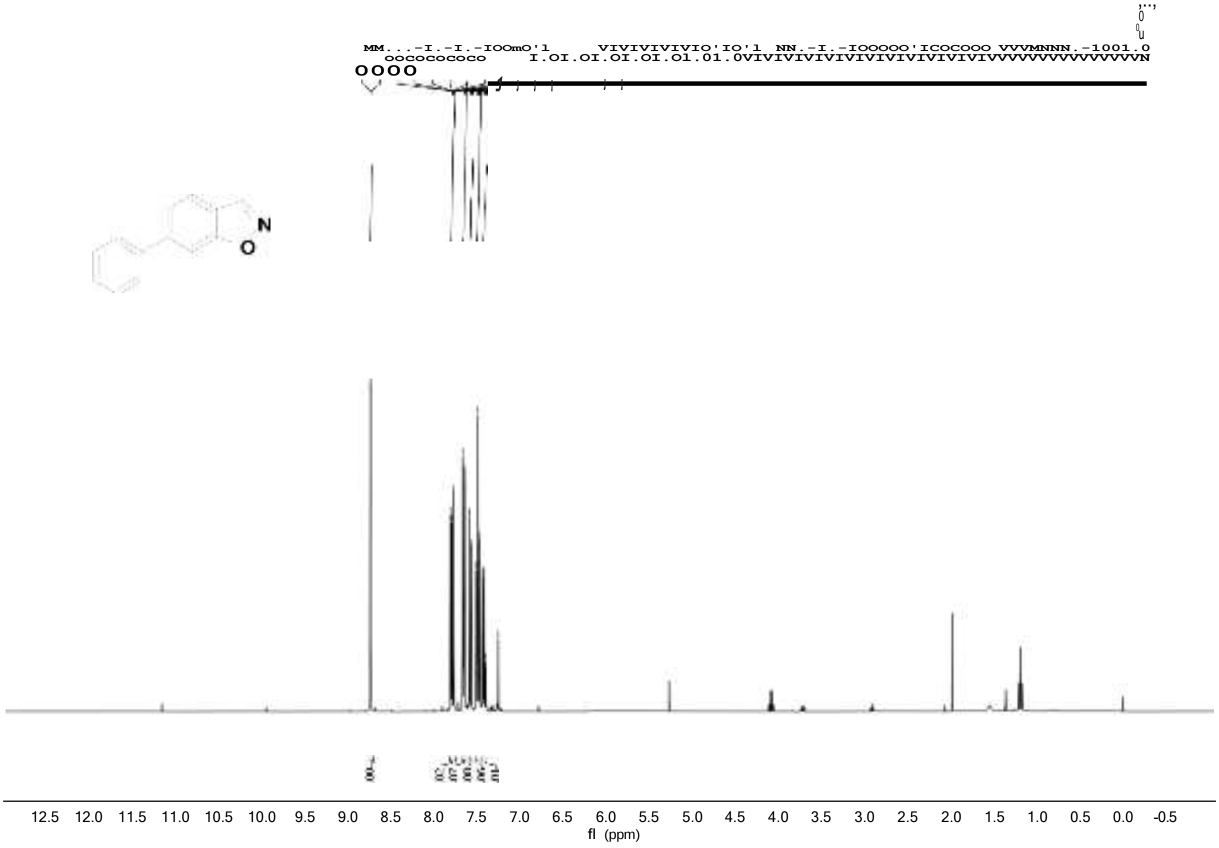

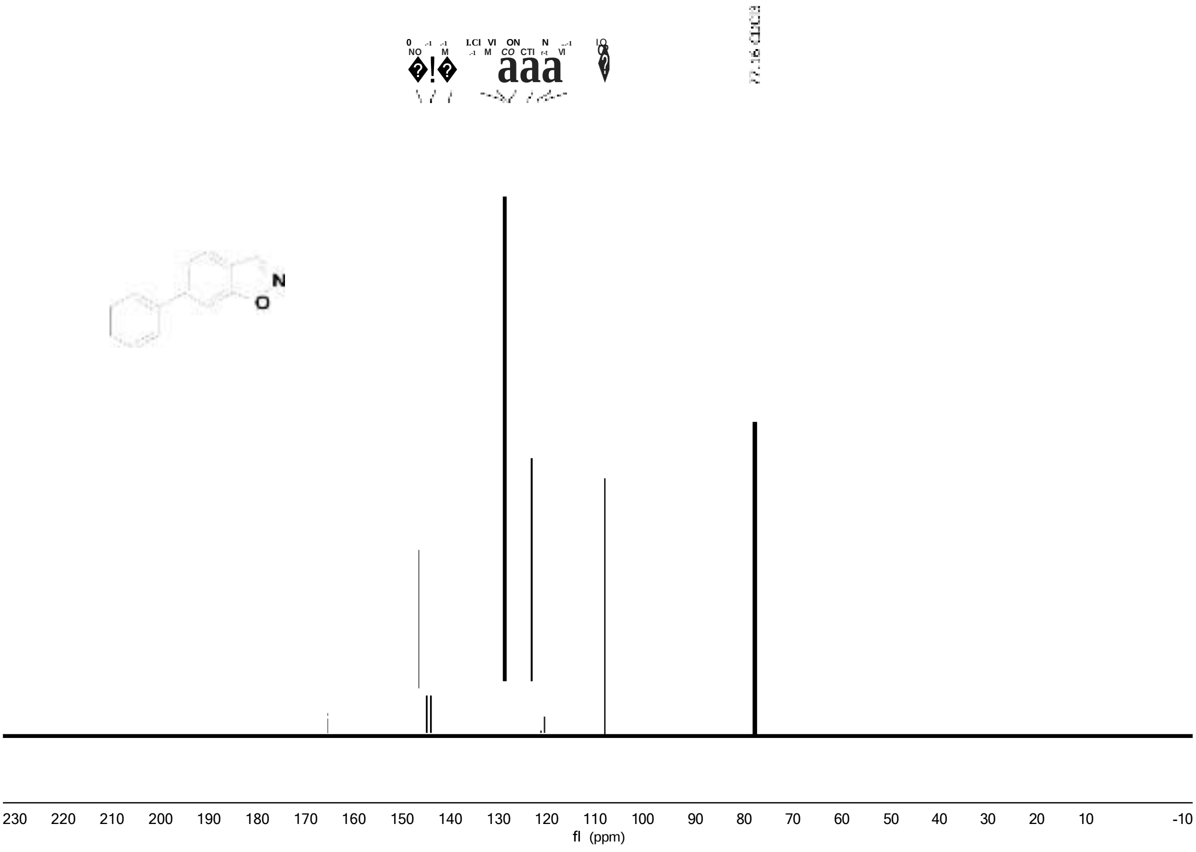

*6-Methoxybenzo[*d*]isoxazole* (***5f***)

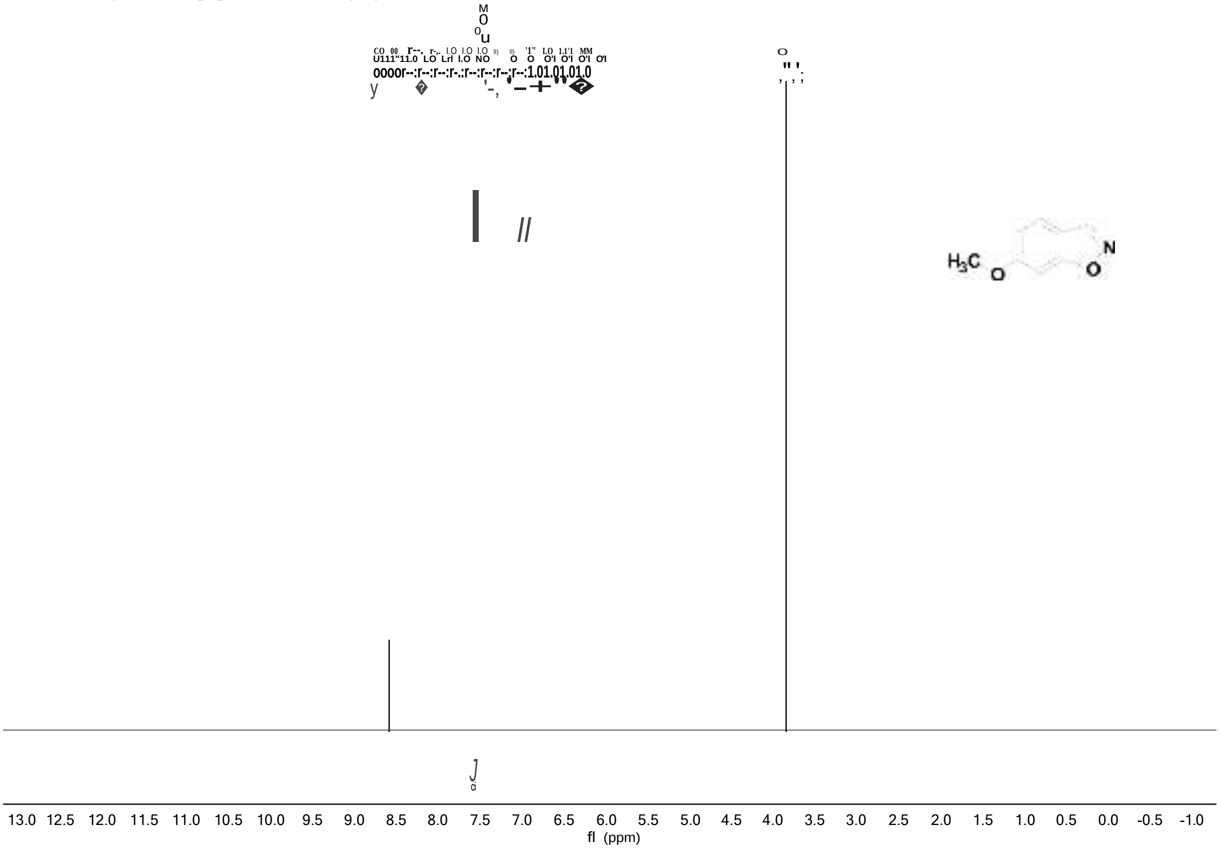

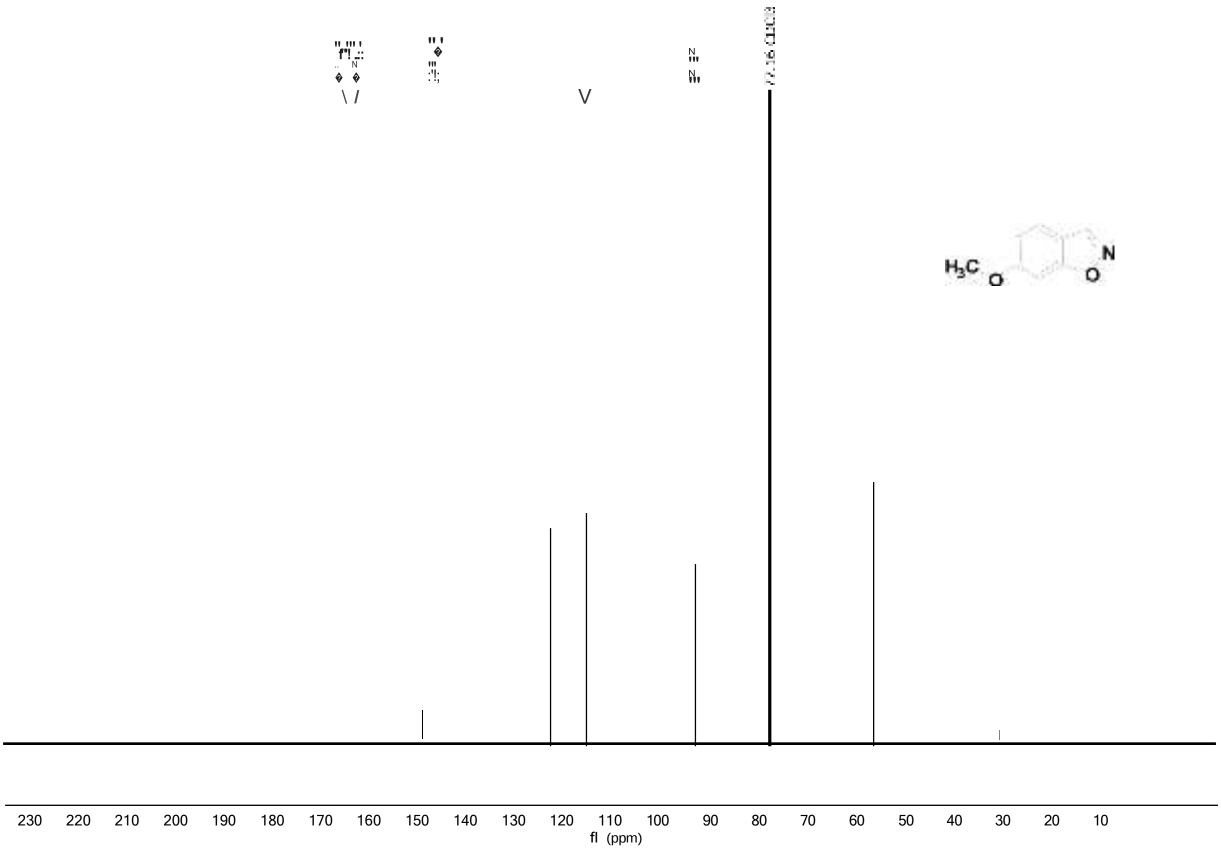

4-methoxy-7H-chromeno[6,7-d]isoxazol-7-one (**5g**)

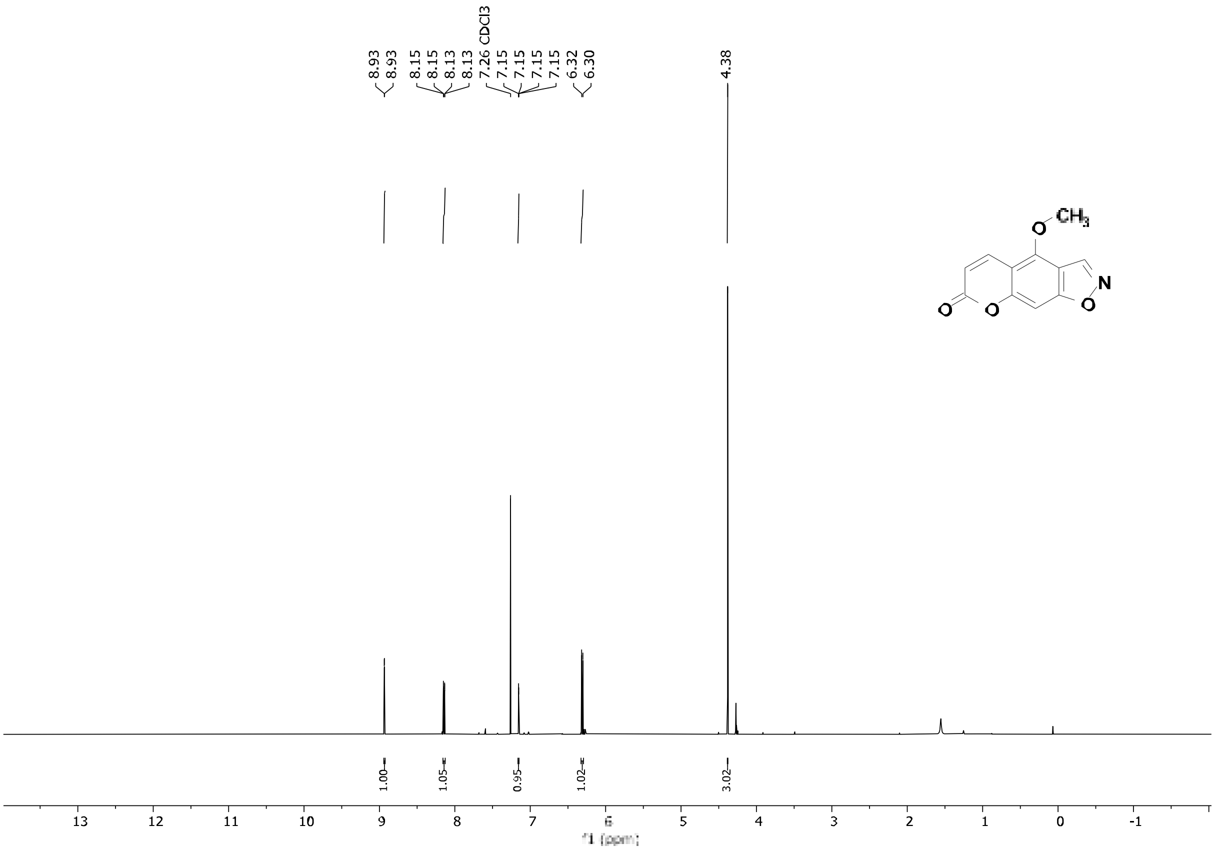

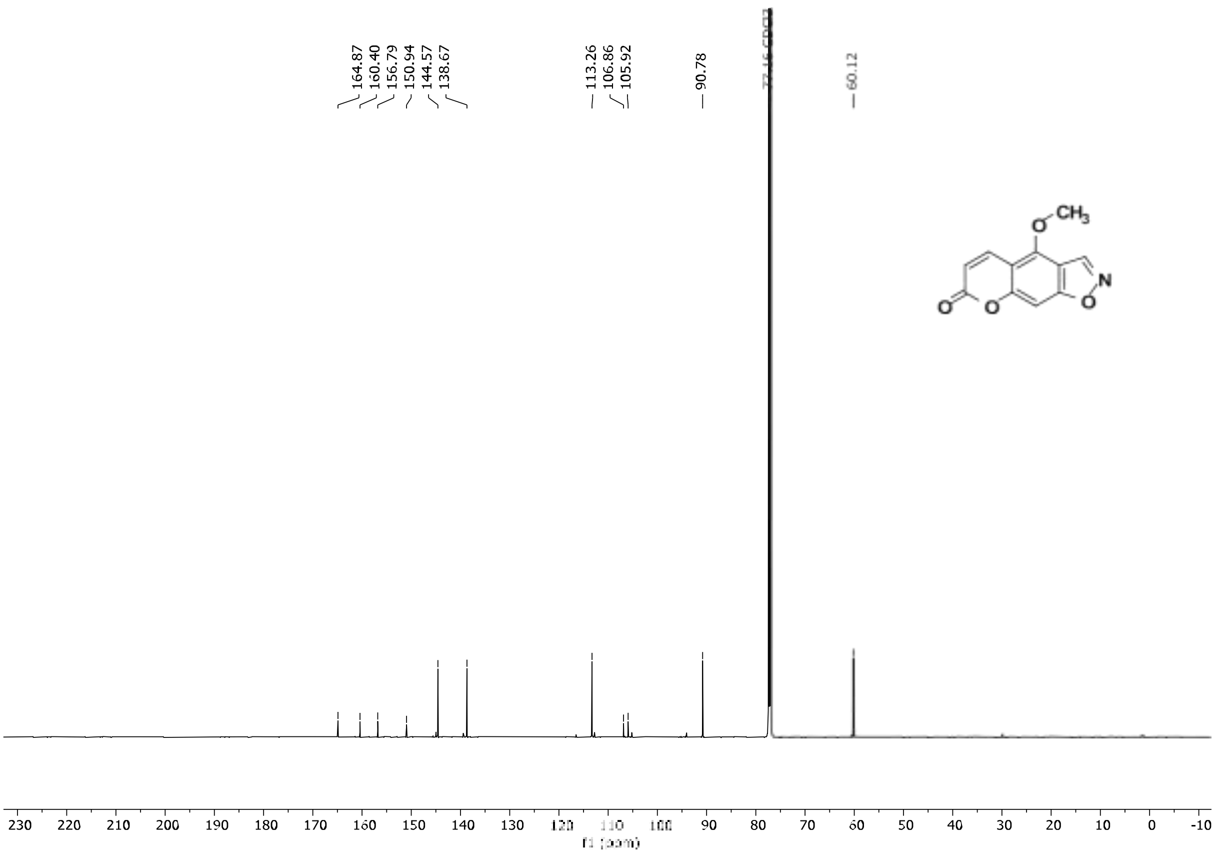

*6-(4-(Methylsulfonyl)phenyl)benzo[*d*]isoxazole* (***5h***)

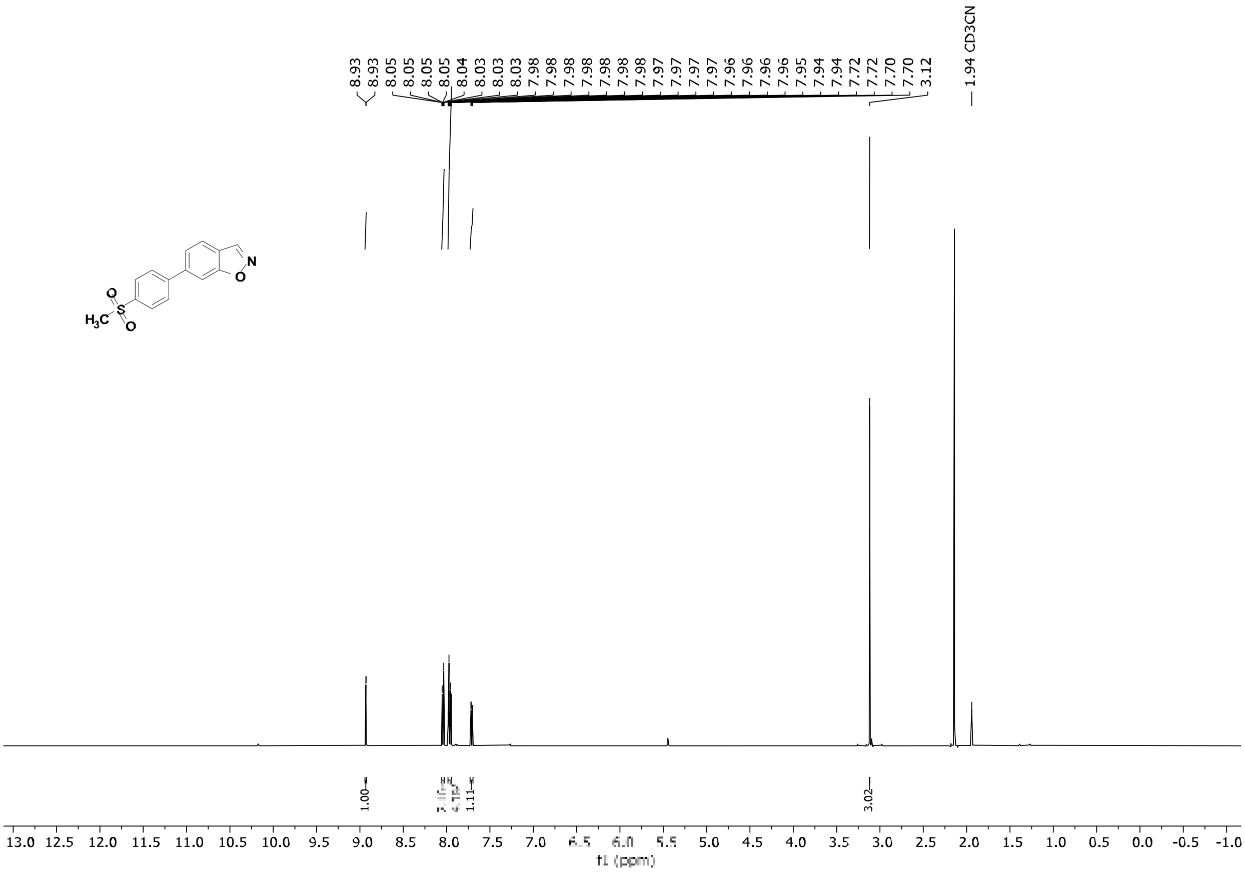

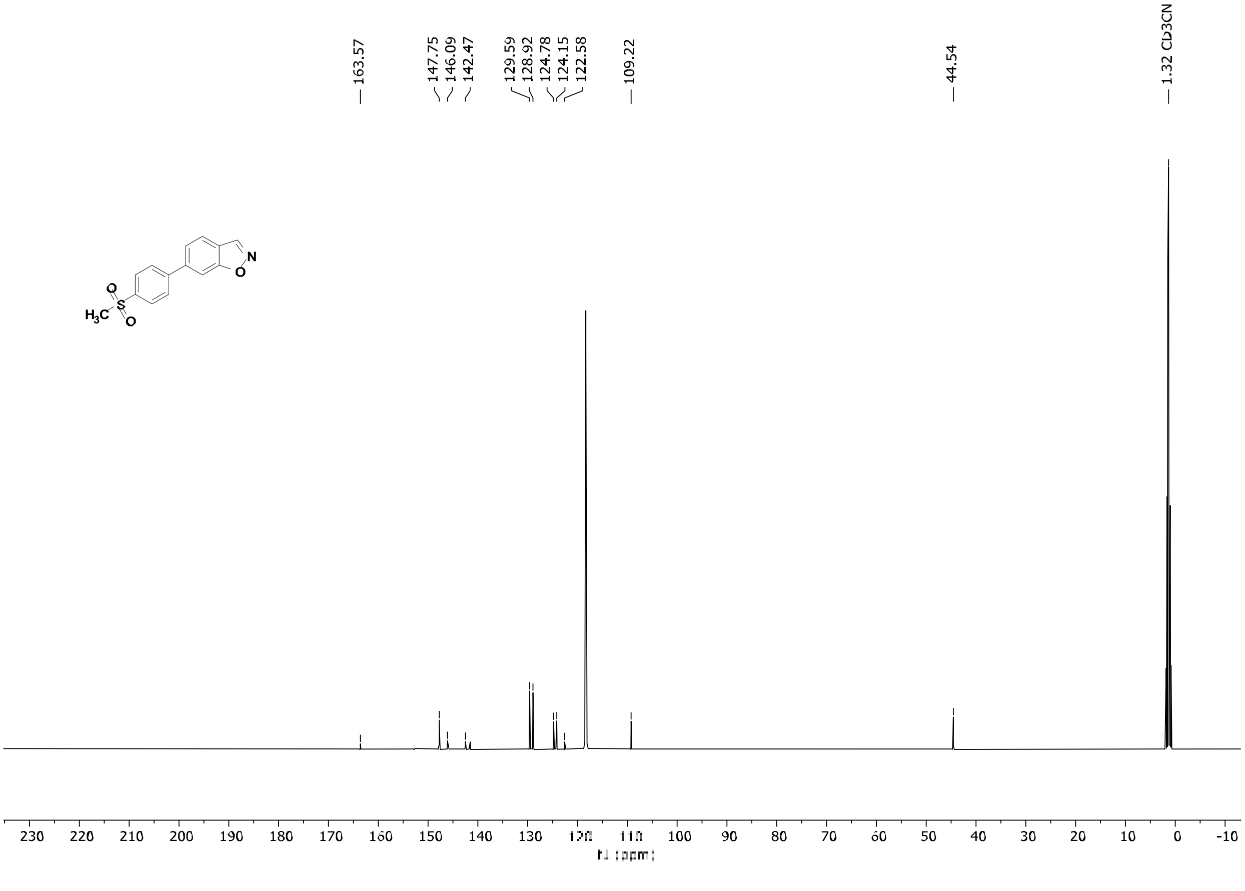

*5-(3-Chlorophenyl)benzo[*d*]isoxazole (**5i**)*

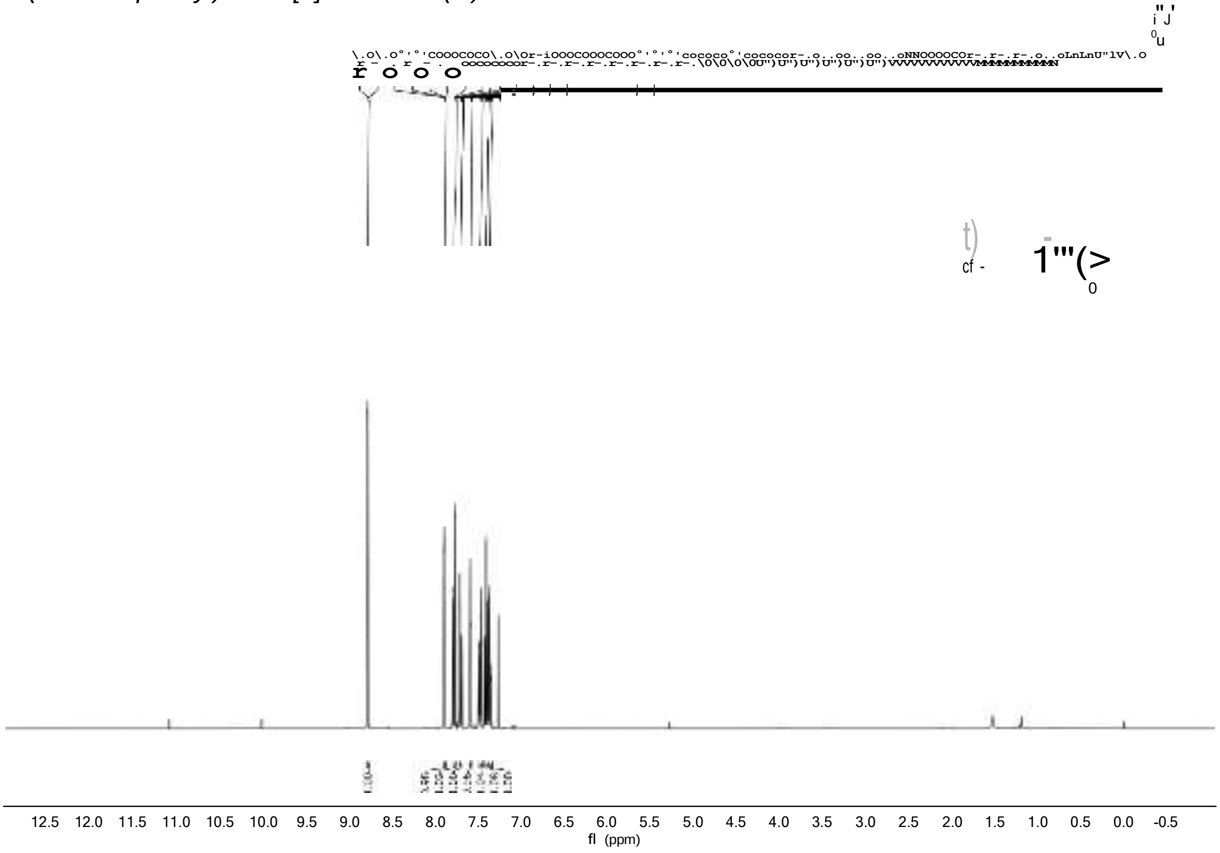

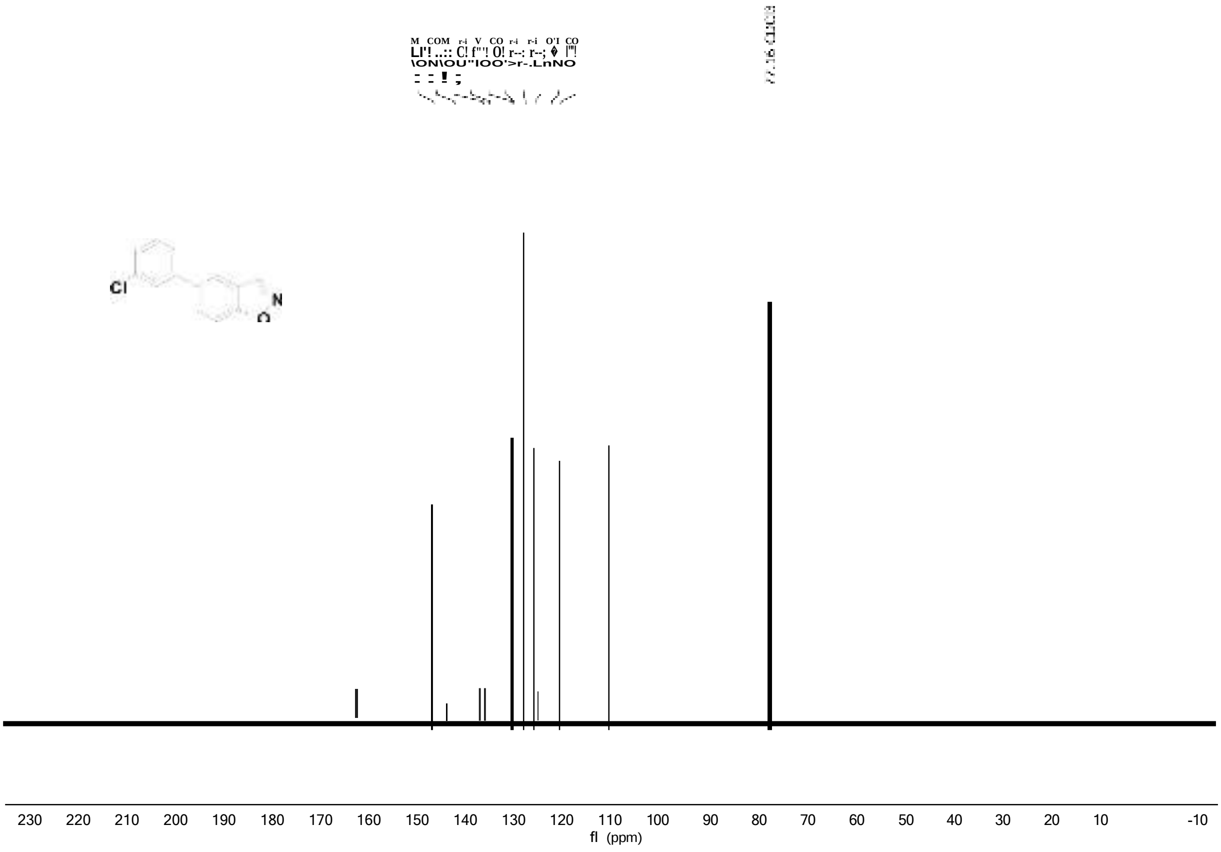

*5-(2-(Trifluoromethyl)phenyl)benzo[*d*]isoxazole (**5j**)*

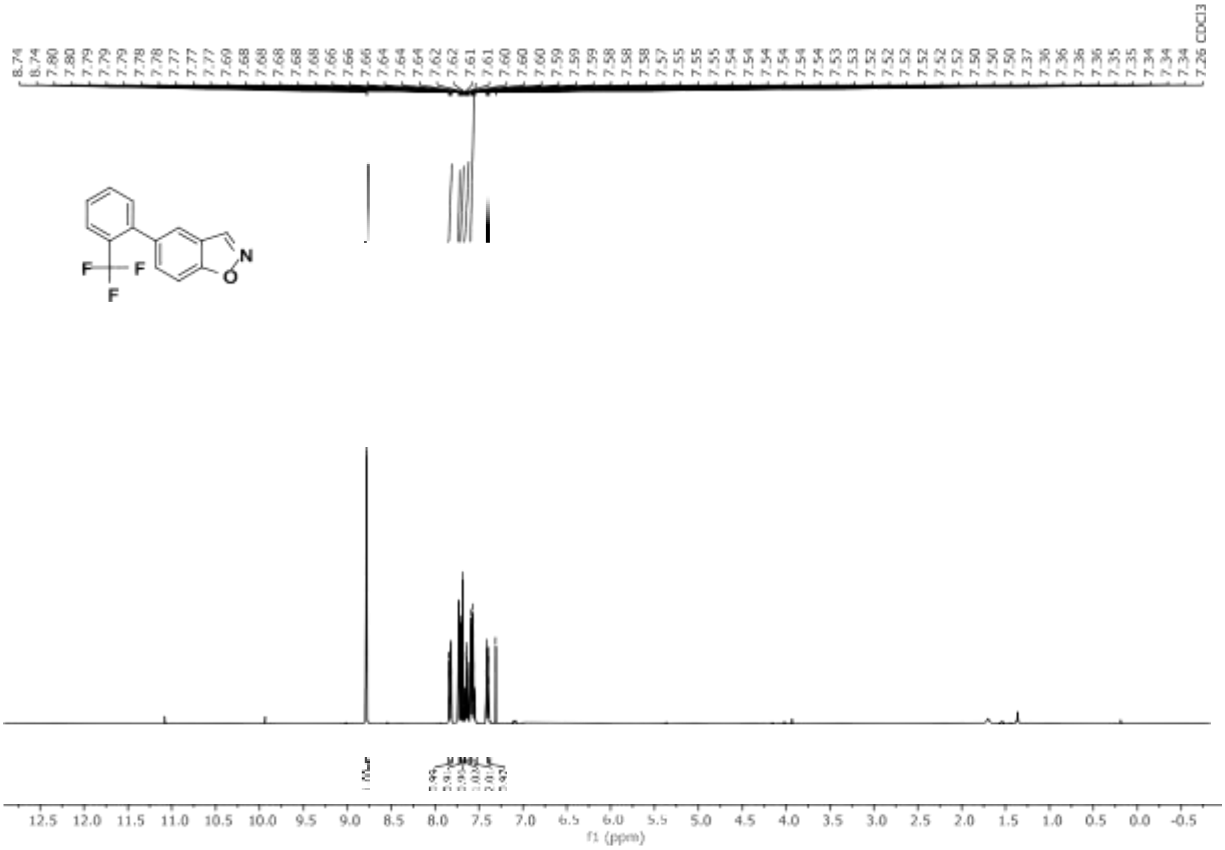

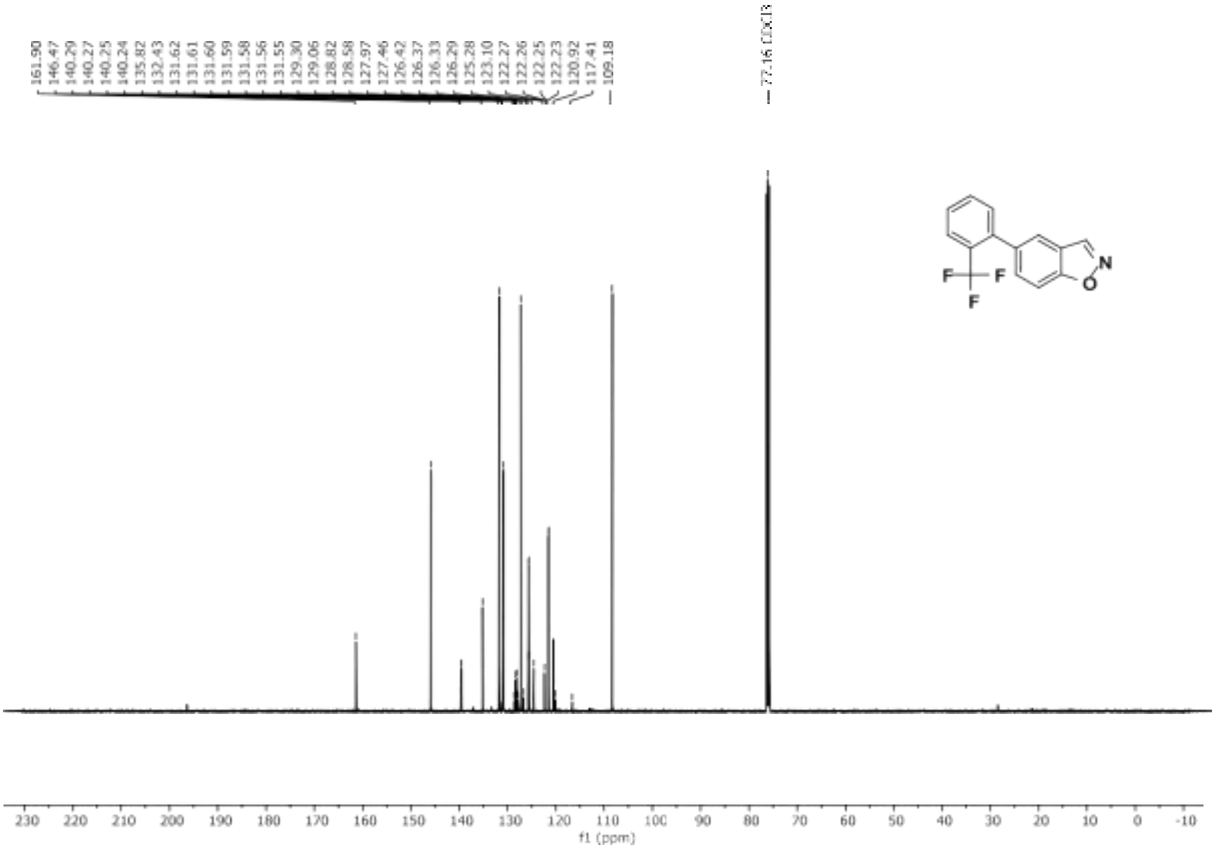

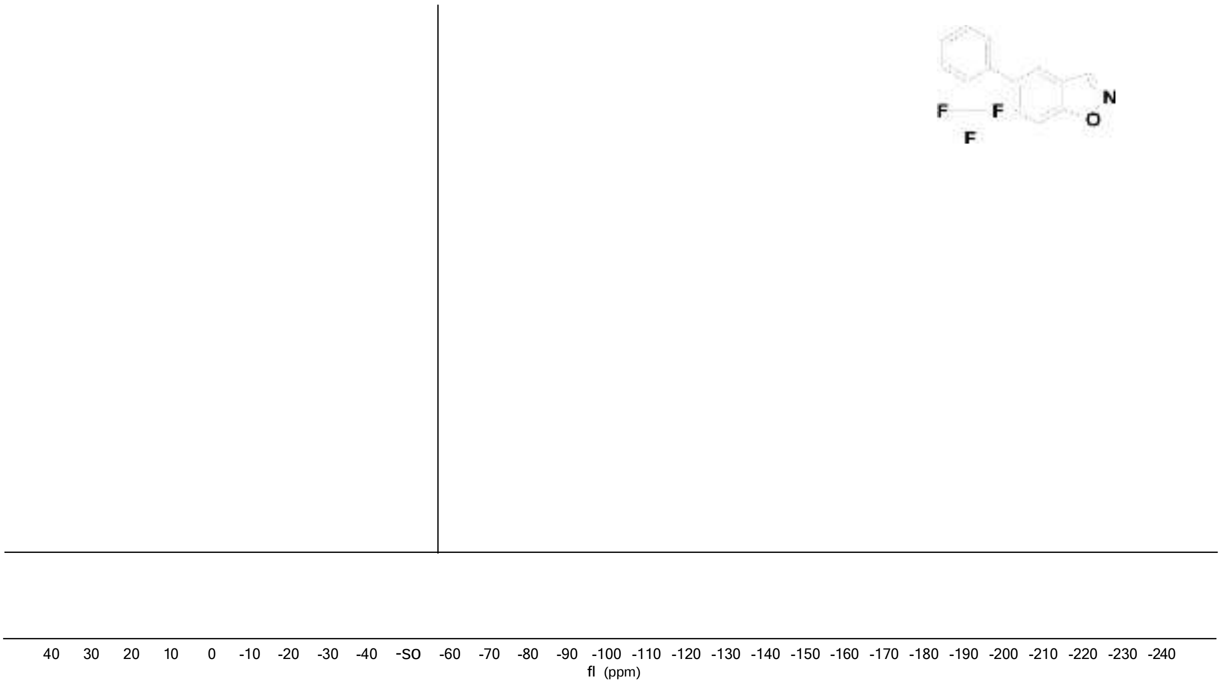

N-Phenethylbenzo[d]isoxazole-5-carboxamide (***5k***)

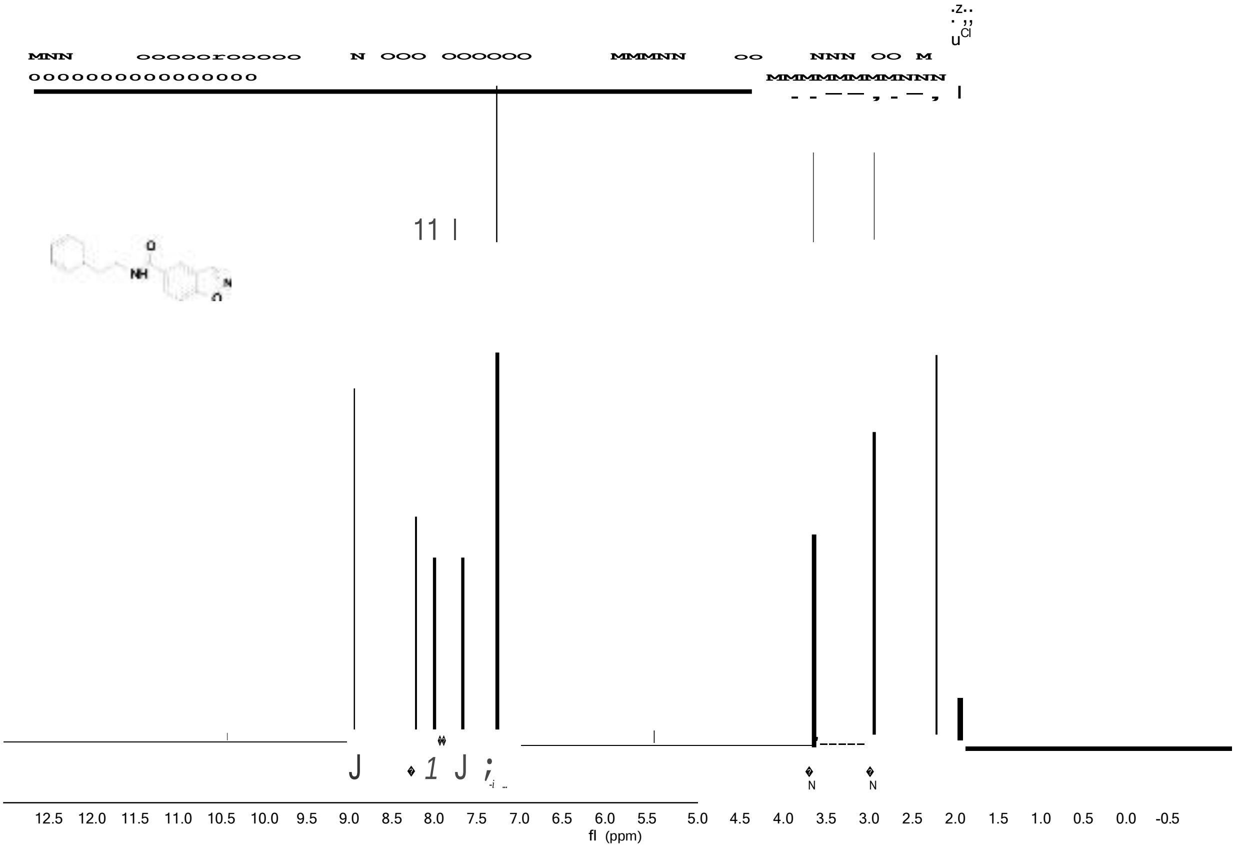

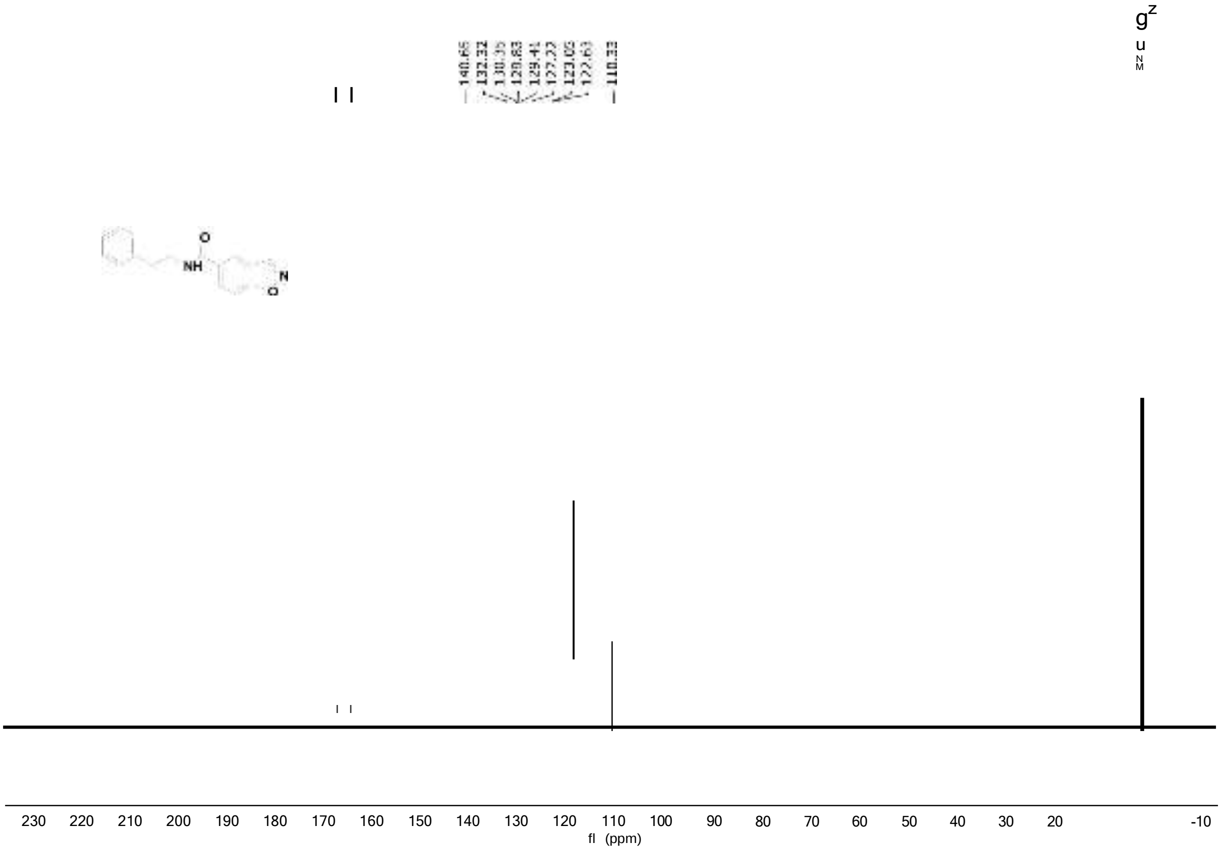

N-*phenethylbenzo[*d*]isoxazole-6-carboxamide* (***5l***)

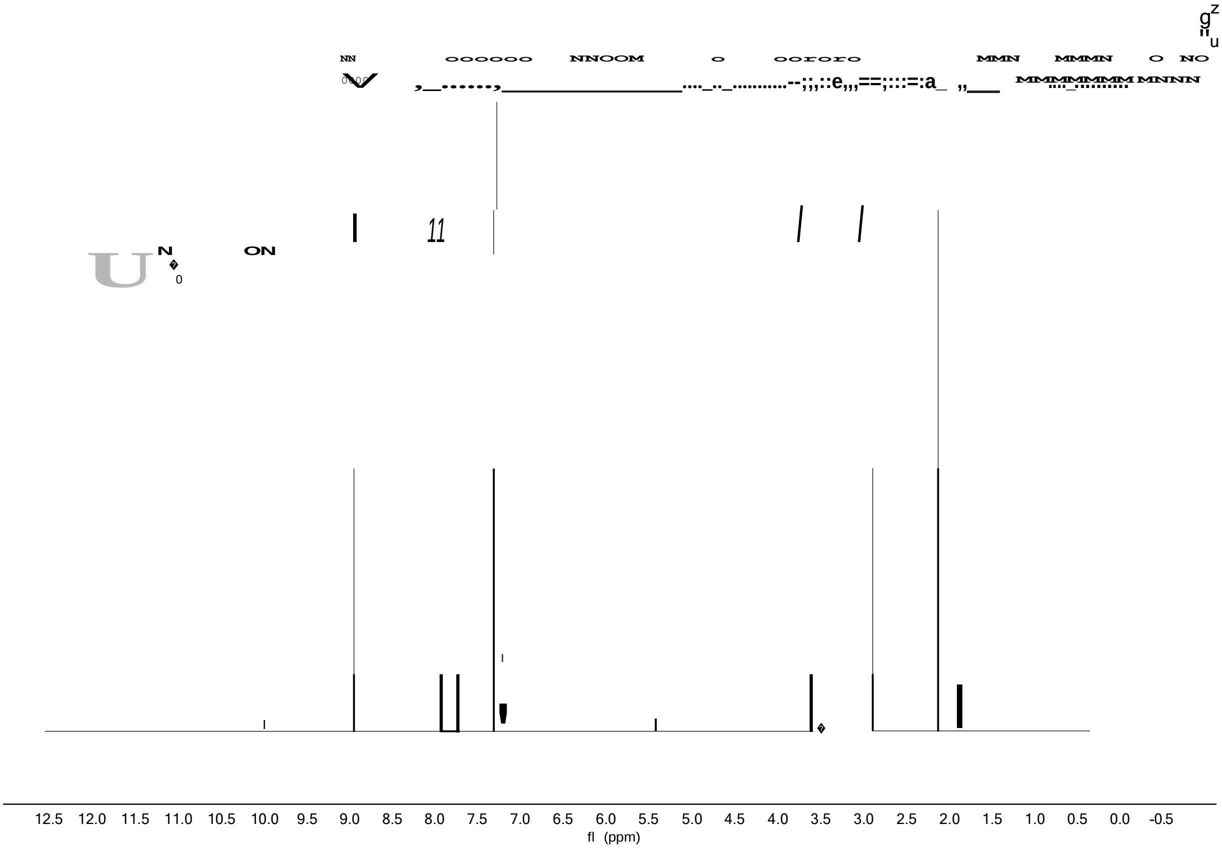

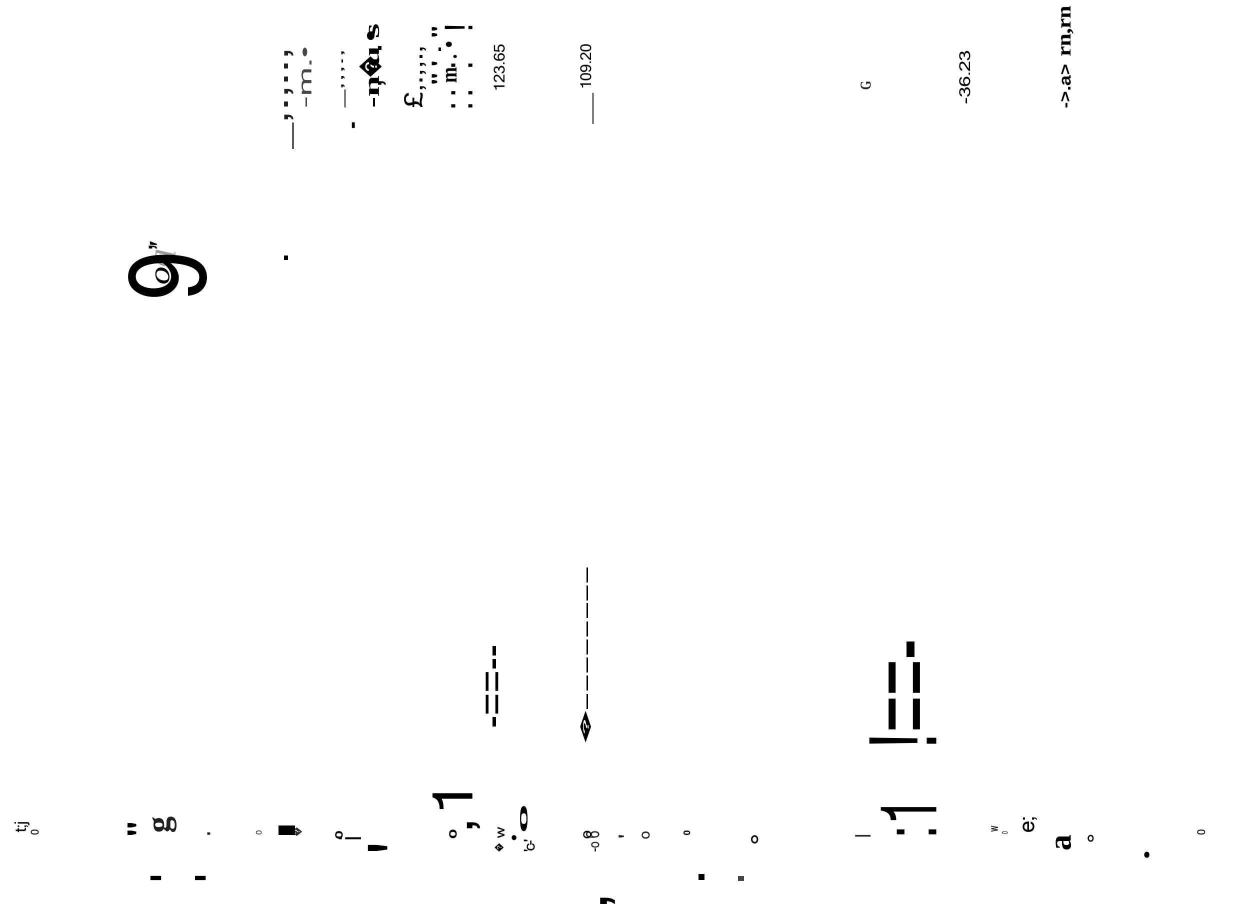

6-Vinylbenzo[*d*]isoxazole ***(5m)***

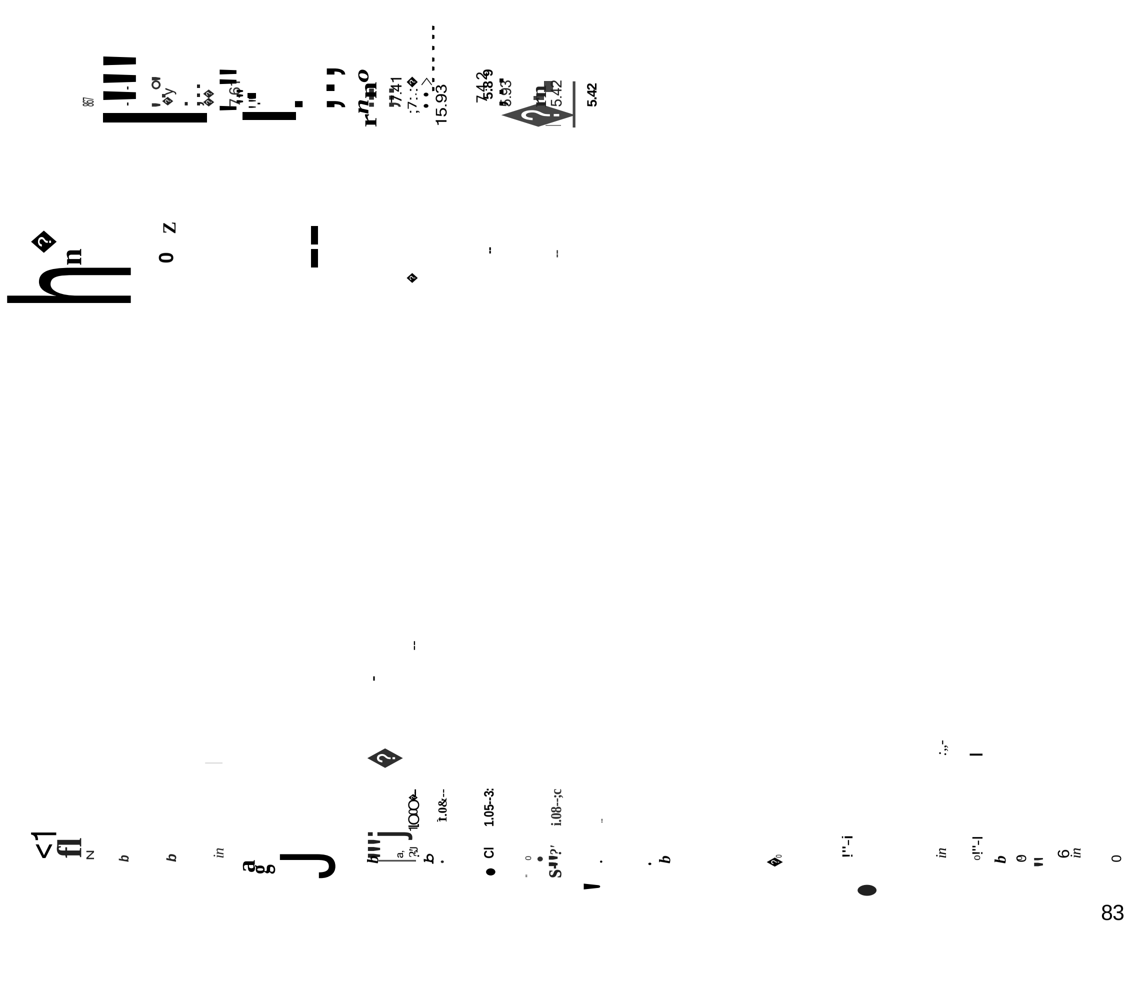

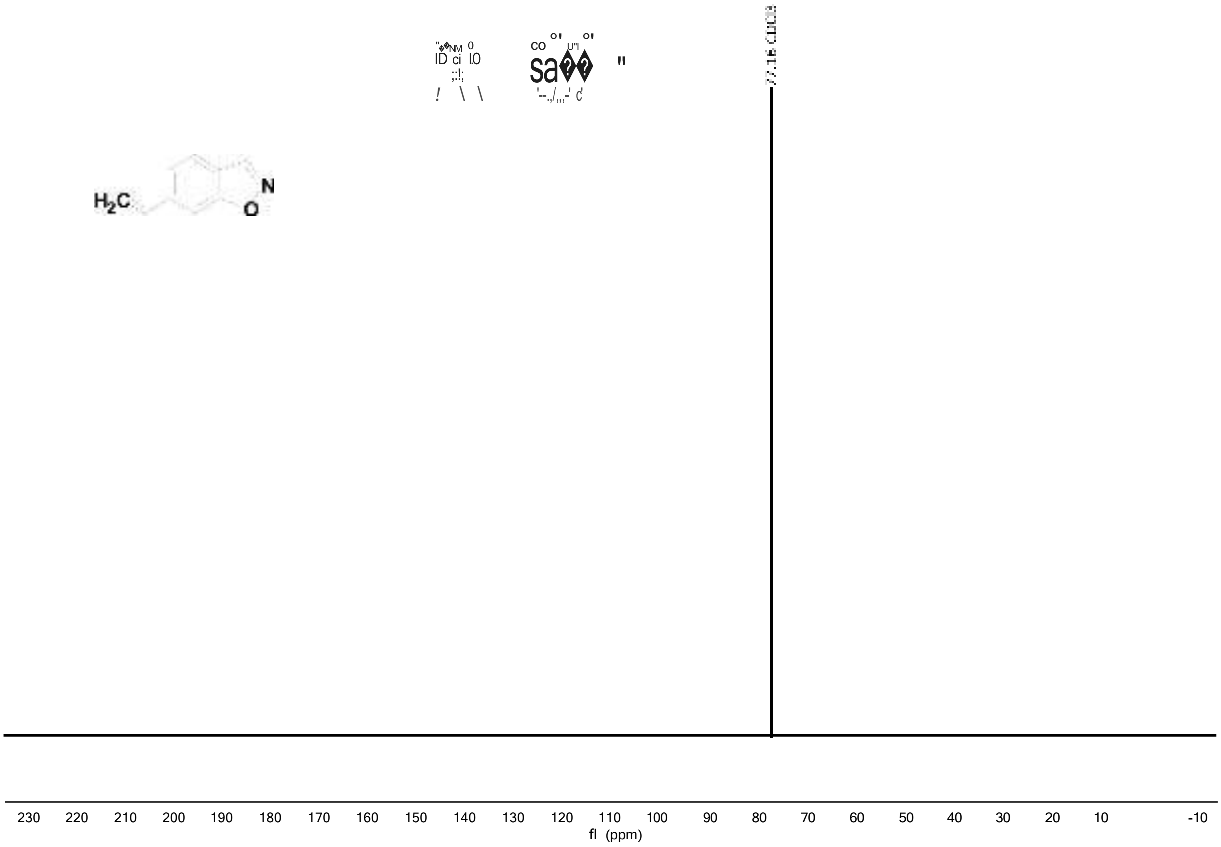
H2C
N
O
230 220 210 200 190 180 170 160 150 140 130 120 110 100 90 80 70 60 50 40 30 20 10 -10
fl (ppm)

NMR Spectra of Benzoxazoles from 3-Substituted Benzofurans

*2-Methylbenzo[*d*]oxazole (**2a**)*

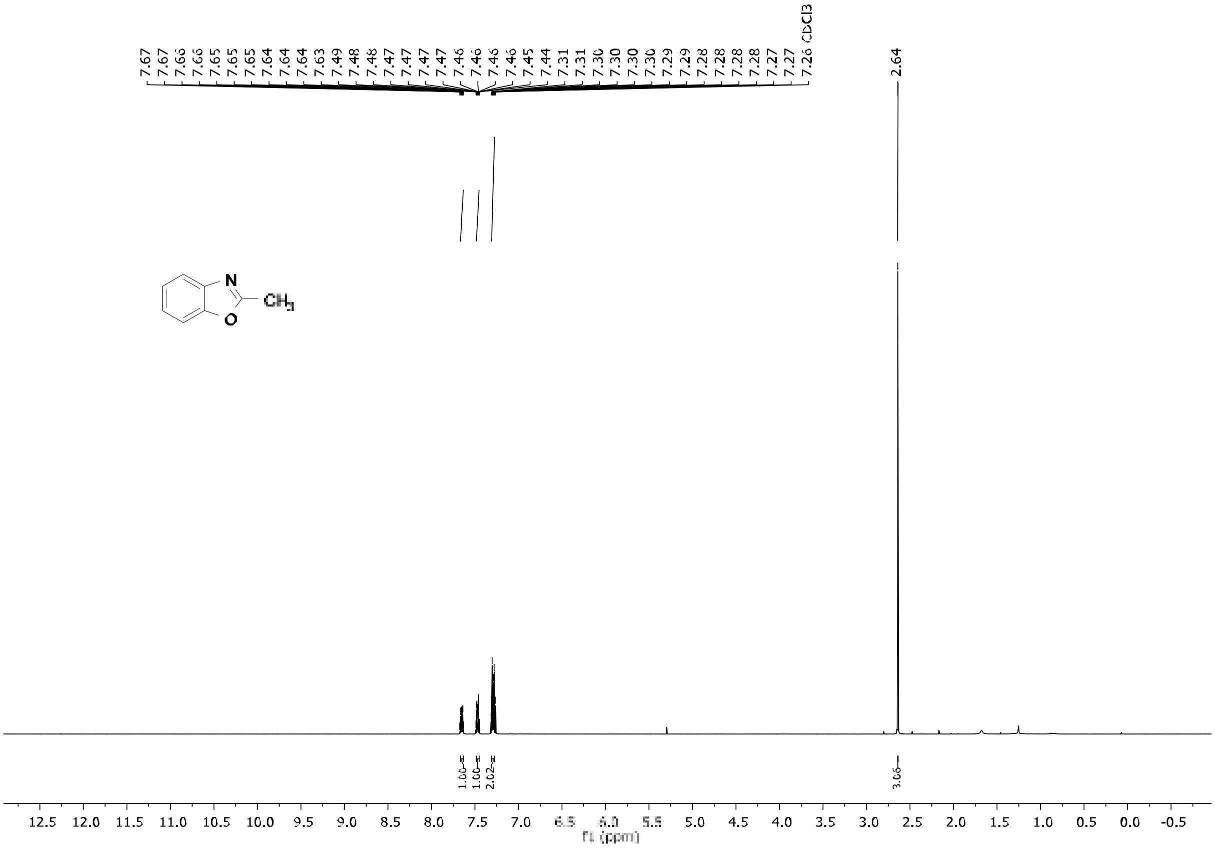

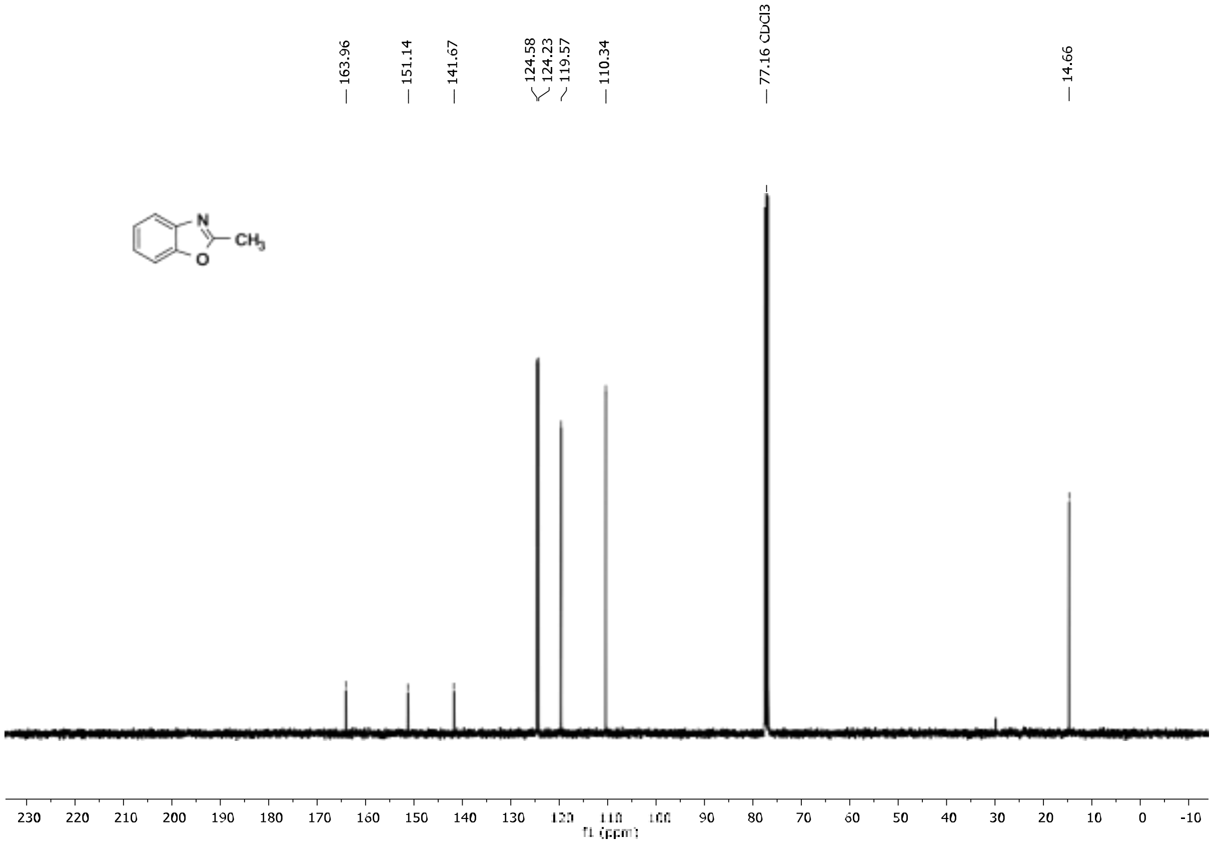

*2-Phenylbenzo[*d*]oxazole (**2b**)*

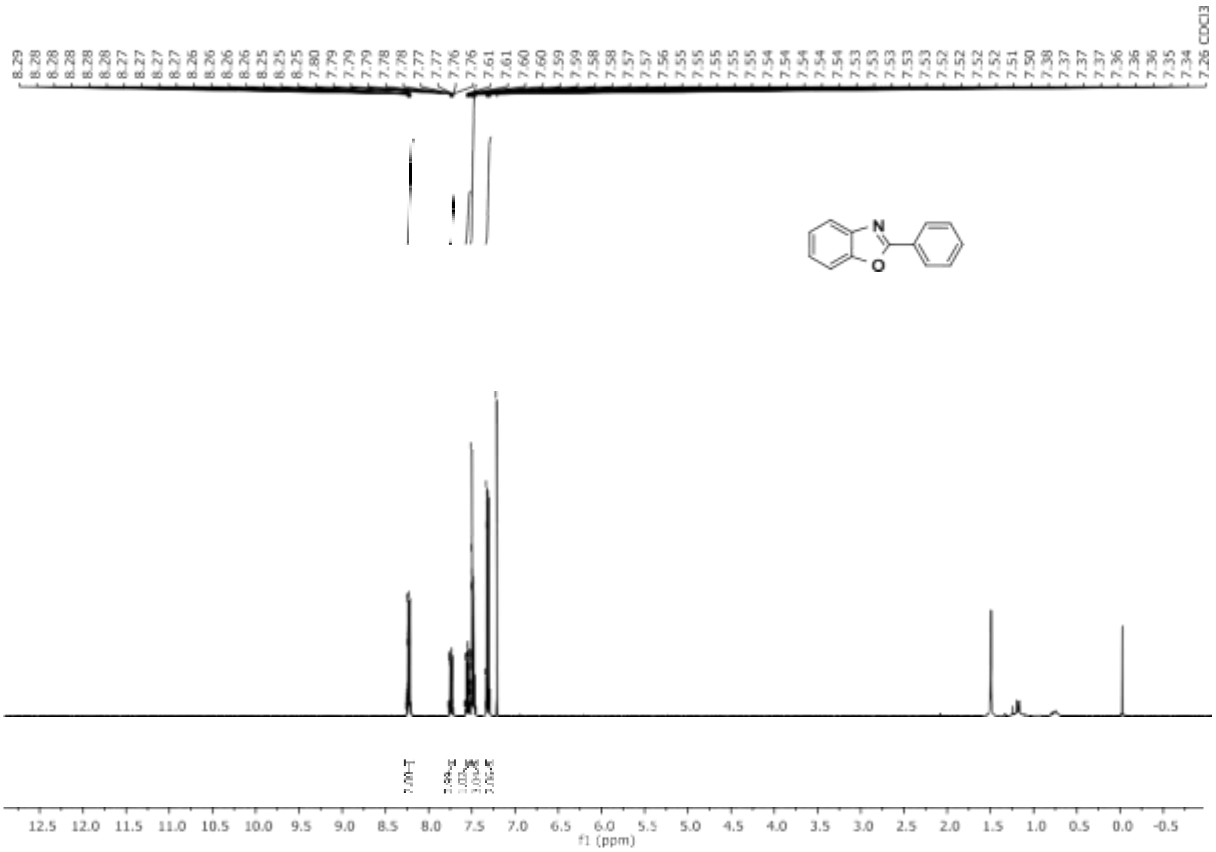

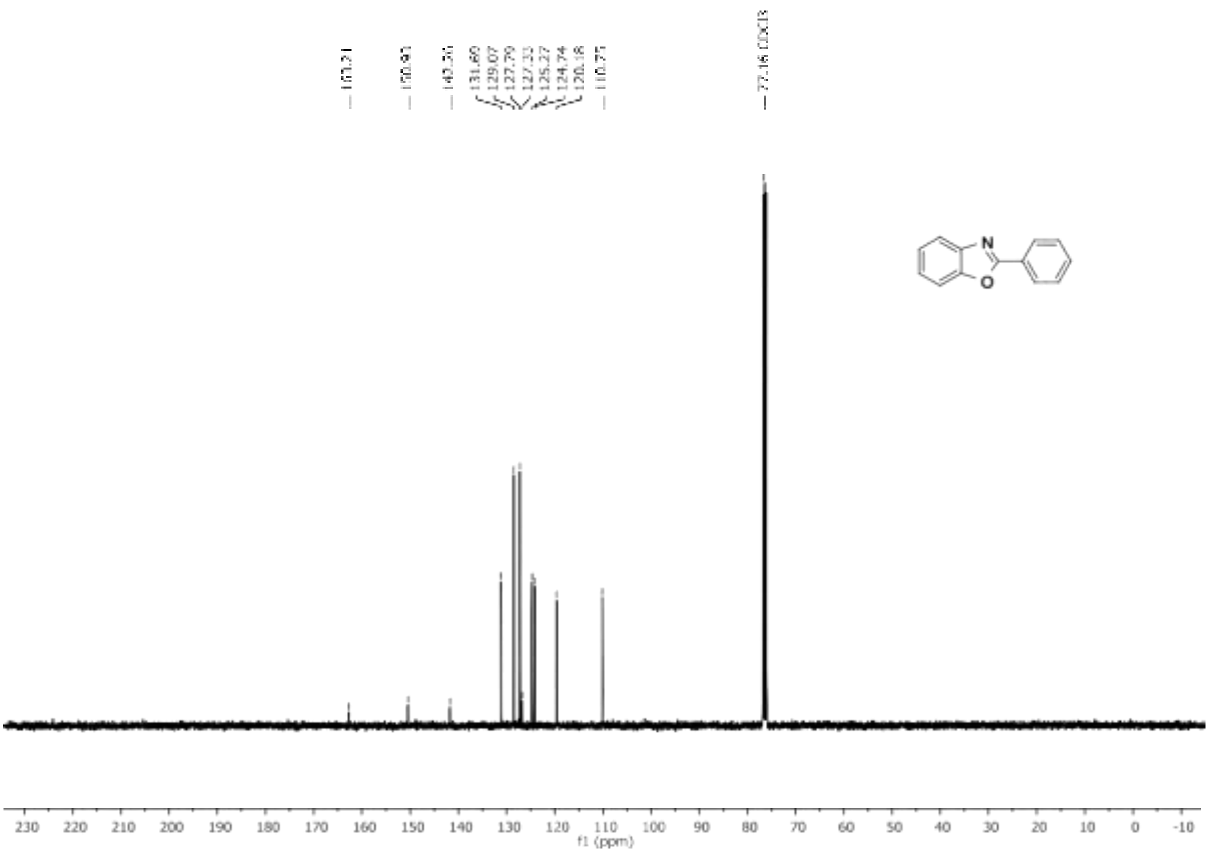

*6-Bromo-2-methylbenzo[*d*]oxazole (**2c**)*

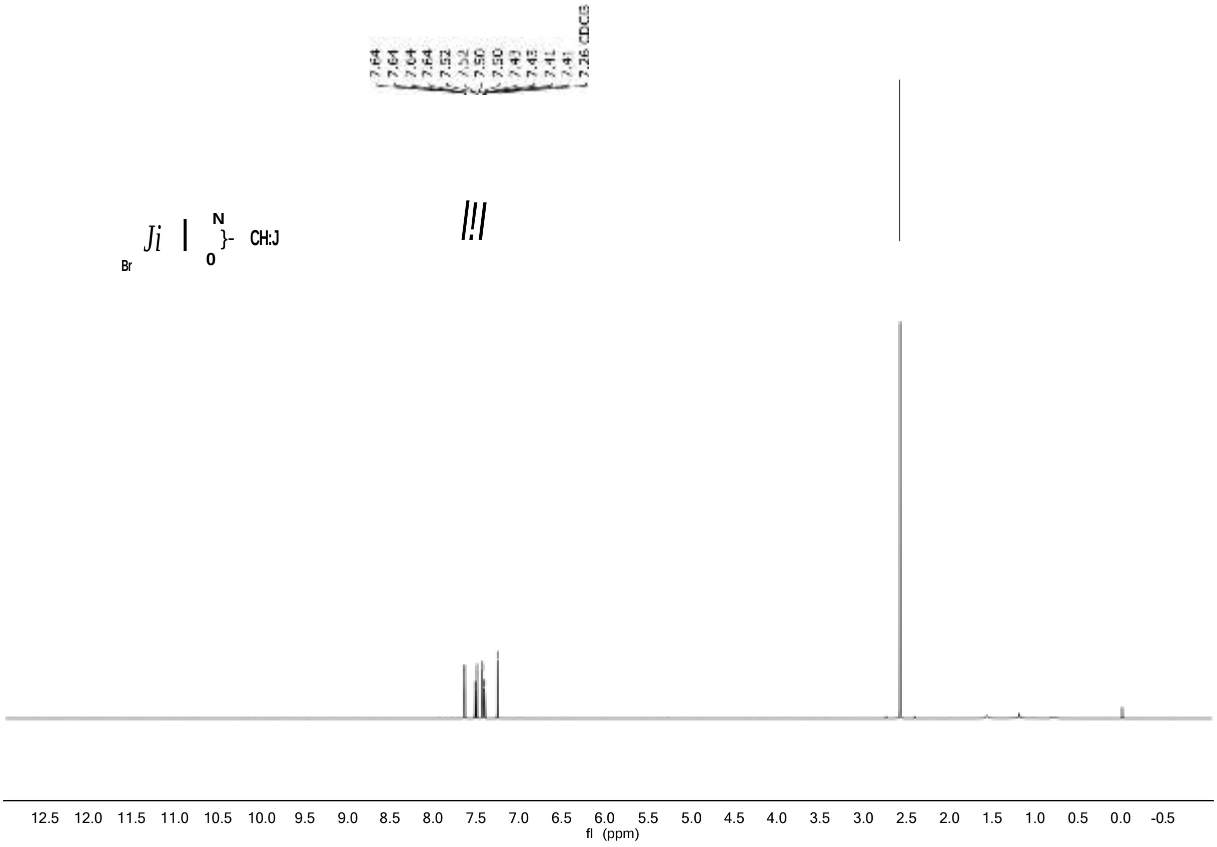

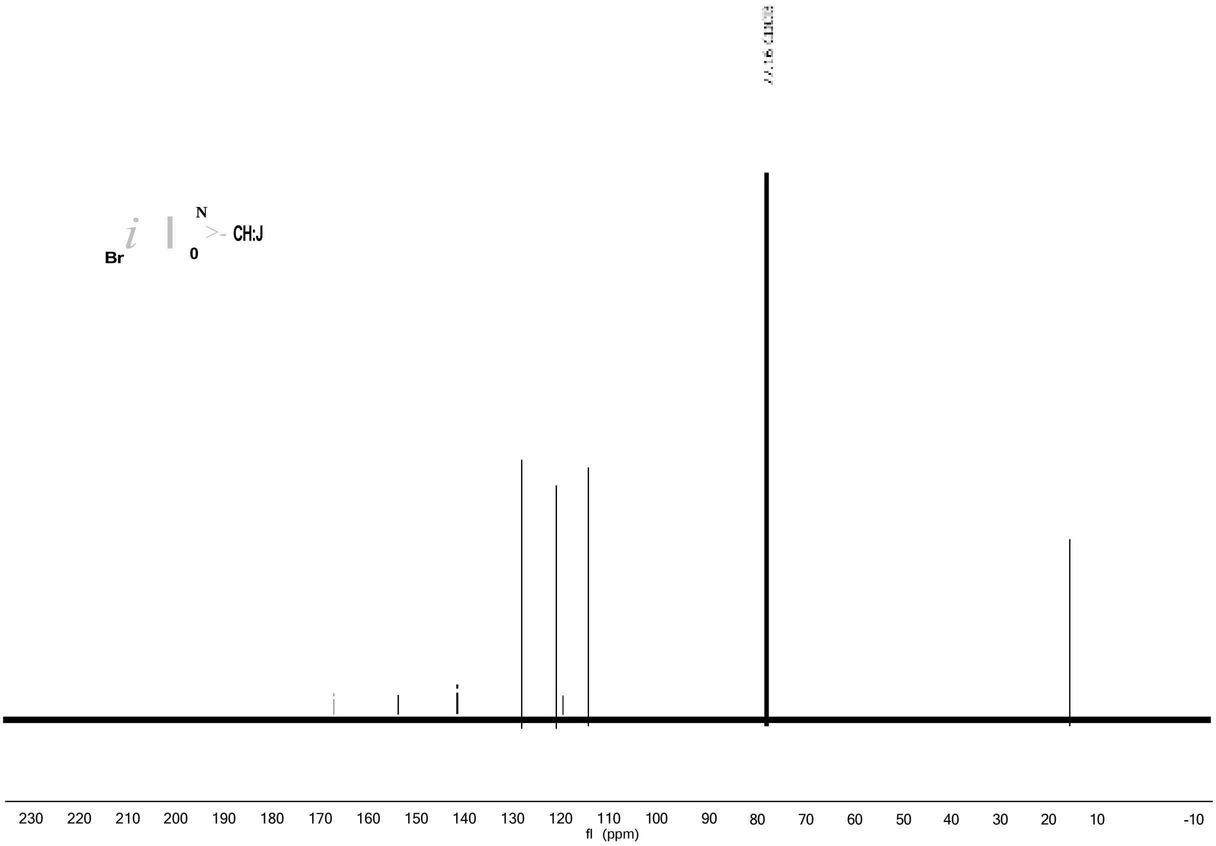

*6-Methoxy-2-methylbenzo[*d*]oxazole* (***2d***)

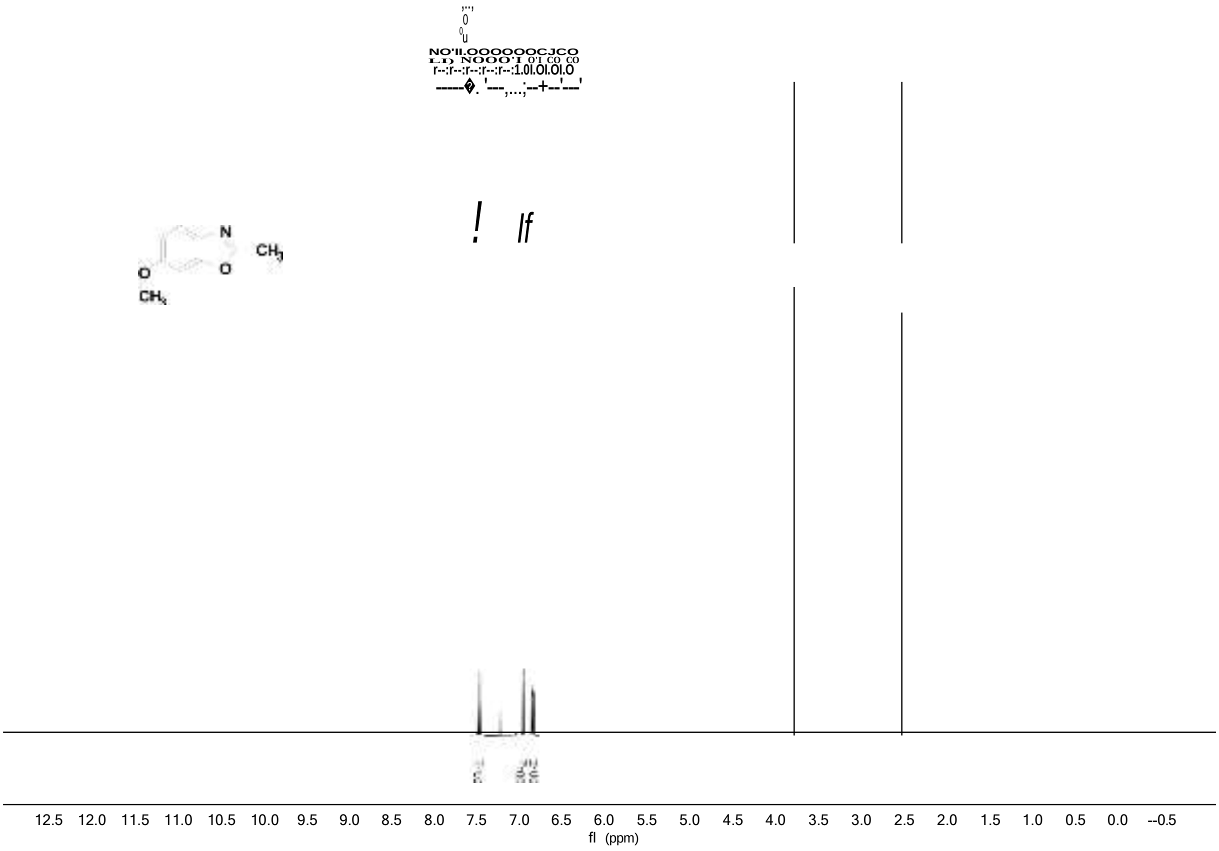

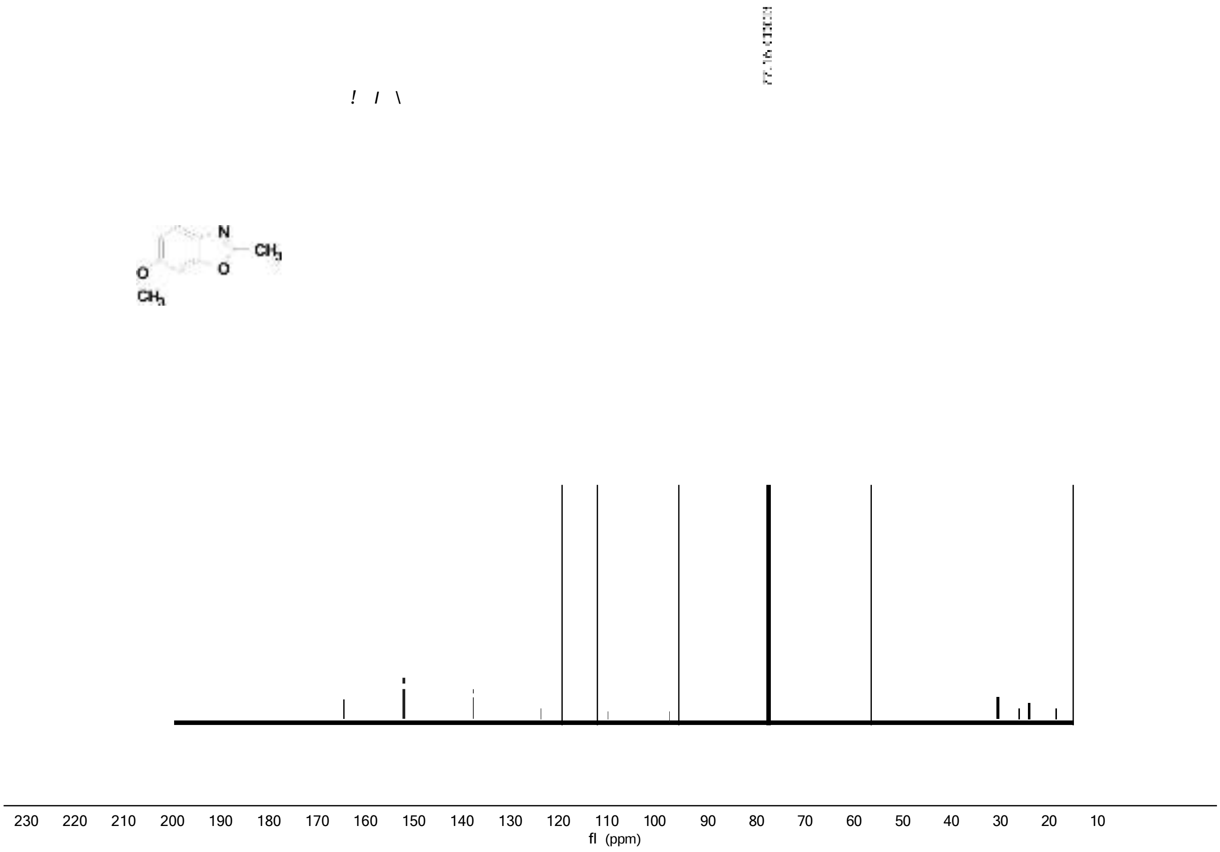

*Methyl 2-methylbenzo[*d*]oxazole-5-carboxylate (**2e**)*

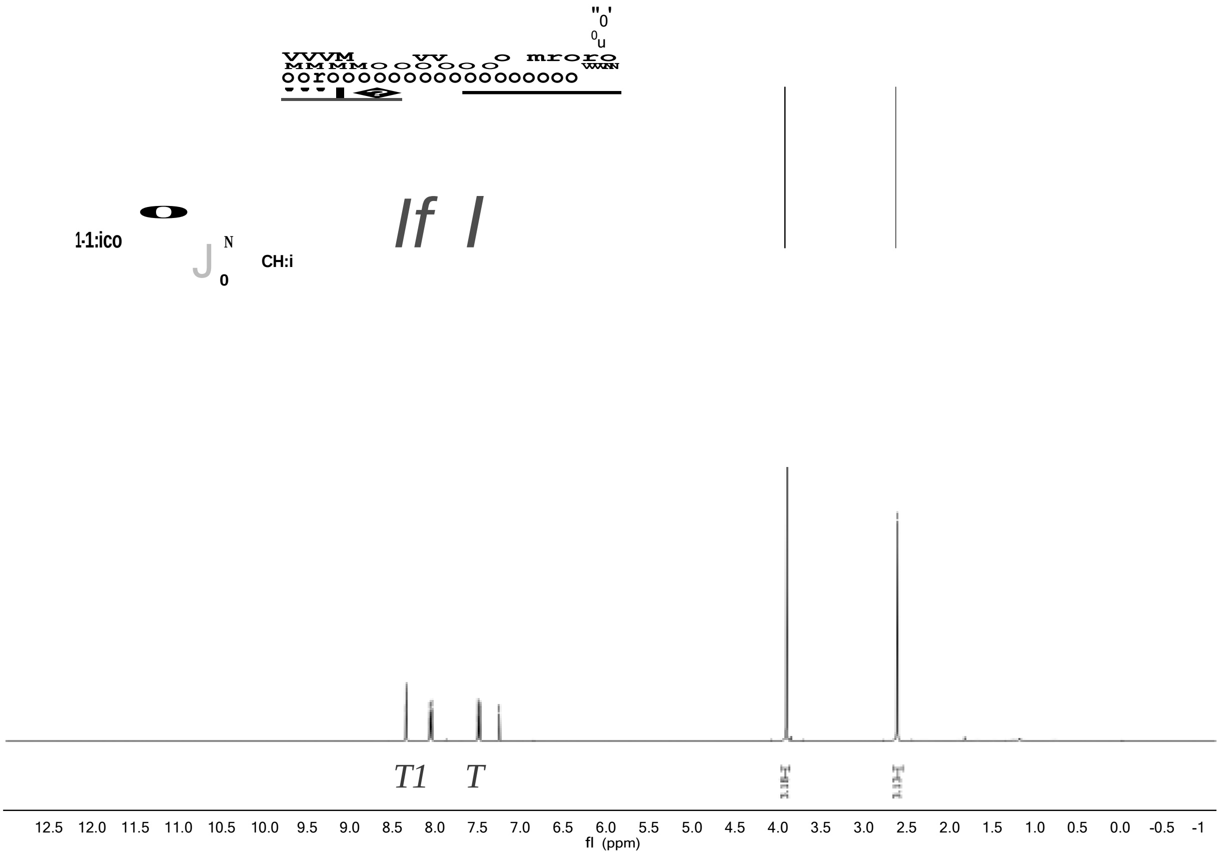

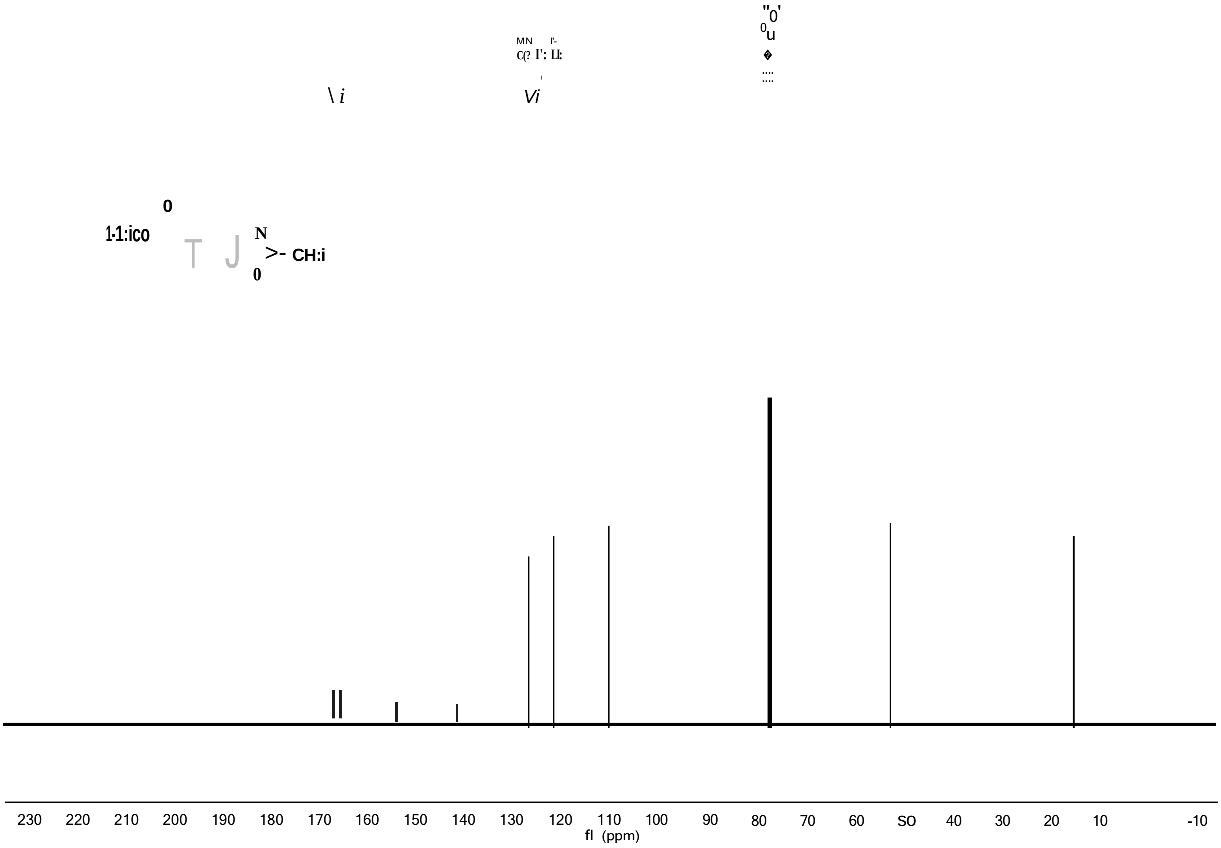

*Methyl 2-methylbenzo[*d*]oxazole-6-carboxylate (**2f**)*

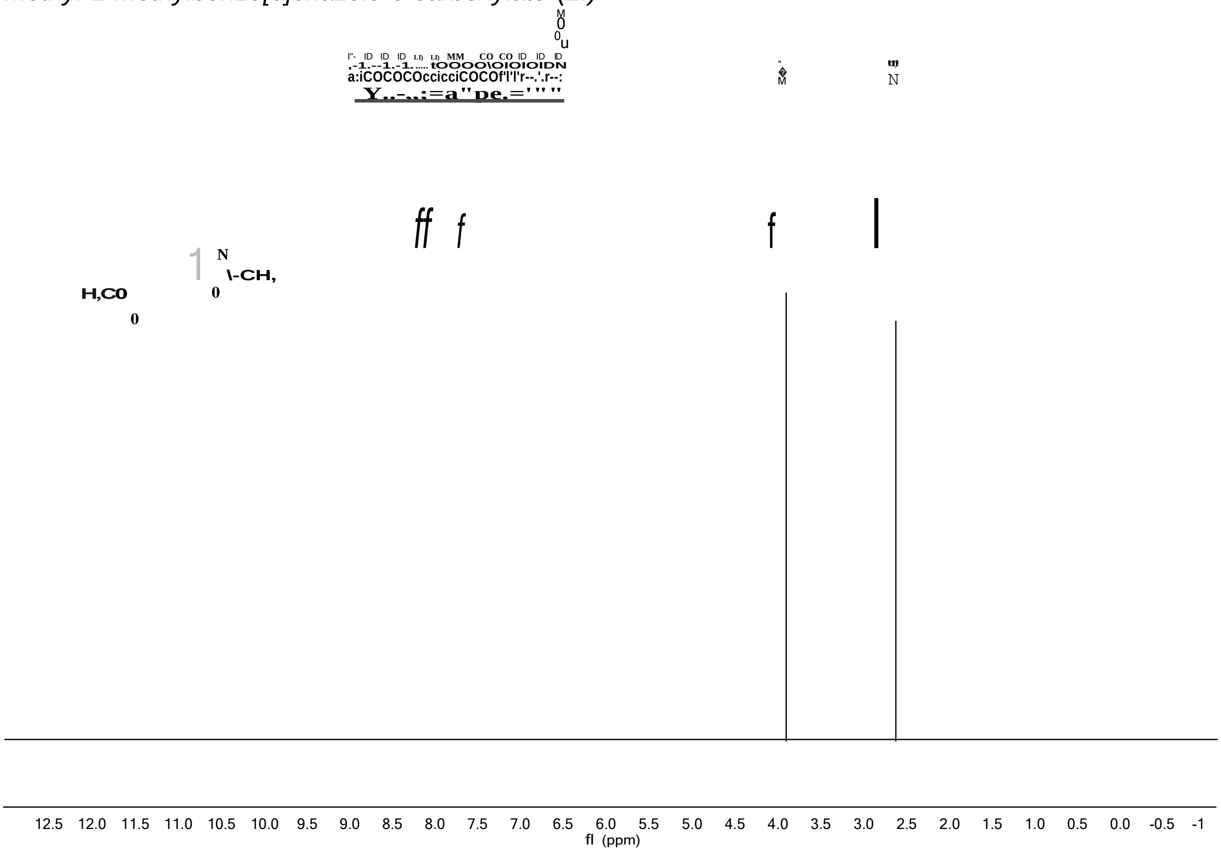

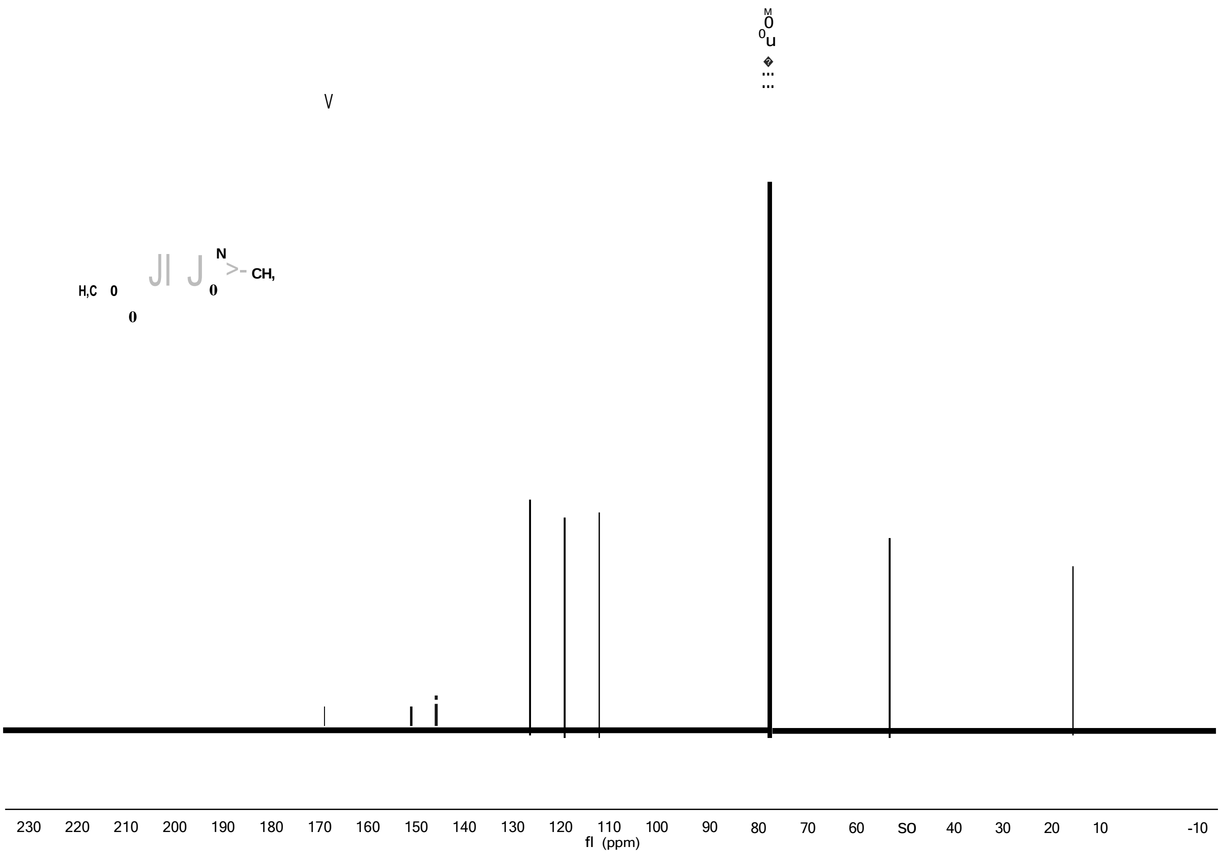

*2-Methyl-5-phenylbenzo[*d*]oxazole* (**2g**)

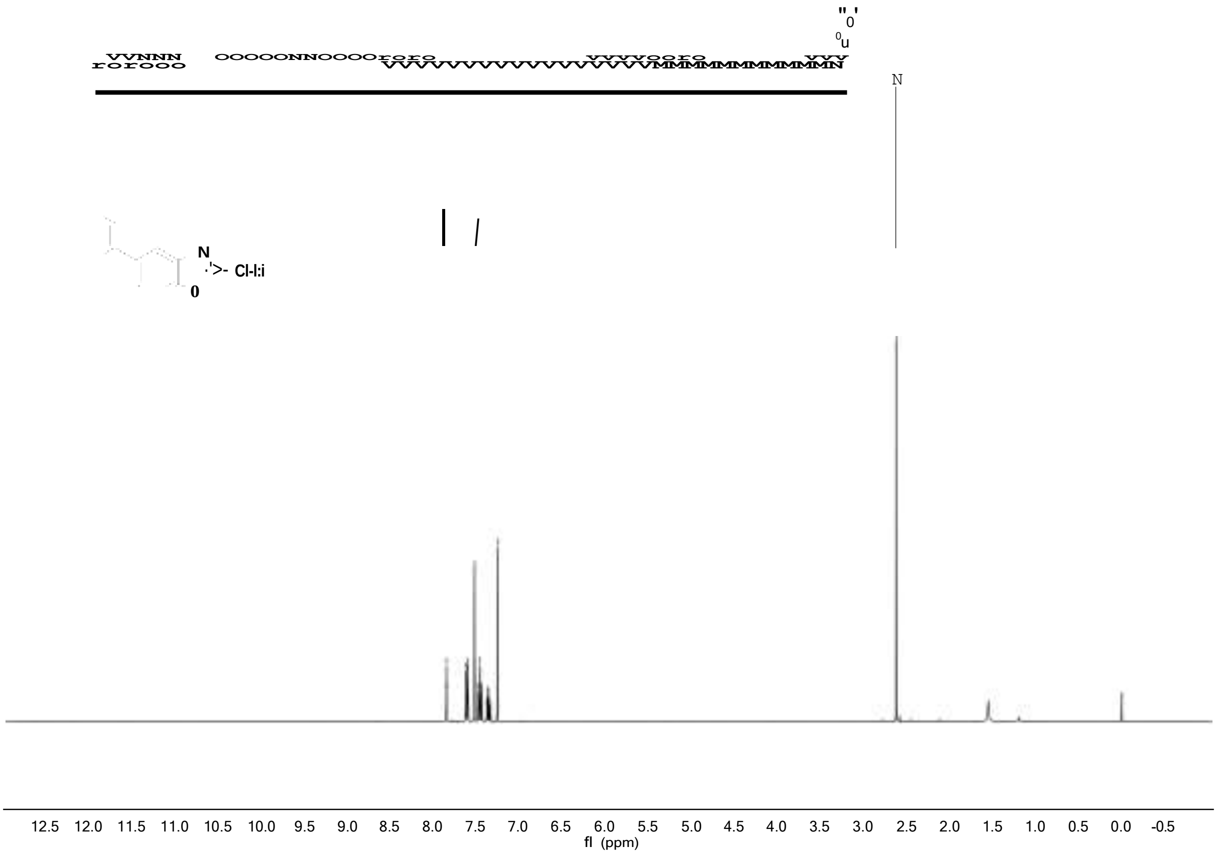

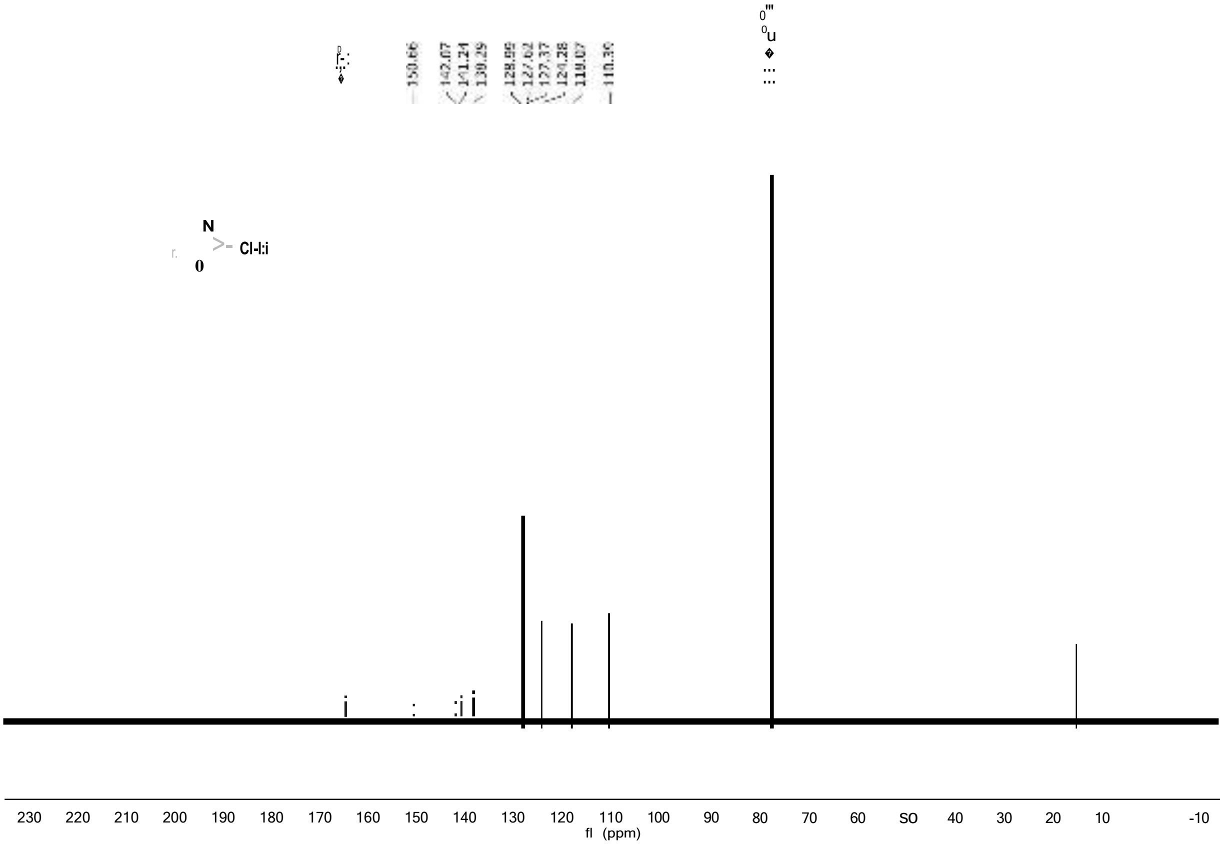

*2-Methyl-6-phenylbenzo[*d*]oxazole (**2h**)*

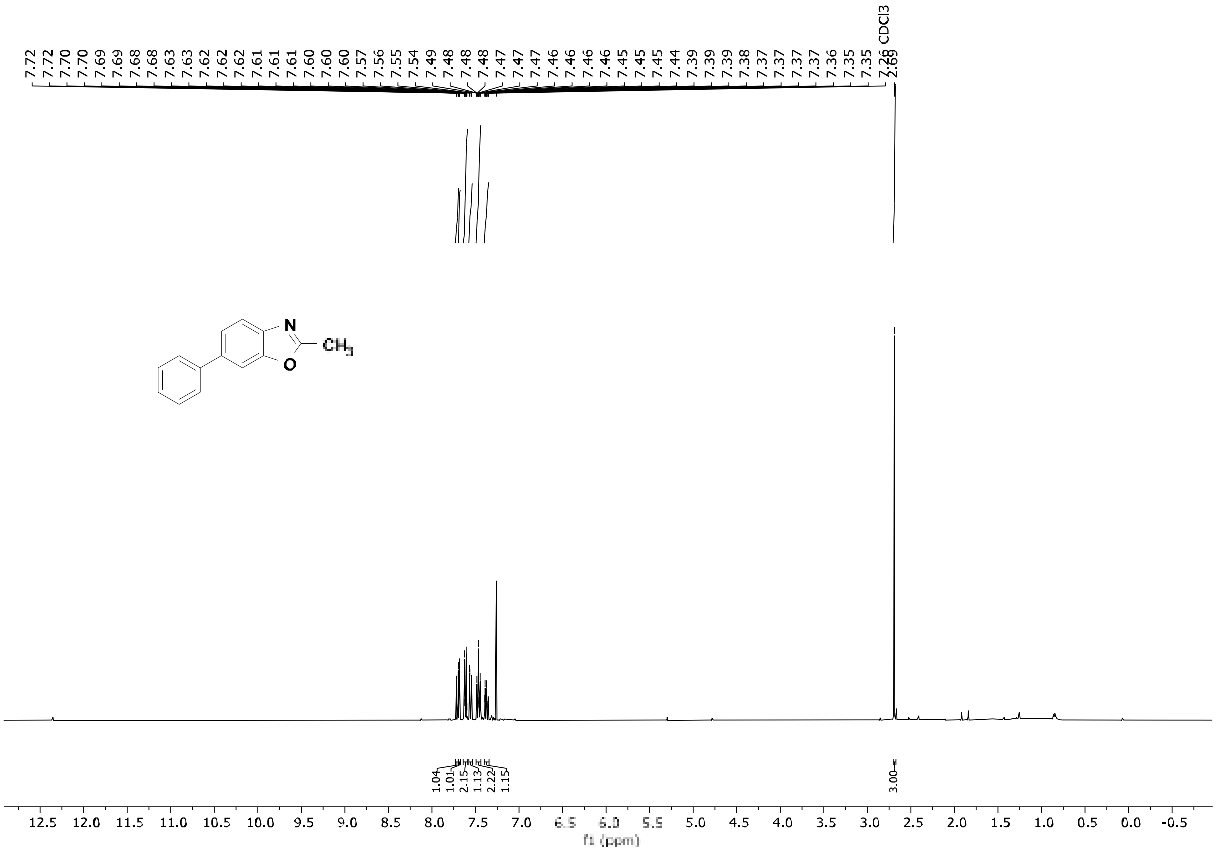

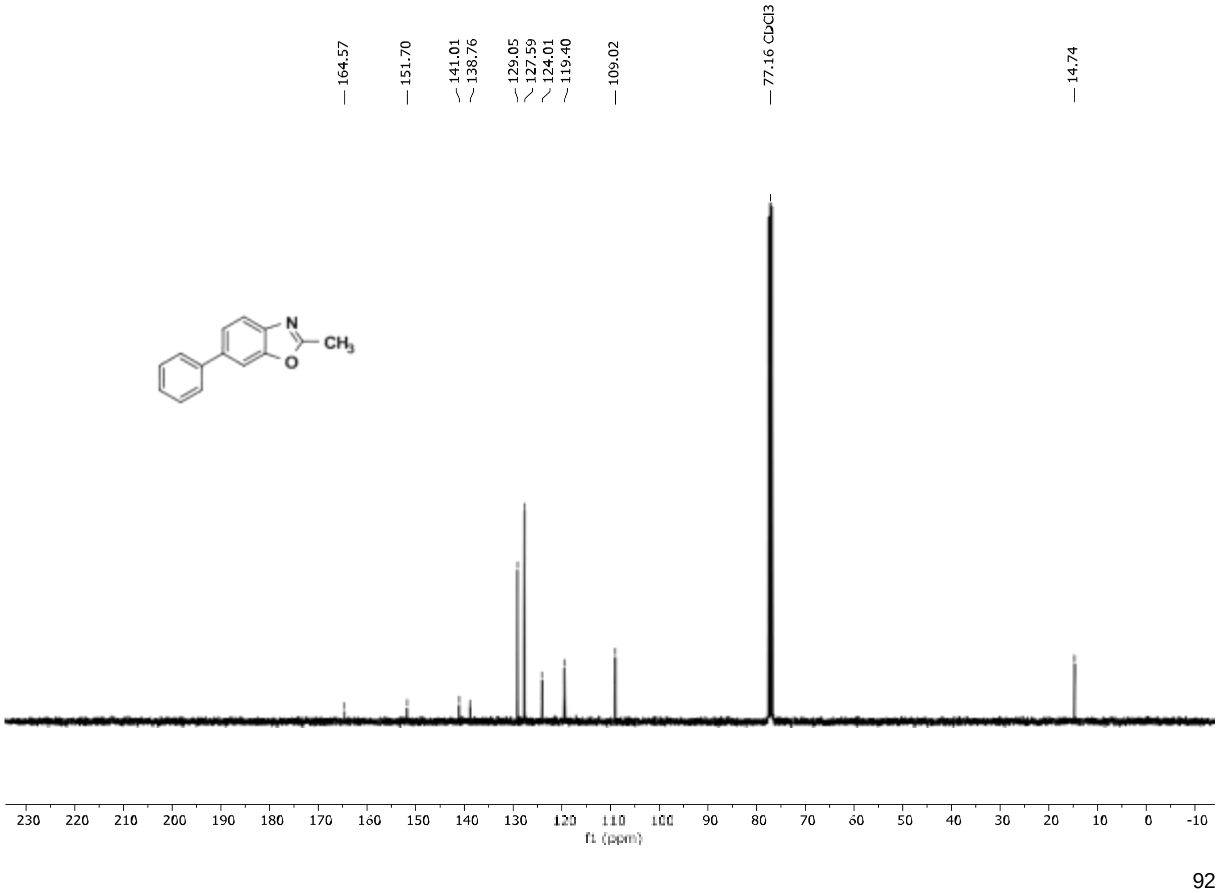

*6-(3-Chlorophenyl)-2-methylbenzo[*d*]oxazole* (***2i***)

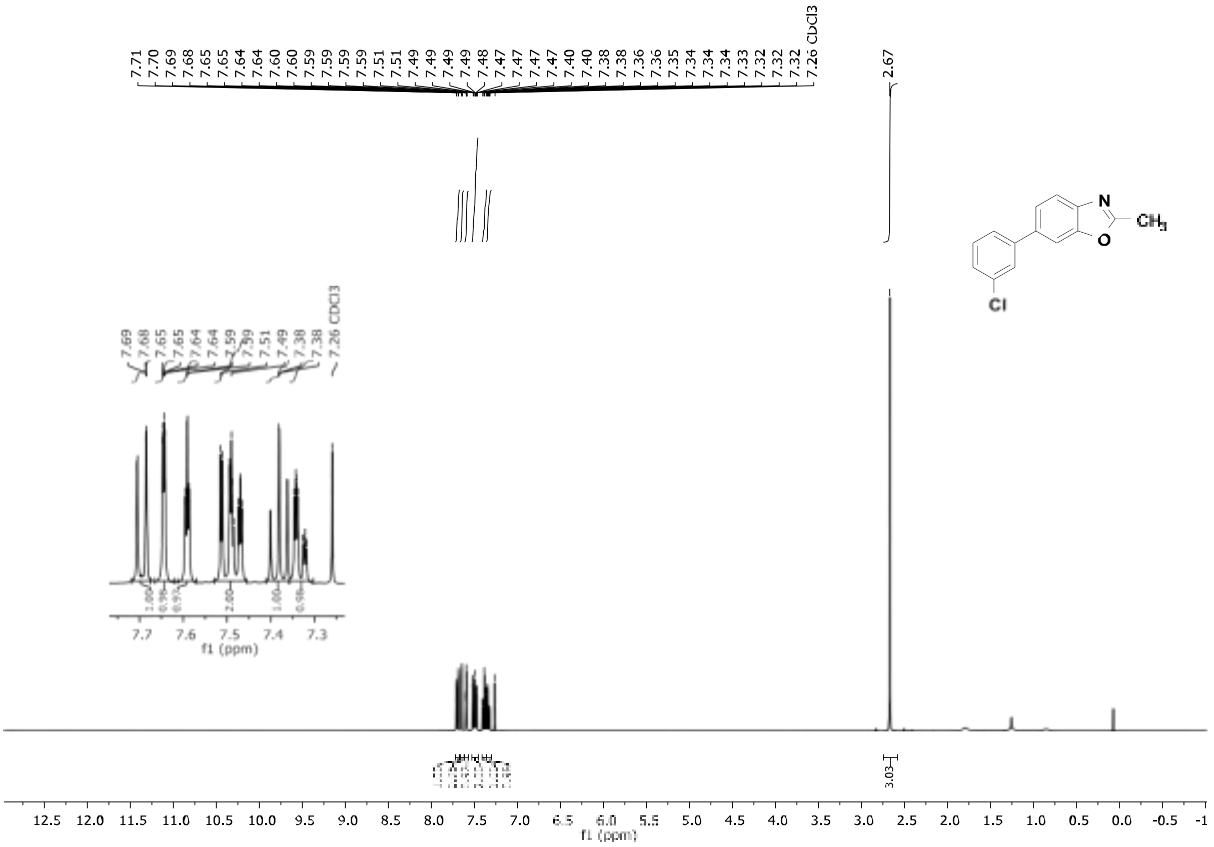

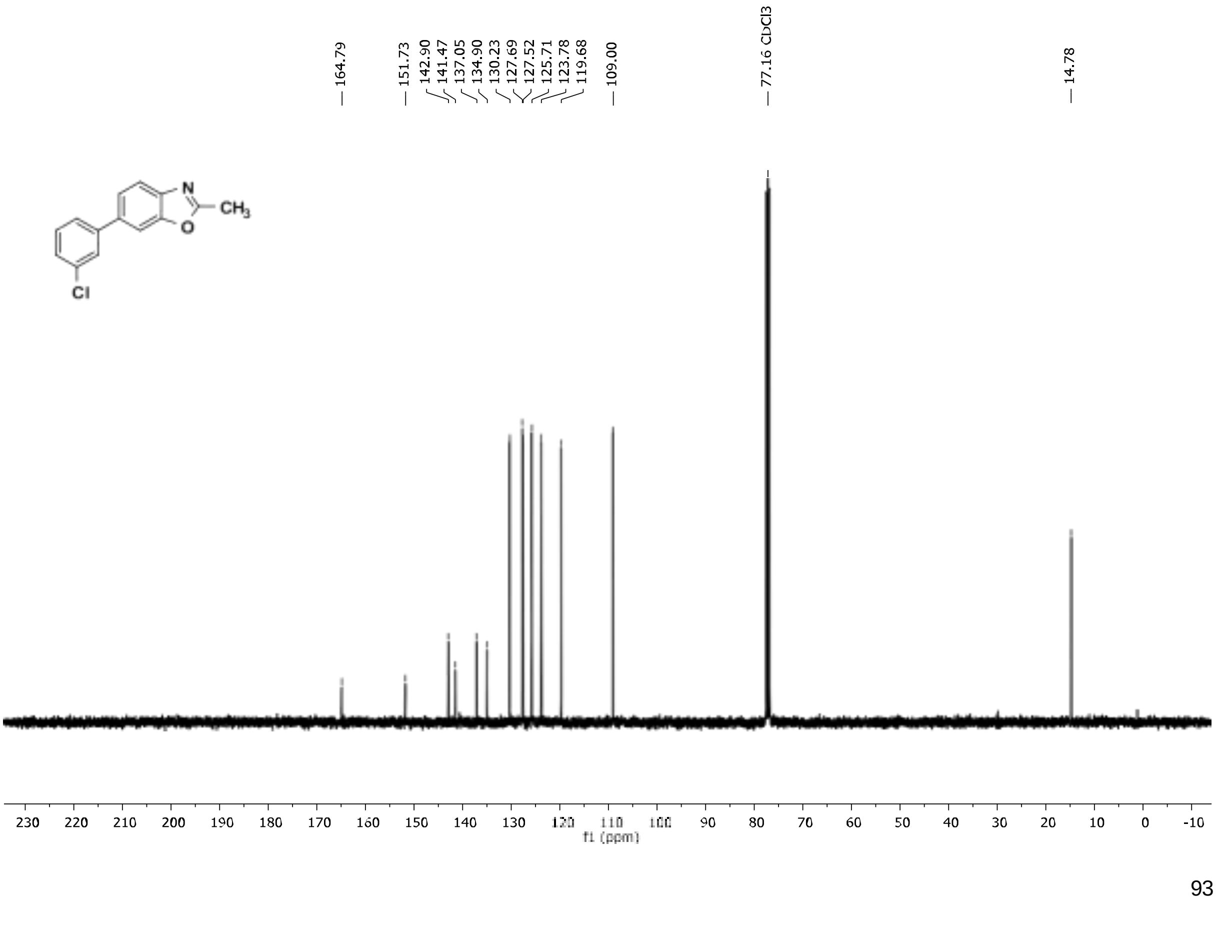

6-(4-Chlorophenyl)-2-methylbenzo[d]oxazole (***2j***)

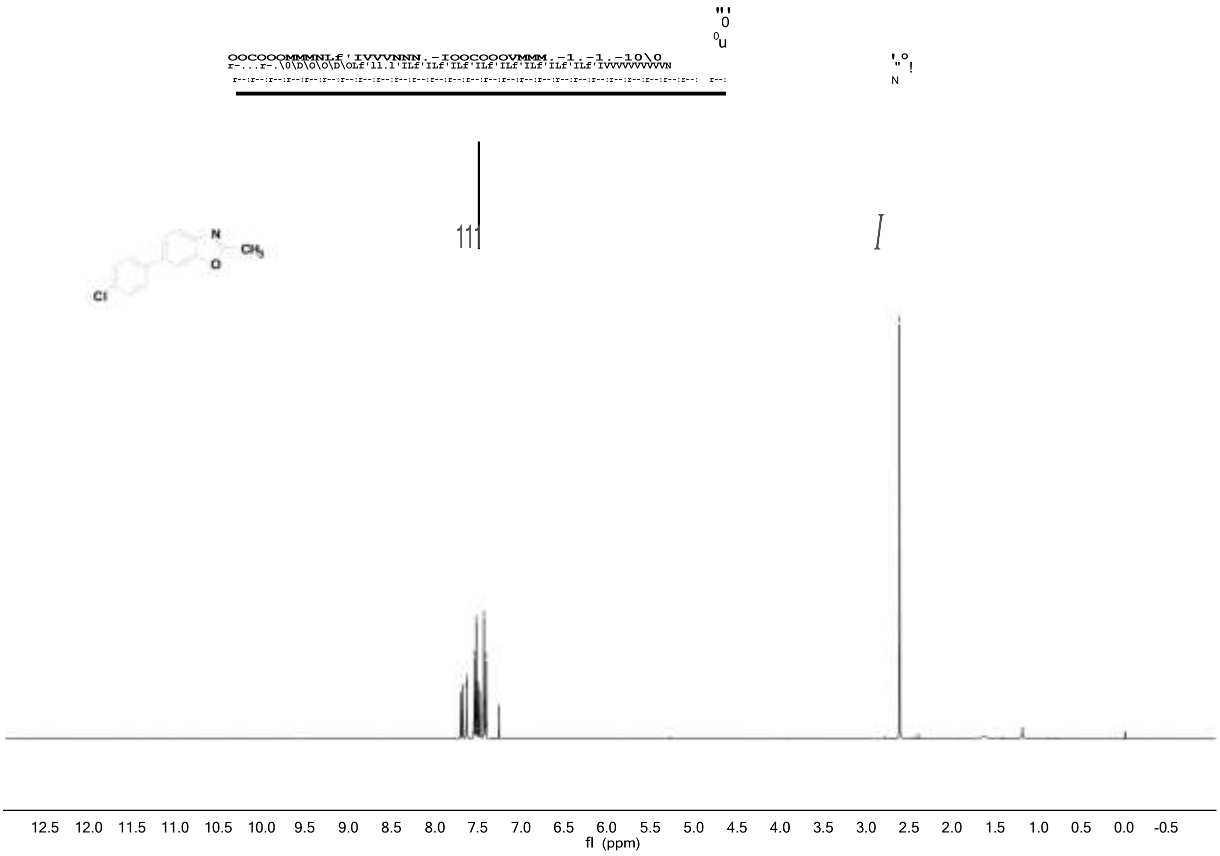

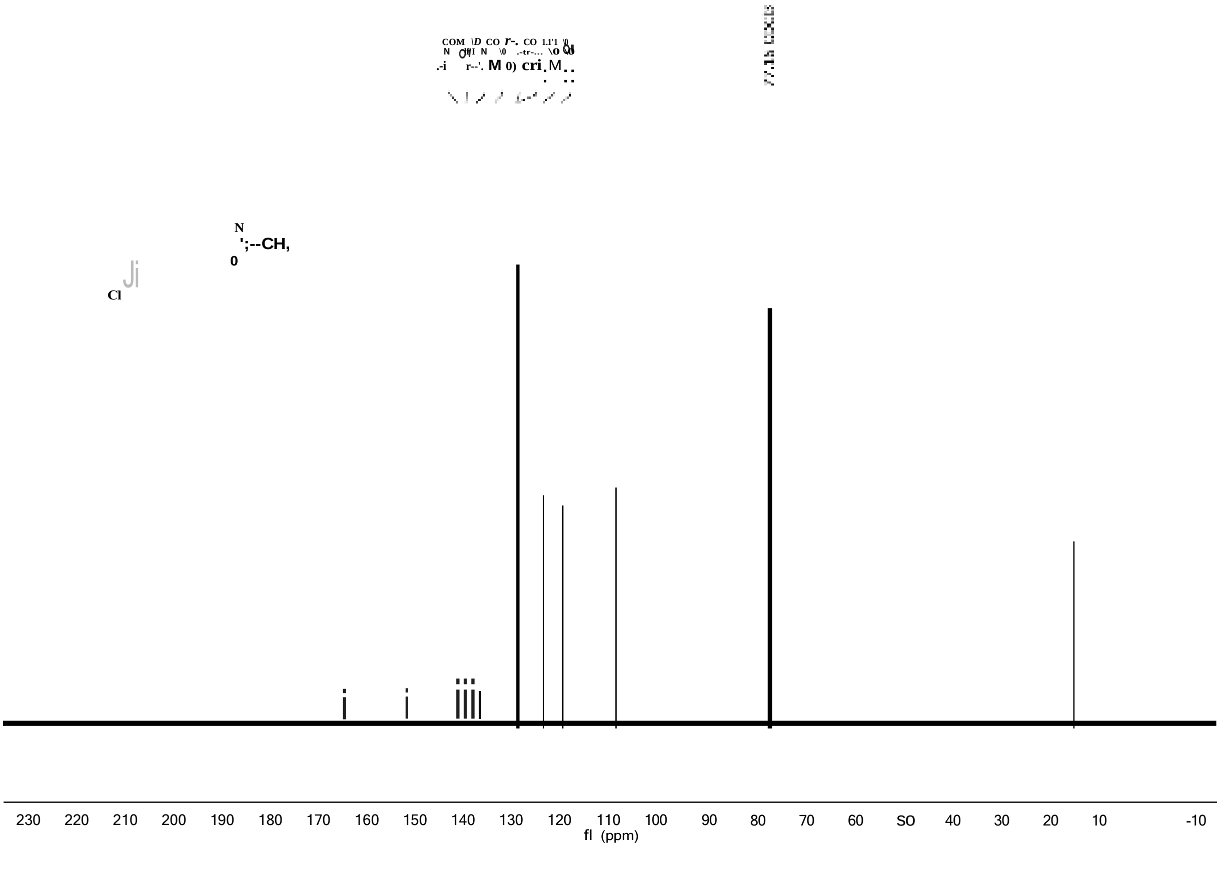

*2-Methyl-6-(2-(trifluoromethyl)phenyl)benzo[*d*]oxazole* ***(2k)***

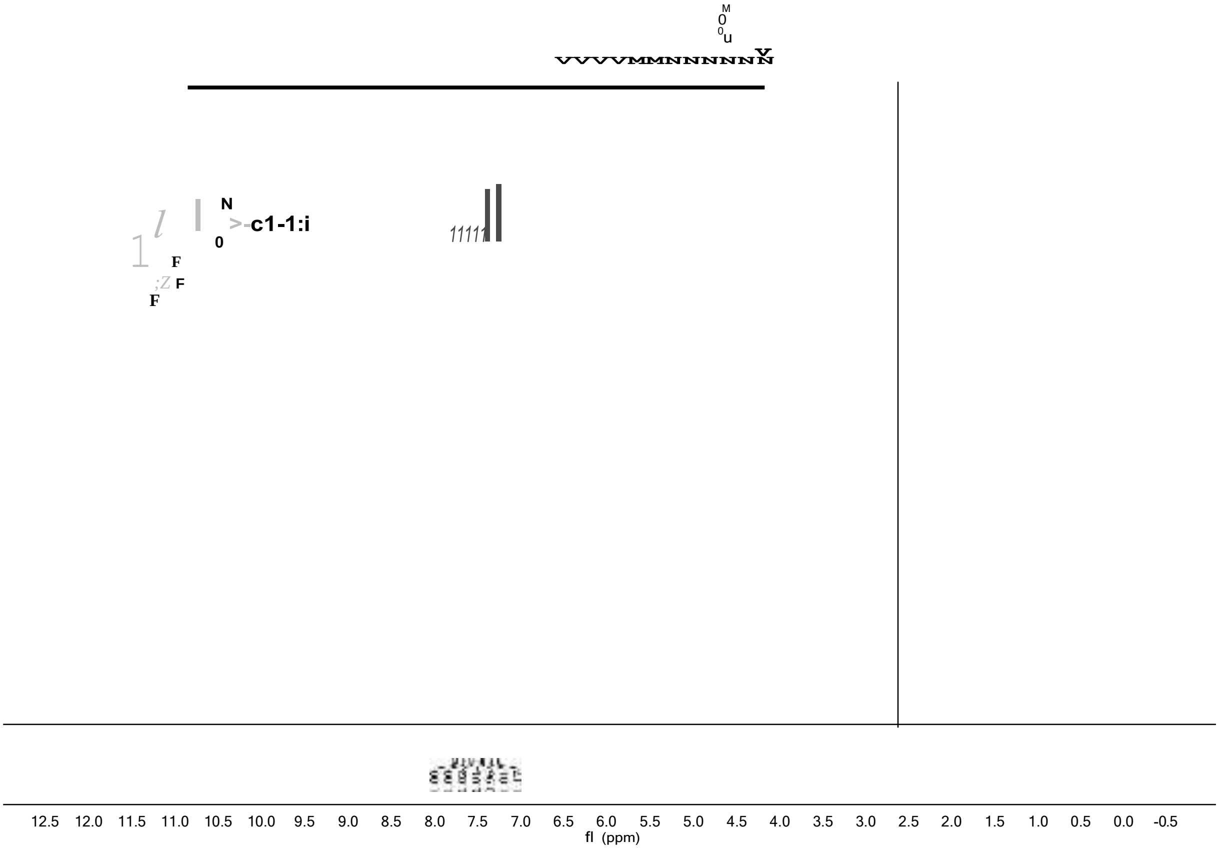

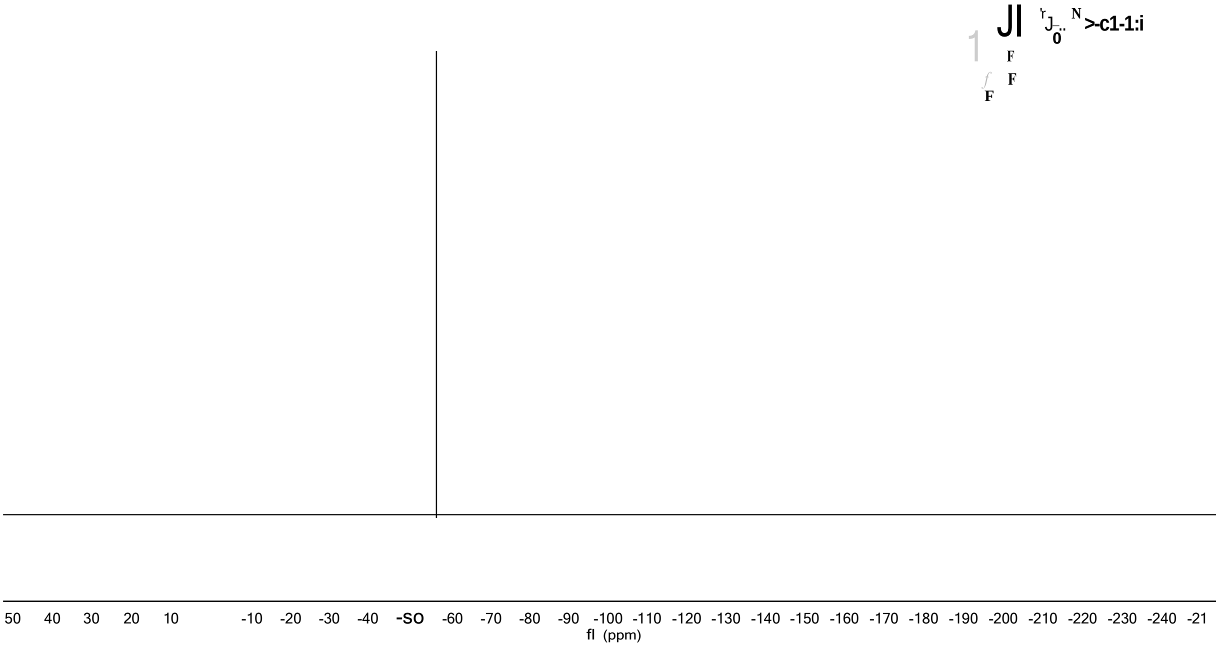

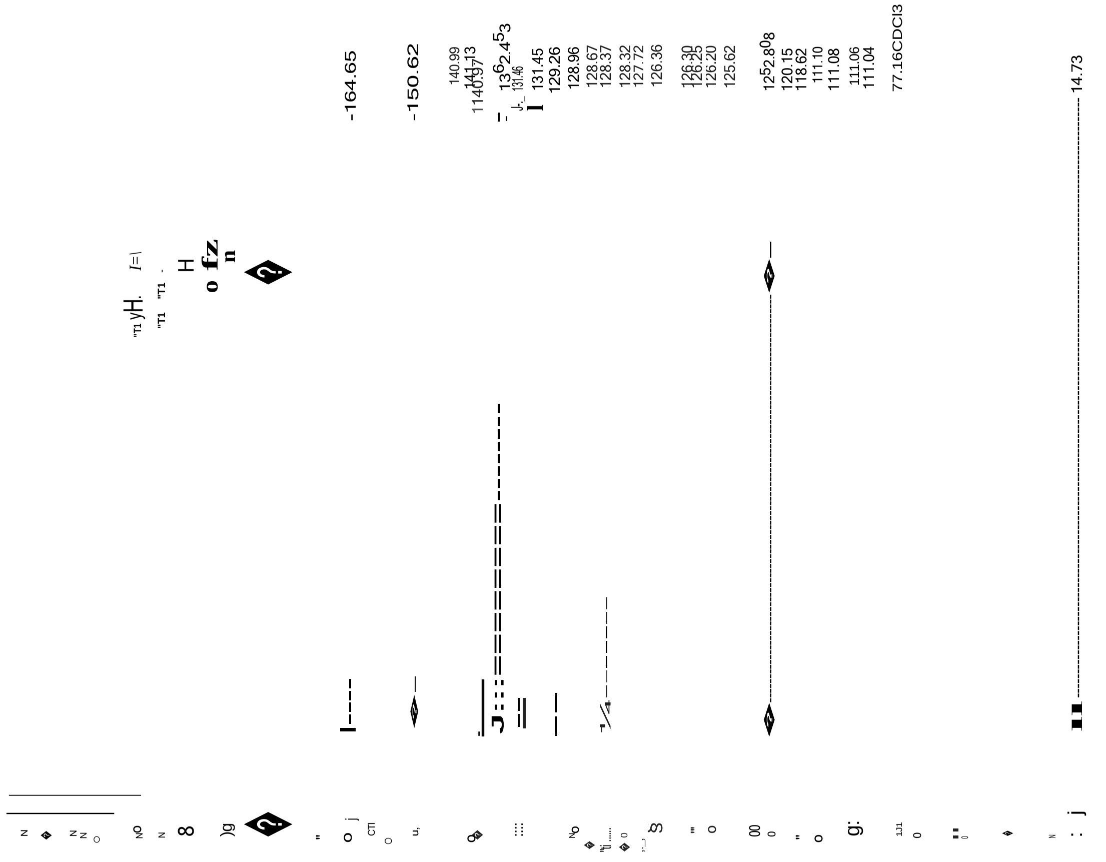

2-Methyl-6-(phenylethynyl)benzo[d]oxazole (**2l**)

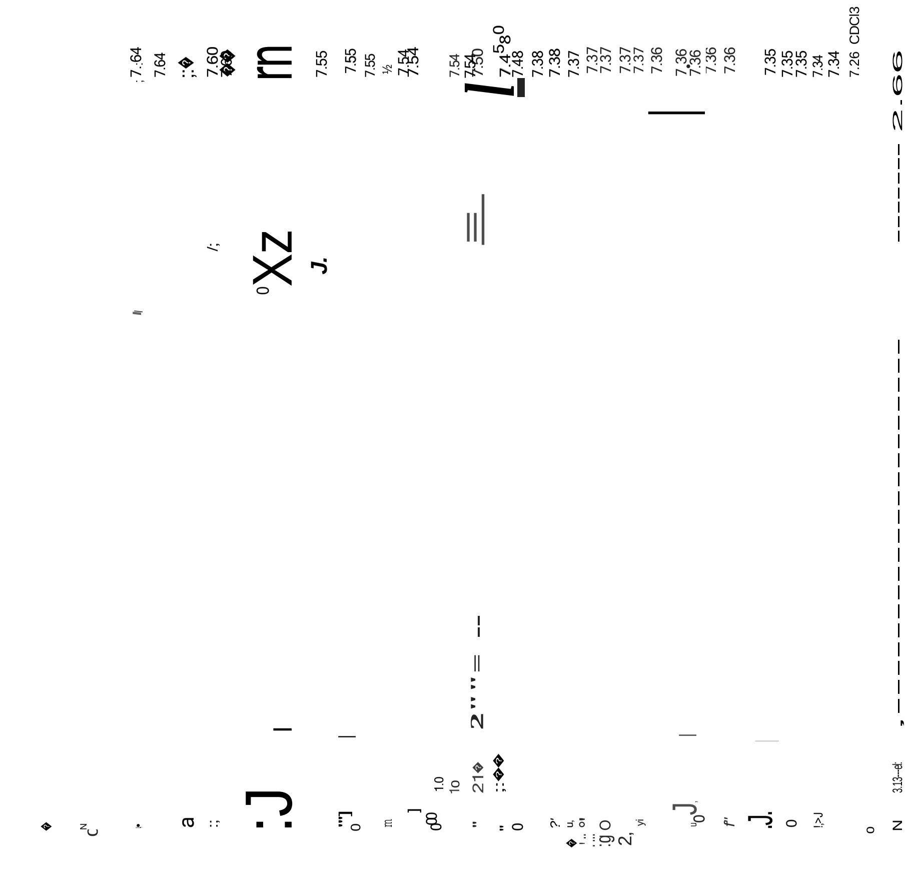

0

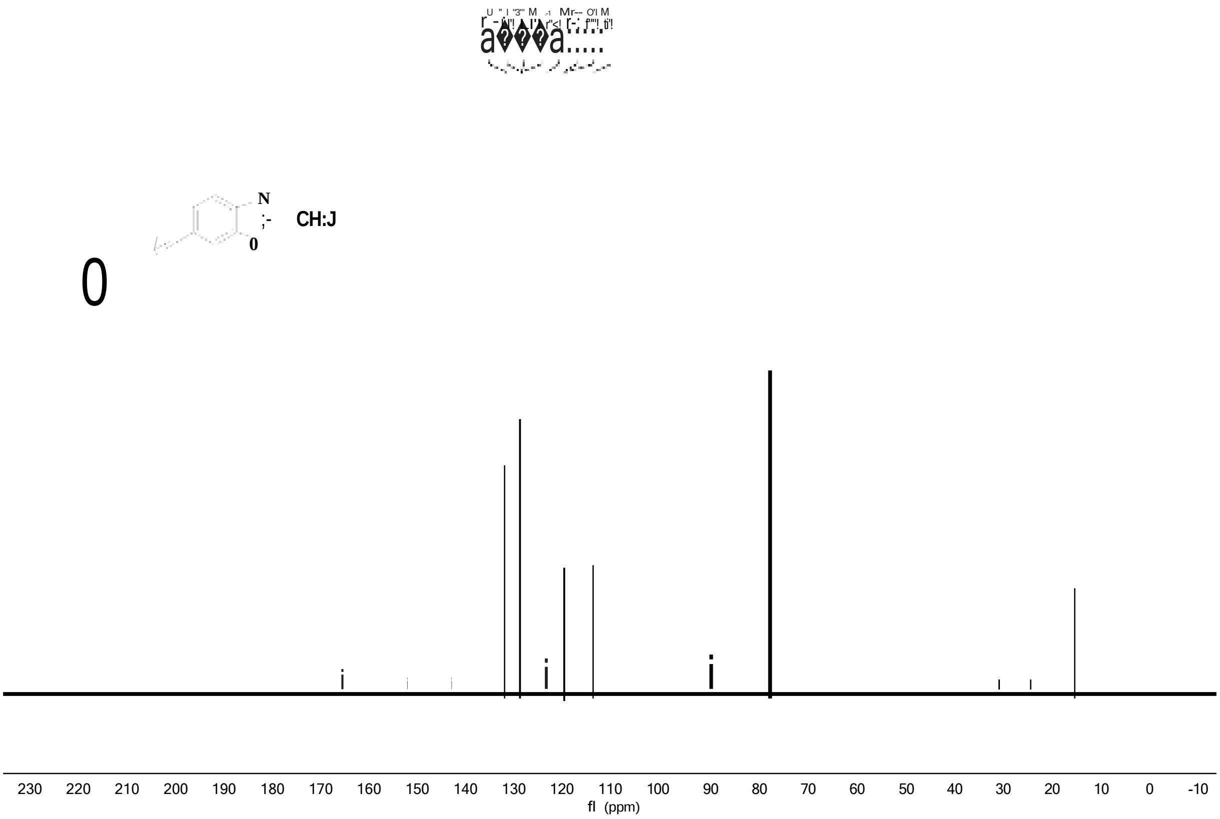
N
0
CH:J
230 220 210 200 190 180 170 160 150 140 130 120 110 100 90 80 70 60 50 40 30 20 10 0 -10
fl (ppm)

NMR Spectra of Benzisoxazoles from 3-Substituted Benzofurans

*3-Methylbenzo[d]isoxazole (**3a**)*

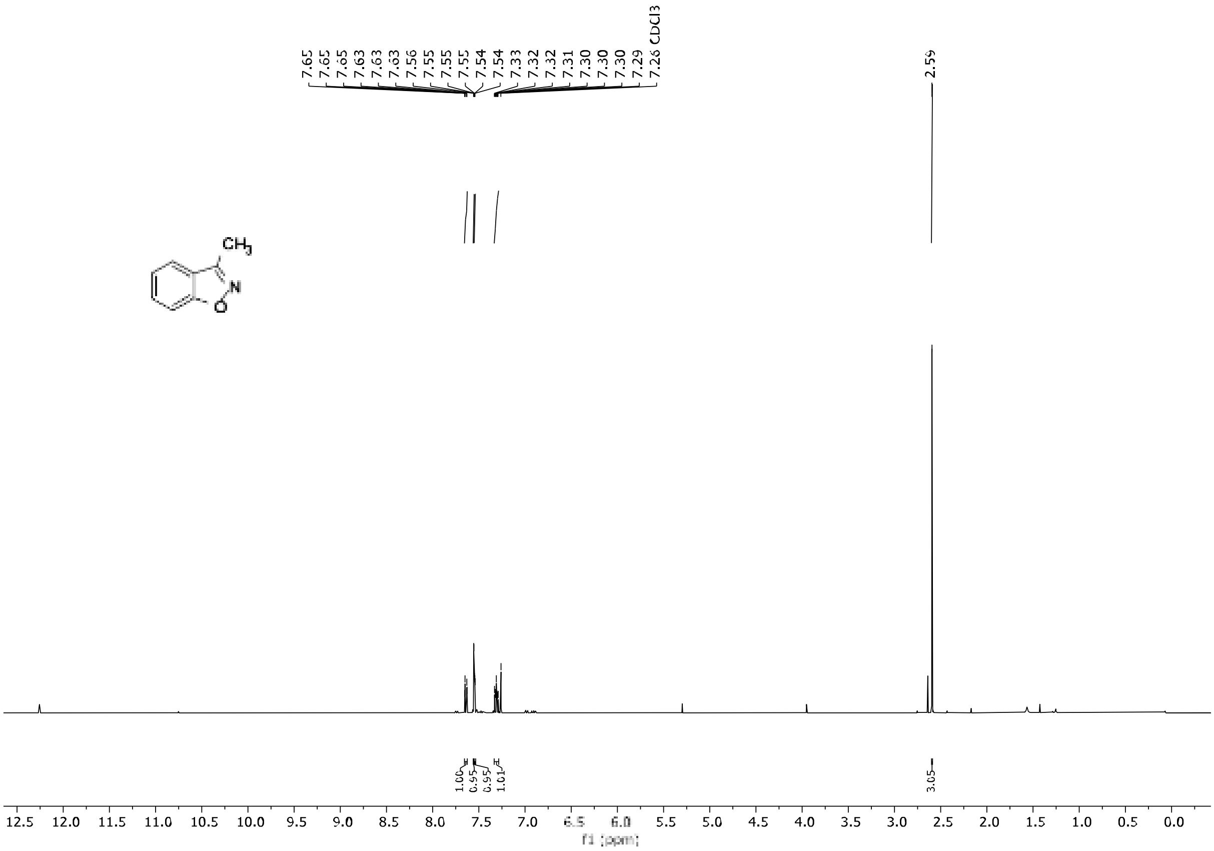

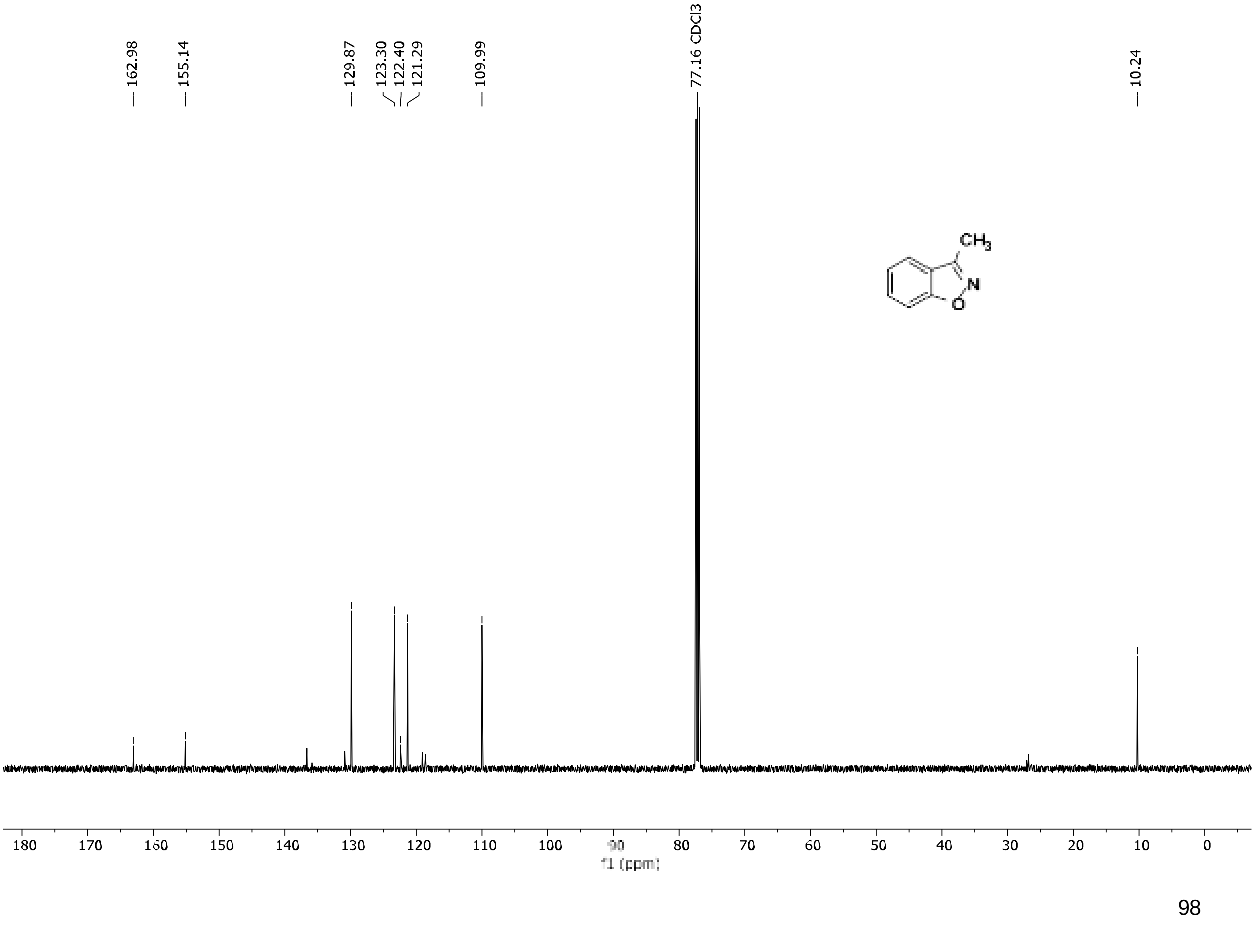

3-Phenylbenzo[d]isoxazole (**3b**)

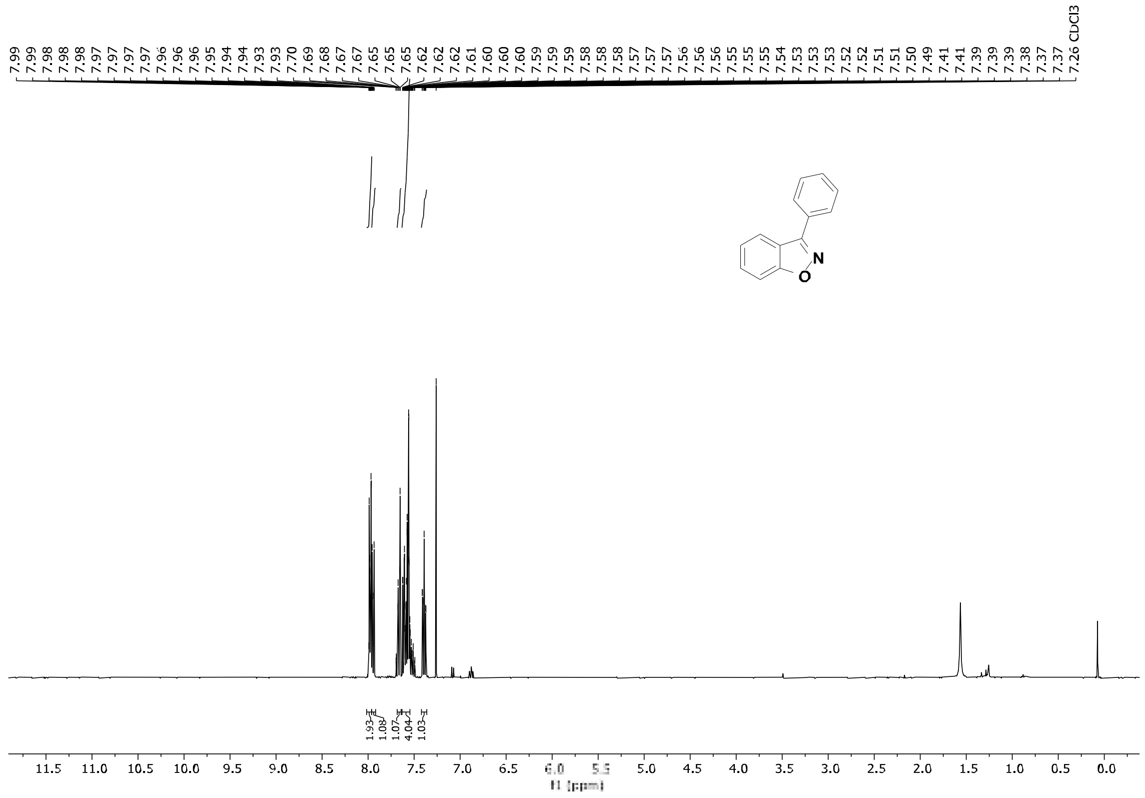

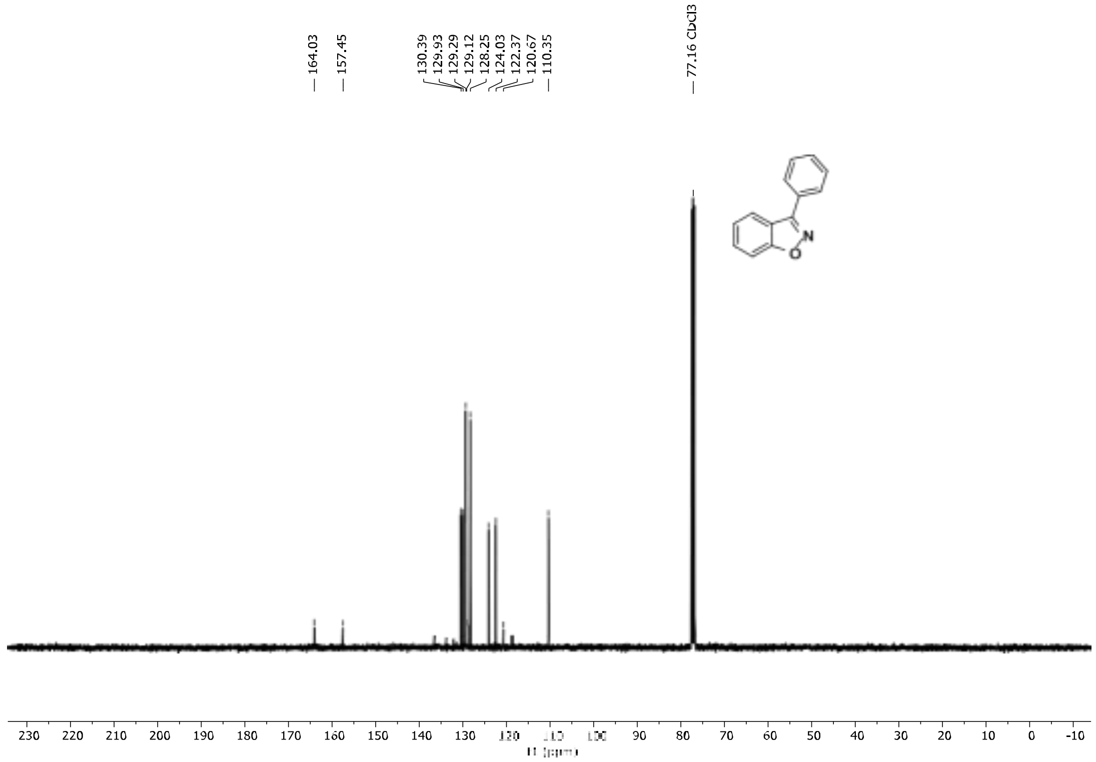

6-Bromo-3-methylbenzo[d]isoxazole (***3c***)

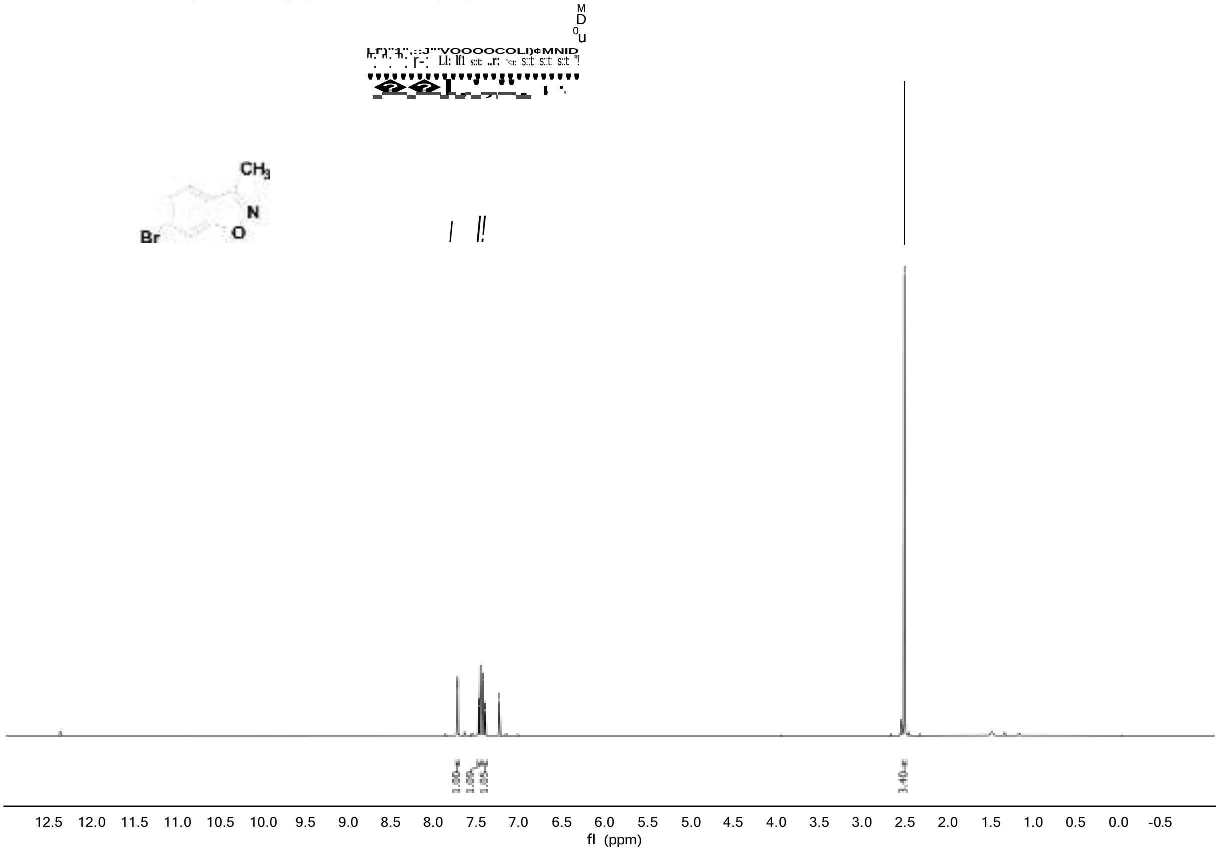

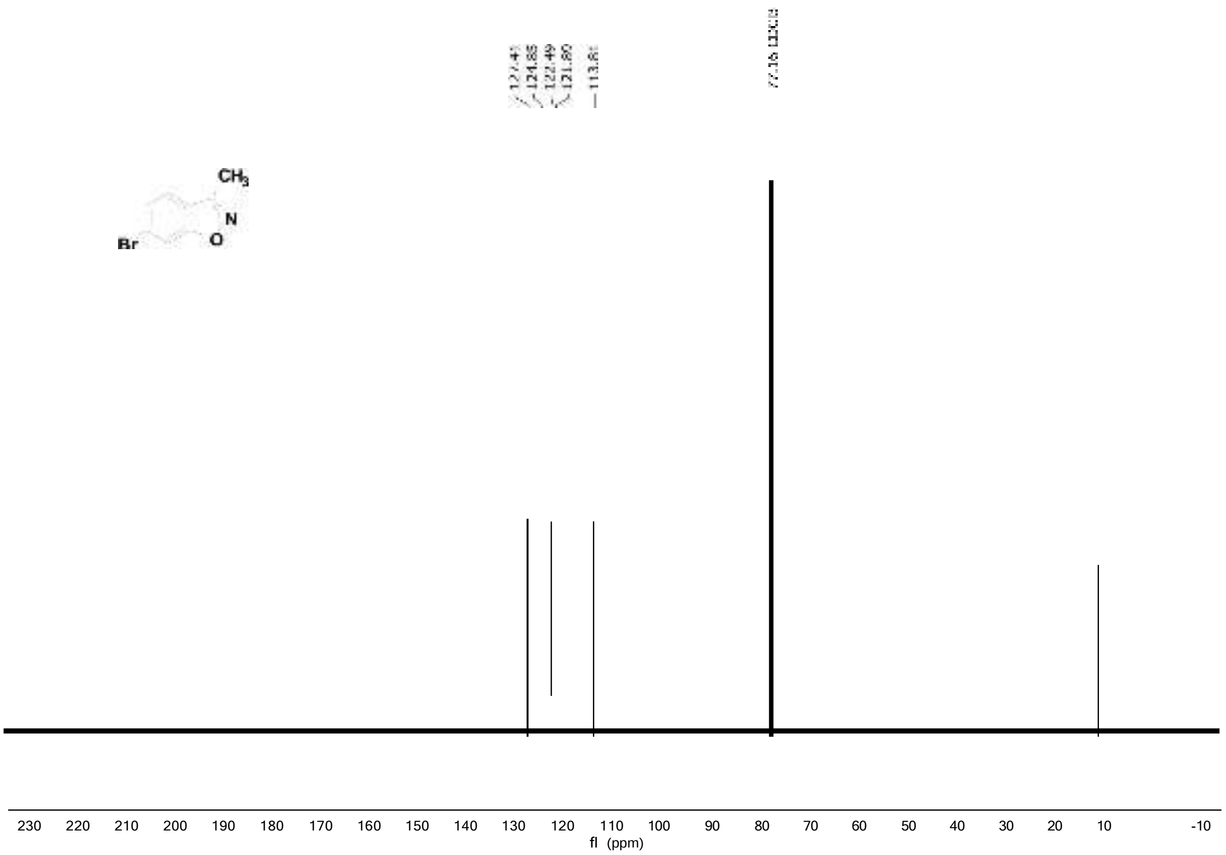

*6-Methoxy-3-methylbenzo[d]isoxazole (**3d**)*

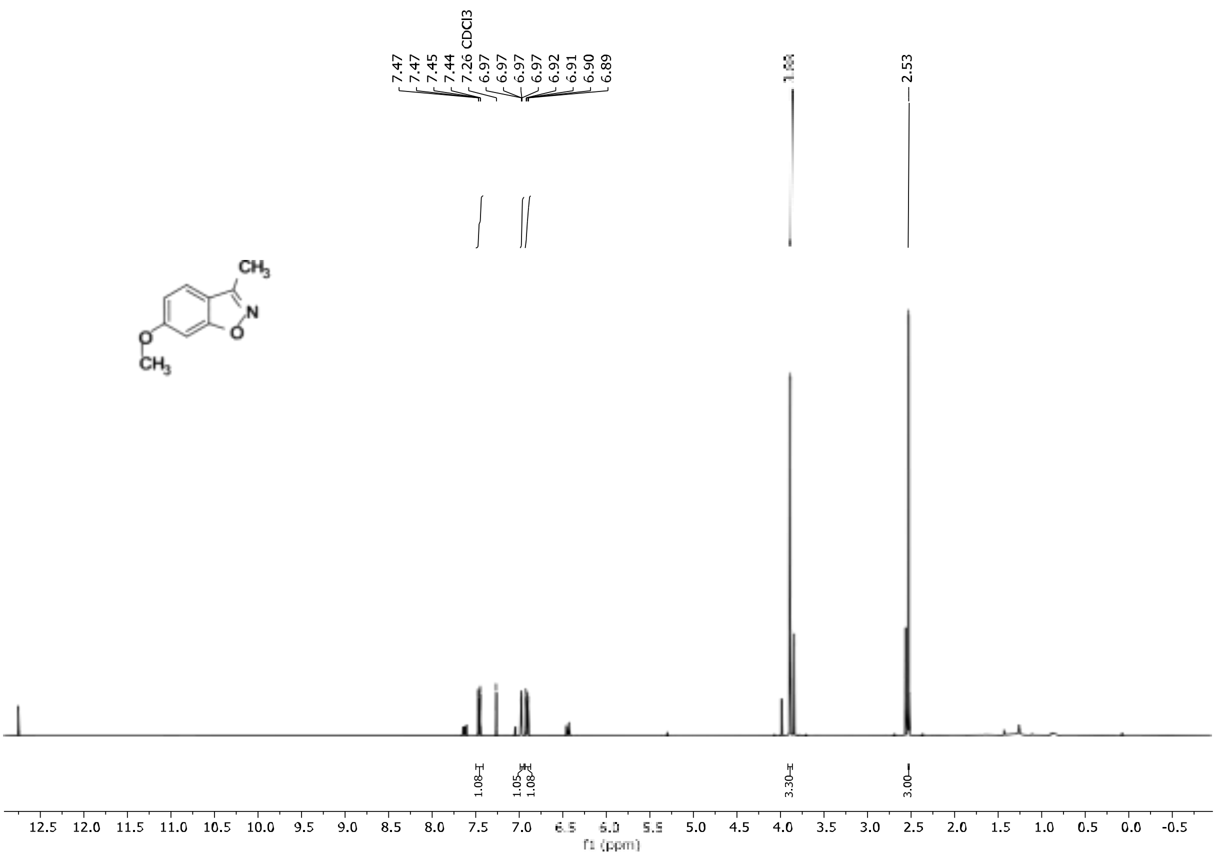

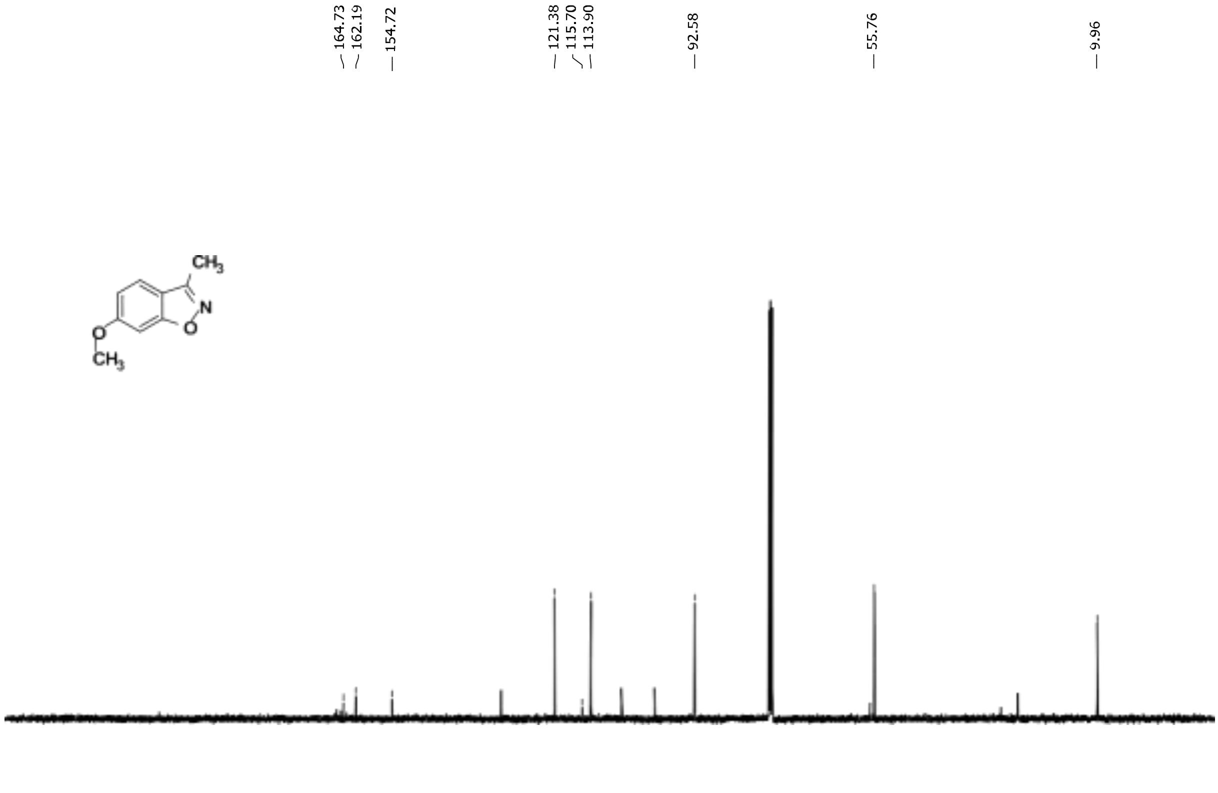

*Methyl 3-methylbenzo[d]isoxazole-5-carboxylate (**3e**)*

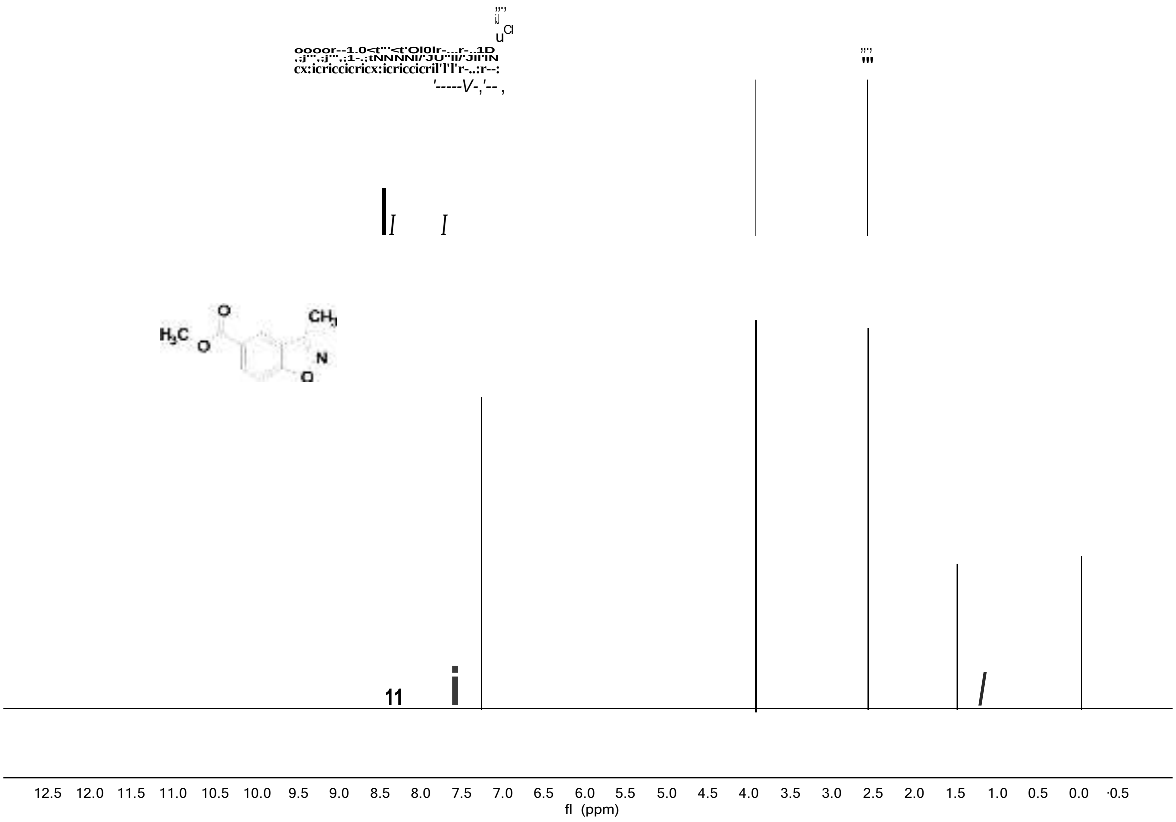

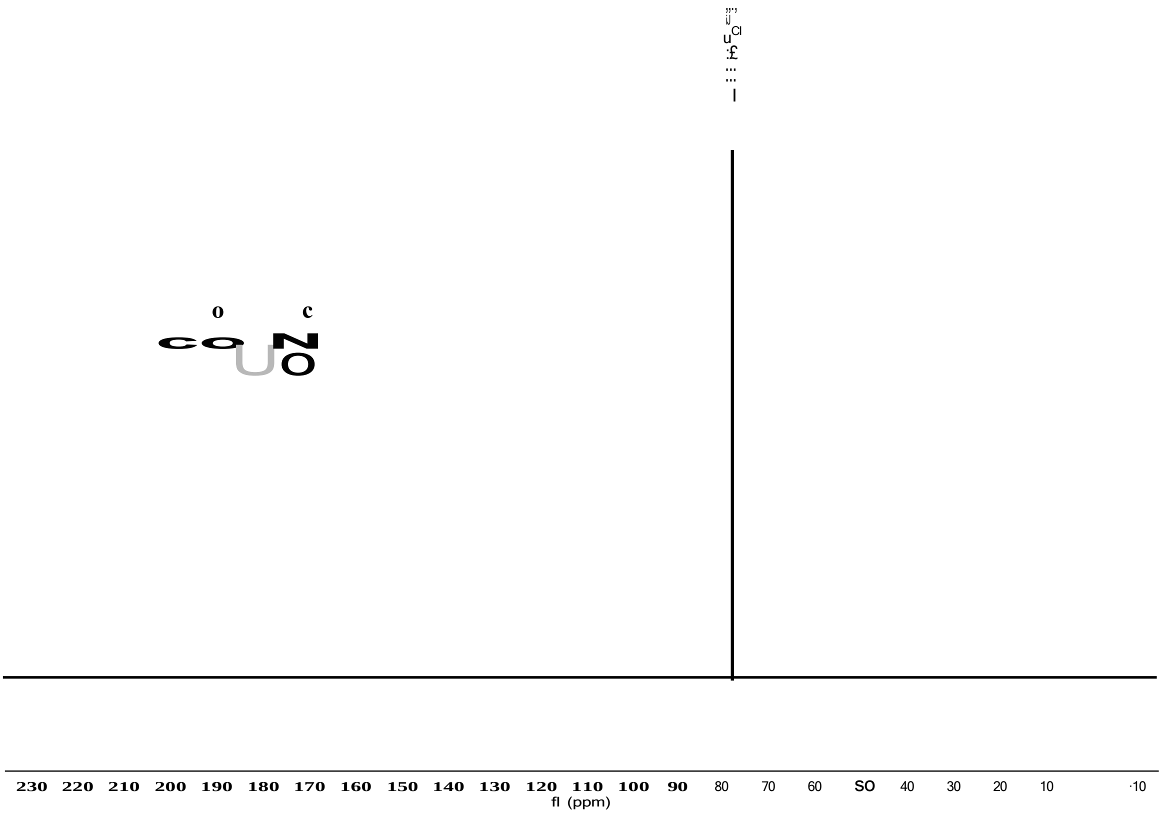

*Methyl 3-methylbenzo[d]isoxazole-6-carboxylate (**3f**)*

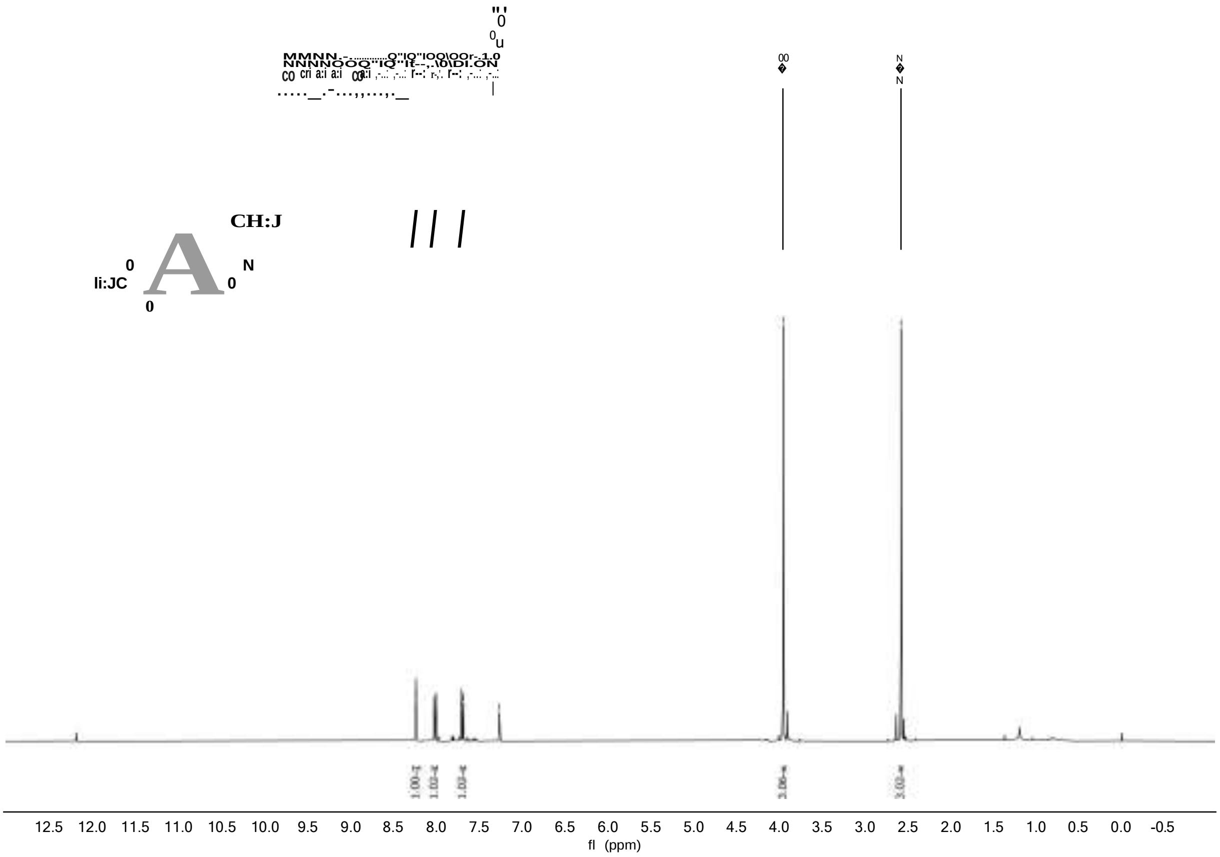

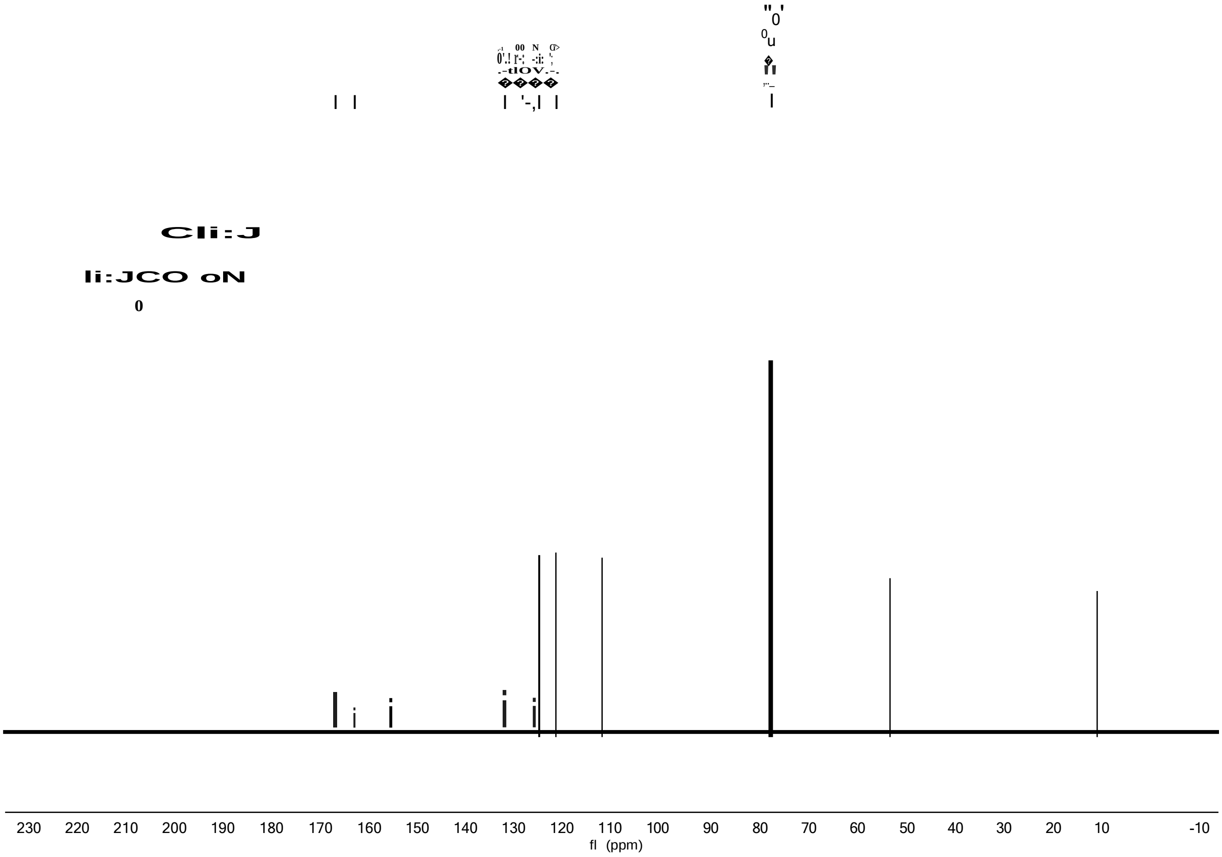

3-Methyl-5-phenylbenzo[d]isoxazole (**3g**)

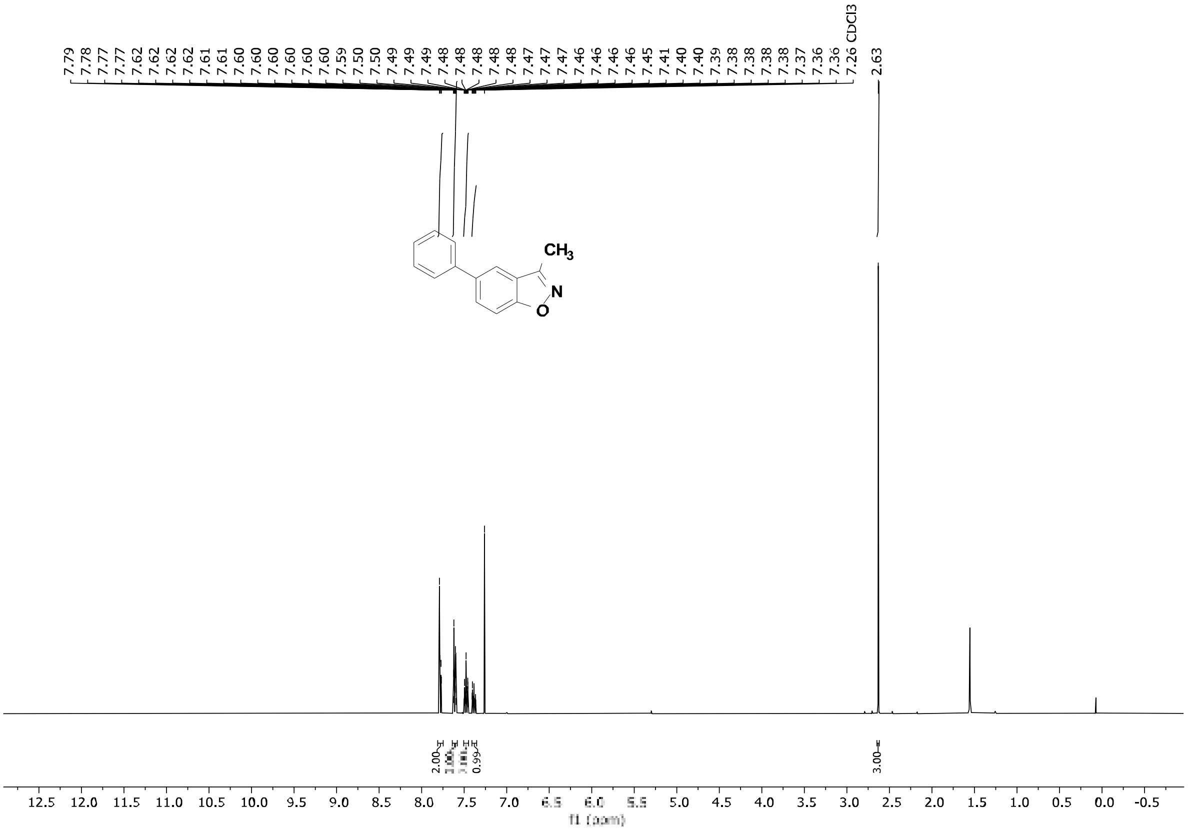

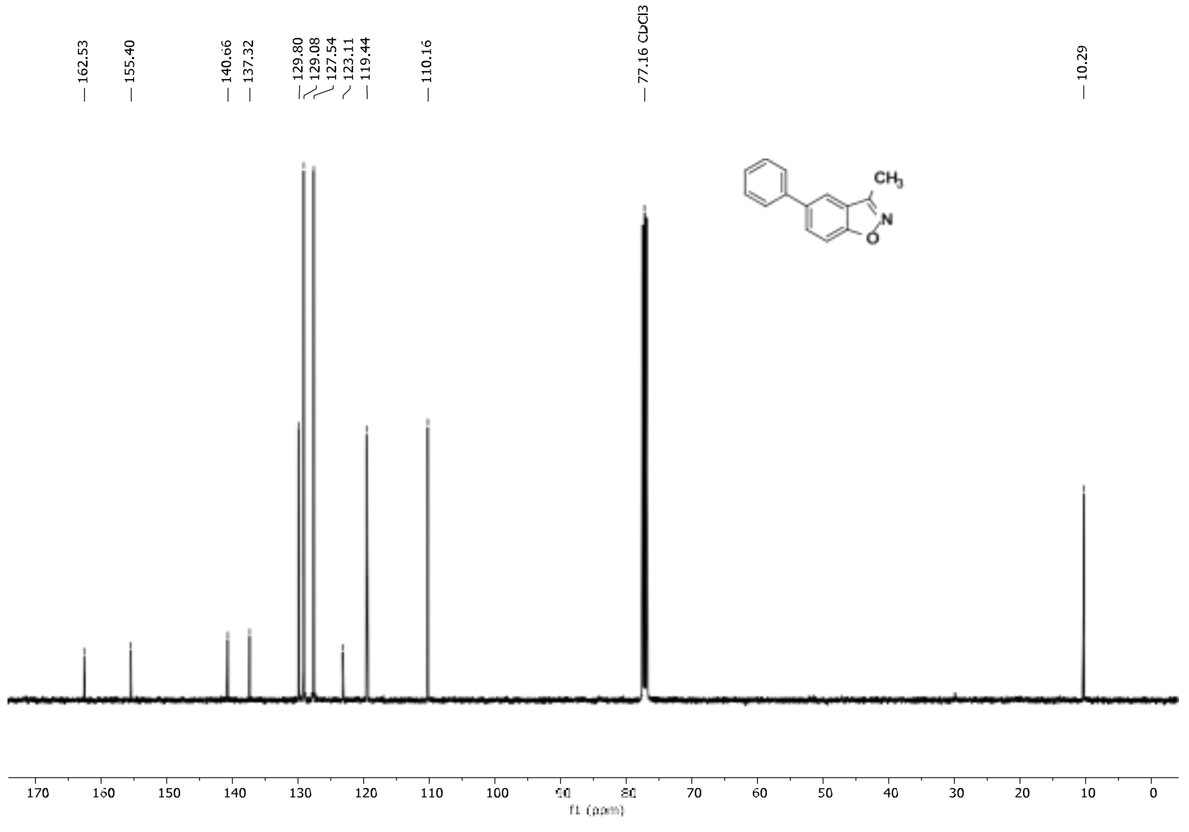

3-Methyl-6-phenylbenzo[d]isoxazole ***(3h)***

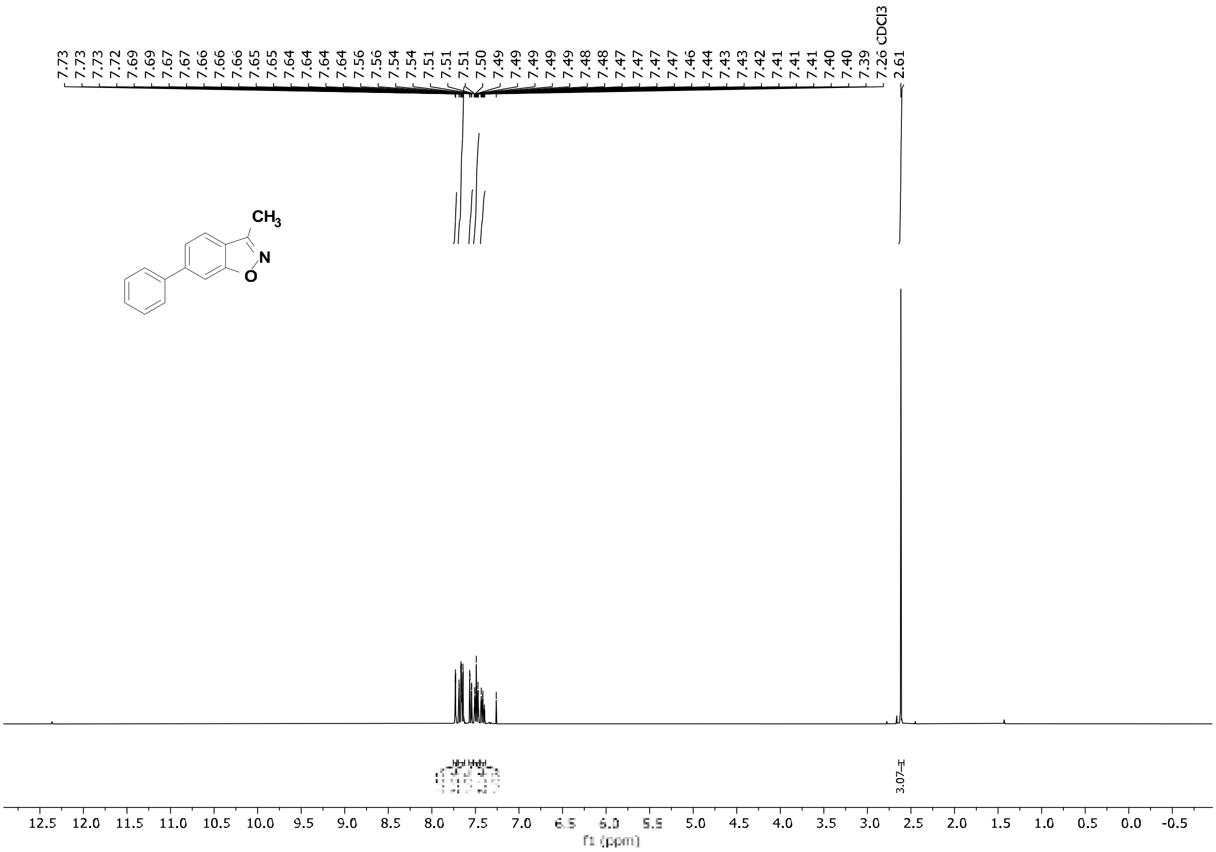

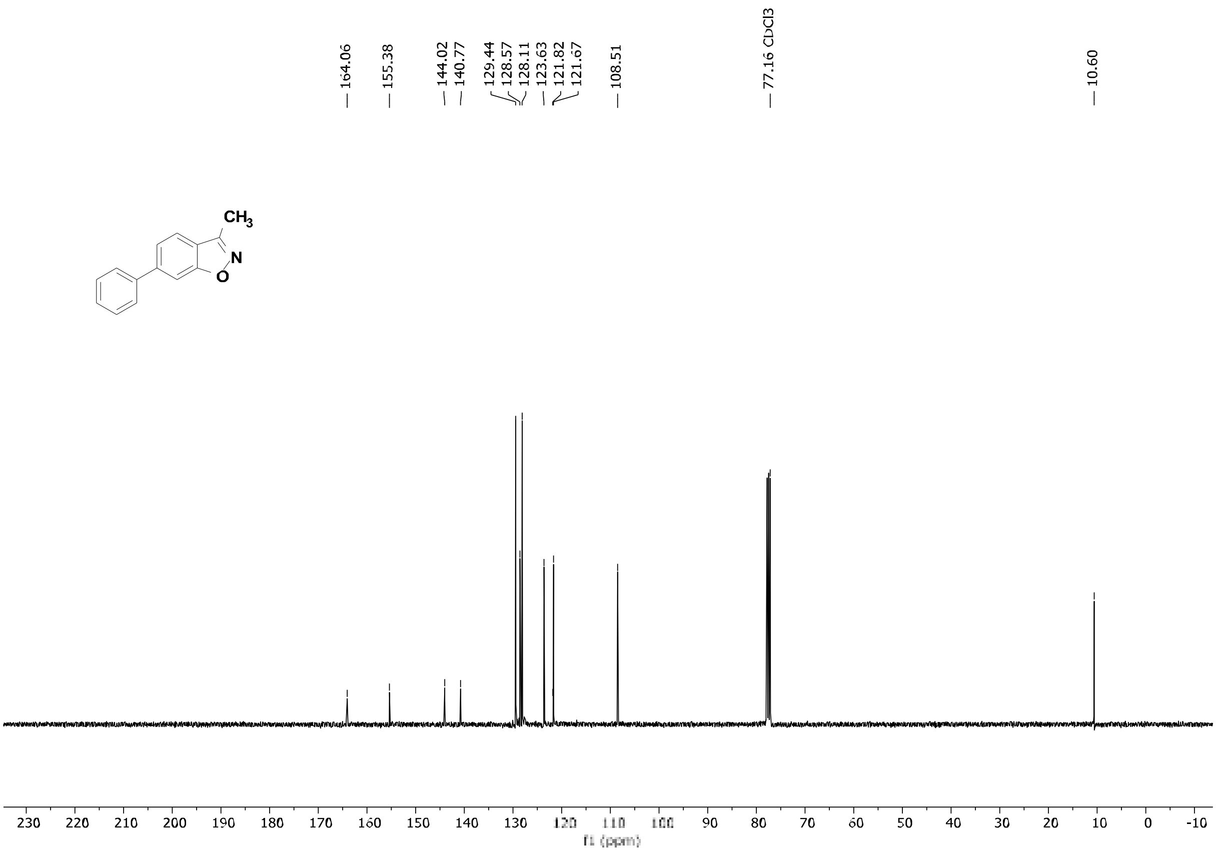

*6-(3-chlorophenyl)-3-methylbenzo[d]isoxazole (**3i**)*

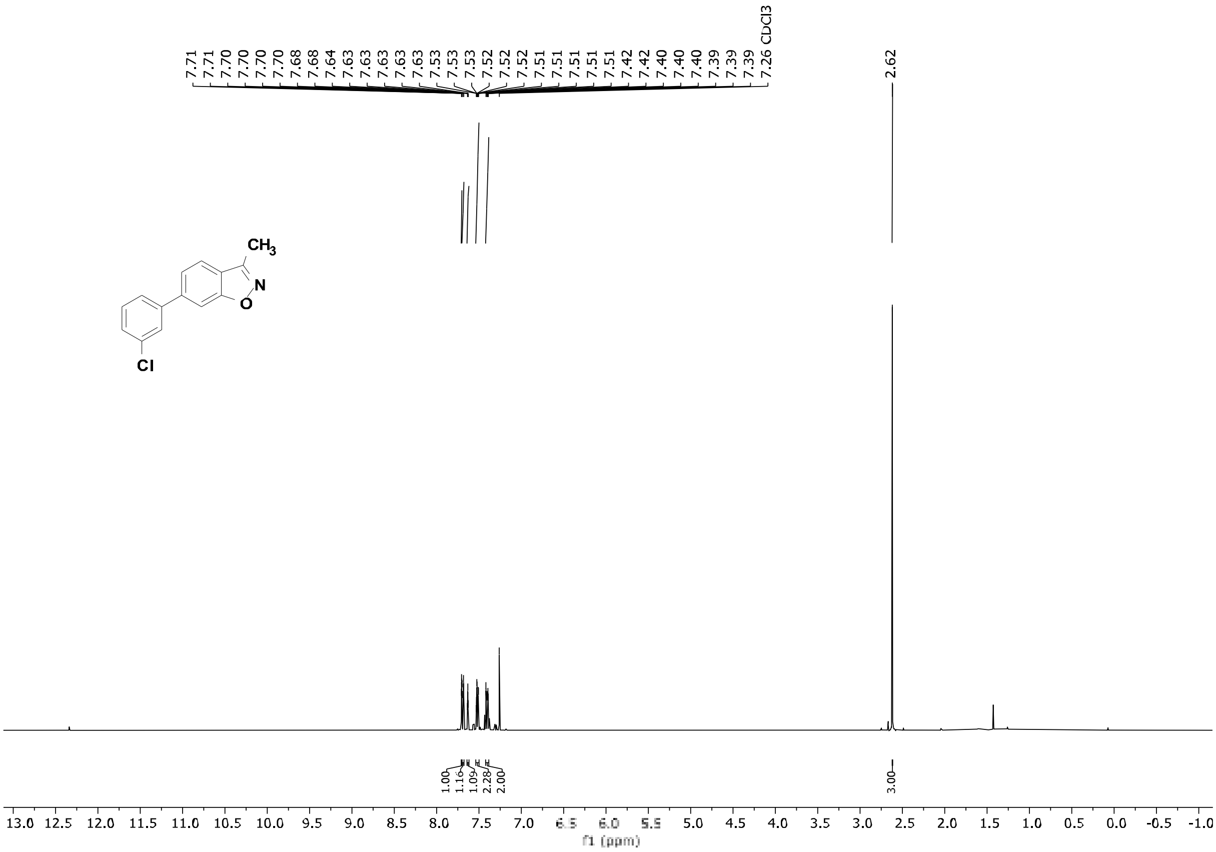

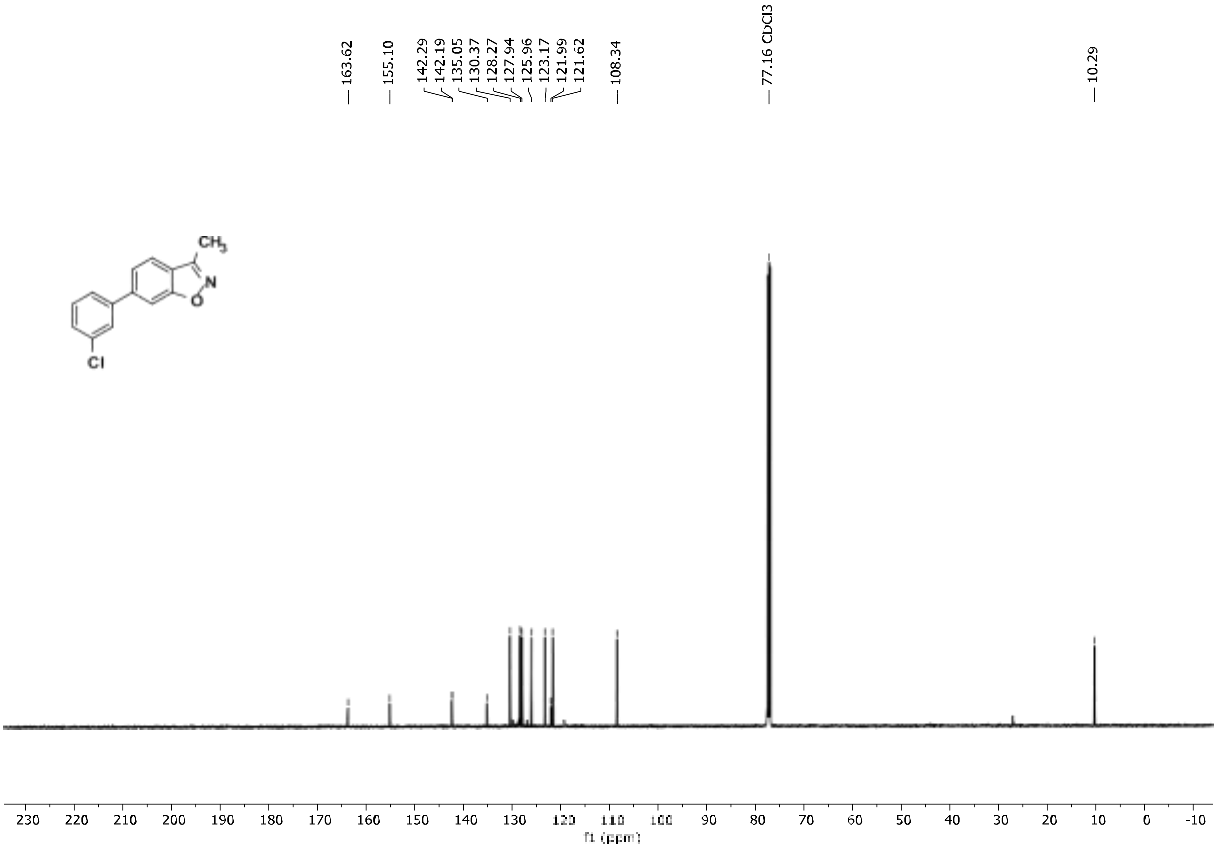

*6-(4-Chlorophenyl)-3-methylbenzo[d]isoxazole (**3j**)*

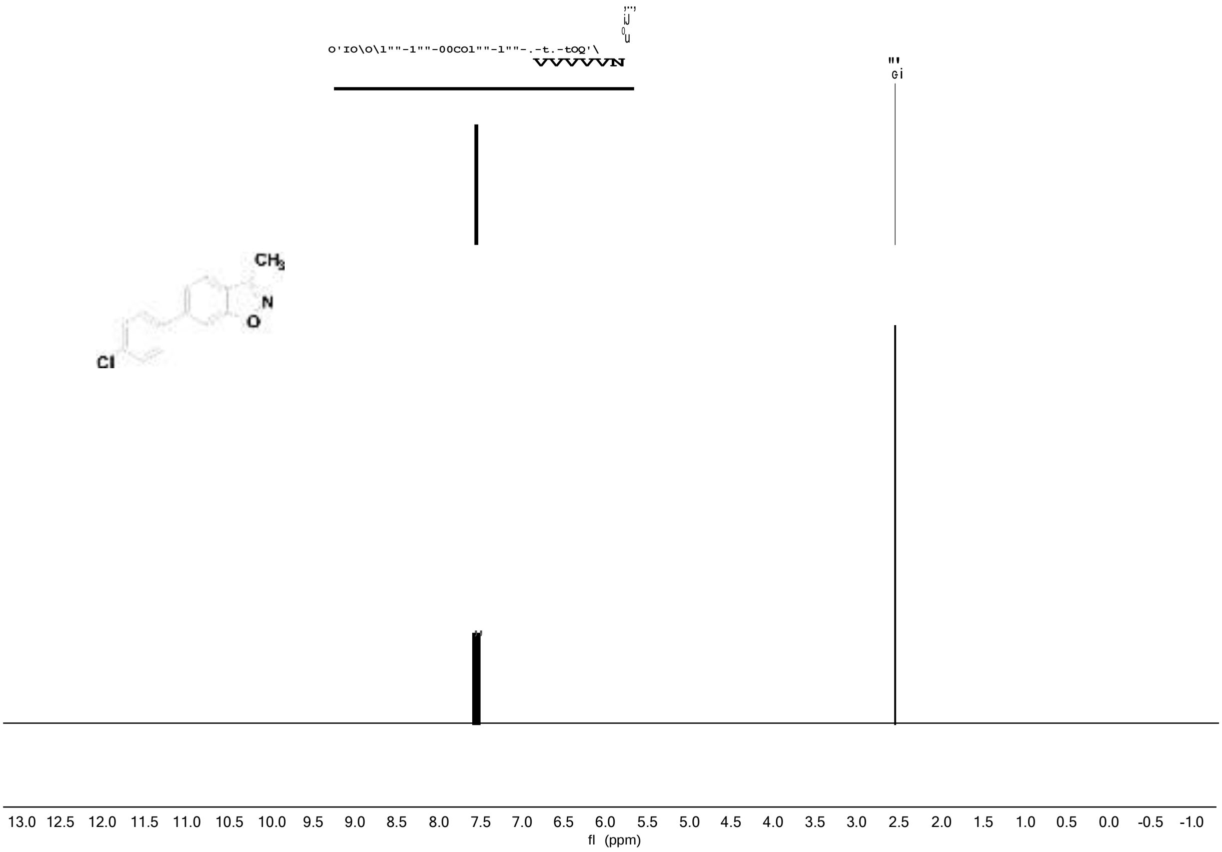

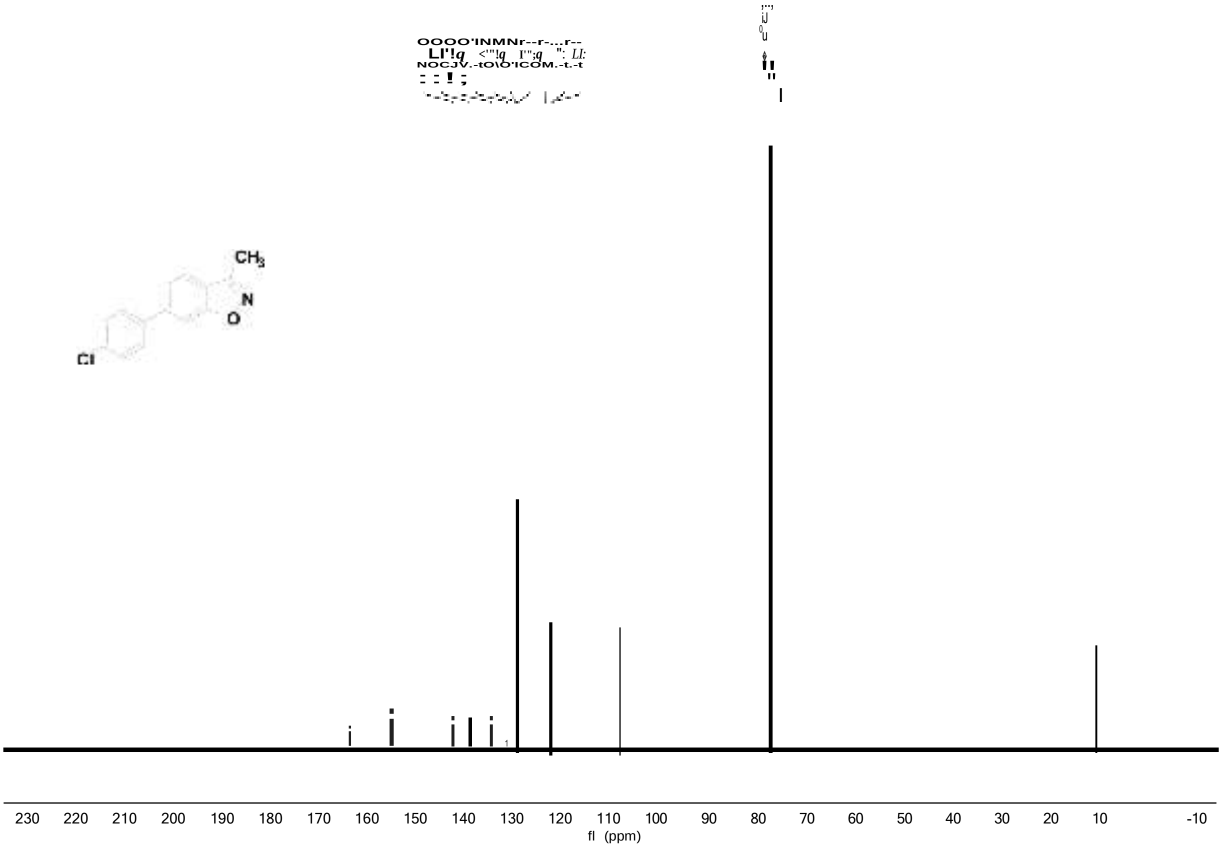

3-Methyl-6-(2-(trifluoromethyl)phenyl)benzo[d]isoxazole (**3k**)

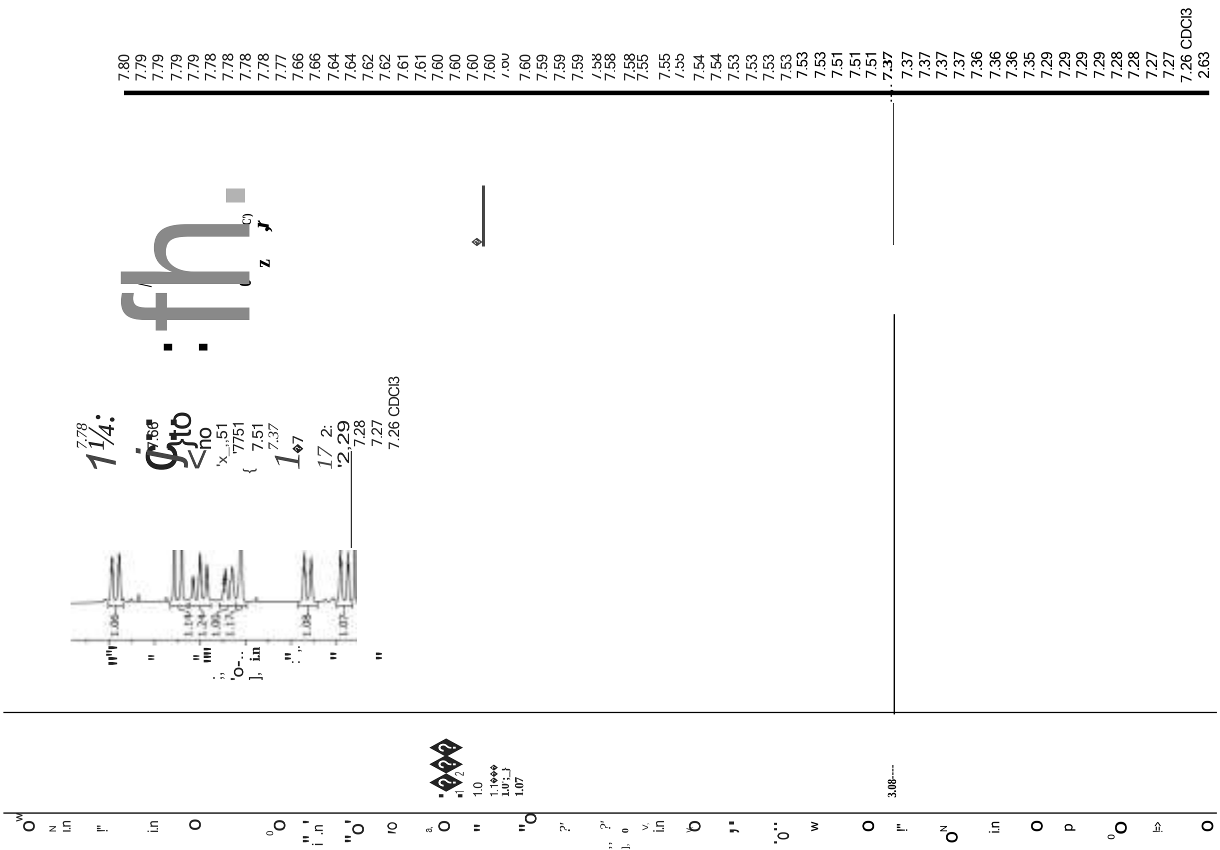

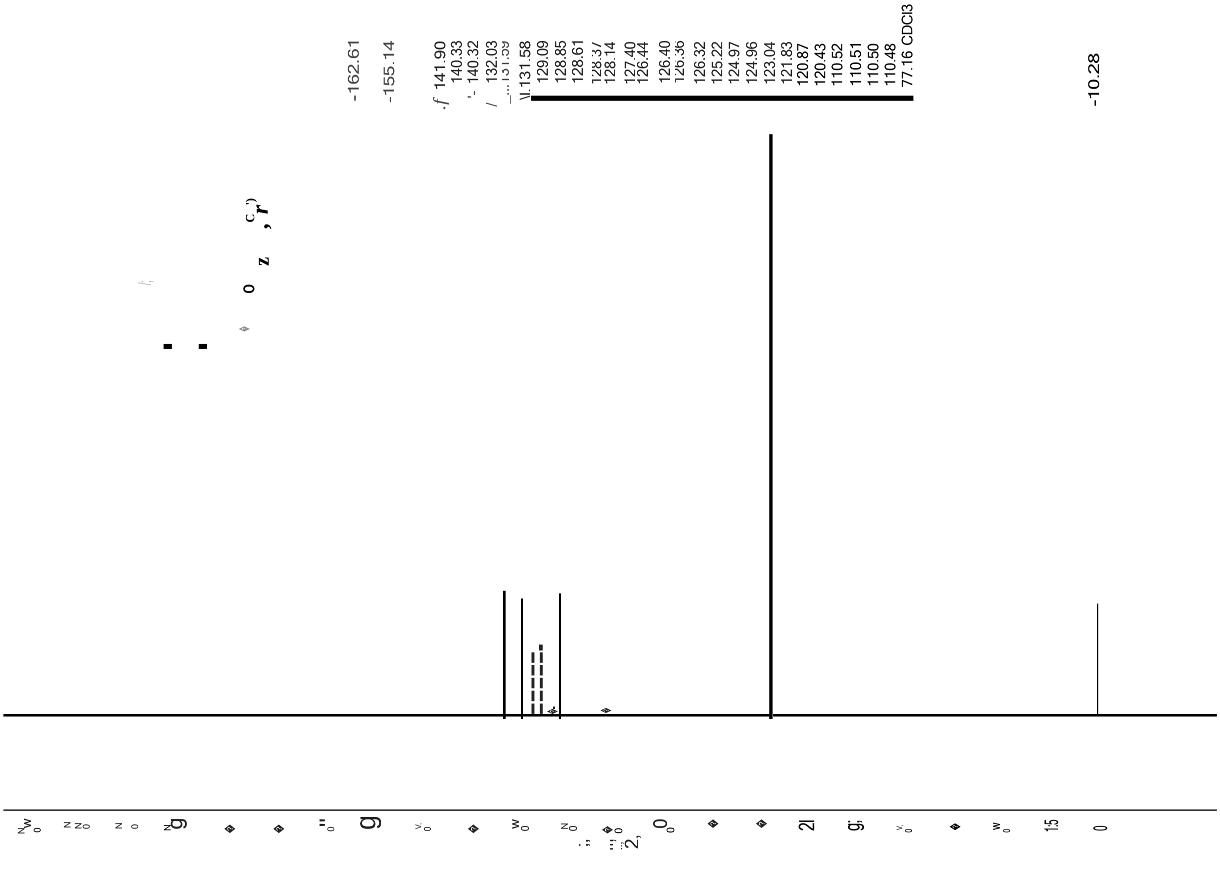

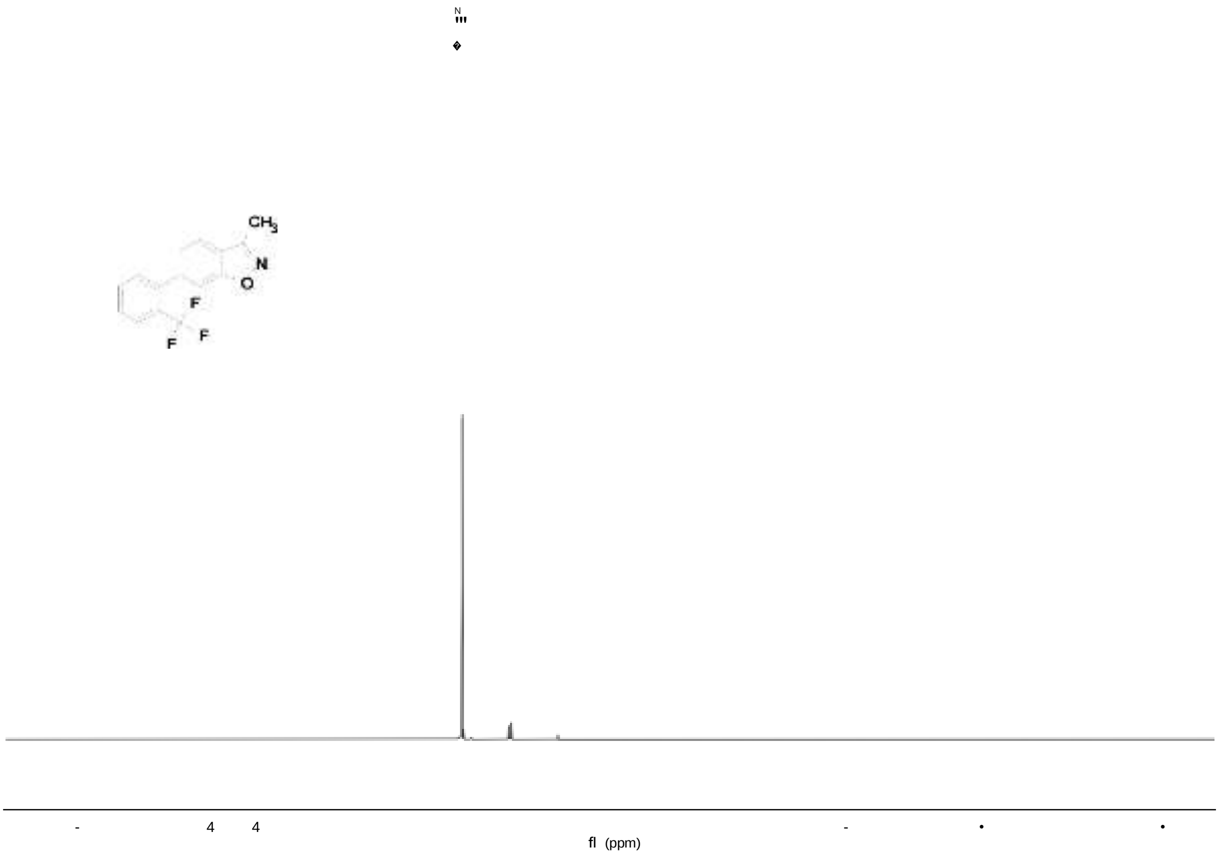

*3-Methyl-6-(phenylethynyl)benzo[d]isoxazole (**3l**)*

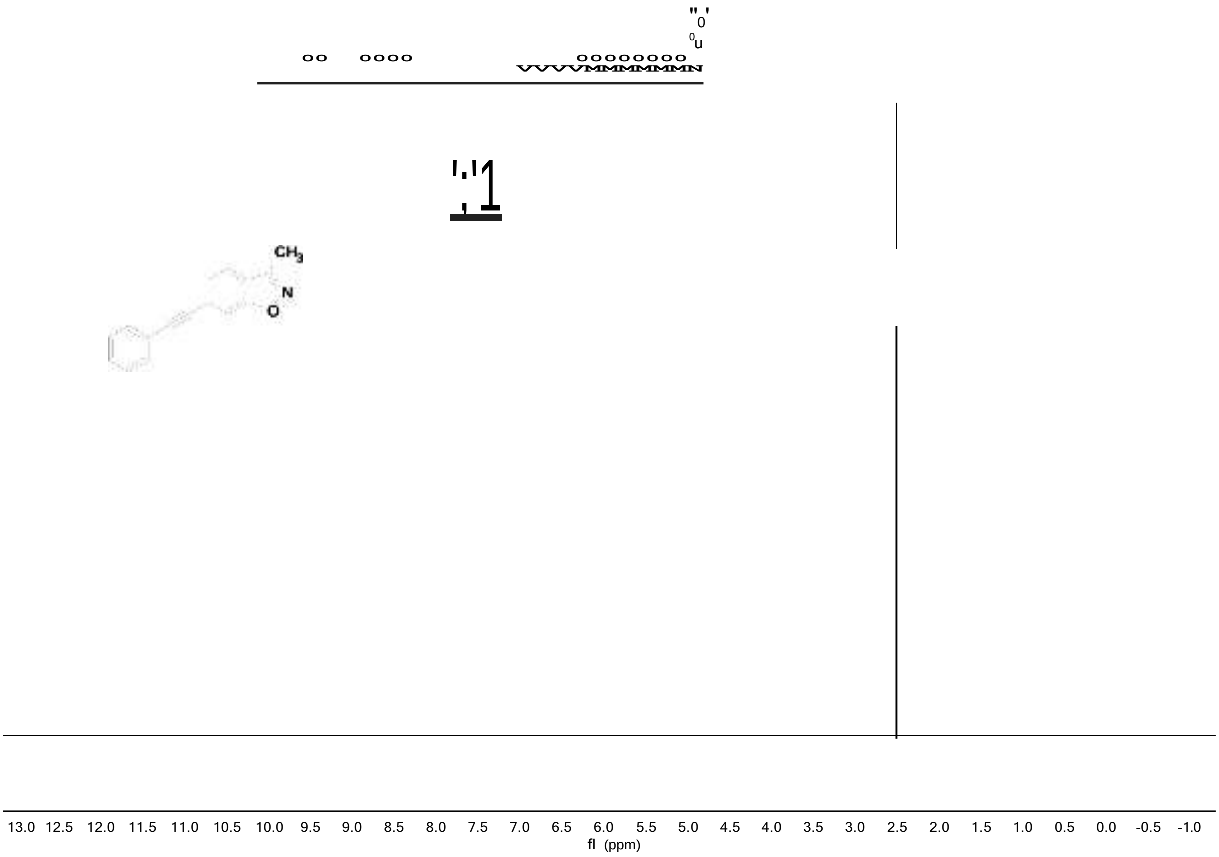

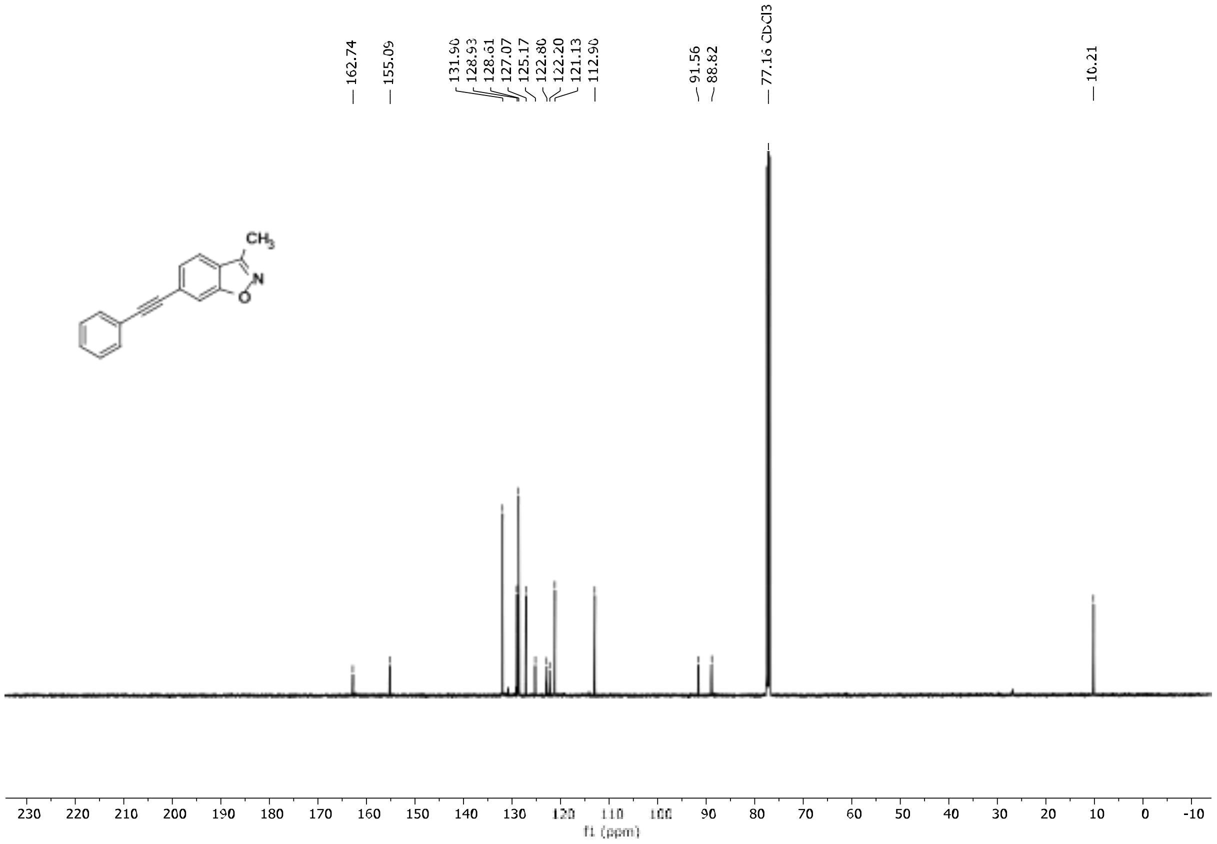

3-Methyl-5-(pyridine-3-yl)benzo[d]isoxazole ***(3m)***

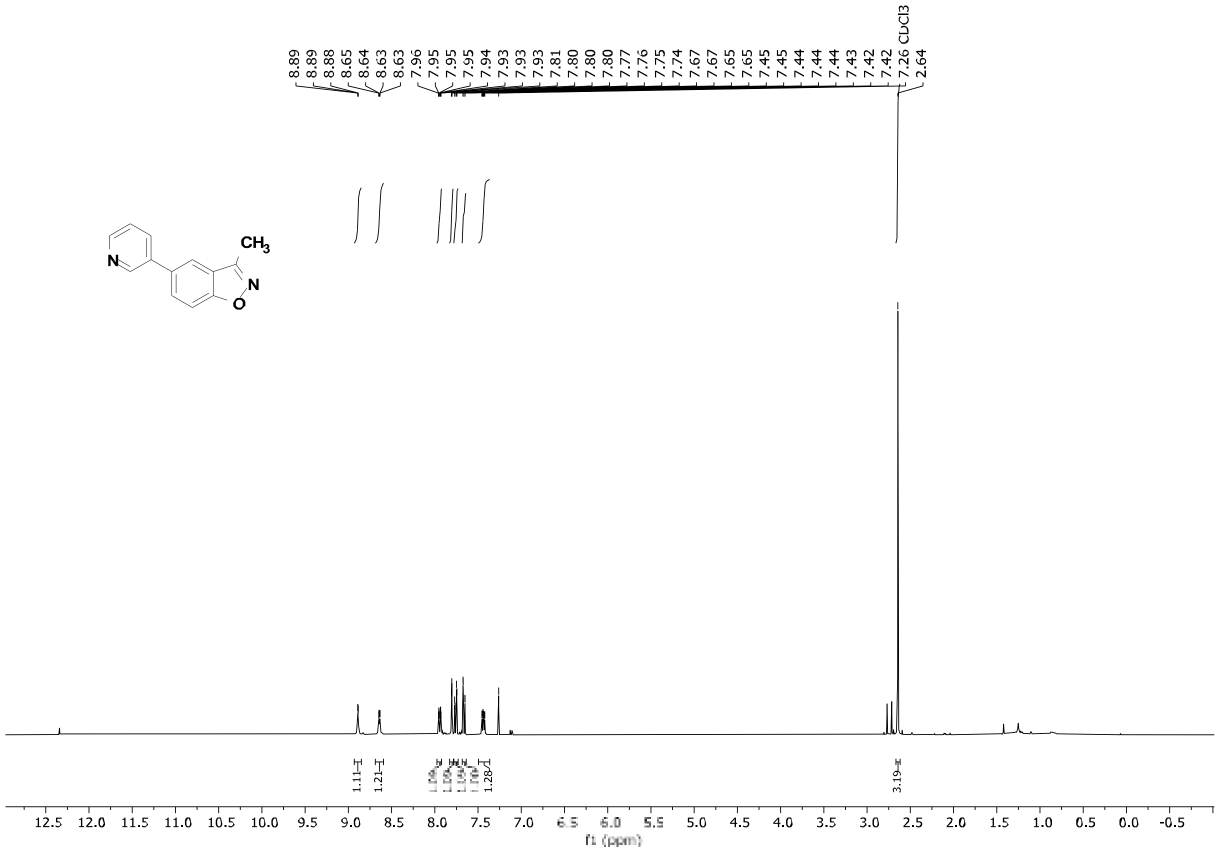

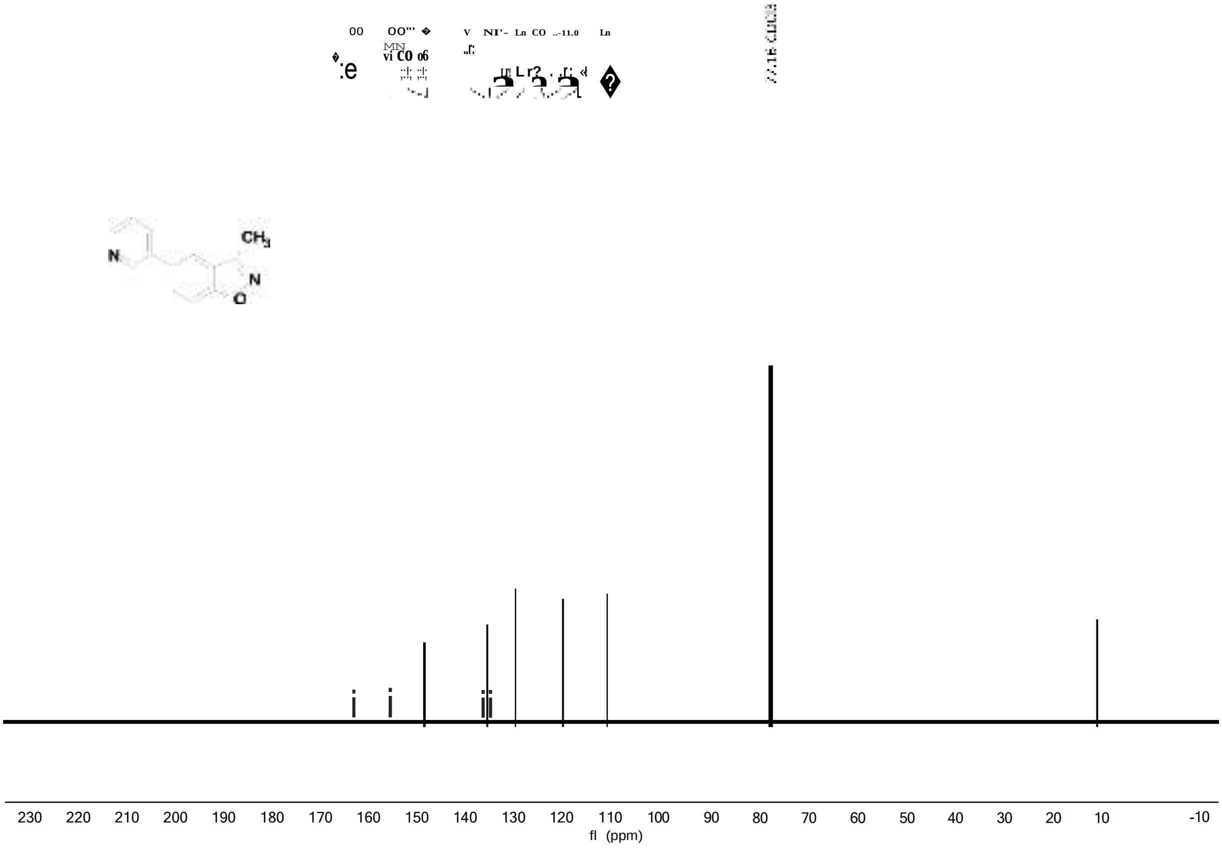

*3-Methyl-6-(pyridine-3-yl)benzo[d]isoxazole (**3n**)*

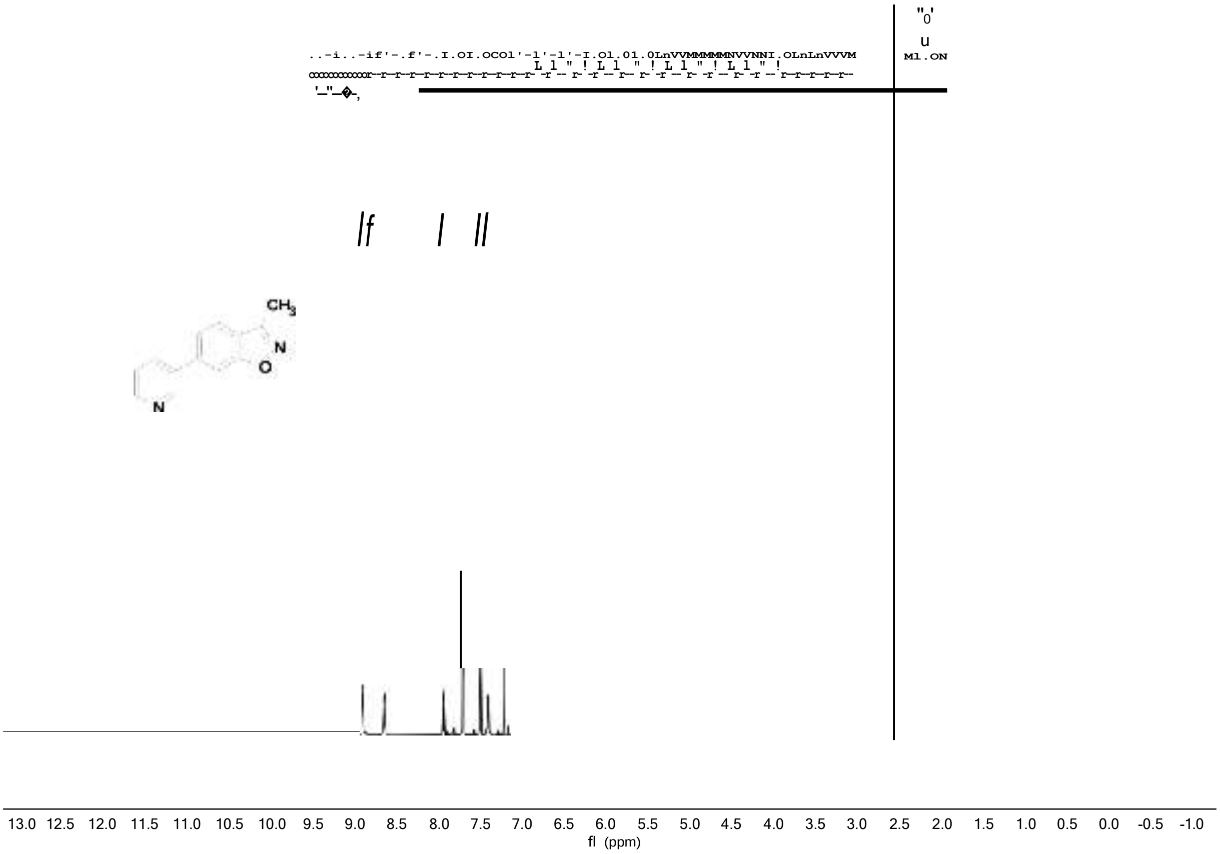

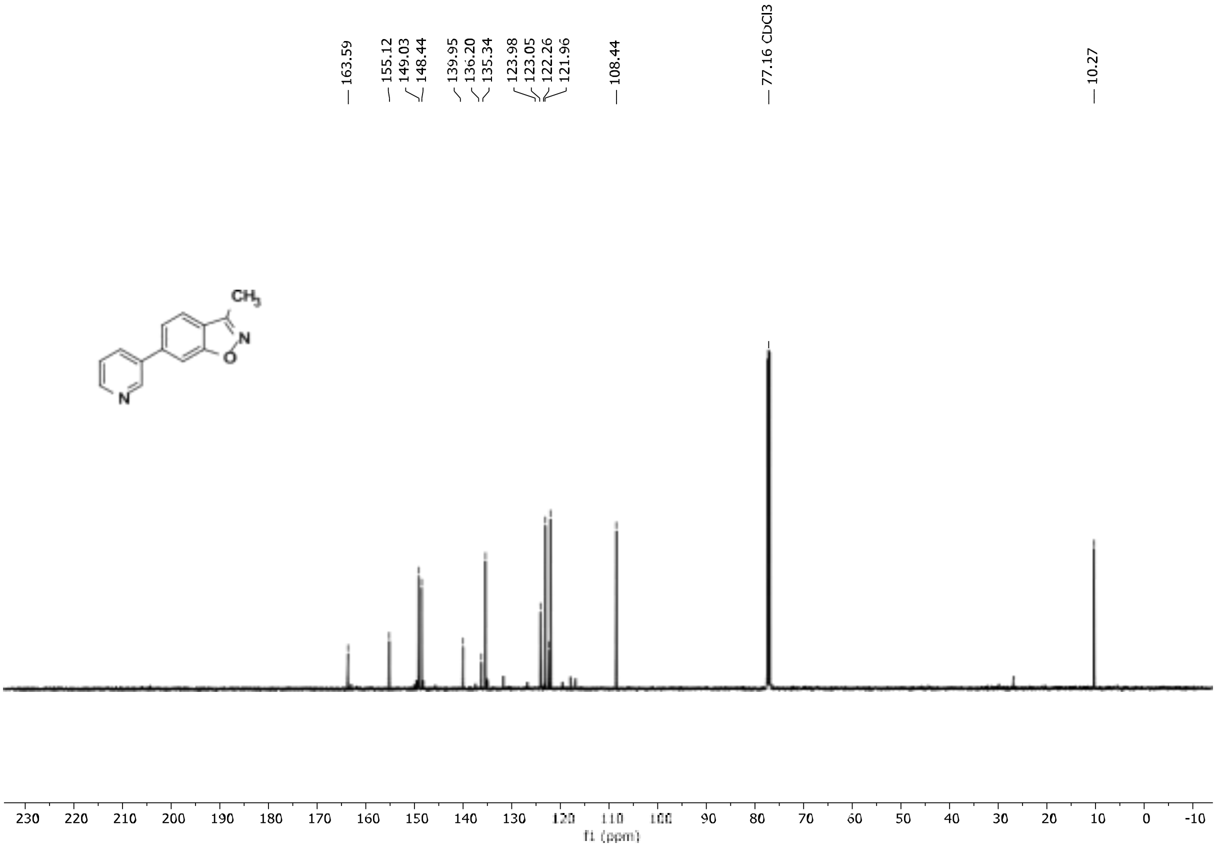

3-Methyl-5-vinylbenzo[d]isoxazole (***3o***)

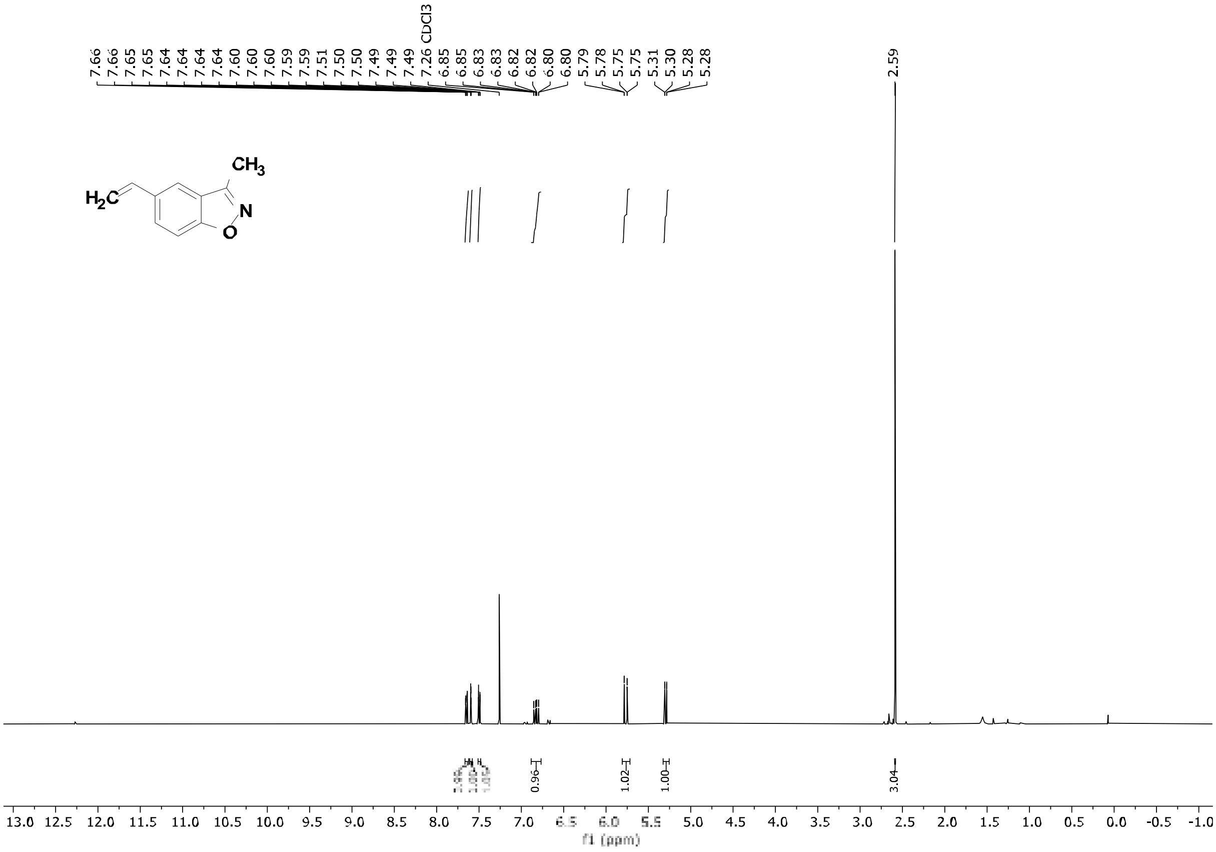

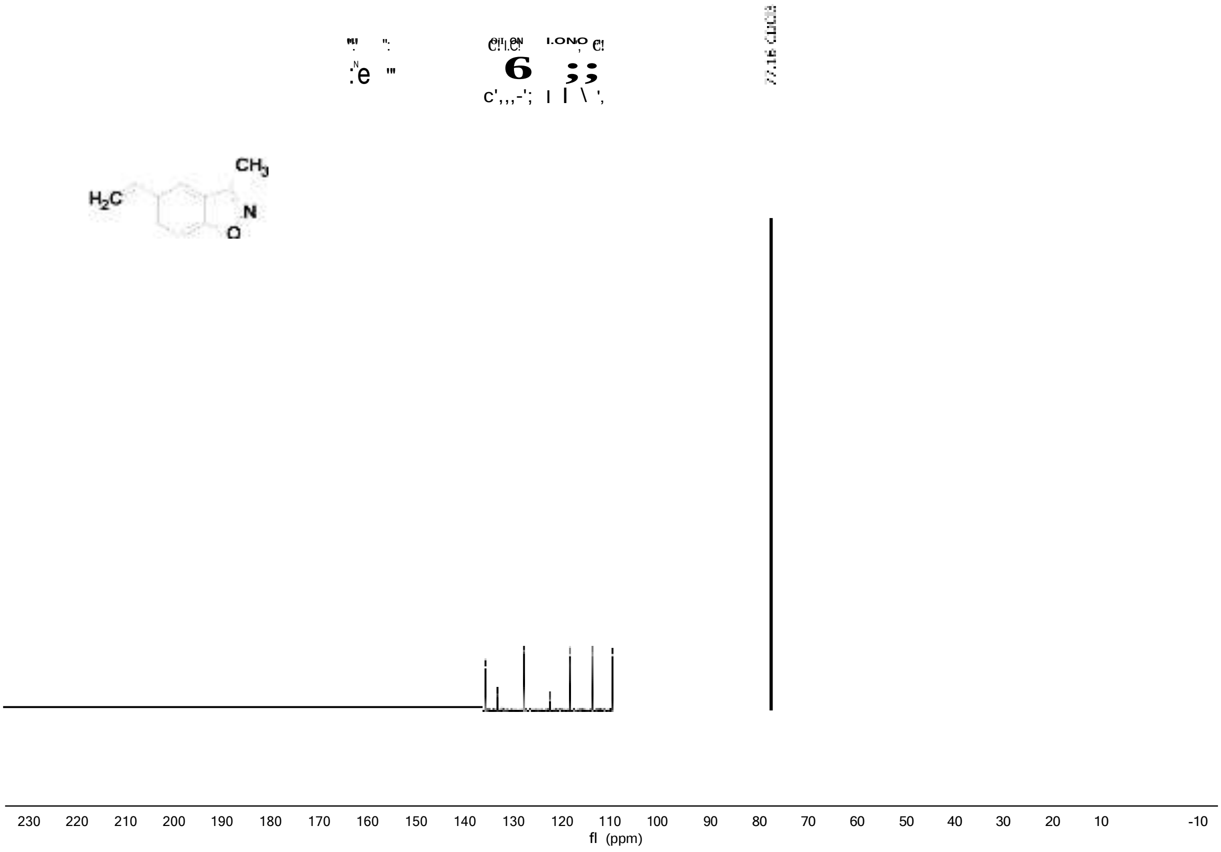

3-Methyl-6-vinylbenzo[d]isoxazole (***3p***)

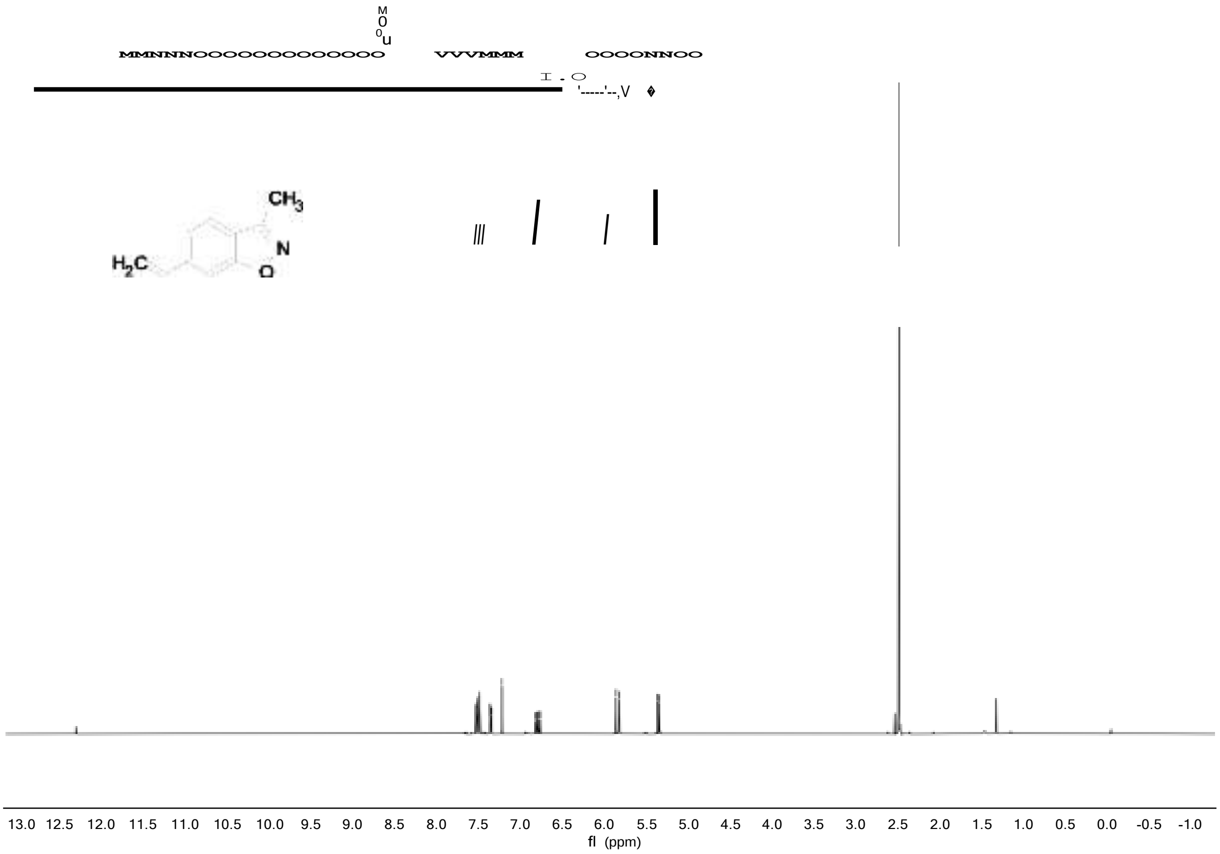

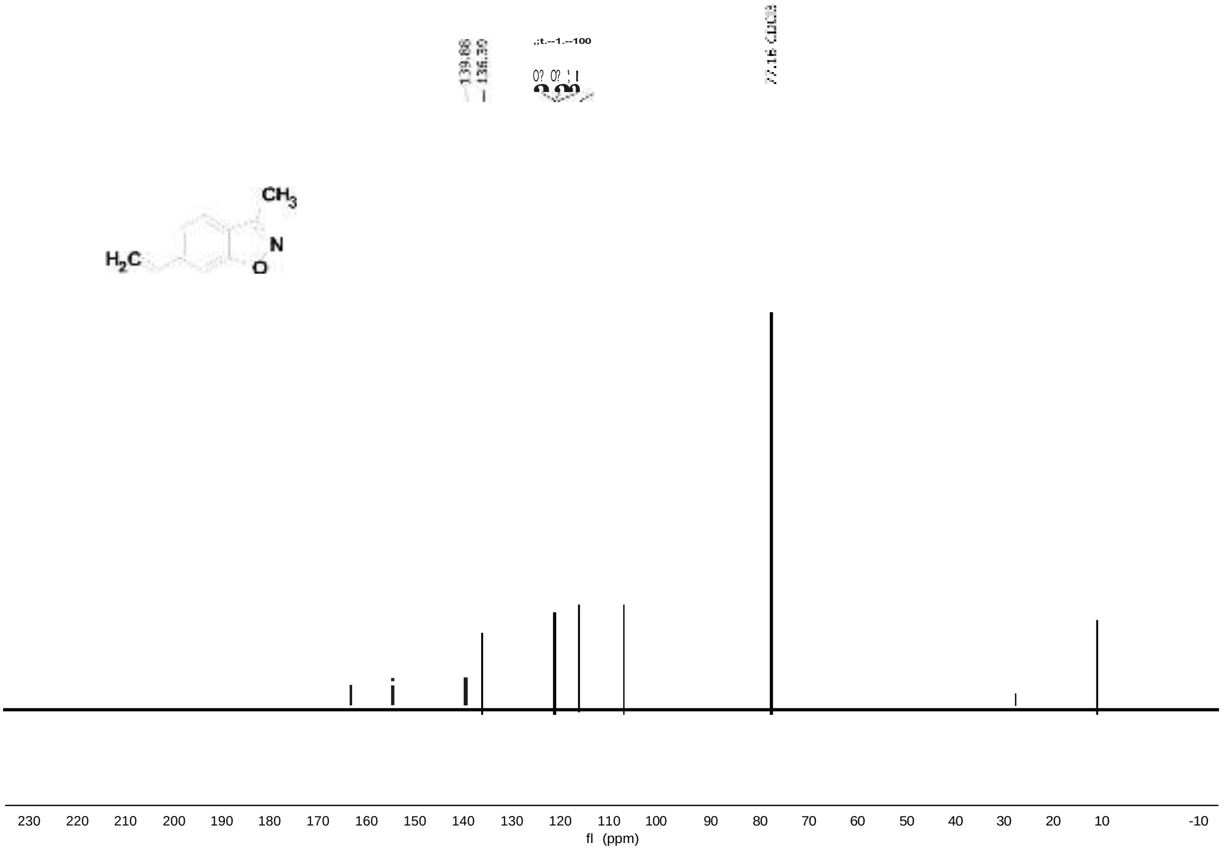
139.86
136.39
CH_3
N
O
H_2C
230 220 210 200 190 180 170 160 150 140 130 120 110 100 90 80 70 60 50 40 30 20 10 -10
fl (ppm)

Crystallographic Data

*tert-Butyl benzofuran-5-ylcarbamate (**4d**)*

CCDC 2418539

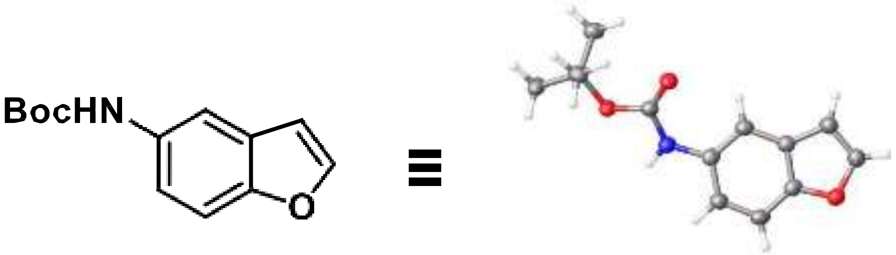

Identification code	bm281124_1_1
Empirical formula	$C_{13}H_{15}NO_3$
Formula weight	233.26
Temperature [K]	100.0(1)
Crystal system	triclinic
Space group (number)	$P1$ (2)
a [Å]	5.2939(2)
b [Å]	10.5894(3)
c [Å]	11.1328(3)
α [°]	102.318(2)
β [°]	100.925(2)
γ [°]	94.671(2)
Volume [Å^3]	593.83(3)
Z	2
ρ_{calc} [gcm^{-3}]	1.305
μ [mm^{-1}]	0.762

F(000)	248
Crystal size [mm^3]	0.039×0.065×0.168
Radiation	Cu K_α (λ=1.54184 Å)
2θ range [°]	8.32 to 149.15 (0.80 Å)
Index ranges	$-6 \le h \le 6$<br>$-13 \le k \le 12$<br>$-12 \le l \le 13$
Reflections collected	15691
Independent reflections	2304<br>R_{int} = 0.0559<br>R_{sigma} = 0.0265
Data / Restraints / Parameters	2304 / 1 / 160
Absorption correction T_{min}/T_{max} (method)	0.7390 / 1.0000 (gaussian)
Goodness-of-fit on F^2	1.029
Final *R* indexes [I≥2σ(I)]	R_1 = 0.0425<br>wR_2 = 0.1145
Final *R* indexes [all data]	R_1 = 0.0515<br>wR_2 = 0.1197
Largest peak/hole [eÅ^{-3}]	0.30/−0.18

*Methyl benzo[d]isoxazole-5-carboxylate (**5c**)*

CCDC 2418540

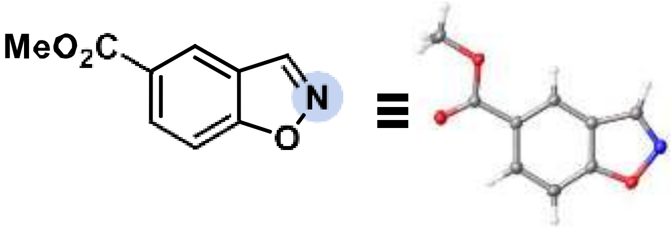

Identification code	bm291124_1_1
Empirical formula	$C_9H_7NO_3$
Formula weight	177.16
Temperature [K]	100.0(1)
Crystal system	monoclinic
Space group (number)	$P2_1/c$ (14)
a [Å]	10.4696(3)
b [Å]	11.1571(3)
c [Å]	7.1041(2)
α [°]	90
β [°]	108.043(3)
γ [°]	90
Volume [Å^3]	789.02(4)
Z	4
ρ_{calc} [gcm^{-3}]	1.491
μ [mm^{-1}]	0.963

F(000)	368
Crystal size [mm^3]	0.039×0.154×0.225
Radiation	Cu K_α (λ=1.54184 Å)
2θ range [°]	8.88 to 148.89 (0.80 Å)
Index ranges	$-13 \le h \le 13$<br>$-13 \le k \le 13$<br>$-8 \le l \le 7$
Reflections collected	14398
Independent reflections	1556<br>R_{int} = 0.0357<br>R_{sigma} = 0.0166
Data / Restraints / Parameters	1556 / 0 / 119
Absorption correction T_{min}/T_{max} (method)	0.6950 / 1.0000 (gaussian)
Goodness-of-fit on F^2	1.067
Final *R* indexes [I≥2σ(I)]	R_1 = 0.0372<br>wR_2 = 0.1013
Final *R* indexes [all data]	R_1 = 0.0414<br>wR_2 = 0.1045
Largest peak/hole [eÅ^{-3}]	0.21/−0.24

*tert-Butyl benzo[d]isoxazol-5-ylcarbamate (**5d**)*
CCDC 2418538

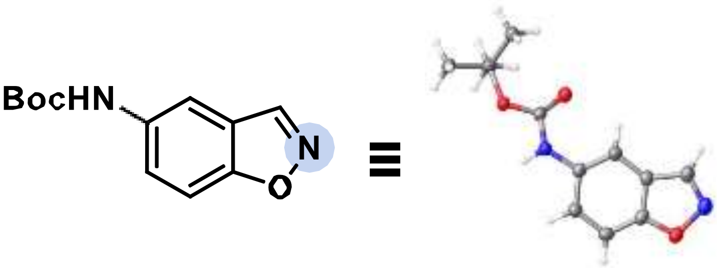

Identification code	bm301124_2_1
Empirical formula	$C_{12}H_{14}N_2O_3$
Formula weight	234.25
Temperature [K]	100.0(1)
Crystal system	triclinic
Space group (number)	$P1$ (2)
a [Å]	5.2172(2)
b [Å]	8.9168(3)
c [Å]	13.0925(4)
α [°]	79.389(3)
β [°]	78.628(3)
γ [°]	76.805(3)
Volume [Å^3]	575.14(4)
Z	2
ρ_{calc} [gcm^{-3}]	1.353
μ [mm^{-1}]	0.816

F(000)	248
Crystal size [mm^3]	0.021×0.077×0.187
Radiation	Cu K_α (λ=1.54184 Å)
2θ range [°]	6.96 to 148.78 (0.80 Å)
Index ranges	−6 ≤ h ≤ 5<br>−11 ≤ k ≤ 10<br>−16 ≤ l ≤ 16
Reflections collected	13801
Independent reflections	2244<br>R_{int} = 0.0484<br>R_{sigma} = 0.0255
Data / Restraints / Parameters	2244 / 1 / 160
Absorption correction T_{min}/T_{max} (method)	0.7370 / 1.0000 (gaussian)
Goodness-of-fit on F^2	1.038
Final *R* indexes [I≥2σ(I)]	R_1 = 0.0488<br>wR_2 = 0.1333
Final *R* indexes [all data]	R_1 = 0.0577<br>wR_2 = 0.1389
Largest peak/hole [eÅ^{-3}]	0.28/−0.23

References

[26] Rigaku, CrysAlis^{PrO}, Program for single crystal X-ray data collection and processing, **2016**.

[27] G. M. Sheldrick, *Acta Crystallogr. Sect. Found. Adv.* 2015, **71**, 3–8.

[28] G. M. Sheldrick, *Acta Crystallogr. A* 2008, **64**, 112–122.

[29] G. M. Sheldrick, *Acta Crystallogr. Sect. C Struct. Chem.* 2015, **71**, 3–8.

[30] O. V. Dolomanov, L. J. Bourhis, R. J. Gildea, J. A. K. Howard, H. Puschmann, *J. Appl. Crystallogr.* 2009, **42**, 339–341.

[31] G. Revol, T. McCallum, M. Morin, F. Gagosz, L. Barriault, *Angew. Chem. Int. Ed.* 2013, **52**, 13342–13345.

[32] A. A. Folgueiras-Amador, A. E. Teuten, M. Salam-Perez, J. E. Pearce, G. Denuault, D. Pletcher, P. J. Parsons, D. C. Harrowven, R. C. D. Brown, *Angew. Chem. Int. Ed.* 2022, **61**, e202203694.

[33] B. Michelet, C. Deldaele, S. Kajouj, C. Moucheron, G. Evano, *Org. Lett.* 2017, **19**, 3576–3579.

[34] G. J. P. Perry, J. M. Quibell, A. Panigrahi, I. Larrosa, *J. Am. Chem. Soc.* 2017, **139**, 11527–11536.

[35] M. C. Maust, C. M. Hendy, N. T. Jui, S. B. Blakey, *J. Am. Chem. Soc.* 2022, **144**, 3776–3781.

[36] G. Zhang, Y. Fu, J. Xiang, C. Guan, Z. Sang, C. Ding, *Org. Lett.* 2024, **26**, 6687–6691.

[37] P. Boehm, S. Roediger, A. Bismuto, B. Morandi, *Angew. Chem. Int. Ed.* 2020, **59**, 17887–17896.

[38] N. Sun, P. Huang, Y. Wang, W. Mo, B. Hu, Z. Shen, X. Hu, *Tetrahedron* 2015, **71**, 4835–4841.

[39] M. Petchey, A. Cuetos, B. Rowlinson, S. Dannevald, A. Frese, P. W. Sutton, S. Lovelock, R. C. Lloyd, I. J. S. Fairlamb, G. Grogan, *Angew. Chem. Int. Ed.* 2018, **57**, 11584–11588.

[40] J. Zhao, T. Shen, Z. Sun, N. Wang, L. Yang, J. Wu, H. You, Z.-Q. Liu, *Org. Lett.* 2021, **23**, 4057–4061.

[41] B. S. Cho, Y. K. Chung, *Chem. Commun.* 2015, **51**, 14543–14546.

[42] Y. Zou, G. Yue, J. Xu, J. (Steve) Zhou, *Eur. J. Org. Chem.* 2014, **2014**, 5901–5905.

[43] M. J. Moure, R. SanMartin, E. Dominguez, *Angew. Chem. Int. Ed.* 2012, **51**, 3220–3224.

[44] X. Xie, B. Chen, J. Lu, J. Han, X. She, X. Pan, *Tetrahedron Lett.* 2004, **45**, 6235–6237.

[45] S. Rashid, B. A. Bhat, G. Mehta, *Tetrahedron Lett.* 2019, **60**, 1122–1125.

[46] P. Nimnual, J. Tummatorn, C. Thongsornkleeb, S. Ruchirawat, *J. Org. Chem.* 2015, **80**, 8657–8667.

[47] M. Choi, M. K. Mehra, C. W. Lee, *Eur. J. Org. Chem.* 2023, **26**, e202201286.

[48] C.-J. Wu, X.-Y. Li, T.-R. Li, M.-Z. Shao, L.-J. Niu, X.-F. Lu, J.-L. Kan, Y. Geng, Y.-B. Dong, *J. Am. Chem. Soc.* 2022, **144**, 18750–18755.

[49] K. Matsumura, M. Ono, A. Kitada, H. Watanabe, M. Yoshimura, S. Iikuni, H. Kimura, Y. Okamoto, M. Ihara, H. Saji, *J. Med. Chem.* 2015, **58**, 7241–7257.

[50] M. Hirano, Y. Fukumoto, N. Matsubara, N. Chatani, *Chem. Lett.* 2018, **47**, 385–388.

[51] C. J. McElhinny, A. H. Lewin, S. W. Mascarella, S. Runyon, L. Brieaddy, F. I. Carroll, *Bioorg. Med. Chem. Lett.* 2012, **22**, 6661–6664.

[52] X. Hua, J. Masson-Makdissi, R. J. Sullivan, S. G. Newman, *Org. Lett.* 2016, **18**, 5312–5315.

[53] M. Novak, K. S. Rangappa, R. K. Manitsas, *J. Org. Chem.* 1993, **58**, 7813–7821.

[54] N. J. Taylor, E. Emer, S. Preshlock, M. Schedler, M. Tredwell, S. Verhoog, J. Mercier, C. Genicot, V. Gouverneur, *J. Am. Chem. Soc*. 2017, **139**, 8267–8276.

[55] X. Zhang, R. Huang, J. Marrot, V. Coeffard, Y. Xiong, *Tetrahedron* 2015, **71**, 700–708.

[56] J. J. Crawford, P. W. Kenny, J. Bowyer, C. R. Cook, J. E. Finlayson, C. Heyes, A. J. Highton, J. A. Hudson, A. Jestel, S. Krapp, S. Martin, P. A. MacFaul, B. P. McDermott, T. M. McGuire, A. D. Morley, J. J. Morris, K. M. Page, L. R. Ribeiro, H. Sawney, S. Steinbacher, C. Smith, A. G. Dossetter, *J. Med. Chem.* 2012, **55**, 8827–8837.

[57] M. Zhang, Y. Zhang, M. Song, X. Xue, J. Wang, C. Wang, C. Zhang, C. Li, Q. Xiang, L. Zou, X. Wu, C. Wu, B. Dong, W. Xue, Y. Zhou, H. Chen, D. Wu, K. Ding, Y. Xu, *J. Med. Chem.* 2018, **61**, 3037–3058.

[58] D. Pooranchand, J. Satyanarayana, H. Ila, H. Junjappa, *Synthesis* 1993, **1993**, 241–244.

[59] C.-y Chen, T. Andreani, H. Li, *Org. Lett.* 2011, **13**, 6300-6303.

[60] M. Guillén, M. Leutzsch, B. List, *J. Am. Chem. Soc*., 2024, **146**, 32292–32297.
